# Comparative efficacy and acceptability of psychosocial interventions for individuals with cocaine and amphetamine addiction: A systematic review and network meta-analysis

**DOI:** 10.1371/journal.pmed.1002715

**Published:** 2018-12-26

**Authors:** Franco De Crescenzo, Marco Ciabattini, Gian Loreto D’Alò, Riccardo De Giorgi, Cinzia Del Giovane, Carolina Cassar, Luigi Janiri, Nicolas Clark, Michael Joshua Ostacher, Andrea Cipriani

**Affiliations:** 1 Department of Psychiatry, University of Oxford, Oxford, United Kingdom; 2 Oxford Health NHS Foundation Trust, Warneford Hospital, Oxford, United Kingdom; 3 Institute of Psychiatry and Clinical Psychology, Catholic University of the Sacred Heart, Rome, Italy; 4 School of Hygiene and Preventive Medicine, University of Rome Tor Vergata, Rome, Italy; 5 Institute of Primary Health Care (BIHAM), University of Bern, Bern, Switzerland; 6 Department of Dynamic and Clinical Psychology, Sapienza University of Rome, Rome, Italy; 7 Mental Health and Substance Abuse, World Health Organization, Geneva, Switzerland; 8 Department of Psychiatry and Behavioral Sciences, Stanford University School of Medicine, Stanford, California, United States of America; 9 Department of Psychiatry, VA Palo Alto Health Care System, Palo Alto, California, United States of America; University of New South Wales, AUSTRALIA

## Abstract

**Background:**

Clinical guidelines recommend psychosocial interventions for cocaine and/or amphetamine addiction as first-line treatment, but it is still unclear which intervention, if any, should be offered first. We aimed to estimate the comparative effectiveness of all available psychosocial interventions (alone or in combination) for the short- and long-term treatment of people with cocaine and/or amphetamine addiction.

**Methods and findings:**

We searched published and unpublished randomised controlled trials (RCTs) comparing any structured psychosocial intervention against an active control or treatment as usual (TAU) for the treatment of cocaine and/or amphetamine addiction in adults. Primary outcome measures were efficacy (proportion of patients in abstinence, assessed by urinalysis) and acceptability (proportion of patients who dropped out due to any cause) at the end of treatment, but we also measured the acute (12 weeks) and long-term (longest duration of study follow-up) effects of the interventions and the longest duration of abstinence. Odds ratios (ORs) and standardised mean differences were estimated using pairwise and network meta-analysis with random effects. The risk of bias of the included studies was assessed with the Cochrane tool, and the strength of evidence with the Grading of Recommendations Assessment, Development and Evaluation (GRADE) approach. We followed the PRISMA for Network Meta-Analyses (PRISMA-NMA) guidelines, and the protocol was registered in PROSPERO (CRD 42017042900). We included 50 RCTs evaluating 12 psychosocial interventions or TAU in 6,942 participants. The strength of evidence ranged from high to very low. Compared to TAU, contingency management (CM) plus community reinforcement approach was the only intervention that increased the number of abstinent patients at the end of treatment (OR 2.84, 95% CI 1.24–6.51, *P* = 0.013), and also at 12 weeks (OR 7.60, 95% CI 2.03–28.37, *P* = 0.002) and at longest follow-up (OR 3.08, 95% CI 1.33–7.17, *P* = 0.008). At the end of treatment, CM plus community reinforcement approach had the highest number of statistically significant results in head-to-head comparisons, being more efficacious than cognitive behavioural therapy (CBT) (OR 2.44, 95% CI 1.02–5.88, *P* = 0.045), non-contingent rewards (OR 3.31, 95% CI 1.32–8.28, *P* = 0.010), and 12-step programme plus non-contingent rewards (OR 4.07, 95% CI 1.13–14.69, *P* = 0.031). CM plus community reinforcement approach was also associated with fewer dropouts than TAU, both at 12 weeks and the end of treatment (OR 3.92, *P* < 0.001, and 3.63, *P* < 0.001, respectively). At the longest follow-up, community reinforcement approach was more effective than non-contingent rewards, supportive-expressive psychodynamic therapy, TAU, and 12-step programme (OR ranging between 2.71, *P* = 0.026, and 4.58, *P* = 0.001), but the combination of community reinforcement approach with CM was superior also to CBT alone, CM alone, CM plus CBT, and 12-step programme plus non-contingent rewards (ORs between 2.50, *P* = 0.039, and 5.22, *P* < 0.001). The main limitations of our study were the quality of included studies and the lack of blinding, which may have increased the risk of performance bias. However, our analyses were based on objective outcomes, which are less likely to be biased.

**Conclusions:**

To our knowledge, this network meta-analysis is the most comprehensive synthesis of data for psychosocial interventions in individuals with cocaine and/or amphetamine addiction. Our findings provide the best evidence base currently available to guide decision-making about psychosocial interventions for individuals with cocaine and/or amphetamine addiction and should inform patients, clinicians, and policy-makers.

## Abstract 2

Using network meta-analysis, Andrea Cipriani and colleagues compare individual and combined psychosocial interventions aimed at treating cocaine and amphetamine addictions, to identify which strategies are acceptable to patients and effective, both short and long-term.

## Author summary

**Why was this study done?:**

- Cocaine and amphetamines are the most commonly abused stimulants in people aged 15–64 years, and they are linked to significant physical and mental illness as well as a substantial burden for society.
- Currently, clinical guidelines recommend the use of psychosocial interventions as first-line treatment for people with cocaine and/or amphetamine addiction.
- However, it is still unclear which psychosocial intervention, if any, is the most effective treatment for such patients.

**What did the researchers do and find?:**

- We used network meta-analysis to analyse 50 clinical studies (6,943 participants) on 12 different psychosocial interventions for cocaine and/or amphetamine addiction.
- We found that the combination of 2 different psychosocial interventions, namely contingency management and community reinforcement approach, was the most efficacious and most acceptable treatment both in the short and long term.

**What do these findings mean?:**

- To our knowledge, this is the best evidence base to guide decision-making about psychosocial interventions for cocaine and/or amphetamine addiction. Clinical guidelines should be updated to reflect these results, and policy-makers are encouraged to invest accordingly.
- Future trials should use contingency management plus community reinforcement approach as the reference treatment.

## Introduction

Drug use disorders are the 15th leading cause of disability-adjusted life years in high-income countries [1]. Cocaine and amphetamines are the most commonly abused stimulants in people aged 15–64 years, with an annual prevalence of misuse of 0.38% and 1.20%, respectively [2]. Patients addicted to stimulants experience a range of psychological and physical sequelae including psychosis and other mental illnesses, neurological disorders and cognitive deficits, cardiovascular dysfunctions, sexually transmitted diseases, and blood-borne viral infections such as HIV and hepatitis B and C [3], and are at increased risk of all-cause mortality [4]. Moreover, the social burden of stimulant abuse is worsened by its association with crime, violence, and sexual abuse [5].

Currently, international clinical guidelines recommend the use of psychosocial interventions for cocaine and/or amphetamine addiction as first-line treatment, and there is little evidence supporting pharmacotherapy or brain stimulation treatments [6–8]. In the absence of approved pharmacotherapies, several structured psychosocial and self-help approaches are available, such as contingency management (CM) (a behavioural approach that consists in providing stimulant users with rewards upon drug-free urine samples), community reinforcement approach (a multi-layered intervention involving functional analysis, coping-skills training, and social, familial, recreational, and vocational reinforcements), and 12-step programme (a set of guiding principles outlining a course of action for self-help recovery from addiction) [8]. International guidelines are unclear on whether any specific intervention should be considered first [9,10]; for example, the National Institute for Health and Care Excellence (NICE) recommends CM alone, cognitive behavioural therapy (CBT) alone, or self-help groups based on 12-step programme alone for the treatment of individuals with stimulant use disorders [8]. However, a recent systematic review showed that CM and CBT were well accepted and moderately efficacious at the end of treatment, but not at follow-up after treatment completion [11].

Previous pairwise meta-analyses relied on a limited number of studies with direct comparisons between different interventions [12,13]. From a clinical perspective, it is important to assess whether psychosocial interventions are effective and acceptable in both the short and long term, and also whether the combination of 2 approaches can produce a significant benefit. We therefore performed a network meta-analysis to compare and rank the efficacy and acceptability of individual and combined psychosocial interventions for the treatment of cocaine and/or amphetamine addiction at different time-points.

## Methods

### Search strategy and selection criteria

This network meta-analysis was conducted following a pre-established protocol registered on PROSPERO (CRD 42017042900), and is reported according to the PRISMA for Network Meta-Analyses (PRISMA-NMA) guidelines [14]. We searched the Cochrane Drugs and Alcohol Group Specialised Register, PubMed, Embase, CINAHL, ISI Web of Science, and PsycINFO from the date of database inception to 8 April 2018. We also screened international registers, hand-searched the reference lists of retrieved articles, and looked at key conference proceedings (for the full search strategy, see S1 Text). When needed, we contacted the investigators and relevant trial authors to obtain information about unpublished or incomplete trials. All searches included non-English language literature.

We included randomised controlled trials (RCTs) comparing any structured psychosocial intervention against a control intervention—another psychosocial intervention or treatment as usual (TAU)—for the treatment of individuals with cocaine and/or amphetamine addiction, according to the Diagnostic and Statistical Manual of Mental Disorders (DSM) III, III-R, IV, IV-TR, or V or the International Classification of Diseases–10th revision (ICD-10) criteria. CBT, CM, community reinforcement approach, meditation-based therapies, non-contingent rewards, supportive-expressive psychodynamic therapy, 12-step programme, and their combinations were all identified as structured psychosocial interventions. We excluded studies on occasional users not actively seeking treatment and RCTs with study duration less than 4 weeks. We did not exclude studies on individuals with a comorbid substance use disorder (including opioid, alcohol, or cannabis use) or with a comorbid psychiatric disorder.

### Data extraction and quality assessment

Four authors (FDC, GLD, MC, RDG) independently screened the references retrieved by the search, selected the studies, and extracted the data, using a predefined data-extraction sheet. The same reviewers discussed any uncertainty regarding study eligibility and data extraction until consensus was reached; conflicts of opinion were resolved with another member of the review team (AC). 2 authors (GLD, MC) independently assessed the risk of bias of the included studies with the Cochrane tool [15], Three authors (FDC, CDG, AC) used the Grading of Recommendations Assessment, Development and Evaluation (GRADE) approach [16], through the Confidence in Network Meta-Analysis Software (CINeMA) [17], to evaluate the strength of evidence for results at the end of treatment from the network meta-analysis (S2 Text).

### Outcomes

We considered as primary outcomes the efficacy and the acceptability of the interventions at the end of treatment [18]. Efficacy was measured as the proportion of individuals abstinent (assessed by urinalysis), and acceptability as the proportion of individuals who dropped out from the study due to any cause. As secondary outcomes, we also measured efficacy and acceptability at 12 weeks from the start of treatment and at the longest follow-up (with follow-up starting at the end of treatment, independent of the duration of the intervention). If 12-week data were not available, we used data ranging between 4 and 20 weeks (giving preference to the time-point closest to 12 weeks). Other secondary outcomes were the longest duration of abstinence measured both at 12 weeks and at the end of treatment.

### Statistical analysis

We performed first pairwise meta-analyses using a random-effects model to estimate pooled odds ratios (ORs) for dichotomous outcomes and standardised mean differences (SMDs) for continuous outcomes with their 95% confidence intervals (CIs) using STATA [19]. We assessed statistical heterogeneity in each pairwise comparison with τ^2^, *I*^2^ statistic, and *P* value [15]. If at least 10 studies were available, we used the funnel plot and Egger’s test to detect publication bias [15].

We incorporated indirect comparisons with direct comparisons using random-effects network meta-analyses within a frequentist framework using STATA (network package), and results are presented with the network graphs package [20]. We report the results of network meta-analyses for both primary and secondary outcomes in league tables with effect sizes (OR or SMD) and their 95% CIs. When dichotomous outcome data were missing, we assumed that patients who dropped out after randomisation had a negative outcome. Missing continuous outcome data were analysed on an endpoint basis, including only participants with a final assessment, as reported by the original study authors. We also calculated the number needed to treat (NNT), which is the number of patients that need to be treated in order for 1 to benefit from the intervention compared with TAU.

We assessed incoherence between direct and indirect sources of evidence using local and global approaches. Coherence (or consistency) is an important assumption to check in network meta-analyses because it is the manifestation of transitivity in the data from a network of interventions: coherence exists when treatment effects from direct and indirect evidence are in agreement (subject to the usual variation due to heterogeneity in the direct evidence) [21]. Local incoherence was measured by using a loop-specific approach (which identified inconsistent loops of evidence) [22] and a side-splitting approach (which separated evidence on a particular comparison into direct and indirect evidence) [23]. Global incoherence was measured with the between-studies standard deviation (SD) (heterogeneity parameter) by using both a coherence and incoherence model and by measuring the chi-squared incoherence, with its *P* value. We estimated the presence of publication bias by plotting comparison-adjusted funnel plots for the network meta-analyses with a linear regression line [24]. We also estimated the ranking probabilities for all treatments, i.e., their probability of being at each possible rank for each intervention. We report the treatment hierarchy as the surface under the cumulative ranking curve (SUCRA) and as the mean rank (S2 Text) [24].

To determine whether the results were affected by study characteristics, we performed subgroup network meta-analyses for abstinence and dropout at the end of treatment according to the following variables: year of publication, sex ratio, mean age group, intensity of the treatment, type of stimulant, risk of bias, opioid therapy, sample size, and comorbid alcohol misuse. Additionally, we performed sensitivity network meta-analyses for the primary outcomes by considering (a) only trials on individuals addicted to cocaine and no other stimulant and (b) only trials on individuals addicted to stimulants and on opioid substitution therapy.

## Results

**Fig 1:**
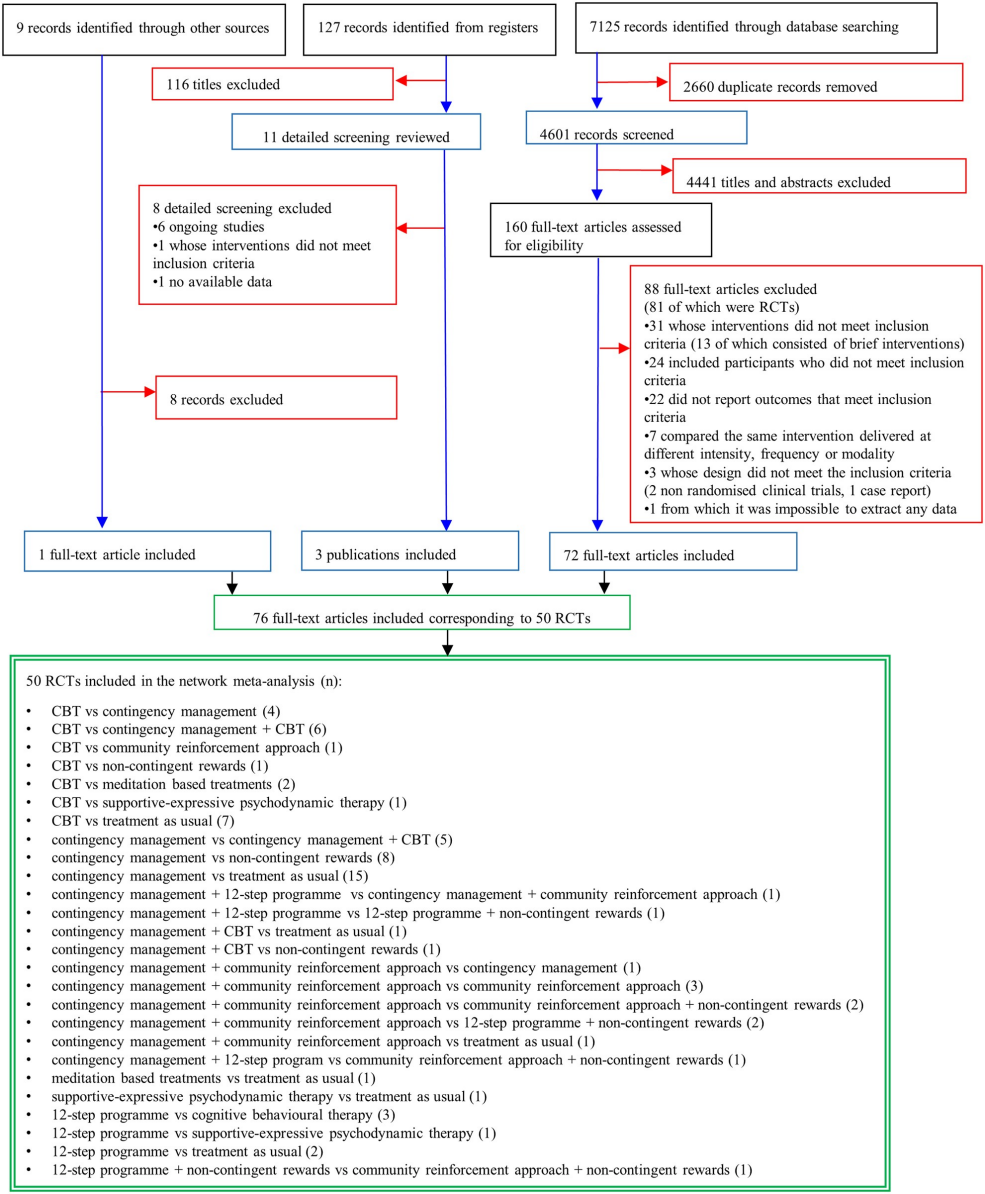
Study selection. The flowchart shows the records identified through database searching (black boxes), the records screened (blue boxes), the records excluded (red boxes), and the studies included (green boxes). CBT, cognitive behavioural therapy; RCT, randomised controlled trial.

**Fig 2:**
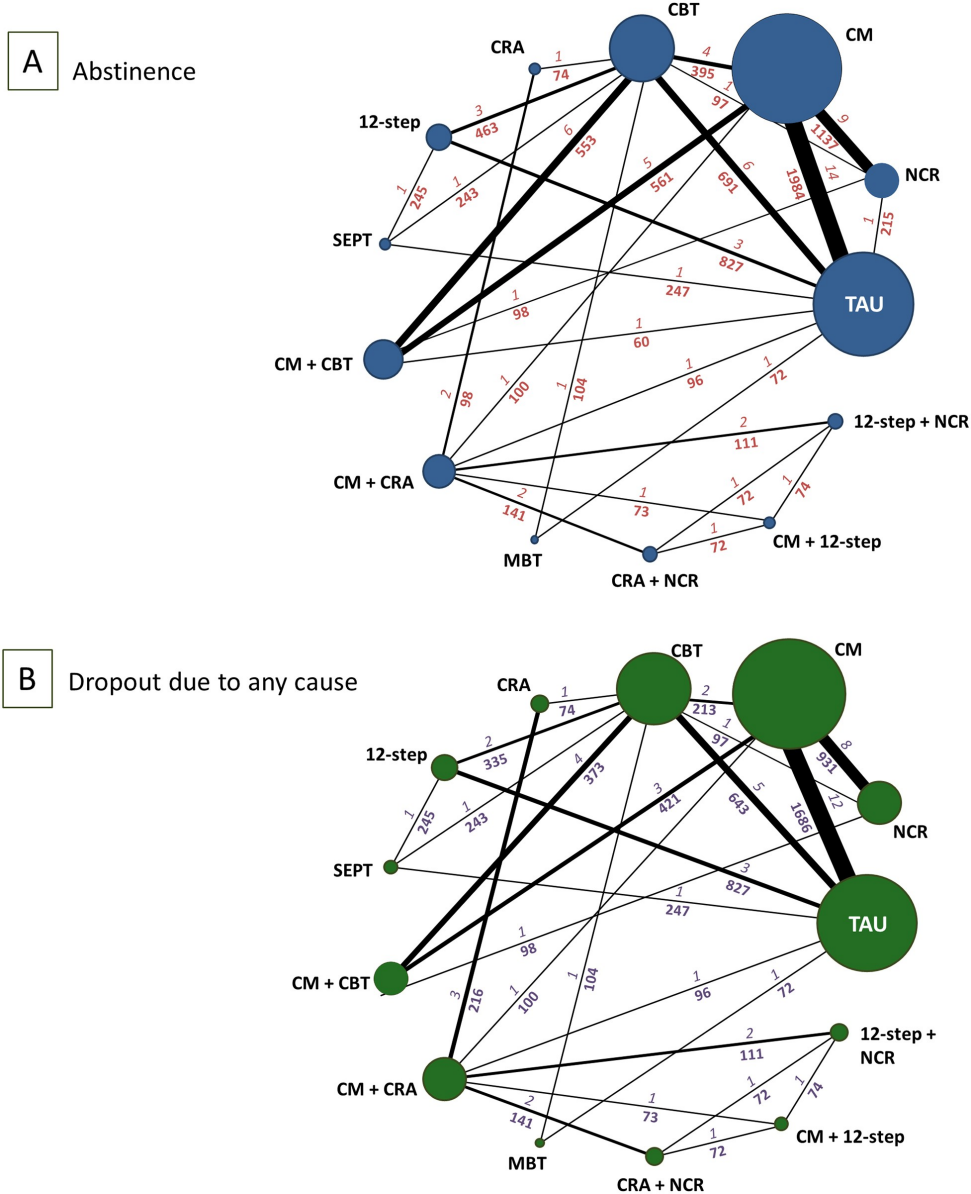
Network of eligible comparisons for abstinence and dropout due to any cause at the end of treatment. The figure plots the network of eligible direct comparisons for abstinence at the end of treatment (46 trials) (A) and dropout due to any cause (43 studies) (B). The width of the lines is proportional to the number of trials comparing every pair of treatments, and the size of every node is proportional to the number of randomised participants. The numbers above each connection relate to the numbers of trials and the numbers below each connection relate to the number of patients for each direct comparison. 12-step, 12-step programme; CBT, cognitive behavioural therapy; CM, contingency management; CRA, community reinforcement approach; MBT, meditation-based treatments; NCR, non-contingent rewards; SEPT, supportive-expressive psychodynamic therapy; TAU, treatment as usual.

From the initially identified 7,261 citations, we retrieved 160 potentially eligible articles in full text (Fig 1). We excluded 88 reports, but then included 4 additional studies (3 from trial registers and 1 from screening the references), resulting in 76 publications (S3 Text) describing 50 RCTs (6,942 participants), published between 1993 and 2016 (Fig 2; Table 1), comparing 12 psychosocial interventions or TAU (listed and defined in S4 Text). Overall, 5,158 participants were randomly assigned to psychosocial treatments, and 1,784 to TAU. Full clinical and demographic characteristics are reported in Table 1. The mean study sample size was 139 participants, ranging between 19 and 487 participants. The median duration of treatment was 12 weeks (range 6–36). Dropout rates varied between 15.1% (CM + CBT) and 60.2% (meditation-based therapies) (S1 Table). A total of 37 studies were followed up after study completion, for a mean duration of 41.4 weeks (range 16–96). A total of 42 (84%) trials recruited patients from North America, 6 from Europe, 1 from Latin America, and 1 from Oceania. About a third of the population was women (35.9%), and the mean age was 36.8 years. A total of 76% of trials (38 of 50) enrolled participants with cocaine addiction, 8% of trials (4 of 50) with amphetamine addiction, and 16% (8 of 50) with both. About one-third of trials (18 of 50) enrolled participants on methadone maintenance. The mean addiction severity was moderate/high (S2 Table). In terms of risk of bias, 22 (44%) trials were rated low risk, 13 (26%) moderate, and 15 (30%) high (S3 Table; S1 Fig).

**Table 1:** Characteristics of included randomised controlled trials.

Study, year	Country	Diagnostic criteria	Intervention (*n*)	Duration of intervention (weeks)	Follow-up (weeks)	Setting	Age mean (SD) (years)	Female *n* (%)	Stimulant abused	Comorbidities
Carroll, 1994 [25]	US	DSM-III-R	CBT (29) CBT (29) TAU (25) TAU (27)	12	NA	Outpatient	28.8 (5.8)	30 (27.3)	Cocaine	Alcohol (<50%)
Carroll, 1998 [26]	US	DSM-III-R	12-step (25) 12-step (25) CBT (26) CBT (19) TAU (27)	12	52	Outpatient	30.5 (5.5)	26 (26.9)	Cocaine	Alcohol (100%)
Carroll, 2012 [27]	US	DSM-IV	12-step^c^ (27) 12-step^d^ (29) TAU^c^ (26) TAU^d^ (30)	12	48	Outpatient	38.3 (7.6)	46 (41.1)	Cocaine	Alcohol (≥50%), Methadone (100%)
Carroll, 2014 [28]	US	DSM-IV	CBT (47) TAU (54)	8	48	Outpatient	41.2 (9.6)	61 (60.4)	Cocaine	Alcohol (<50%), Methadone (100%)
Carroll, 2016 [29]	US	DSM-IV	CBT^c^ (26) CBT^d^ (28) CBT + CM^c^ (22) CBT + CM^d^ (23)	12	48	Outpatient	39.3 (7.5)	27 (27.3)	Cocaine	Alcohol (≥50%)
Chen, 2013 [30]	US	DSM-IV	MBT (37) TAU (35)	12	NA	Outpatient	45.1 (6.5)	31 (42.9)	Cocaine	NA
Crits-Cristoph, 1999 [31]	US	DSM-IV	12-step (121) CBT (119) SEPT (124) TAU (123)	36	48	Outpatient	33.9 (6.3)	113 (23.2)	Cocaine	NA
Donovan, 2013 [32]	US	DSM-IV	12-step (234) TAU (237)	8	24	Outpatient	38.3 (9.7)	277 (58.8)	Amphetamine and cocaine	Alcohol (<50%)
Dursteler-MacFarland, 2013 [33]	Switzerland	DSM-IV	CBT^c^ (17) CBT^e^ (15) TAU^c^ (15) TAU^e^ (15)	12	NA	Outpatient	35.9 (6.1)	22 (35.6)	Cocaine	Methadone (100%)
Epstein, 2003 [34]	US	DSM-III-R	CBT (48) CBT + CM (49) CM (47) NCR (49)	12	52	Outpatient	39.0 (6.8)	90 (47.0)	Cocaine	Alcohol (<50%), Methadone (100%)
Festinger, 2014 [35]	US	DSM-IV	CM^f^ (71) CM^g^ (73) TAU (78)	12	NA	Outpatient	37.1 (9.9)	73 (32.9)	Cocaine	Methadone (100%)
Garcia-Fernandez, 2011 [36]	Spain	DSM-IV	CM + CRA (29) CRA (29)	24	48	Outpatient	29.9 (5.7)	7 (12.1)	Cocaine	Alcohol (≥50%)
Garcia-Rodriguez, 2007 [37]	Spain	DSM-IV	CM + CRA (44) TAU (52)	24	NA	Outpatient	29.1 (5.5)	9 (9.2)	Cocaine	Alcohol (≥50%)
Ghitza, 2007 [38]	US	DSM-IV	CM (76) NCR (40)	12	24	Outpatient	37.3 (8.4)	51 (44.3)	Cocaine	Methadone (100%)
Hagedorn, 2013 [39]	US	NR	CM (71) TAU (68)	8	48	Outpatient	NA	2 (1.4)	Amphetamine and cocaine	Alcohol (≥50%)
Higgins, 1993 [40]	US	DSM-III-R	12-step + NCR (19) CM + CRA (19)	24	52	Outpatient	29.3 (5.2)	0 (0)	Cocaine	Alcohol (≥50%)
Higgins, 1994 [41]	US	DSM-III-R	CM + CRA (20) CRA (20)	24	52	Outpatient	31.4 (5.1)	13 (32.5)	Cocaine	Alcohol (≥50%)
Higgins, 2000 [42]	US	DSM-III-R	CM + CRA (36) CRA + NCR (34)	24	78	Outpatient	30.4 (5.0)	19 (27.1)	Cocaine	Alcohol (≥50%)
Higgins, 2003 [43]	US	DSM-III-R	CM (51) CM + CRA (49)	24	96	Outpatient	33.9 (6.3)	41 (41.0)	Cocaine	Alcohol (≥50%)
Kirby, 1998 [44]	US	DSM-III-R	CM (44) TAU (46)	12	NA	Outpatient	NA	NA	Cocaine	NA
Landovitz, 2015 [45]	US	NR	CM (70) NCR (100)	8	24	Outpatient	36.7 (11)	0 (0)	Amphetamine and cocaine	NA
Ledgerwood, 2006 [46]	US	DSM-IV	CM (104) TAU (38)	12	NA	Outpatient	36.6 (7.3)	77 (54.3)	Cocaine	Alcohol (<50%)
Maude-Griffin, 1998 [47]	US	DSM-III-R	12-step (69) CBT (59)	12	26	Outpatient	NA	NA	Cocaine	Alcohol (≥50%)
McDonell, 2013 [48]	US	DSM-IV	CM (91) NCR (85)	12	24	Outpatient	41.7 (9.6)	61 (34.5)	Amphetamine and cocaine	Alcohol (<50%)
McKay, 1997 [49]	US	DSM-III-R	CBT (46) TAU (52)	24	NA	Outpatient	40.1 (7.1)	NA	Cocaine	Alcohol (<50%)
Menza, 2010 [50]	US	NR	CM (70) TAU (57)	6	24	Outpatient	38.7 (NA)	0 (0)	Amphetamine	NA
Miguel, 2016 [51]	Brazil	DSM-IV	CM (33) TAU (32)	12	NA	Outpatient	35.3 (8.5)	9 (14.3)	Cocaine	Alcohol (≥50%)
Milby, 2008 [52]	US	DSM-IV	CBT + CM (103) CM (103)	24	52	Outpatient	40.1 (7.2)	56 (27.2)	Cocaine	Alcohol (<50%)
Peirce, 2006 [53]	US	DSM-IV	CM (198) TAU (190)	12	24	Outpatient	42.0 (8.6)	171 (44.1)	Amphetamine and cocaine	Alcohol (<50%), Methadone (100%)
Petitjean, 2014 [54]	Switzerland	DSM-IV	CBT (31) CBT + CM (29)	24	48	Outpatient	34.5 (7.7)	12 (20.0)	Cocaine	Methadone (<50%)
Petry, 2002 [55]	US	DSM-IV	CM (19) TAU (23)	12	24	Outpatient	38.5 (4.6)	30 (71.3)	Cocaine	Alcohol (<50%), Methadone (100%)
Petry, 2005 [56]	US	DSM IV	CM (209) TAU (206)	12	24	Outpatient	35.8 (8.7)	230 (55.4)	Amphetamine and cocaine	Alcohol (<50%)
Petry, 2005 [57]	US	DSM-IV	CM (40) TAU (37)	12	24	Outpatient	39.5 (1.1)	56 (72.7)	Cocaine	Methadone (100%)
Petry, 2007 [58]	US	DSM-IV	CM^f^ (27) CM^g^ (30) TAU (19)	12	36	Outpatient	41.6 (8.2)	43 (56.6)	Cocaine	Alcohol (<50%), Methadone (100%)
Petry, 2012 [59]	US	DSM-IV	CM (71) TAU (59)	12	36	Outpatient	36.6 (9.4)	61 (46.9)	Cocaine	Alcohol (<50%), Methadone (100%)
Petry, 2012 [60]	US	DSM-IV	CM^h^^,^^i^ (118) CM^h^^,^^j^ (35) CM^j^^,^^k^ (40) NCR^,^^i^ (107) TAU^,^^i^ (108) TAU^j^ (34)	6	36	Outpatient	36.5 (9.1)	176 (52.8)	Cocaine	Alcohol (<50%)
Petry, 2013 [61]	US	DSM-IV	CM (10) TAU (9)	8	NA	Outpatient	NA	NA	Cocaine	Methadone (≥50%)
Poling, 2006 [62]	US	DSM-IV	CM^l^ (27) CM^c^ (25) NCR^l^ (30) NCR^c^ (24)	25	NA	Outpatient	34.6 (NA)	32 (30.2)	Cocaine	Alcohol (<50%), Methadone (100%)
Rawson, 2002 [63]	US	DSM-IV	CBT (30) CBT + CM (30) CM (30) TAU (30)	16	52	Outpatient	43.6 (NA)	54 (45.0)	Cocaine	Methadone (100%)
Rawson, 2006 [64]	US	DSM-IV	CBT (58) CBT + CM (59) CM (60)	16	52	Outpatient	NA	42 (23.7)	Amphetamine and cocaine	NA
Roll, 2013 [65]	US	DSM-IV	CM^m^ (30) CM^n^ (30) CM^o^ (29) TAU (29)	16	48	Outpatient	32.2 (9.5)	53 (44.9)	Amphetamine	NA
Sánchez-Hervás, 2010 [66]	Spain	DSM-IV	CBT (34) CRA (40)	24	52	Outpatient	31.2 (6.3)	10 (13.5)	Cocaine	Alcohol (≥50%)
Schottenfeld, 2011 [67]	US	DSM-IV	12-step + CM (37) 12-step + NCR (37) CM + CRA (36) CRA + NCR (35)	24	48	Outpatient	31.1 (30.7)	145 (100)	Cocaine	Alcohol (<50%)
Secades-Villa, 2013 [68]	Spain	DSM-IV	CM + CRA (50) CRA (68)	24	NA	Outpatient	31.2 (6.6)	17 (14.4)	Cocaine	Alcohol (≥50%)
Shoptaw, 2005 [69]	US	DSM-IV	CBT (40) CBT + CM (40) CM (42) CBT^p^ (40)	16	52	Outpatient	37.2 (7.4)	0 (0)	Amphetamine	NA
Shoptaw, 2008 [70]	US	NR	CBT (64) TAU (64)	16	52	Outpatient	37.1 (7.7)	0 (0)	Amphetamine and cocaine	Alcohol (<50%)
Silverman, 1996 [71]	US	DSM-III-R	CM (19) NCR (18)	12	16	Outpatient	36.1 (1.5)	NA	Cocaine	Alcohol (<50%), Methadone (100%)
Silverman, 1998 [72]	US	DSM-III-R	CM^f^ (20) CM^q^ (20) NCR (19)	12	18	Outpatient	37.8 (5.1)	20 (33.9)	Cocaine	Alcohol (<50%), Methadone (100%)
Smout, 2010 [73]	Australia	DSM-IV	CBT (53) MBT (51)	12	24	Outpatient	NA	NA	Amphetamine	NA
Umbricht, 2014 [74]	US	DSM-IV	CM (39) CM^r^ (40) NCR (47) NCR^r^ (45)	12	NA	Outpatient	42.0 (7.0)	82 (47.9)	Cocaine	Alcohol (<50%), Methadone (100%)

^a^For full reference list, see S3 Text

^b^With desipramine

^c^With placebo

^d^With disulfiram

^e^With methylphenidate

^f^With voucher

^g^With cash/prize

^h^$250 reward

^i^Cocaine negative at intake

^j^$560 reward

^k^Cocaine negative at intake

^l^With bupropion hydrochloride

^m^One-month duration of treatment

^n^Two-month duration of treatment

^o^Four-month duration of treatment

^p^Gay-specific CBT

^q^With voucher and $50 bonus

^r^With topiramate.

CBT, cognitive behavioural therapy; CM, contingency management; CRA, community reinforcement approach; DSM-III-R, Diagnostic and Statistical Manual of Mental Disorders, 3rd edition, revised; DSM-IV, Diagnostic and Statistical Manual of Mental Disorders, 4th edition; MBT, meditation-based treatments; NA, not assessed; NCR, non-contingent rewards; NR, not reported; SEPT, supportive-expressive psychodynamic therapy; TAU, treatment as usual; 12-step, 12-step programme.

We present all the networks for specific outcomes in S2 Fig. Eight psychosocial interventions had at least 1 trial versus TAU, and all of them were directly compared with at least another psychosocial intervention. We obtained unpublished or supplementary information for 5 of the included studies [45,49,59–61].

The pairwise meta-analyses are presented in S4 Table, while data on heterogeneity are presented in S5 Table. The pairwise meta-analyses showed some statistically significant results in terms of abstinence and dropout. CM plus CBT, CM, and 12-step programme were superior to TAU in terms of abstinence at the end of treatment, while CBT, CM, and the combination of CM plus community reinforcement approach were superior to TAU in terms of dropout at the end of treatment (S4 Table).

**Fig 3:**
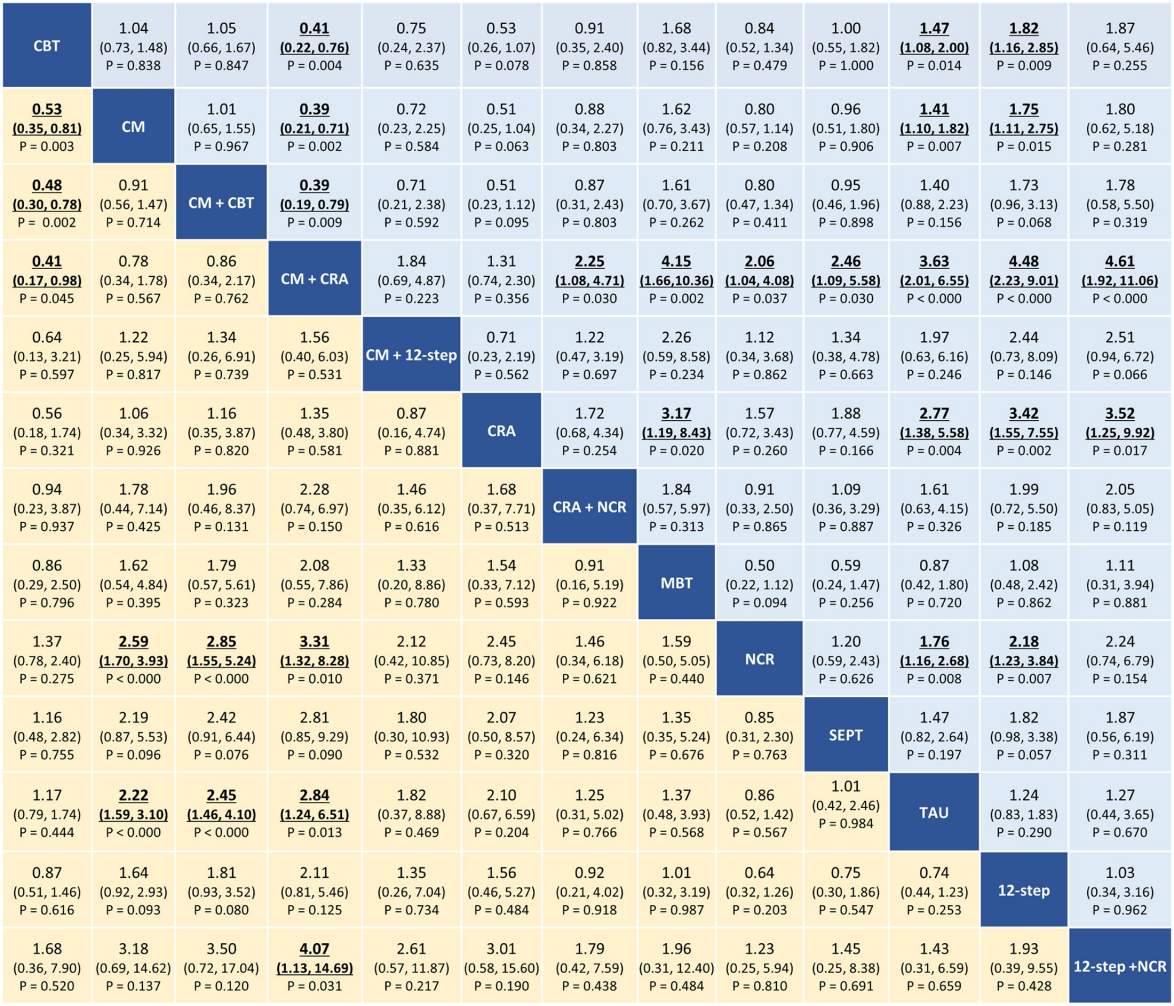
Network meta-analysis of efficacy (yellow) and acceptability (blue) at the end of treatment. Psychosocial treatments are reported in alphabetical order. Comparisons should be read from left to right. Abstinence and dropout estimates are located at the intersection between the column-defining and the row-defining treatment. For abstinence, ORs above 1 favour the column-defining treatment. For dropout, ORs above 1 favour the row-defining treatment. To obtain ORs for comparisons in the opposite direction, reciprocals should be taken. Significant results are in bold and underlined. CBT, cognitive behavioural therapy; CM, contingency management; CRA, community reinforcement approach; MBT, meditation-based therapies; NCR, non-contingent rewards; OR, odds ratio; SEPT, supportive-expressive psychodynamic therapy; TAU, treatment as usual; 12-step, 12-step programme.

The results of the network meta-analysis are presented in Fig 3. In terms of abstinence at the end of treatment, the combination of CM plus community reinforcement approach, the combination of CM plus CBT, and CM alone were superior to non-contingent rewards (OR ranging between 2.59 [95% CI 1.70–3.93], *P* < 0.001, and 3.31 [95% CI 1.32–8.28], *P* = 0.010) and to TAU (OR ranging between 2.22 [95% CI 1.59–3.10], *P* < 0.001, and 2.84 [95% CI 1.24–6.51], *P* = 0.013). Moreover, the combination of CM plus community reinforcement approach was also superior to the combination 12-step programme plus non-contingent rewards and to CBT (OR 4.07 [95% CI 1.13–14.69], *P* = 0.031, and 2.43 [95% CI 1.02–5.88], *P* = 0.045, respectively), while CM alone and the combination of CM plus CBT were superior to CBT (OR 1.88 [95% CI 1.52–2.85], *P* = 0.003, and 2.08 [95% CI 1.28–3.33], *P* = 0.002, respectively). In terms of dropouts at the end of treatment, the combination of CM plus community reinforcement approach, community reinforcement approach alone, non-contingent rewards, CM alone, and CBT were better accepted than TAU (OR ranging between 1.41 [95% CI 1.10–1.82], *P* = 0.007, and 3.63 [95% CI 2.01–6.55], *P* < 0.001). Moreover, the combination of CM plus community reinforcement approach was better accepted than CBT, CM alone, CM plus CBT, community reinforcement approach plus non-contingent rewards, meditation-based therapies, non-contingent rewards, supportive-expressive psychodynamic therapy, 12-step programme alone, and 12-step programme plus non-contingent rewards (OR ranging between 2.06 [95% CI 1.04–4.08], *P* = 0.037, and 4.61 [95% CI 1.92–11.06], *P* < 0.001).

**Fig 4:**
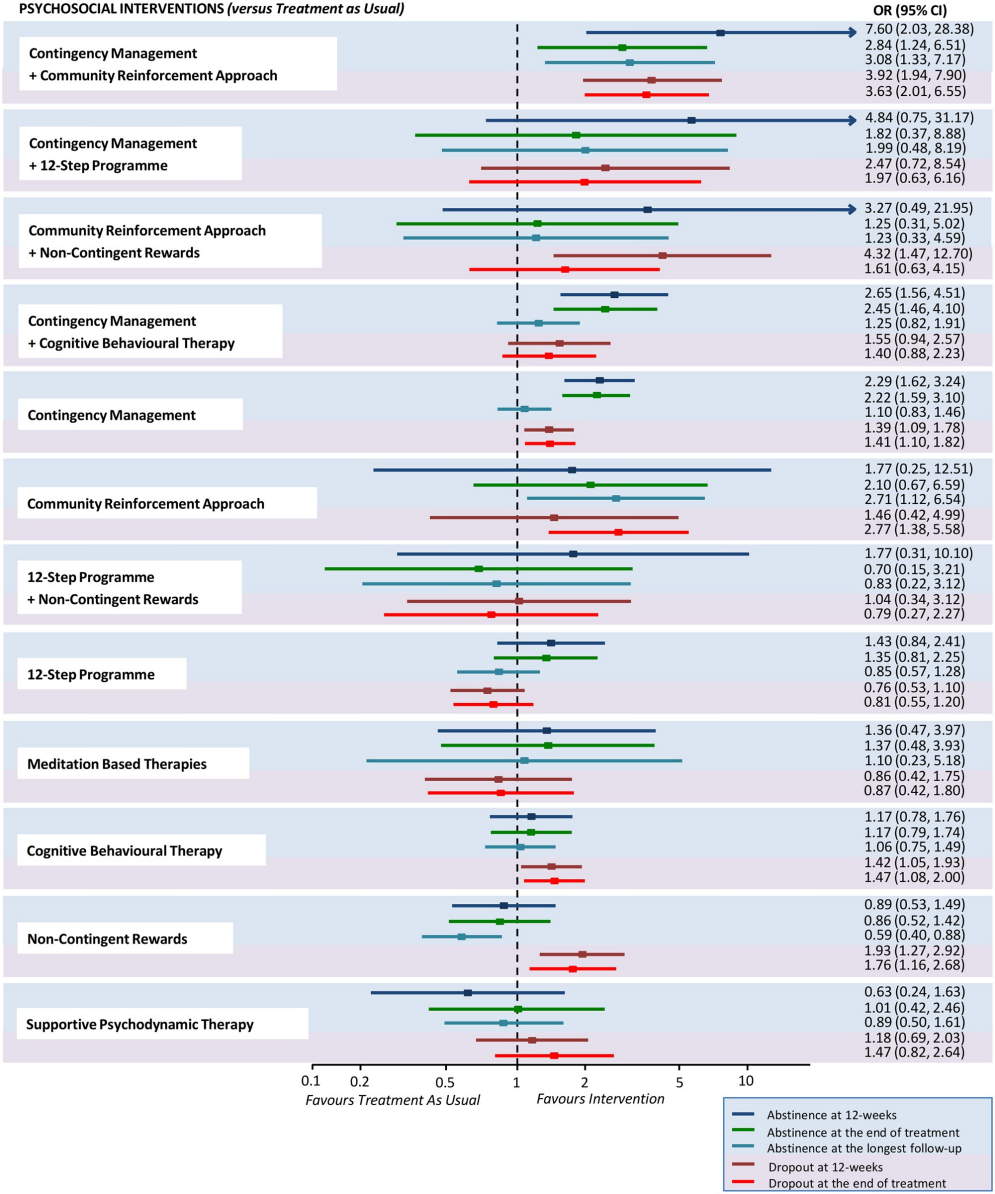
Abstinence and dropout at different time-points for each psychosocial intervention versus treatment as usual. Estimates are reported by ORs, where an OR above 1 favours the psychosocial intervention indicated on the left side over treatment as usual. For each intervention, efficacy outcomes are reported in the blue-shaded area, while acceptability outcomes are reported in the pink-shaded area. OR, odds ratio.

Fewer studies reported results for abstinence measured at 12 weeks of treatment (S3 Fig) and at the longest follow-up after treatment completion (S4 Fig), but findings were in line with the outcome data at the end of treatment. Comparative abstinence and dropout at different time-points for each psychosocial intervention versus TAU are presented in Fig 4.

In terms of the longest duration of abstinence measured at 12 weeks (S5 Fig), we found that the combination of CM plus CBT and CM alone were superior to TAU (SMD 0.75 [95% CI 0.31–1.19] and 0.62 [95% CI 0.43–0.80], respectively). Likewise, for the longest duration of abstinence measured at the end of treatment (S6 Fig), we found that the combination of CM plus CBT and CM alone were superior to TAU (SMD 0.74 [95% CI 0.43–1.06] and 0.60 [95% CI 0.43–0.76], respectively).

The common heterogeneity SD for the coherence model was 0.46 and 0.21 for abstinence and dropout at the end of treatment, respectively (it was 0.47 and 0.19 for abstinence and dropout at 12 weeks, respectively). The global incoherence was not significant for all the outcomes considered (S6 Table). Tests of local incoherence did not show any inconsistent loops for abstinence and dropout at the end of treatment, although in some cases the ratio of the odds ratios (RoR) from direct and indirect evidence was large (i.e., RoR > 2), and we cannot definitely exclude the presence of incoherence [22]. We found only 1 inconsistent loop for abstinence measured at 12 weeks and no other inconsistent loops for the other outcomes considered at 12 weeks (S7 Table; S7 Fig). The test of incoherence from the side-splitting model did not show significant differences for abstinence at the end of treatment but found some differences between some comparisons for dropout at the end of treatment (S8 Table). The comparison-adjusted funnel plots of the network meta-analysis for abstinence and dropout at the end of treatment were not suggestive for significant publication bias (S8 Fig).

The ranking of psychosocial interventions based on cumulative probability plots and SUCRAs is presented in S9 Table and S9 Fig. We also performed subgroup analyses for abstinence and dropout at the end of treatment to study the effect of several potential moderator variables, the findings of which did not substantially differ from those of the primary analysis for most of the comparisons (S10 Table). Pre-planned sensitivity analysis on individuals addicted to cocaine only did not affect the main results (S10 Fig), while pre-planned sensitivity analysis on individuals on opioid substitution therapy showed a superiority of CM alone and CM plus CBT over TAU and non-contingent rewards, and a superiority of CM alone over CBT (S11 Fig). Predictivity intervals of mixed estimates are presented in S11 Table, while the overall limitations per comparison are presented in S12 and S13 Figs.

We found that 4 patients needed to be treated with CM plus community reinforcement approach to have 1 additional patient abstinent at the end of treatment compared toTAU (NNT 4.07, 95% CI 2.29–21.95), with consistent results at longest follow-up after treatment completion (NNT 3.68, 95% CI 2.36–14.24). Similarly, 3 patients needed to be treated with CM plus community reinforcement approach to have 1 fewer patient dropping out at the end of treatment compared to TAU (NNT 3.25, 95% CI 2.42–5.79). For abstinence at the end of treatment, the NNT for the combination of CM plus CBT and for CM alone versus TAU was 4.80 (95% CI 2.99–12.12) and 5.44 (95% CI 3.74–9.75), respectively. For dropout at the end of treatment, the NNT ranged from 4.02 (95% CI 2.58–12.62) for CRA to 7.15 (95% CI 4.15–27.66) for non-contingent rewards, 10.52 (95% CI 5.83–53.65) for CBT, and 11.82 (95% CI 6.74–43.26) for CM.

The overall strength of evidence according to GRADE is summarised in S12 Table for abstinence and for dropout at the end of treatment. For CM plus community reinforcement approach versus TAU, the strength of evidence was rated as “moderate” for abstinence and as “high” for dropout due to any cause. For CM plus CBT and CM alone versus TAU, the strength of evidence for abstinence was rated as “moderate” for both, while the strength of evidence for dropout due to any cause was rated as “very low” and “moderate”, respectively.

## Discussion

This network meta-analysis is based on 50 studies including 6,942 individuals randomly assigned to 12 different psychosocial interventions or TAU. To our knowledge, it is the most comprehensive synthesis of data for all available psychosocial interventions in individuals with cocaine and/or amphetamine addiction.

We found that CM alone or in combination with either community reinforcement approach or CBT had superior efficacy and acceptability compared to TAU at 12 weeks and at the end of treatment. This effect was not significantly influenced by clinical modifiers in the subgroup analyses and remained significant in the sensitivity analyses. Moreover, CM in combination with community reinforcement approach and community reinforcement approach alone were more effective than TAU at the longest follow-up after treatment completion. The clinical relevance of this finding is key, because achieving long-term abstinence is the main treatment goal for individuals with cocaine and/or amphetamine addiction [18].

Several major guidelines recommend the use of either CBT or CM alone for the treatment of cocaine and/or amphetamine addiction [6,8,10]. Self-help groups following the 12-step programme are also recommended [8]. Our results do not support these recommendations. We found that CBT alone was more acceptable than TAU (NNT 10.5, 95% CI 5.8–53.6), but it was not superior for abstinence on any dichotomous or continuous outcome measured and was less effective than CM alone. CM alone showed greater efficacy (NNT 5.2, 95% CI 3.6–9.3) and acceptability (NNT 12.5, 95% CI 7.0–48.8) than TAU at 12 weeks of treatment and at the end of treatment (NNT 5.4, 95% CI 3.8–9.8, and 11.9, 95% CI 6.8–43.3, respectively), but the effect was not sustained at the longest follow-up after treatment completion. Both CBT and CM were inferior to CM in combination with community reinforcement approach at the longest follow-up after treatment completion. Community reinforcement approach alone was not different from TAU for abstinence at 12 weeks of treatment or at the end of treatment, but showed increased abstinence at the longest follow-up after treatment completion (NNT 4.1, 95% CI 2.4–36.2). CM in combination with community reinforcement approach was superior to TAU for abstinence at 12 weeks of treatment (NNT 2.1, 95% CI 1.6–6.2), at the end of treatment (NNT 4.1, 95% CI 2.3–21.9), and at the longest follow-up after treatment completion (NNT 3.7, 95% CI 2.4–14.2), as well as for acceptability at 12 weeks of treatment (NNT 3.1, 95% CI 2.2–6.1) and at the end of treatment (NNT 3.3, 95% CI 2.3–6.3), as shown in Fig 4. CM plus community reinforcement approach was also superior to 12-step programme for abstinence and dropout at 12 weeks of treatment, for dropout at the end of treatment, and for abstinence at the longest follow-up after study completion.

Behavioural interventions have proved effective for the treatment of other forms of addiction [75–77]. There is growing evidence that reducing punishment—such as incarceration—and adopting positive reinforcement for people with substance use improves their access to services, their reintegration into society, and, ultimately, public safety [78–80]. Recent experimental data emphasise the potential of interventions that focus on improving goal-directed behaviour and positive reinforcement rather than punishment in people with cocaine addiction [81]. The efficacy of a purely behavioural intervention—such as CM alone—shows that financial rewards can compete with biological rewards mediated by cocaine and amphetamine cues and incentives [82]. This seems to be true only if rewards are contingent upon the provision of drug-free urine samples, as non-contingent rewards were not shown to be effective (Fig 4). Indeed, CM strategies help individuals to overcome apathy or resistance to the recovery process. In this study we found that, although CM was efficacious at the end of treatment, the effect of CM alone was not sustained at longest follow-up after treatment completion (Fig 4). Cocaine and amphetamine addiction is conceptualised as a chronic and recurrent brain disease, which entails behavioural and psychological abnormalities (primarily reward-processing deficits) following the learned or conditioned pairing of situational and social cues with the reinforcing effects of drug use [83,84]. It seems unlikely that a behavioural strategy alone could address in the long term the whole complexity of biological, psychological, and behavioural factors that underlie addiction. The addition of community reinforcement approach to CM potentiates an otherwise purely behavioural intervention with psychological and social components that may enhance its effect. Notably, community reinforcement approach alone performs no differently from TAU in the short term, but its effect is more sustained at longest follow-up. The combined intervention, CM plus community reinforcement approach, overall achieves the best outcomes.

Cocaine and amphetamine addiction is highly prevalent in the world and is incredibly costly economically. In 2015, illicit drugs cost tens of millions of disability-adjusted life years, with Europeans proportionately experiencing more, but with the greatest mortality rate in low- and middle-income countries [85]. We did not do a formal cost-effectiveness analysis. Indeed, recent cost-effectiveness analyses on psychosocial interventions for substance use are encouraging [8,86], but without a full economic model our recommendation cannot be made unequivocally.

This study has some limitations. Some comparisons were appraised as having low or very low quality, potentially restricting the validity of those results. All RCTs of psychosocial interventions for cocaine and/or amphetamine addiction are not blinded, which increases the risk of performance bias for self-reported outcomes. For this reason, we only reported data based on objective outcomes (abstinence on urinalysis and data on attrition), which are less likely to be influenced by the lack of blinding. The risk of selective study reporting was minimised as we contacted study authors to retrieve unpublished data, but we cannot exclude that some unpublished studies remain missing or that published reports overestimated the efficacy of treatments. Finally, some interventions are designed to last more than 12 weeks, namely, CM in combination with community reinforcement approach (24 weeks) [40], community reinforcement approach alone (time unlimited, but in the studies included the intervention lasted always 24 weeks), and supportive-expressive psychodynamic therapy (which could be either time-limited or unlimited and lasted 36 weeks in the only study included) [87]. For these interventions, the evaluation at 12 weeks was extracted before the end of treatment, which was a disadvantage over other interventions requiring shorter duration.

### Conclusions

The results of this network meta-analysis support the use of combined CM plus community reinforcement approach as the most effective and acceptable intervention for both short- and long-term treatment of individuals with cocaine and/or amphetamine addiction. The provision of evidence-based psychosocial treatments for stimulant use disorders is all the more important because of the lack of validated pharmacological or brain-stimulation-based treatment for cocaine and/or amphetamine addiction. These findings may influence clinical guidelines; however, further studies are warranted to confirm these results and evaluate cost-effectiveness.

## Supporting information

S1 Data Open data extraction. (XLSX)

S1 Fig Risk of bias graph. (DOCX)

S2 Fig Network of eligible comparisons for each outcome. (DOCX)

S3 Fig Network meta-analysis of abstinence and dropout at 12 weeks of treatment. (DOCX)

S4 Fig Network meta-analysis for abstinence at the longest follow-up after study completion. (DOCX)

S5 Fig Network meta-analysis for the longest duration of abstinence at 12 weeks. (DOCX)

S6 Fig Network meta-analysis for the longest duration of abstinence at the end of treatment. (DOCX)

S7 Fig Evaluation of the local incoherence for each outcome. (DOCX)

S8 Fig Comparison-adjusted funnel plot for each outcome. (DOCX)

S9 Fig Cumulative probability plots (random-effects model) for each outcome. (DOCX)

S10 Fig Sensitivity network meta-analysis for abstinence at the end of treatment and dropout at the end of treatment considering only the trials on individuals addicted to cocaine and no other stimulant. (DOCX)

S11 Fig Sensitivity network meta-analysis for abstinence and dropout at the end of treatment considering only the trials on individuals addicted to stimulants and on opioid substitution therapy. (DOCX)

S12 Fig Study limitations for each pairwise estimate as the risk of bias judgments from all direct estimates for abstinence and dropout at the end of treatment. (DOCX)

S13 Fig Network plot by risk of bias for abstinence and dropout at the end of treatment. (DOCX)

S1 PRISMA Checklist Preferred Reporting Items for Systematic Reviews and Meta-Analyses (PRISMA) checklist of items to include when reporting a systematic review involving a network meta-analysis. (DOCX)

S1 Table Dropout rates based on each intervention. (DOCX)

S2 Table Addiction severity. (DOCX)

S3 Table Risk of bias summary. (DOCX)

S4 Table Summary numbers of studies and patients from pairwise meta-analysis of direct comparisons and summary estimates from pairwise meta-analysis of direct comparisons. (DOCX)

S5 Table Heterogeneity test result, *I*^2^, and heterogeneity estimate for each outcome. (DOCX)

S6 Table Evaluation of the global incoherence. (DOCX)

S7 Table Evaluation of the local incoherence for each outcome. (DOCX)

S8 Table Evaluation of the incoherence by side-splitting model for each outcome. (DOCX)

S9 Table Treatment ranking for each outcome. (DOCX)

S10 Table Subgroup network meta-analyses of each psychosocial treatment for abstinence and for dropout due to any cause at the end of treatment compared with treatment as usual, with odds ratio (95% CI). (DOCX)

S11 Table Predictivity intervals of mixed estimates for abstinence and dropout at the end of treatment. (DOCX)

S12 Table Evaluation of the quality of evidence using the GRADE framework for abstinence and dropout at the end of treatment. (DOCX)

S1 Text Search strategy and results. (DOCX)

S2 Text Summary of statistical analysis and GRADE. (DOCX)

S3 Text Full reference list of included trials. (DOCX)

S4 Text Description of psychosocial interventions used in the trials. (DOCX)

## Abbreviations

12-step: 12-step programme
CBT: cognitive behavioural therapy
CM: contingency management
CRA: community reinforcement approach
GRADE: Grading of Recommendations Assessment, Development and Evaluation
MBT: meditation-based treatments
NCR: non-contingent rewards
NNT: number needed to treat
OR: odds ratio
RCT: randomised controlled trial
SEPT: supportive-expressive psychodynamic therapy
SMD: standardised mean difference
SUCRA: surface under the cumulative ranking curve
TAU: treatment as usual
