# Supplementary material for: Comparative efficacy and acceptability of psychosocial interventions for individuals with cocaine and amphetamine addiction: A systematic review and network meta-analysis
Source: PLoS Med. 2018 Dec 26;15(12):e1002715. doi: 10.1371/journal.pmed.1002715 (PMC6306153; doi:10.1371/journal.pmed.1002715)
Supplement: S1 Fig — (DOCX) [file pmed.1002715.s002.docx]

**S1 Fig. Risk of Bias Graph.**

**These are review authors' judgments about each risk of bias item presented as percentages across all included studies.**
