# Supplementary material for: Comparative efficacy and acceptability of psychosocial interventions for individuals with cocaine and amphetamine addiction: A systematic review and network meta-analysis
Source: PLoS Med. 2018 Dec 26;15(12):e1002715. doi: 10.1371/journal.pmed.1002715 (PMC6306153; doi:10.1371/journal.pmed.1002715)
Supplement: S2 Fig — (DOCX) [file pmed.1002715.s003.docx]

**S2a Fig. Network of Eligible Comparisons for Abstinence at 12 Weeks (42 Studies).**

**
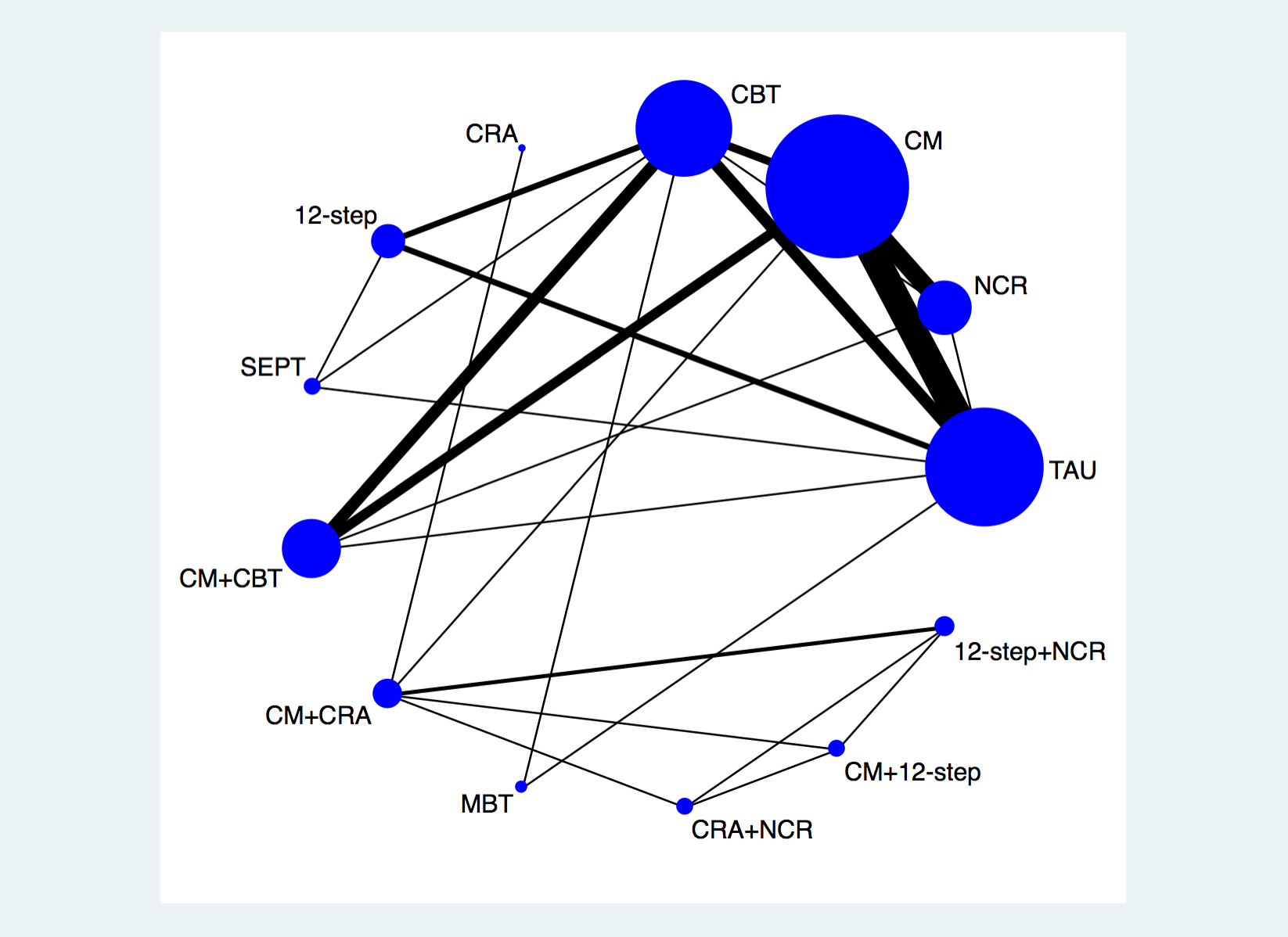
**

**S2b Fig. Network of Eligible Comparisons for Abstinence at the End of Treatment (Efficacy) (46 Studies).**

**
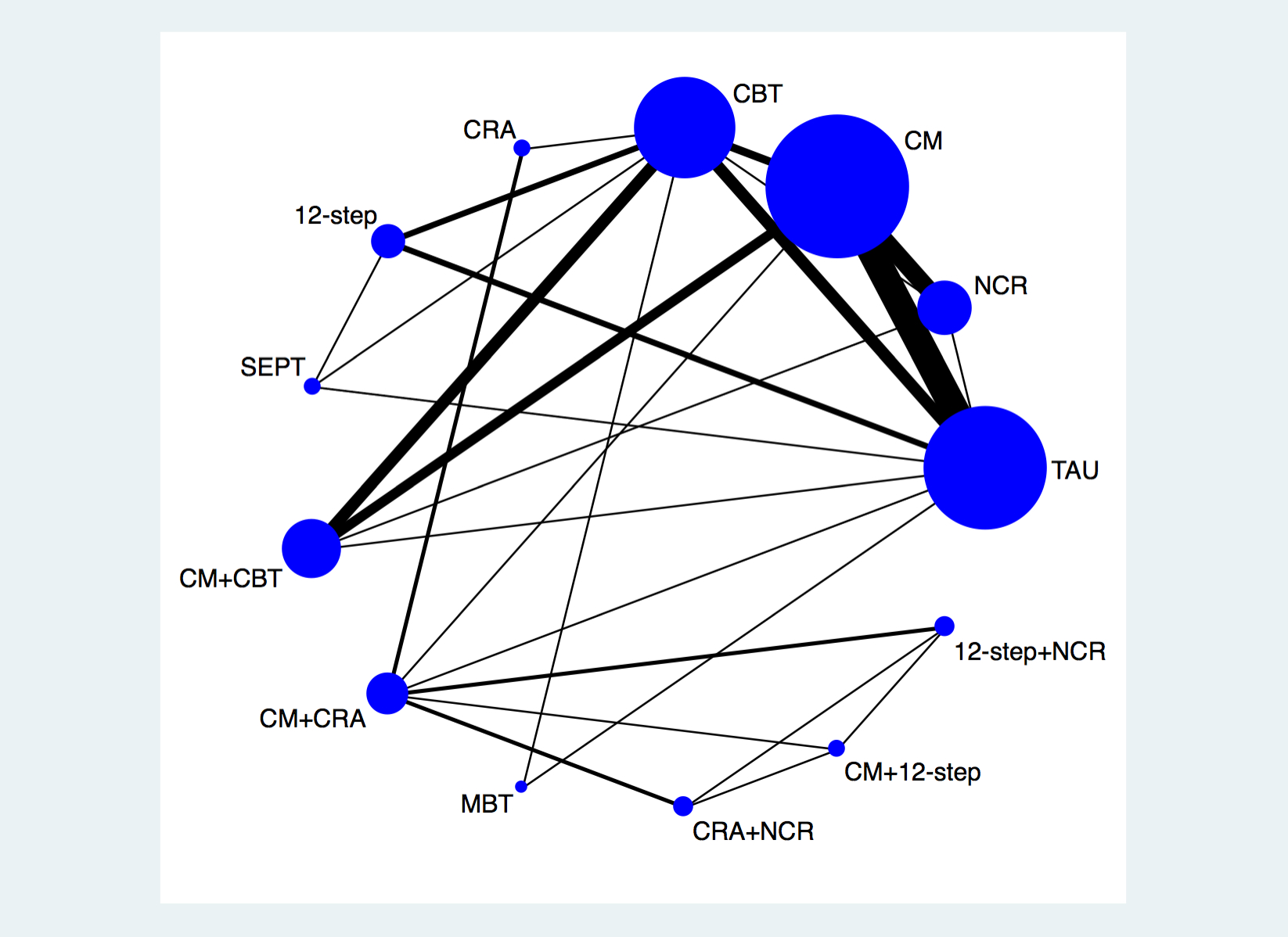
**

**S2c Fig. Network of Eligible Comparisons for Abstinence at the Longest Follow-Up after Study Completion (32 Studies).**

**
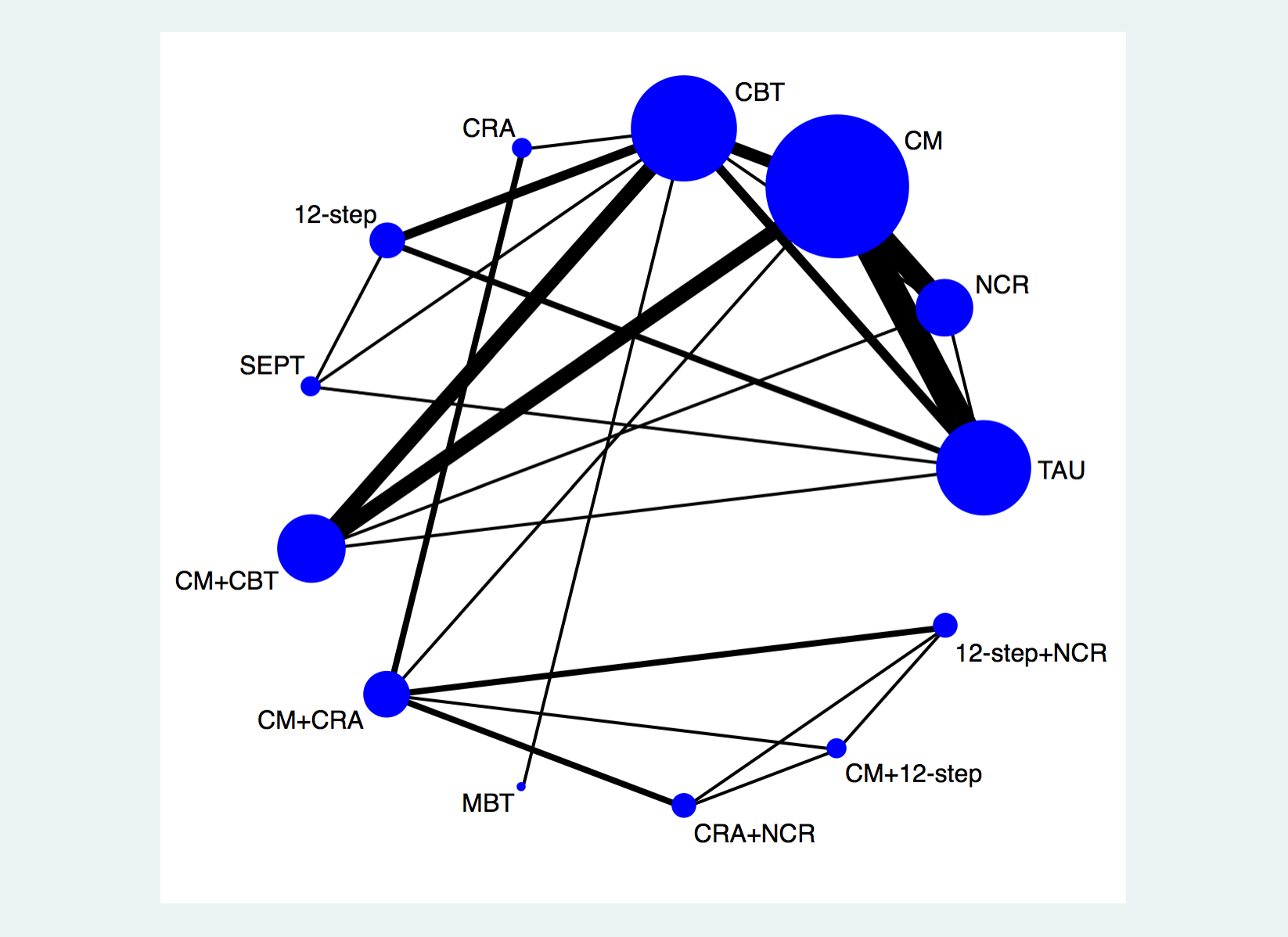
**

**S2d Fig. Network of Eligible Comparisons for Dropout due to any Cause at 12 Weeks (41 Studies).**

**
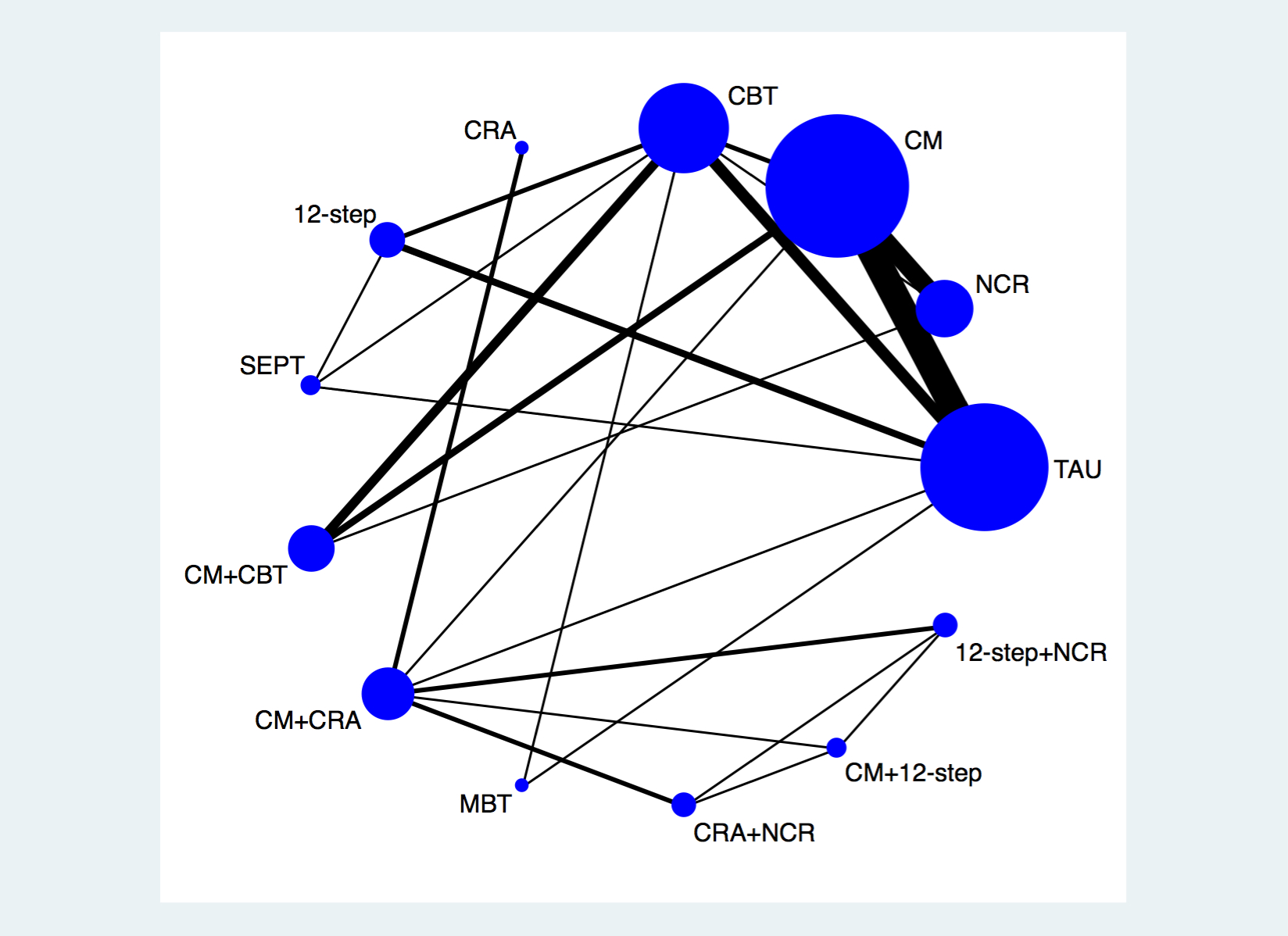
**

**S2e Fig. Network of Eligible Comparisons for Dropout due to any Cause at the End of Treatment (43 Studies).**

**
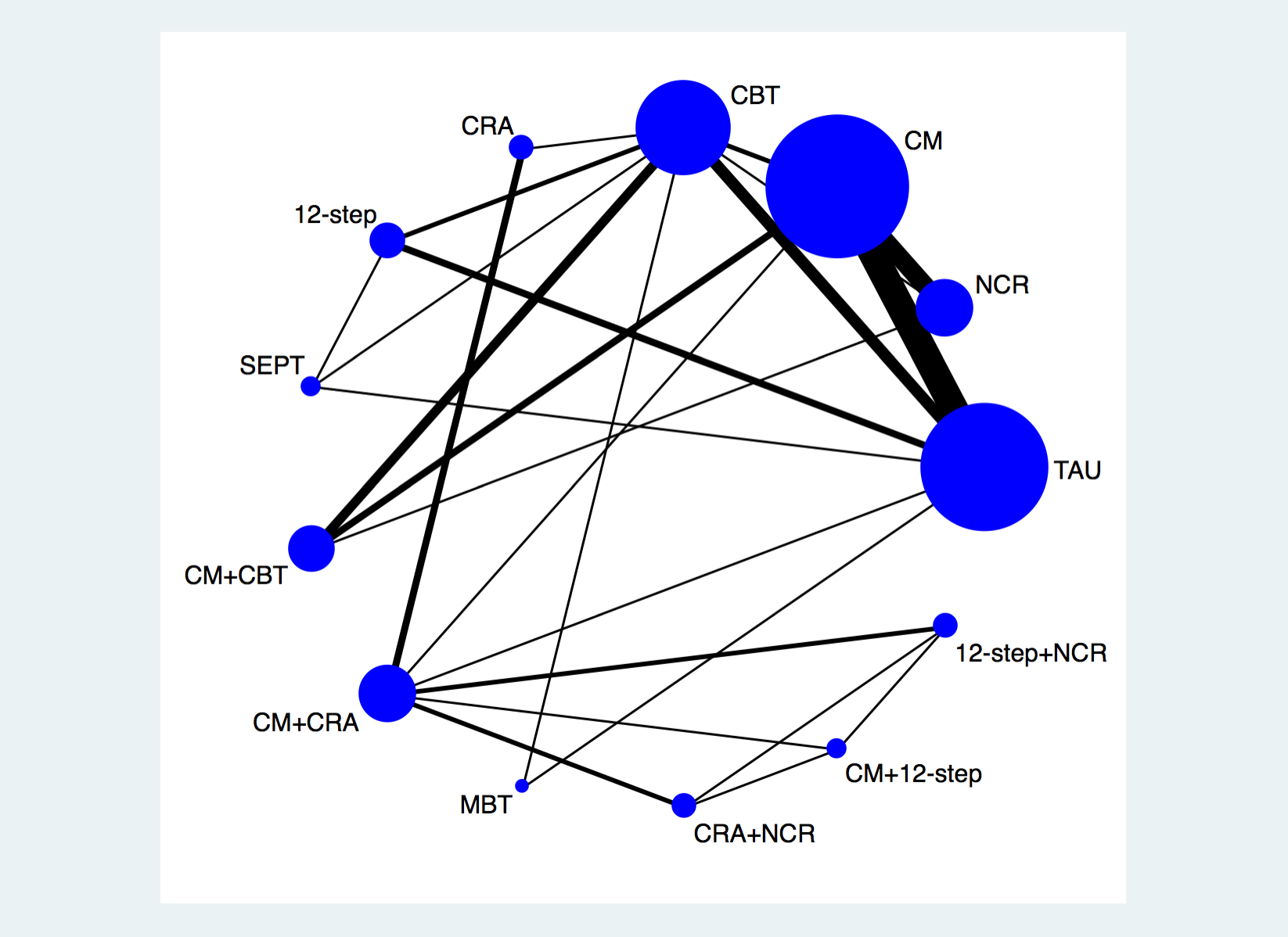
**

**S2f Fig. Network of Eligible Comparisons for the Longest Duration of Abstinence at 12 Weeks (21 Studies).**

**
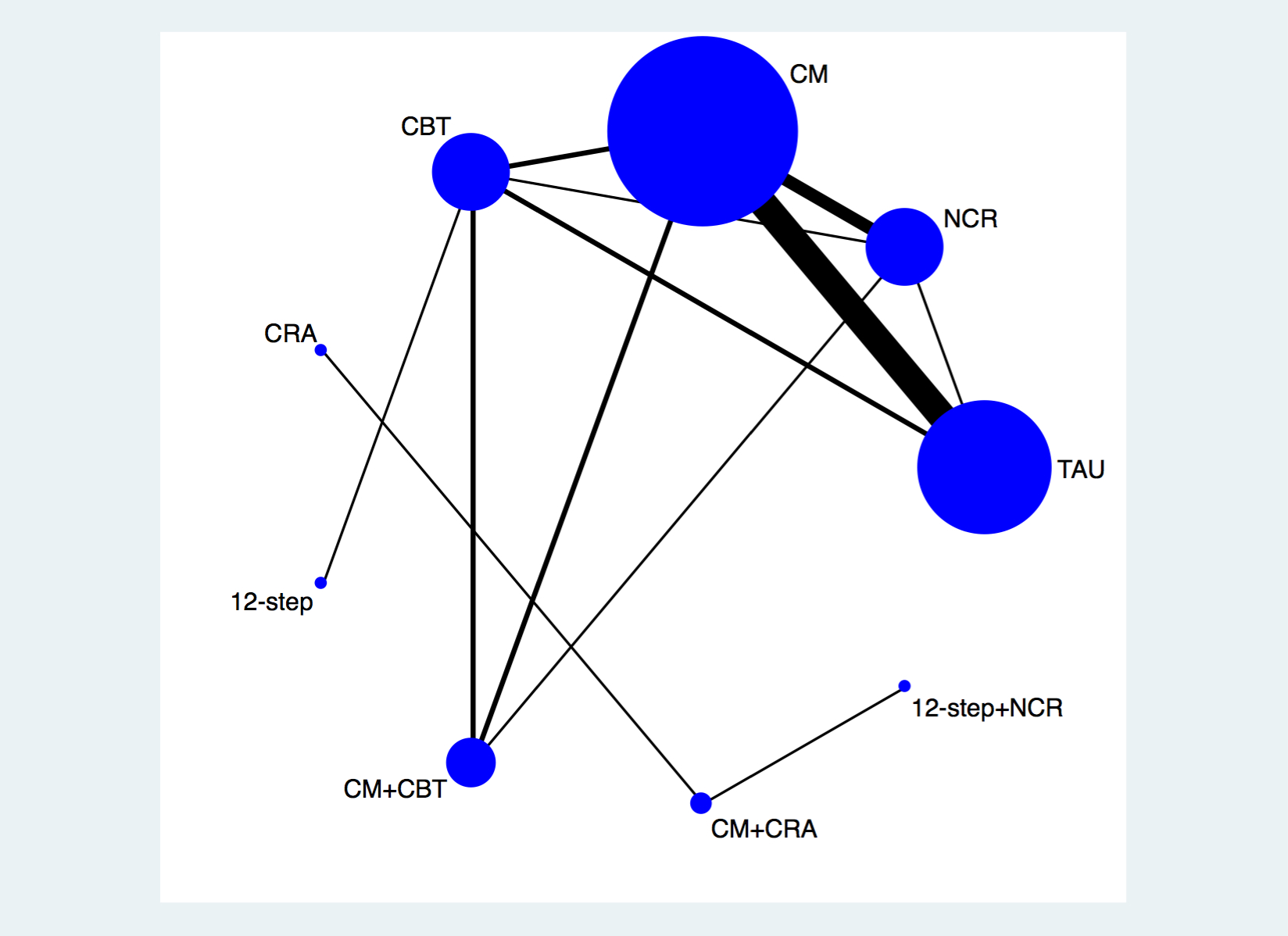
**

**S2g Fig. Network of Eligible Comparisons for the Longest Duration of Abstinence at the End of Treatment (25 Studies).**

**
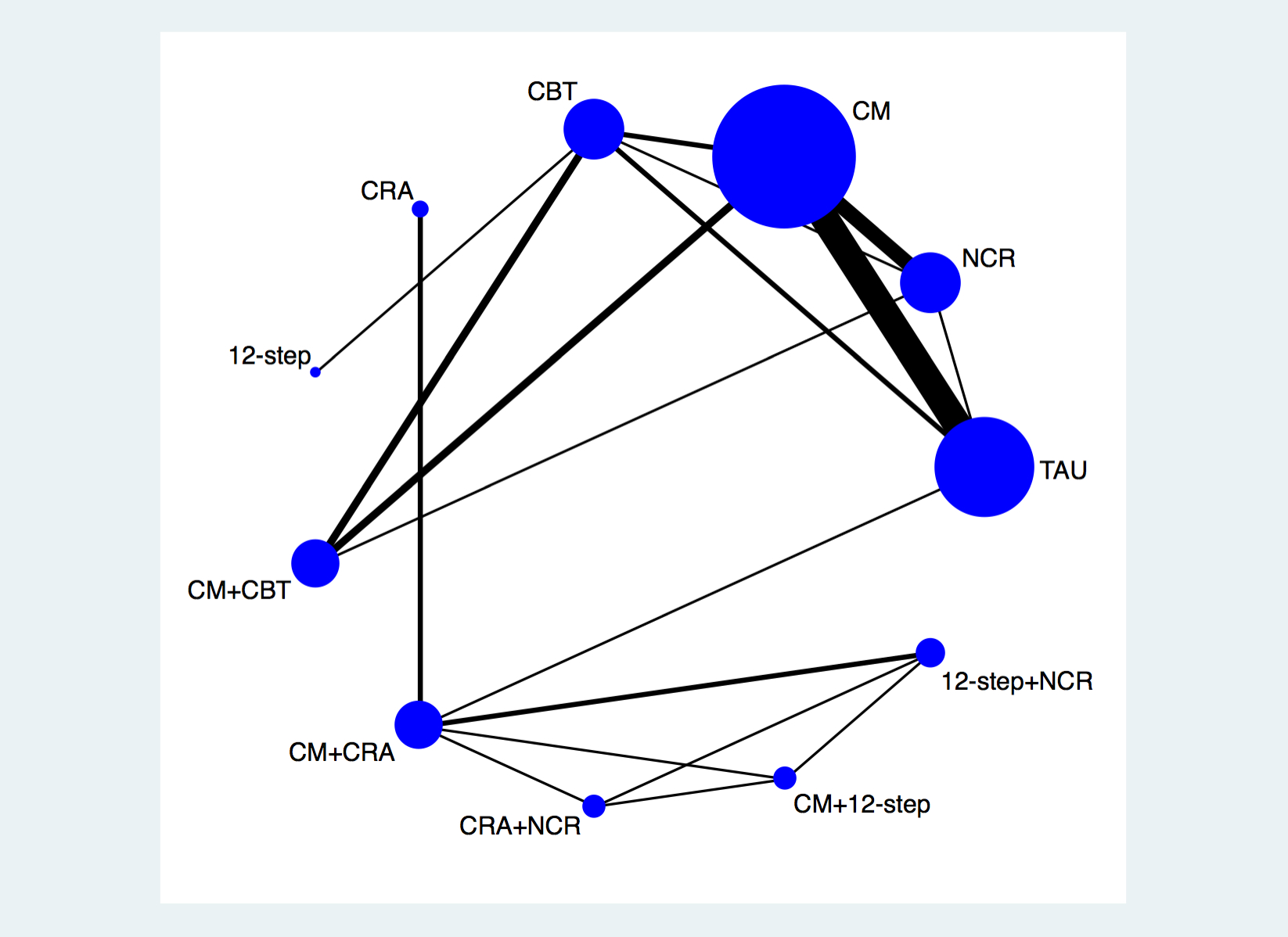
**
