# Supplementary material for: Comparative efficacy and acceptability of psychosocial interventions for individuals with cocaine and amphetamine addiction: A systematic review and network meta-analysis
Source: PLoS Med. 2018 Dec 26;15(12):e1002715. doi: 10.1371/journal.pmed.1002715 (PMC6306153; doi:10.1371/journal.pmed.1002715)
Supplement: S3 Fig — (DOCX) [file pmed.1002715.s004.docx]

**S3 Fig. Network Meta-Analysis of Abstinence and Dropout at 12 Weeks of Treatment.**

| **CBT** | 1.02  (0.72, 1.45) | 0.92  (0.56, 1.51) | **0.36**  **(0.17, 0.77)** | 0.58  (0.16, 2.05) | 0.98  (0.28, 3.46) | 0.33  (0.11, 1.00) | 1.66  (0.82, 3.34) | 0.74  (0.46, 1.18) | 1.21  (0.69, 2.10) | **1.42**  **(1.05, 1.93)** | **1.87**  **(1.22, 2.86)** | 1.37  (0.43, 4.30) |
| --- | --- | --- | --- | --- | --- | --- | --- | --- | --- | --- | --- | --- |
| **0.51**  **(0.33, 0.79)** | **CM** | 0.90  (0.55, 1.46) | **0.36**  **(0.18, 0.72)** | 0.56  (0.16, 1.95) | 0.96  (0.28, 3.29) | **0.32**  **(0.11, 0.95)** | 1.62  (0.77, 3.39) | 0.72  (0.51, 1.02) | 1.18  (0.66, 2.11) | **1.39**  **(1.09, 1.78)** | **1.83**  **(1.19, 2.82)** | 1.34  (0.44, 4.07) |
| **0.44**  **(0.27, 0.72)** | 0.86  (0.53, 1.42) | **CM + CBT** | **0.40**  **(0.17, 0.92)** | 0.63  (0.17, 2.36) | 1.07  (0.29, 3.98) | 0.36  (0.11, 1.16) | 1.80  (0.78, 4.17) | 0.80  (0.46, 1.41) | 1.31  (0.64, 2.67) | 1.55  (0.94, 2.57) | **2.04**  **(1.12, 3.73)** | 1.49  (0.45, 4.97) |
| **0.15**  **(0.04, 0.59)** | 0.30  (0.08, 1.07) | 0.35  (0.09, 1.37) | **CM + CRA** | 1.58  (0.57, 4.40) | 2.69  (0.98, 7.43) | 0.91  (0.40, 2.06) | **4.56**  **(1.69, 12.32)** | 2.03  (0.93, 4.45) | **3.32**  **(1.37, 8.01)** | **3.92**  **(1.94, 7.90)** | **5.16**  **(2.35, 11.33)** | **3.76**  **(1.59, 8.93)** |
| 0.24  (0.04, 1.59) | 0.47  (0.08, 2.95) | 0.55  (0.08, 3.65) | 1.57  (0.42, 5.86) | **CM+12step** | 1.70  (0.40, 7.17) | 0.57  (0.20, 1.66) | 2.88  (0.69, 11.96) | 1.28  (0.35, 4.64) | 2.09  (0.54, 8.07) | 2.47  (0.72, 8.54) | 3.25  (0.90, 11.82) | 2.38  (0.86, 6.56) |
| 0.66  (0.09, 4.77) | 1.29  (0.19, 8.87) | 1.50  (0.21, 10.94) | **4.30**  **(1.01, 18.24)** | 2.74  (0.39, 19.34) | **CRA** | 0.34  (0.09, 1.24) | 1.69  (0.41, 7.00) | 0.76  (0.21, 2.72) | 1.23  (0.32, 4.72) | 1.46  (0.42, 4.99) | 1.91  (0.53, 6.91) | 1.40  (0.37, 5.29) |
| 0.36  (0.05, 2.45) | 0.70  (0.11, 4.56) | 0.81  (0.12, 5.63) | 2.33  (0.59, 9.21) | 1.48  (0.35, 6.26) | 0.54  (0.07, 3.99) | **CRA + NCR** | **5.03**  **(1.39, 18.22)** | 2.24  (0.72, 6.96) | **3.66**  **(1.10, 12.18)** | **4.32**  **(1.47, 12.70)** | **5.68**  **(1.82, 17.71)** | **4.15**  **(1.58, 10.88)** |
| 0.86  (0.29, 2.53) | 1.68  (0.55, 5.07) | 1.94  (0.61, 6.18) | **5.57**  **(1.03, 30.05)** | 3.55  (0.42, 30.10) | 1.30  (0.14, 11.95) | 2.39  (0.27, 21.08) | **MBT** | 0.45  (0.20, 1.00) | 0.73  (0.31, 1.73) | 0.86  (0.42, 1.75) | 1.13  (0.52, 2.47) | 0.83  (0.22, 3.07) |
| 1.31  (0.74, 2.34) | **2.56**  **(1.68, 3.91)** | **2.97**  **(1.59, 5.53)** | **8.51**  **(2.23, 32.50)** | 5.42  (0.83, 35.45) | 1.98  (0.28, 14.22) | 3.66  (0.54, 24.95) | 1.53  (0.47, 4.91) | **NCR** | 1.63  (0.84, 3.19) | **1.93**  **(1.27, 2.92)** | **2.54**  **(1.47, 4.38)** | 1.85  (0.58, 5.92) |
| 1.86  (0.72, 4.85) | **3.64**  **(1.35, 9.82)** | **4.22**  **(1.48, 12.00)** | **12.09**  **(2.41, 60.64)** | 7.70  (0.96, 61.74) | 2.81  (0.32, 24.55) | 5.20  (0.62, 43.29) | 2.17  (0.53, 8.88) | 1.42  (0.49, 4.11) | **SEPT** | 1.18  (0.69, 2.03) | 1.55  (0.88, 2.74) | 1.13  (0.33, 3.90) |
| 1.17  (0.78, 1.76) | **2.29**  **(1.62, 3.24)** | **2.65**  **(1.56, 4.51)** | **7.60**  **(2.03, 28.38)** | 4.84  (0.75, 31.17) | 1.77  (0.25, 12.51) | 3.27  (0.49, 21.95) | 1.36  (0.47, 3.97) | 0.89  (0.53, 1.49) | 0.63  (0.24, 1.63) | **TAU** | 1.32  (0.91, 1.89) | 0.96  (0.32, 2.92) |
| 0.82  (0.48, 1.40) | 1.60  (0.89, 2.90) | 1.86  (0.94, 3.67) | **5.33**  **(1.31, 21.68)** | 3.39  (0.50, 23.24) | 1.24  (0.17, 9.30) | 2.29  (0.32, 16.34) | 0.96  (0.30, 3.06) | 0.63  (0.31, 1.26) | 0.44  (0.17, 1.16) | 0.70  (0.42, 1.18) | **12 step** | 0.73  (0.23 2.35) |
| 0.66  (0.11, 3.85) | 1.29  (0.23, 7.13) | 1.50  (0.25, 8.86) | **4.29**  **(1.37, 13.43)** | 2.73  (0.69, 10.82) | 1.00  (0.16, 6.30) | 1.85  (0.44, 7.73) | 0.77  (0.10, 5.89) | 0.50  (0.09, 2.93) | 0.36  (0.05, 2.56) | 0.57  (0.10, 3.23) | 0.81  (0.13, 4.91) | **12step+NCR** |

Psychosocial treatment Abstinence at 12 weeks (OR [95% Cl]) Dropout due to any cause at 12 weeks (OR [95% Cl]

**Notes**. Psychosocial treatments are reported in alphabetical order. Comparisons should be read from left to right. The abstinence and dropout estimates are located at the intersection of the column-defining treatment and the row-defining treatment. For abstinence at 12 weeks, an OR above 1 favors the column-defining treatment. For dropout due to any cause qt 12 weeks, an OR above 1 favors the row-defining treatment. To obtain ORs for comparisons in the opposing direction, reciprocals should be taken. Significant results are in bold and underlined. CBT: cognitive behavioural therapy; CM: contingency management; CRA: community reinforcement approach; MBT: meditation based therapies; NCR: not contingent rewards; SEPT: supportive expressive psychodynamic therapy; TAU: treatment as usual; 12 step: twelve-step programme.
