# Supplementary material for: Comparative efficacy and acceptability of psychosocial interventions for individuals with cocaine and amphetamine addiction: A systematic review and network meta-analysis
Source: PLoS Med. 2018 Dec 26;15(12):e1002715. doi: 10.1371/journal.pmed.1002715 (PMC6306153; doi:10.1371/journal.pmed.1002715)
Supplement: S4 Fig — (DOCX) [file pmed.1002715.s005.docx]

**S4 Fig. Network Meta-Analysis** **for Abstinence at the Longest Follow-Up after Study Completion.**

| **CBT** |  | | | | | | | | | | | |
| --- | --- | --- | --- | --- | --- | --- | --- | --- | --- | --- | --- | --- |
| 0.96  (0.68, 1.35) | **CM** |  | | | | | | | | | | |
| 0.85  (0.57, 1.26) | 0.88  (0.60, 1.29) | **CM+ CBT** |  | | | | | | | | | |
| **0.34**  **(0.15, 0.79)** | **0.36**  **(0.16, 0.80)** | **0.40**  **(0.17, 0.98)** | **CM+CRA** |  | | | | | | | | |
| 0.53  (0.13, 2.18) | 0.55  (0.14, 2.25) | 0.63  (0.15, 2.65) | 1.55  (0.50, 4.86) | **CM+12step** |  | | | | | | | |
| **0.39**  **(0.17, 0.91)** | **0.41**  **(0.17, 0.97)** | 0.46  (0.19, 1.15) | 1.14  (0.52, 2.48) | 0.73  (0.18, 2.91) | **CRA** |  | | | | | | |
| 0.86  (0.23, 3.21) | 0.90  (0.24, 3.30) | 1.02  (0.27, 3.90) | 2.52  (0.91, 6.96) | 1.62  (0.47, 5.64) | 2.21  (0.61, 7.93) | **CRA+NCR** |  | | | | | |
| 0.96  (0.21, 4.34) | 1.00  (0.21, 4.71) | 1.13  (0.24, 5.42) | 2.80  (0.50, 15.76) | 1.80  (0.23, 14.28) | 2.46  (0.43, 13.91) | 1.11  (0.15, 8.26) | **MBT** |  | | | | |
| **1.79**  **(1.13, 2.84)** | **1.86**  **(1.31, 2.66)** | **2.11**  **(1.30, 3.45)** | **5.22**  **(2.16, 12.61)** | 3.36  (0.80, 14.17) | **4.58**  **(1.82, 11.55)** | 2.07  (0.54, 7.95) | 1.86  (0.38, 9.04) | **NCR** |  | | | |
| 1.18  (0.65, 2.14) | 1.23  (0.66, 2.30) | 1.40  (0.71, 2.75) | **3.45**  **(1.27, 9.37)** | 2.22  (0.49, 10.10) | **3.03**  **(1.09, 8.41)** | 1.37  (0.33, 5.70) | 1.23  (0.24, 6.24) | 0.66  (0.33, 1.32) | **SEPT** |  | | |
| 1.06  (0.75, 1.49) | 1.10  (0.83, 1.46) | 1.25  (0.82, 1.91) | **3.08**  **(1.33, 7.17)** | 1.99  (0.48, 8.19) | **2.71**  **(1.12, 6.54)** | 1.23  (0.33, 4.59) | 1.10  (0.23, 5.18) | **0.59**  **(0.40, 0.88)** | 0.89  (0.50, 1.61) | **TAU** |  | |
| 1.24  (0.80, 1.91) | 1.29  (0.81, 2.06) | 1.46  (0.86, 2.50) | **3.61**  **(1.45, 8.99)** | 2.33  (0.54, 9.99) | **3.17**  **(1.24, 8.08)** | 1.44  (0.37, 5.61) | 1.29  (0.27, 6.21) | 0.69  (0.40, 1.19) | 1.05  (0.57, 1.92) | 1.17  (0.78, 1.75) | **12 step** |  |
| 1.27  (0.34, 4.76) | 1.33  (0.36, 4.90) | 1.50  (0.39, 5.79) | **3.71**  **(1.34, 10.33)** | 2.39  (0.67, 8.59) | 3.26  (0.90, 11.77) | 1.48  (0.43, 5.12) | 1.33  (0.18, 9.86) | 0.71  (0.18, 2.74) | 1.08  (0.26, 4.50) | 1.20  (0.32, 4.53) | 1.03  (0.26, 4.04) | **12step+NCR** |

Psychosocial treatment Abstinence at follow-up (OR [95% Cl])

**Notes**. Psychosocial treatments are reported in alphabetical order. Comparisons should be read from left to right. The abstinence at the longest follow-up after study completion estimate is located at the intersection of the column-defining treatment and the row-defining treatment. An OR above 1 favors the column-defining treatment, while an OR below 1 favors the row-defining treatment. To obtain ORs for comparisons in the opposing direction, reciprocals should be taken. Significant results are in bold and underlined. CBT: cognitive behavioural therapy; CM: contingency management; CRA: community reinforcement approach; MBT: meditation based therapies; NCR: not contingent rewards; SEPT: supportive expressive psychodynamic therapy; TAU: treatment as usual; 12 step: twelve-step programme.
