# Supplementary material for: Comparative efficacy and acceptability of psychosocial interventions for individuals with cocaine and amphetamine addiction: A systematic review and network meta-analysis
Source: PLoS Med. 2018 Dec 26;15(12):e1002715. doi: 10.1371/journal.pmed.1002715 (PMC6306153; doi:10.1371/journal.pmed.1002715)
Supplement: S5 Fig — (DOCX) [file pmed.1002715.s006.docx]

**S5 Fig. Network Meta-Analysis for** **the Longest Duration of Abstinence at 12 Weeks.**

| **CBT** |  |  |  |  |  |  |  |  |
| --- | --- | --- | --- | --- | --- | --- | --- | --- |
| **-0.56**  **(-0.88, -0.23)** | **CM** |  |  |  |  |  |  |  |
| **-0.69**  **(-1.12, -0.26)** | -0.13  (-0.55, 0.29) | **CM + CBT** |  |  |  |  |  |  |
| -0.04  (-0.43, 0.35) | **0.52**  **(0.22, 0.81)** | **0.65**  **(0.18, 1.12)** | **NCR** |  |  |  |  |  |
| 0.06  (-0.27, 0.38) | **0.62**  **(0.43, 0.80)** | **0.75**  **(0.31, 1.19)** | 0.10  (-0.22, 0.42) | **TAU** |  |  |  |  |
| 0.08  (-0.58, 0.73) | 0.64  (-0.10, 1.37) | 0.77  (-0.02, 1.55) | 0.12  (-0.65, 0.88) | 0.02  (-0.71, 0.75) | **12 step** |  |  |  |
| - | - | - | - | - | - | **CM + CRA** |  |  |
| - | - | - | - | - | - | 0.72  (-0.10, 1.54) | **CRA** |  |
| - | - | - | - | - | - | 0.49  (-0.34, 1.31) | -0.24  (-1.40, 0.93) | **12 step + NCR** |

Psychosocial treatment Longest duration of abstinence at 12 weeks (SMD [95% Cl])

**Notes**. Psychosocial treatments are reported in alphabetical order. Comparisons should be read from left to right. The “longest duration of abstinence at 12 weeks“ estimate is located at the intersection of the column-defining treatment and the row-defining treatment. An SMD above 0 favors the column-defining treatment. Significant results are in bold and underlined. CBT: cognitive behavioural therapy; CM: contingency management; CRA: community reinforcement approach; NCR: not contingent rewards; TAU: treatment as usual; 12 step: twelve-step programme.
