# Supplementary material for: Comparative efficacy and acceptability of psychosocial interventions for individuals with cocaine and amphetamine addiction: A systematic review and network meta-analysis
Source: PLoS Med. 2018 Dec 26;15(12):e1002715. doi: 10.1371/journal.pmed.1002715 (PMC6306153; doi:10.1371/journal.pmed.1002715)

**S7a Fig. Evaluation of the Local Incoherence. Abstinence at 12 Weeks.**


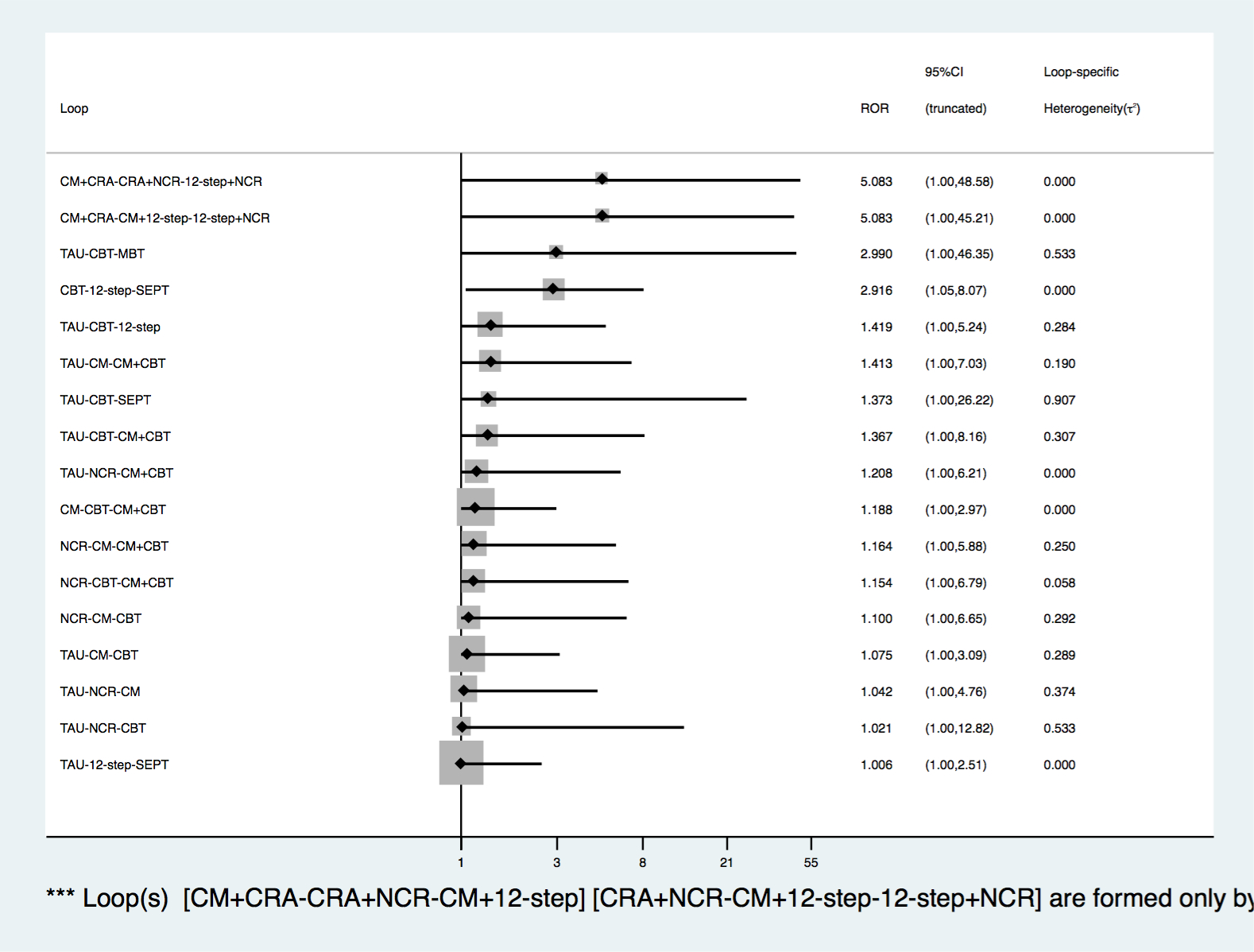


**S7b Fig. Evaluation of the Local Incoherence. Abstinence at the End of Treatment.**


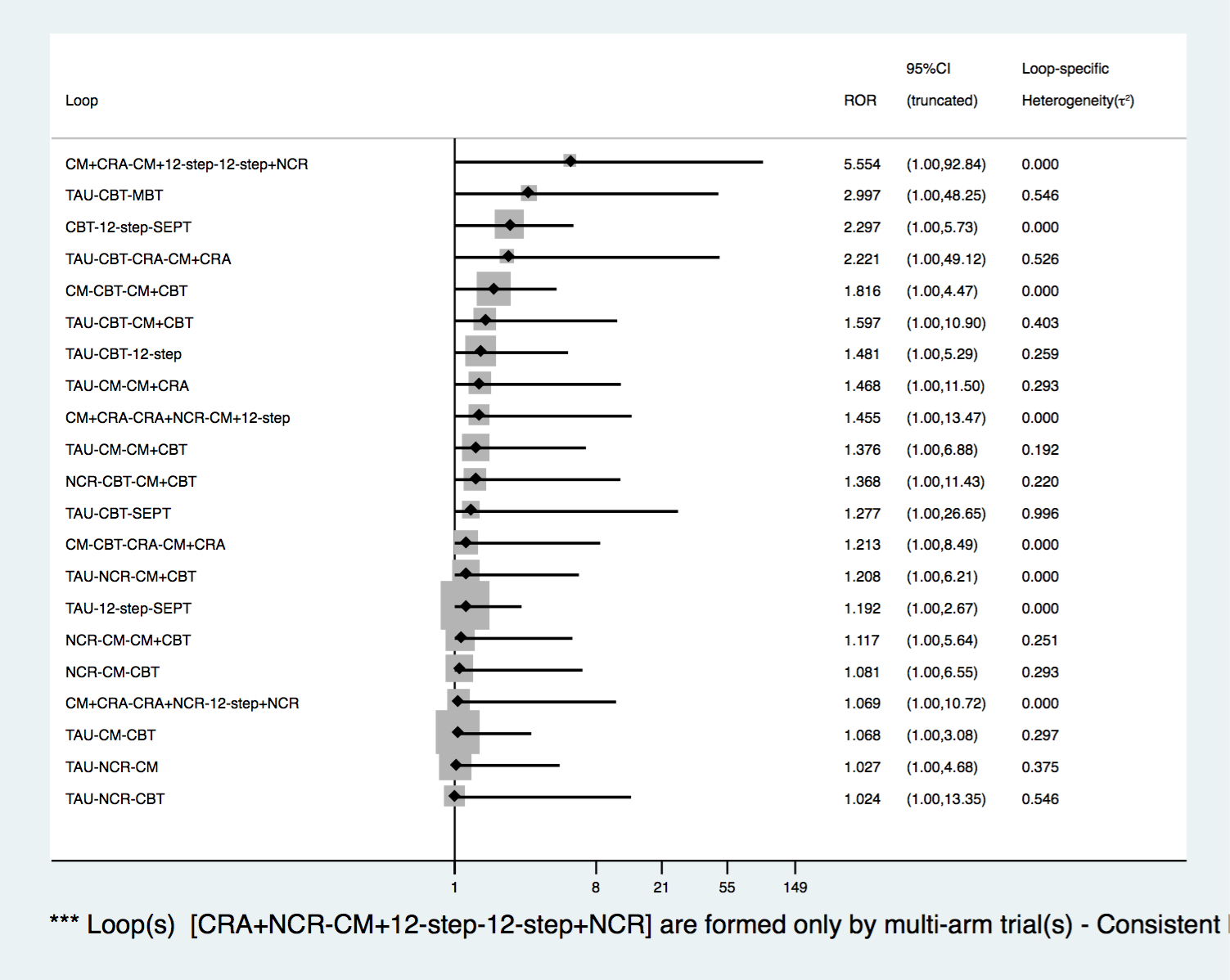


**S7c Fig. Evaluation of the Local Incoherence. Abstinence at the Longest Follow-Up after Study Completion.**


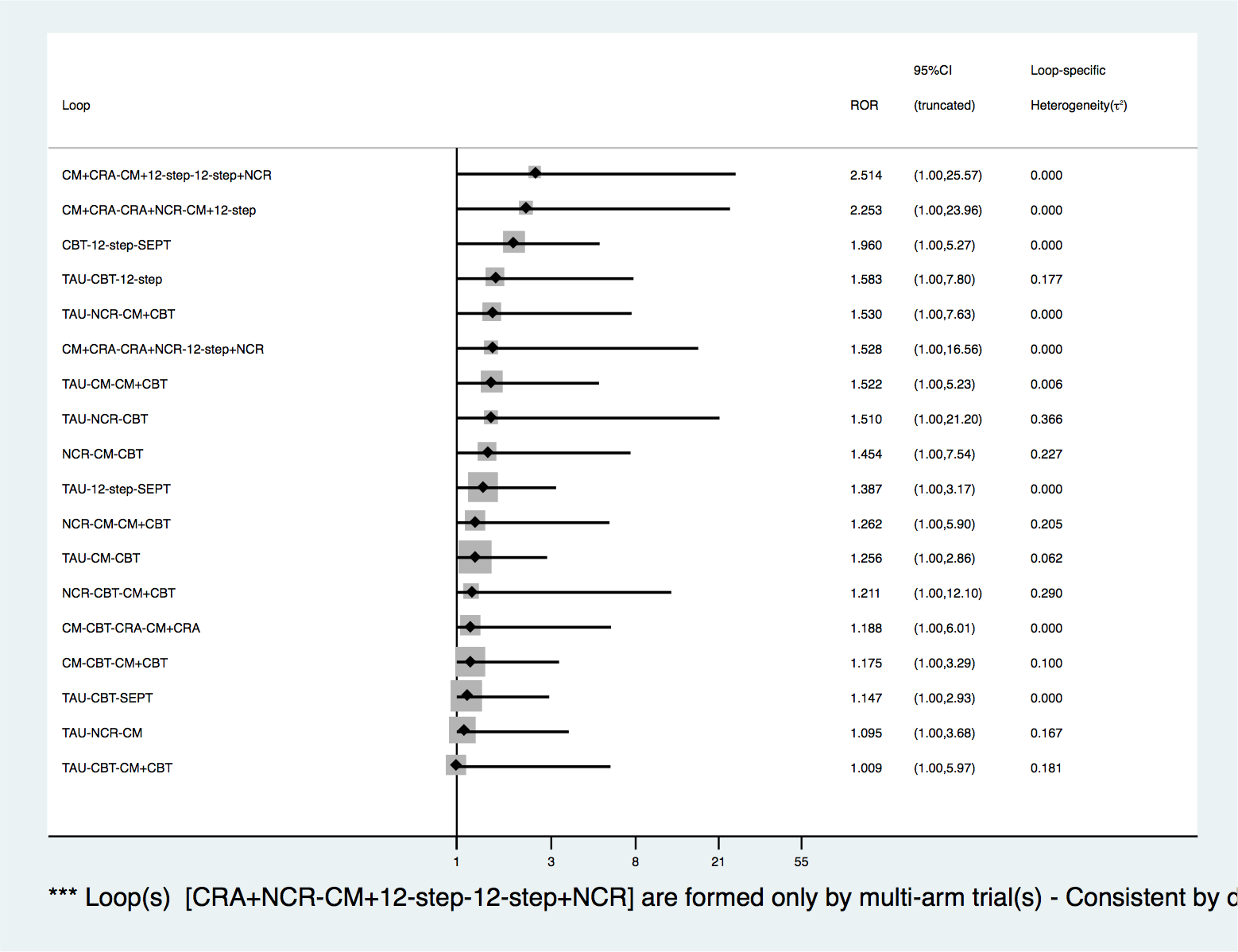


**S7d Fig. Evaluation of the Local Incoherence. Dropout due to any Cause at 12 Weeks.**


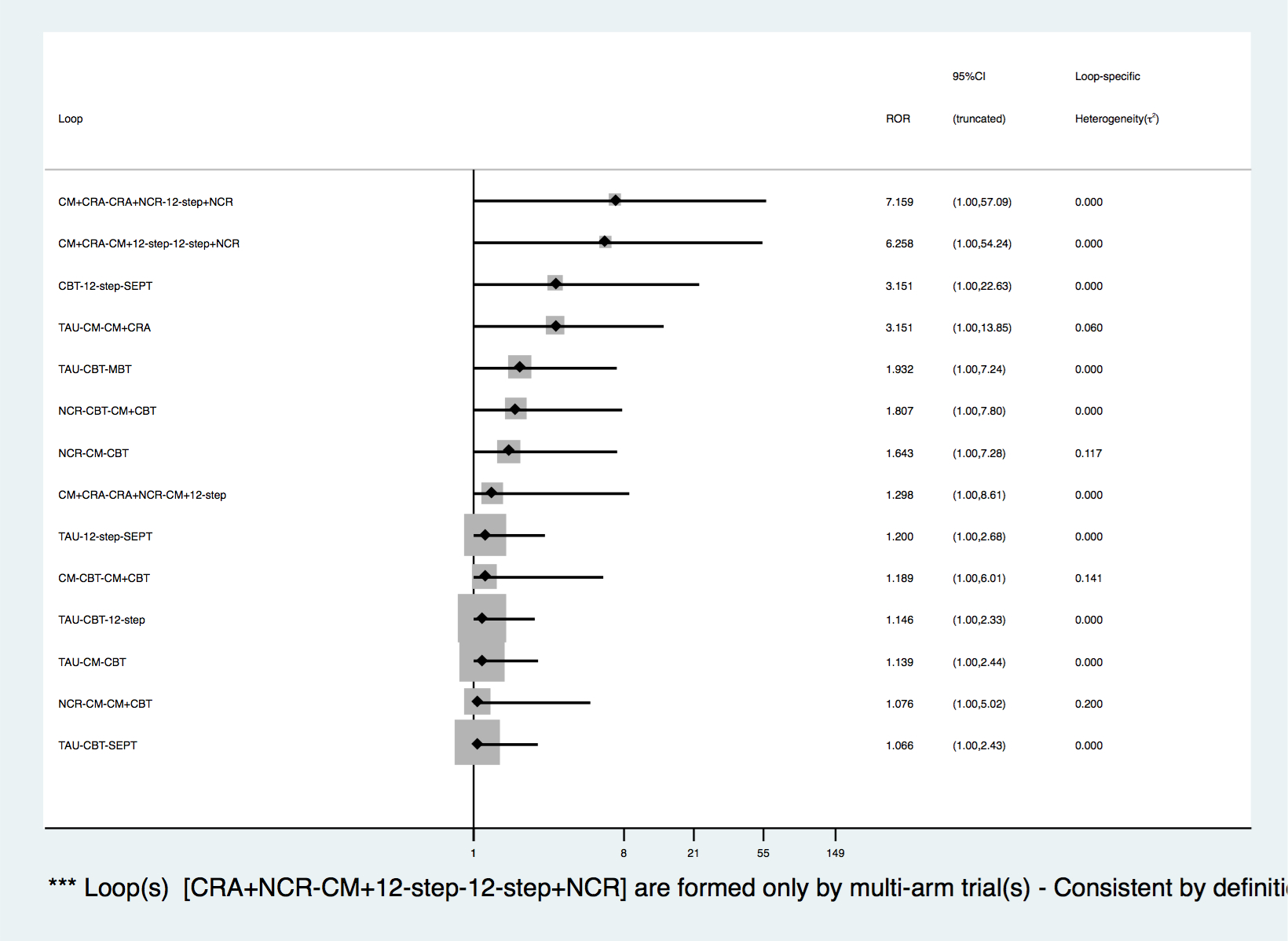


**S7e Fig. Evaluation of the Local Incoherence. Dropout due to any Cause at the End of Treatment.**


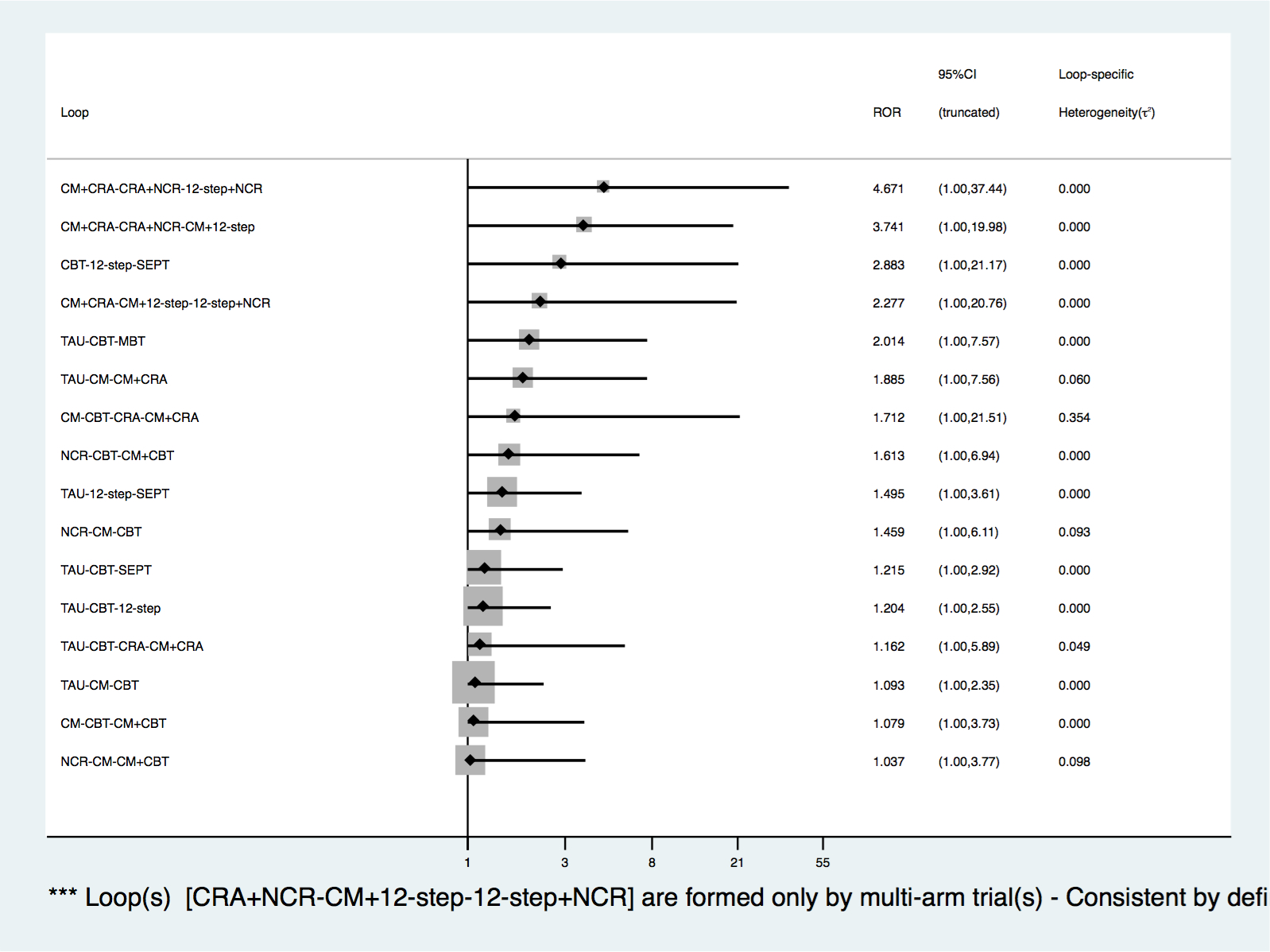


**S7f Fig. Evaluation of the Local Incoherence. Longest Duration of Abstinence at 12 Weeks.**


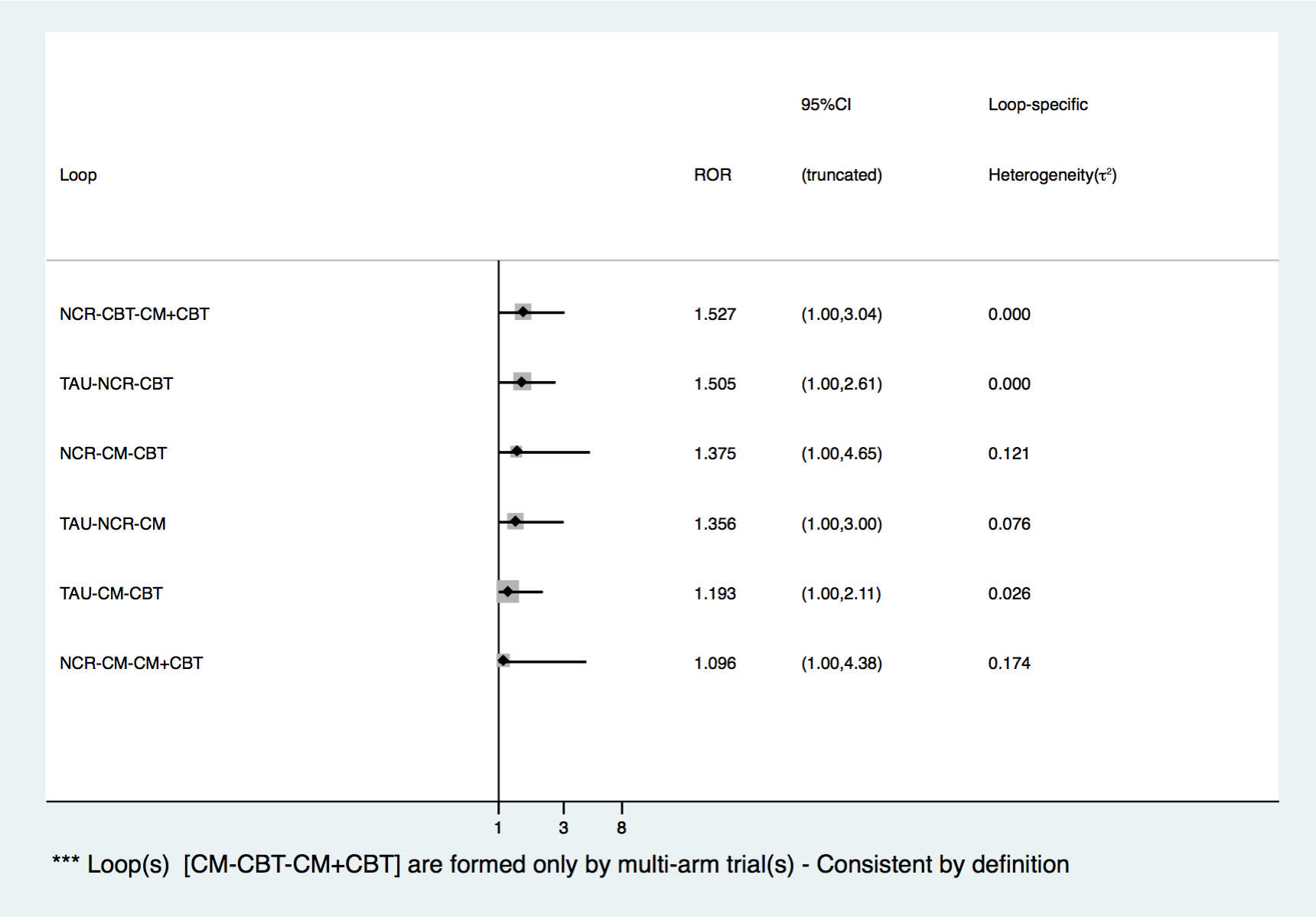


**S7g Fig. Evaluation of the Local Incoherence. Longest Duration of Abstinence at the End of Treatment.**


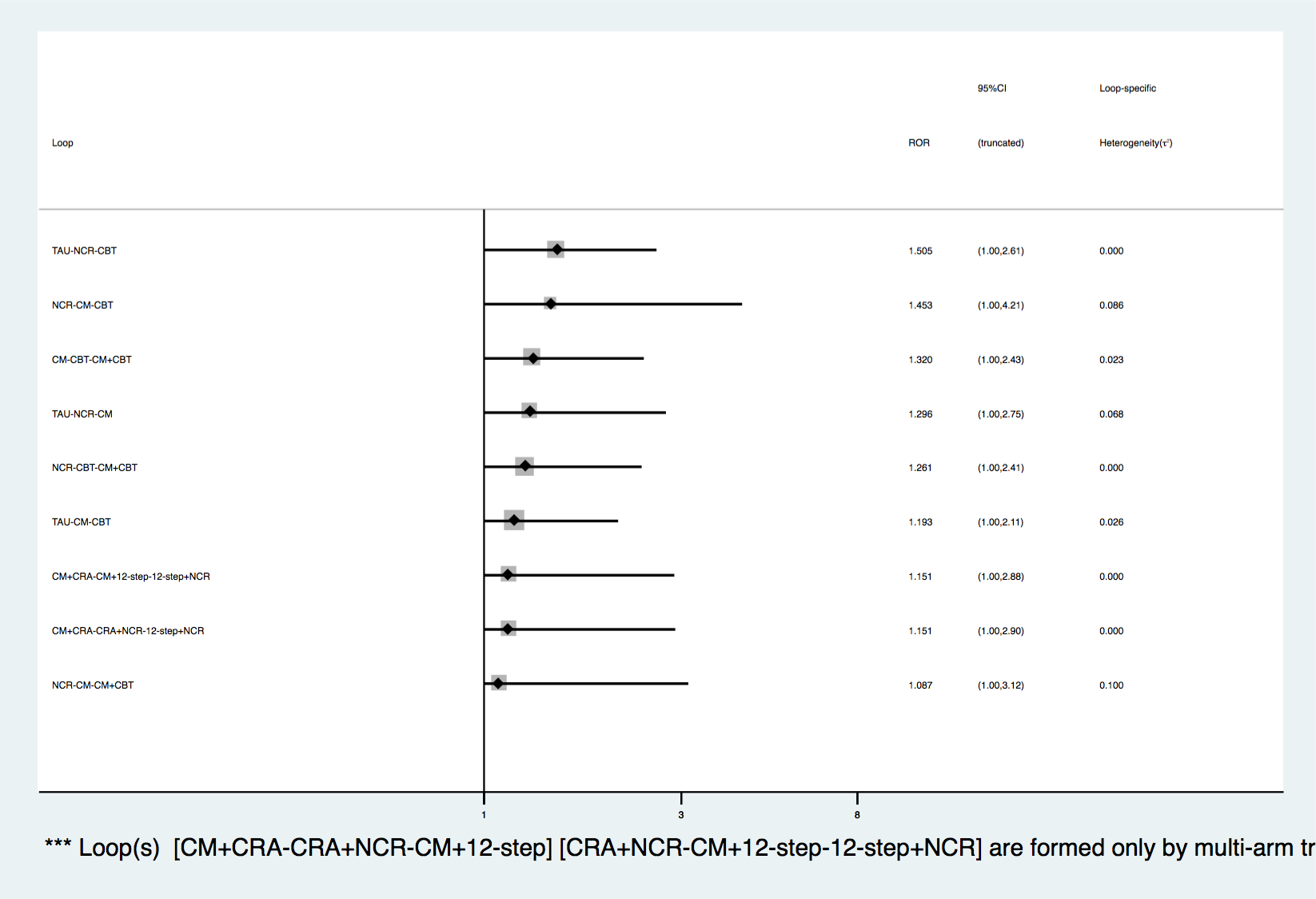

Supplement: S7 Fig — (DOCX) [file pmed.1002715.s008.docx]
