# Supplementary material for: Comparative efficacy and acceptability of psychosocial interventions for individuals with cocaine and amphetamine addiction: A systematic review and network meta-analysis
Source: PLoS Med. 2018 Dec 26;15(12):e1002715. doi: 10.1371/journal.pmed.1002715 (PMC6306153; doi:10.1371/journal.pmed.1002715)
Supplement: S8 Fig — (DOCX) [file pmed.1002715.s009.docx]

**S8a Fig. Comparison-Adjusted Funnel Plot for Abstinence at 12 Weeks.**


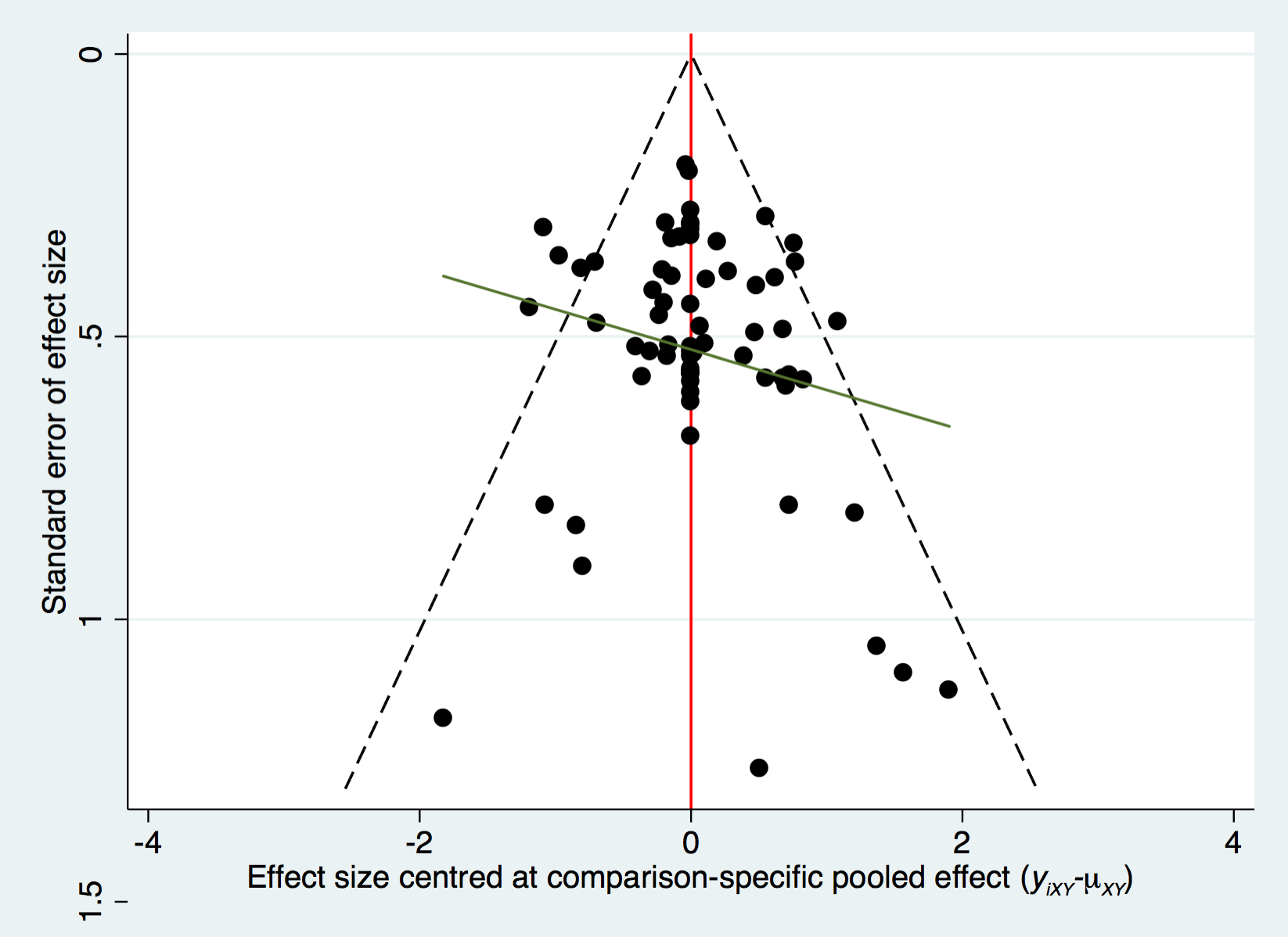


**S8b Fig. Comparison-Adjusted Funnel Plot for Abstinence at the End of Treatment.**

**
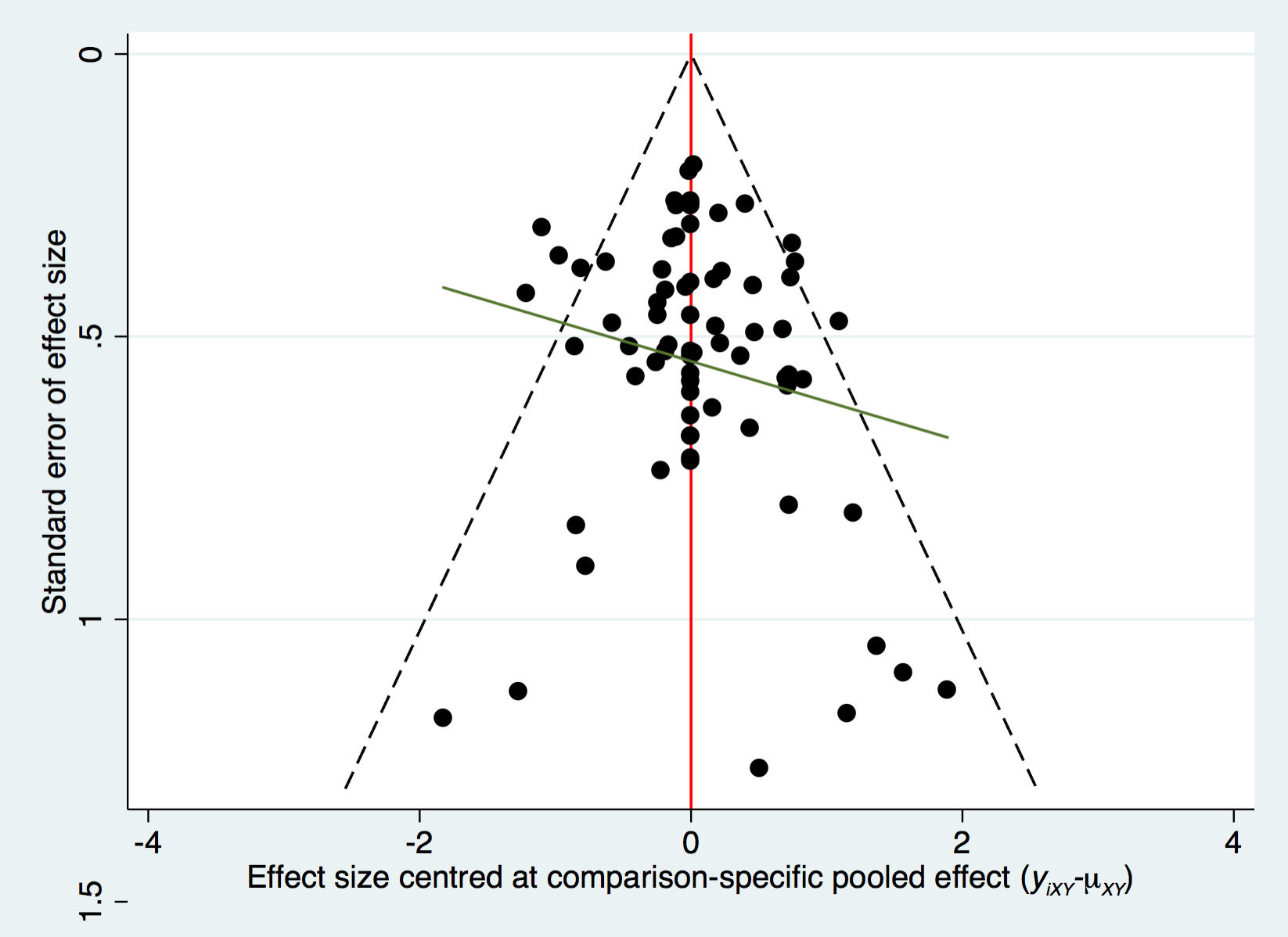
**

**S8c Fig. Comparison-Adjusted Funnel Plot for the Longest Follow-Up after Study Completion.**

**
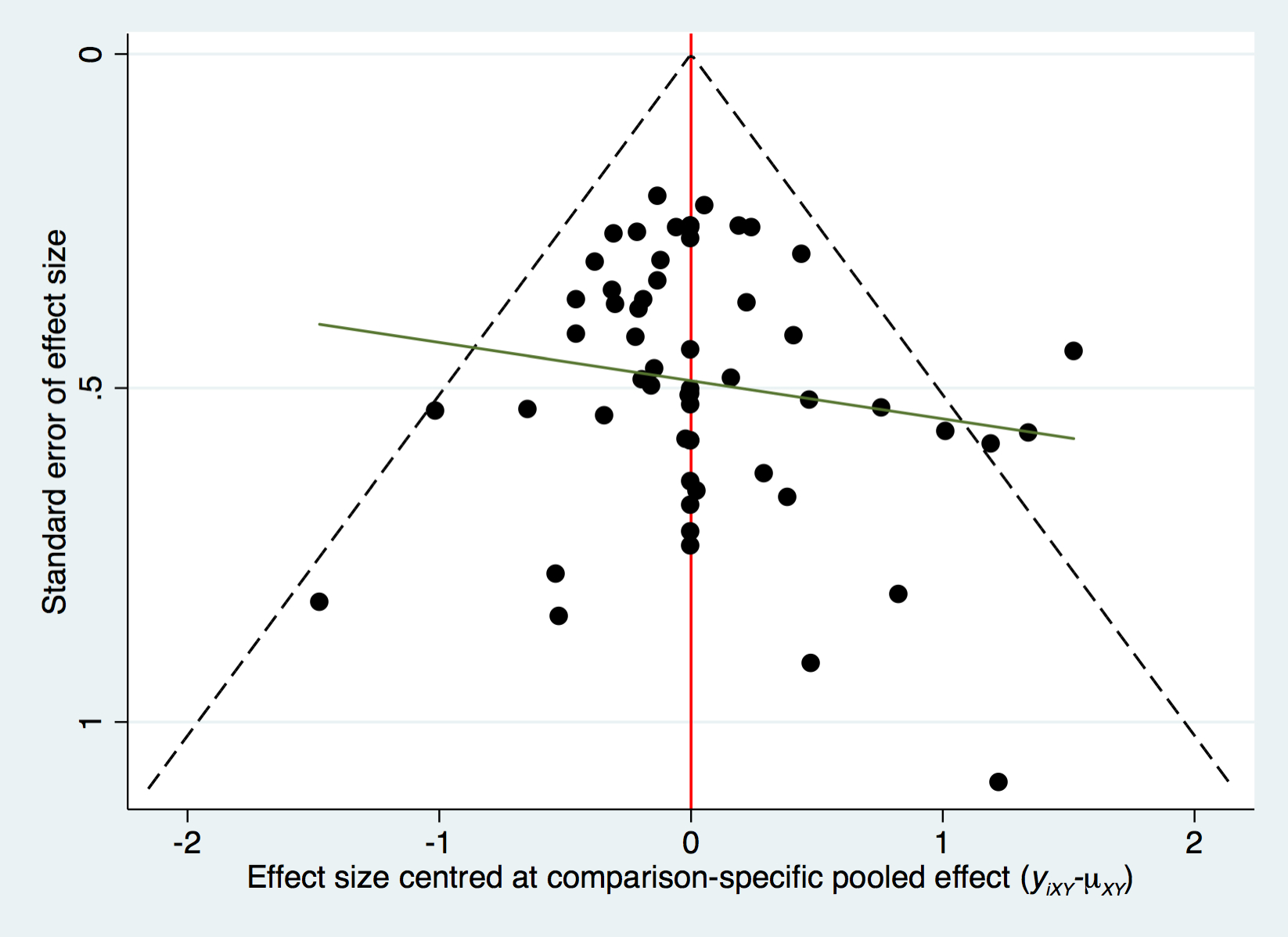
**

**S8d Fig. Comparison-Adjusted Funnel Plot for Dropout due to any Cause at 12 Weeks.**

**
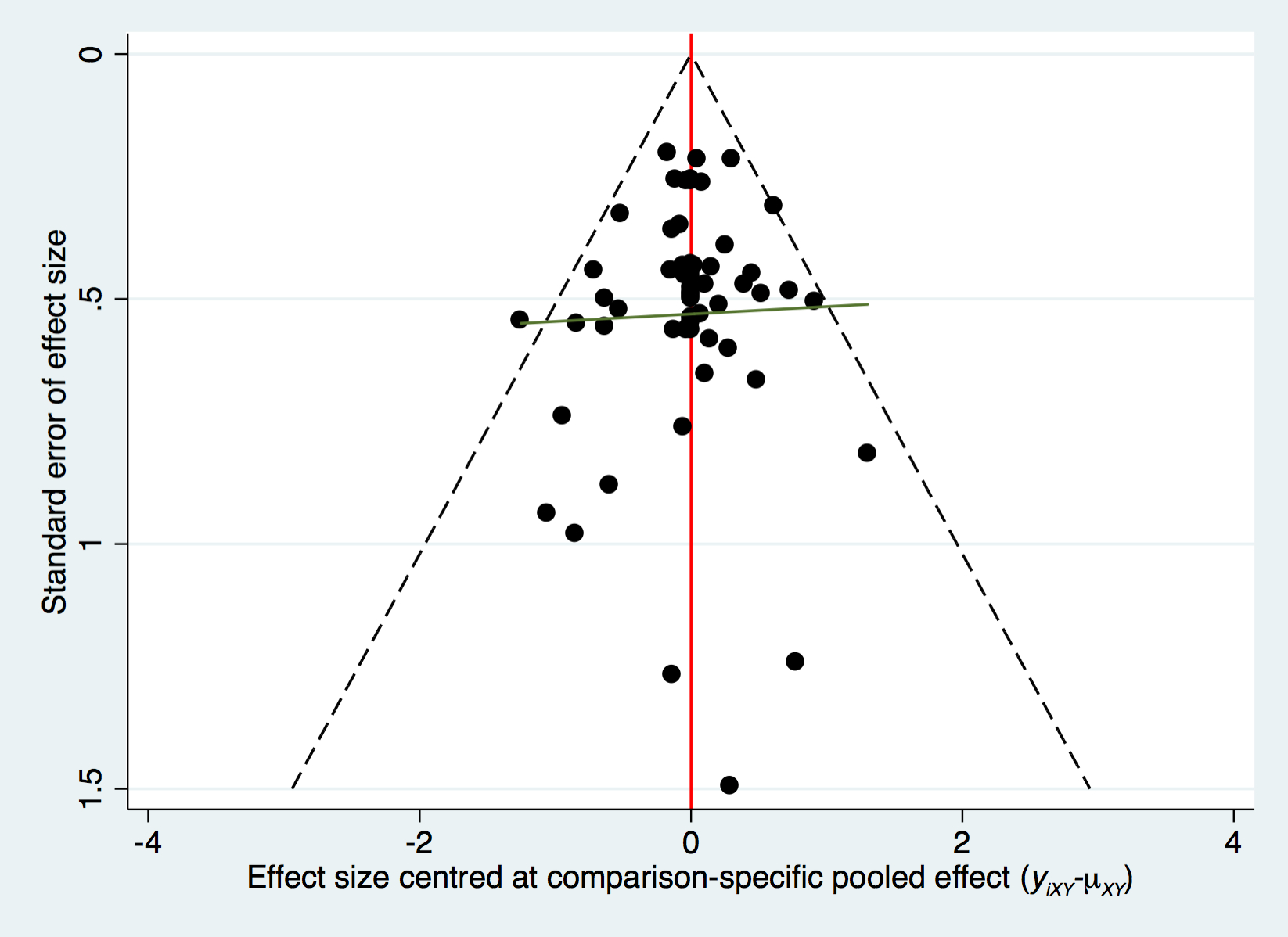
**

**S8e Fig. Comparison-Adjusted Funnel Plot for Dropout due to any Cause at the End of Treatment.**


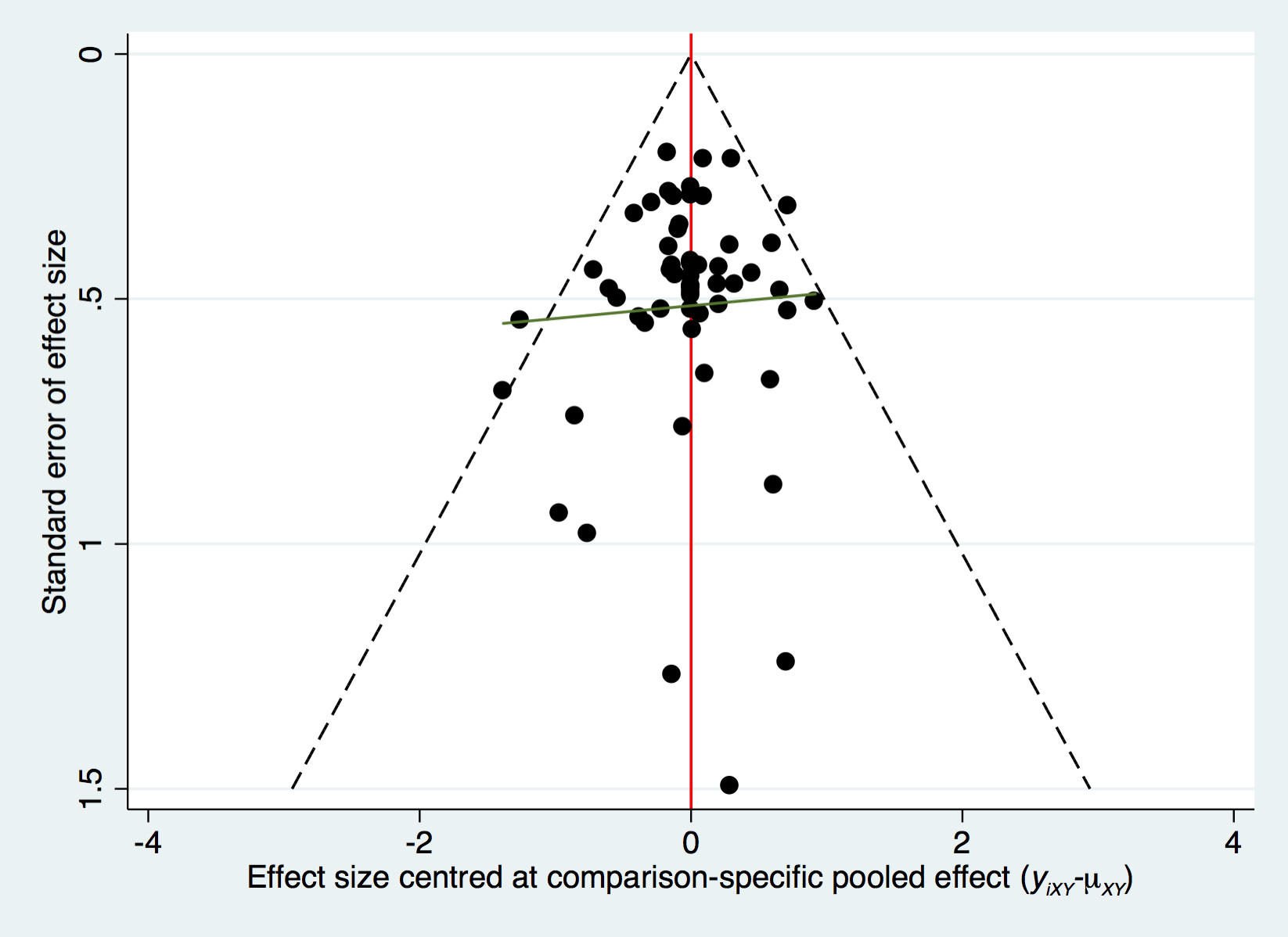


**S8f Fig. Comparison-Adjusted Funnel Plot for the Longest Duration of Abstinence at 12 Weeks.**

**
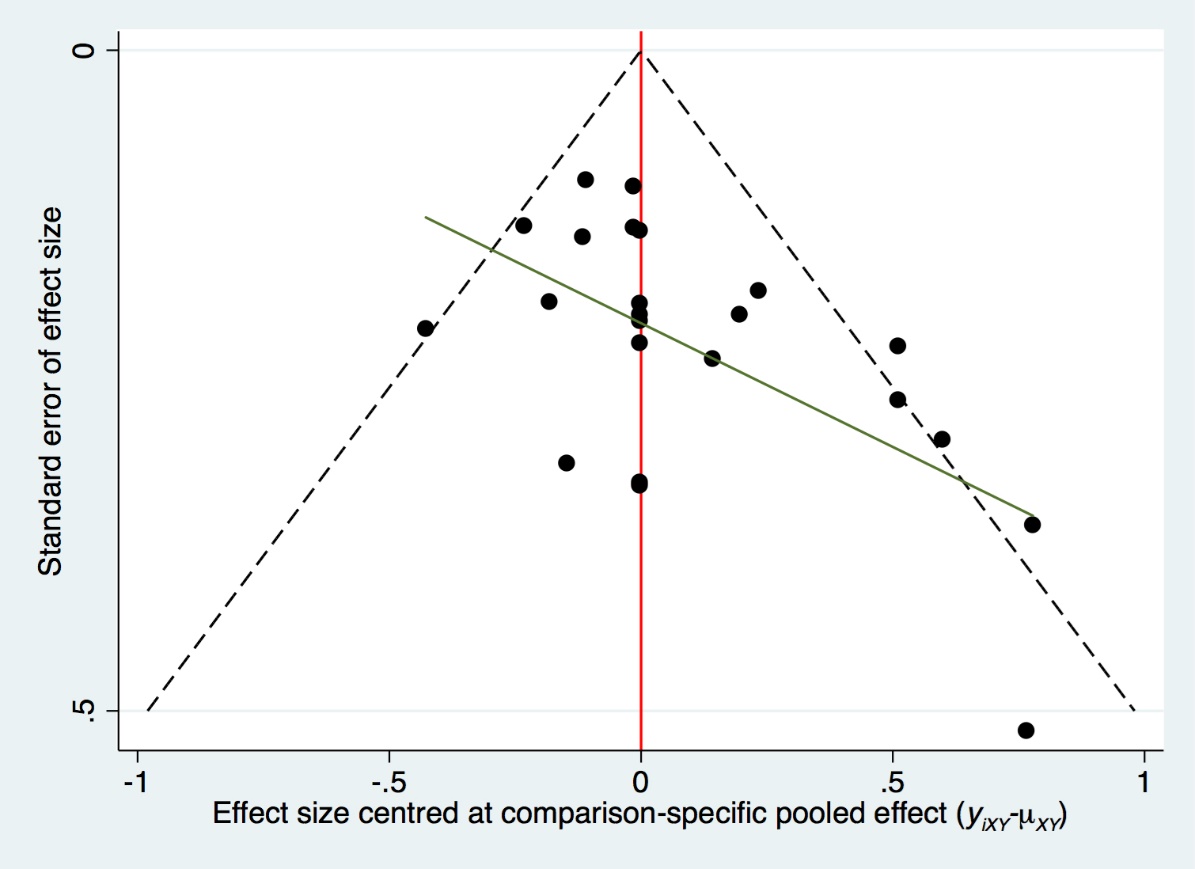
**

**S8g Fig. Comparison-Adjusted Funnel Plot for the Longest Duration of Abstinence at the End of Treatment.**

**
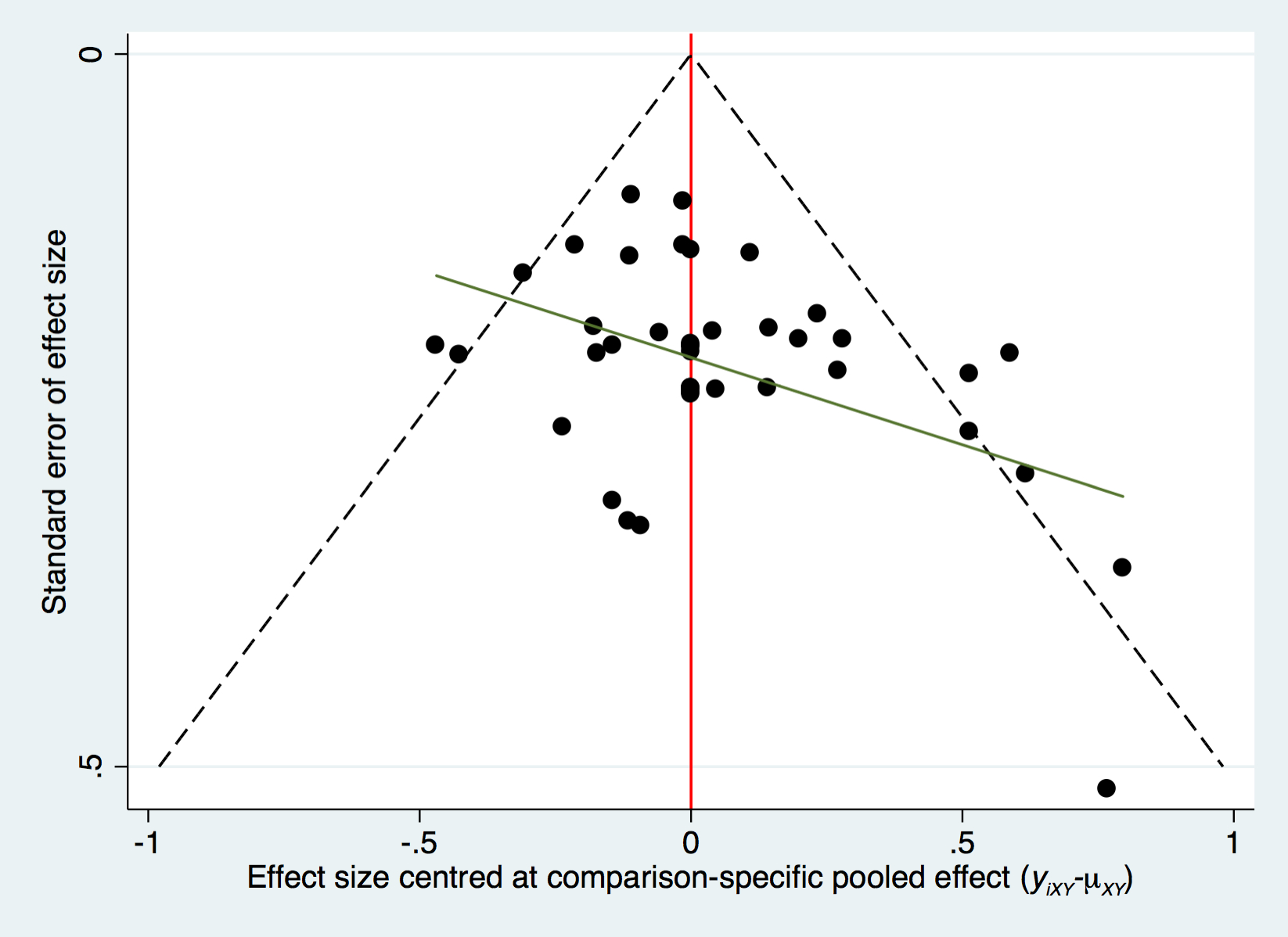
**
