# Supplementary material for: Comparative efficacy and acceptability of psychosocial interventions for individuals with cocaine and amphetamine addiction: A systematic review and network meta-analysis
Source: PLoS Med. 2018 Dec 26;15(12):e1002715. doi: 10.1371/journal.pmed.1002715 (PMC6306153; doi:10.1371/journal.pmed.1002715)

**S9a Fig. Cumulative Probability Plots (Random Effects Model). Abstinence at 12 Weeks.**

**
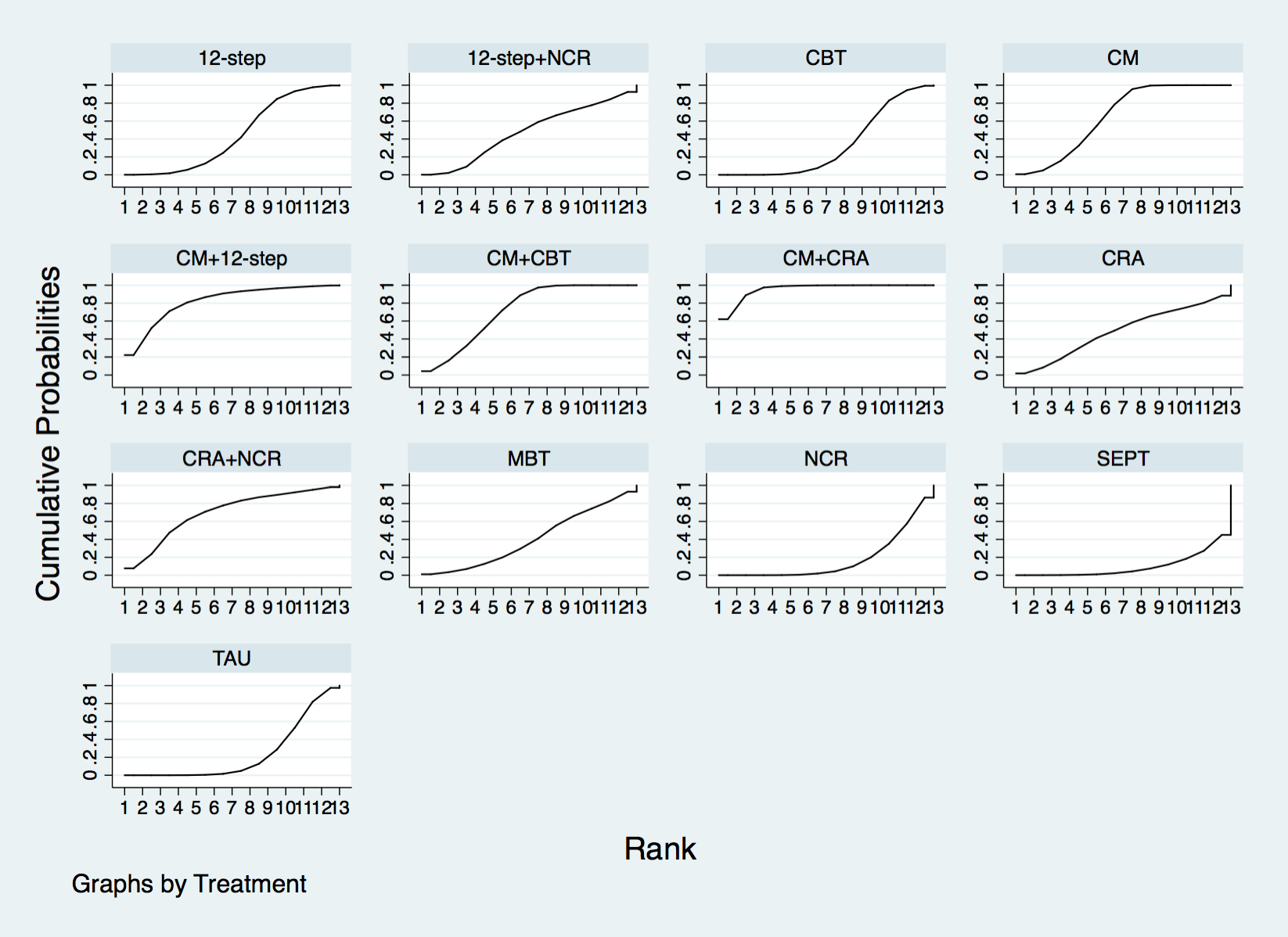
**

**S9b Fig. Cumulative Probability Plots (Random Effects Model). Abstinence at the End of Treatment.**

**
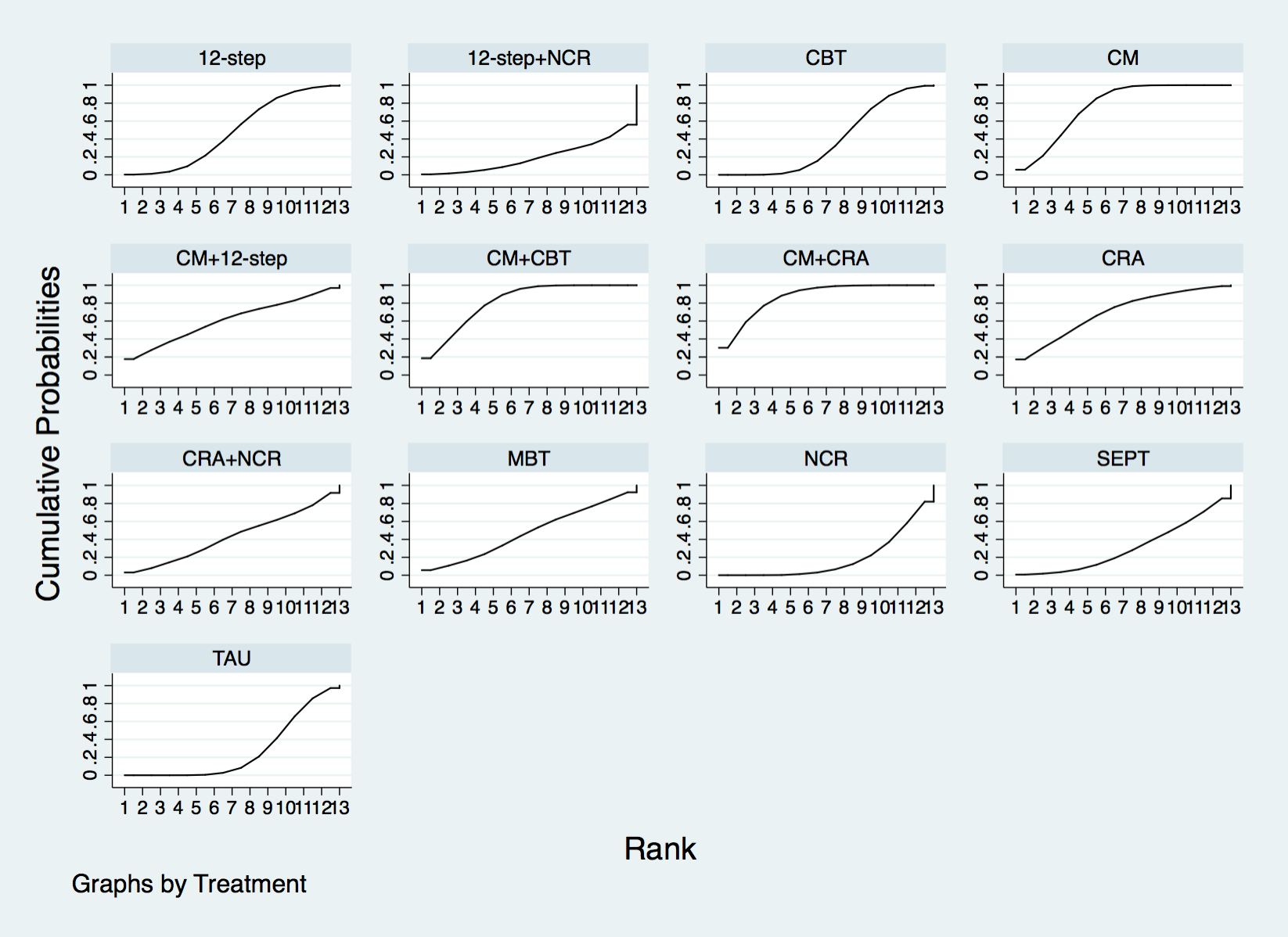
**

**S9c Fig. Cumulative Probability Plots (Random Effects Model). Longest Follow-Up after Study Completion.**

**
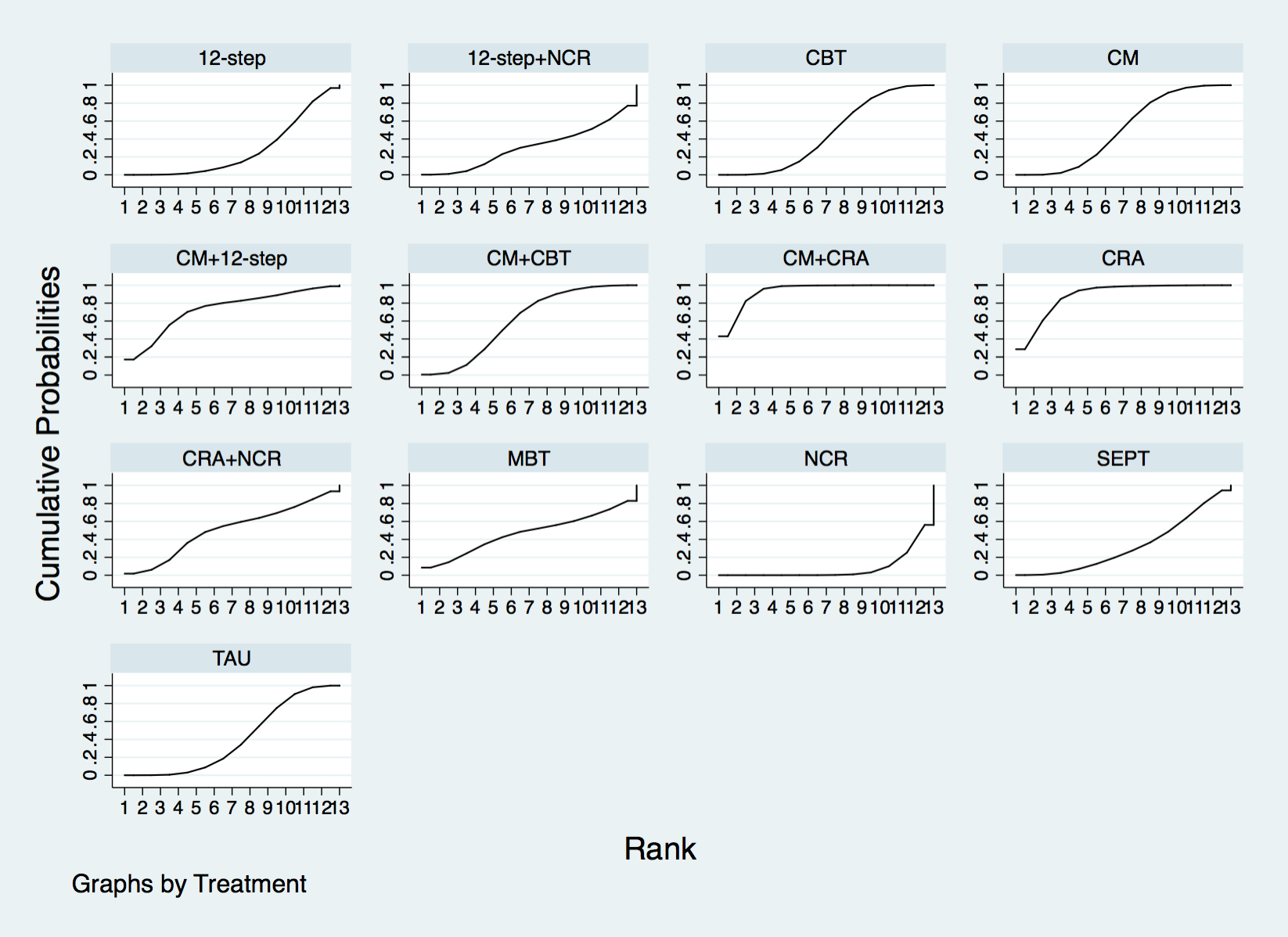
**

**S9d Fig. Cumulative Probability Plots (Random Effects Model). Dropout due to any Cause at 12 Weeks.**

**
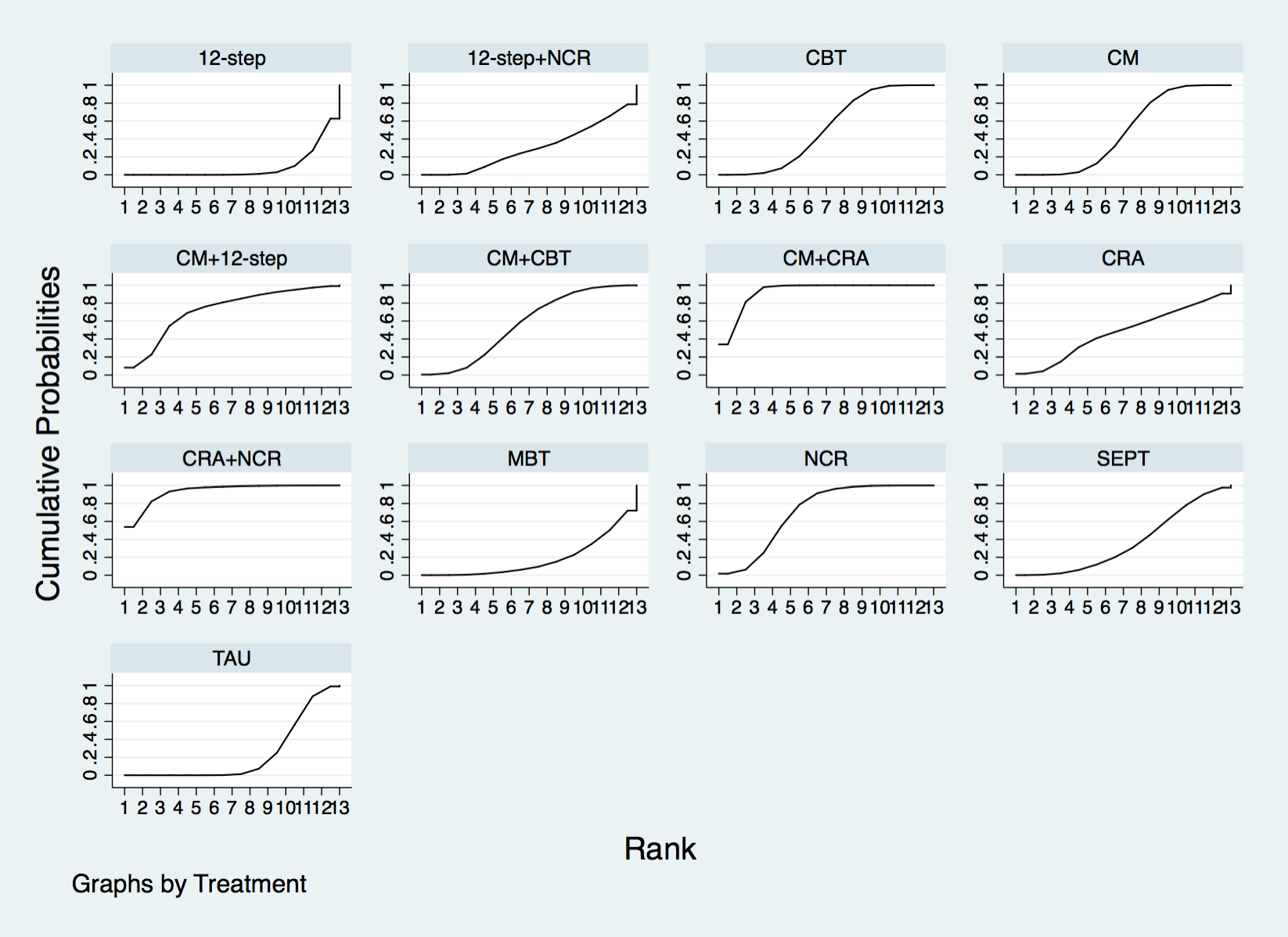
**

**S9e Fig. Cumulative Probability Plots (Random Effects Model). Dropout due to any Cause at the End of Treatment.**


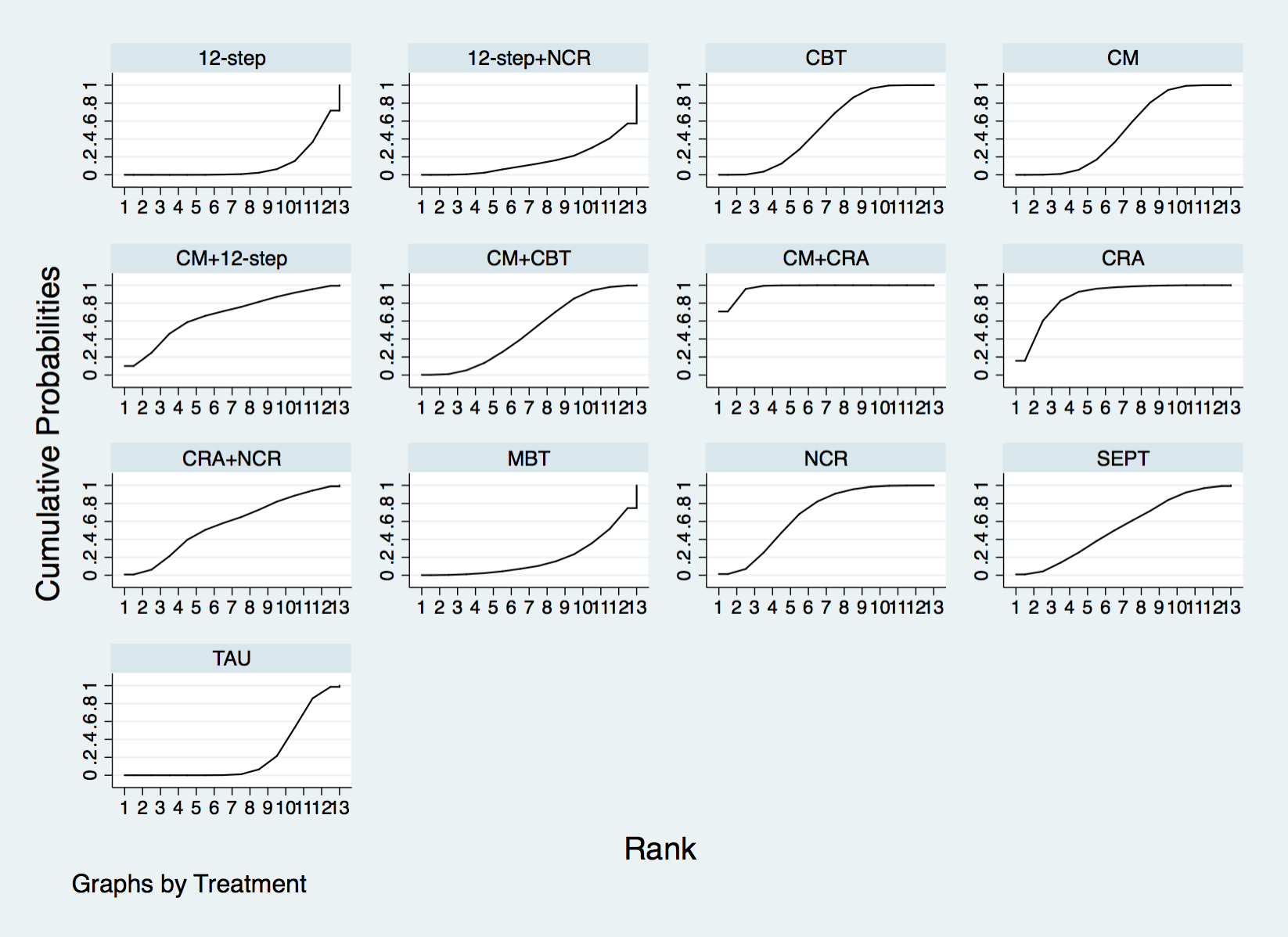


**S9f Fig. Cumulative Probability Plots (Random Effects Model). Longest Duration of Abstinence at 12 Weeks.**


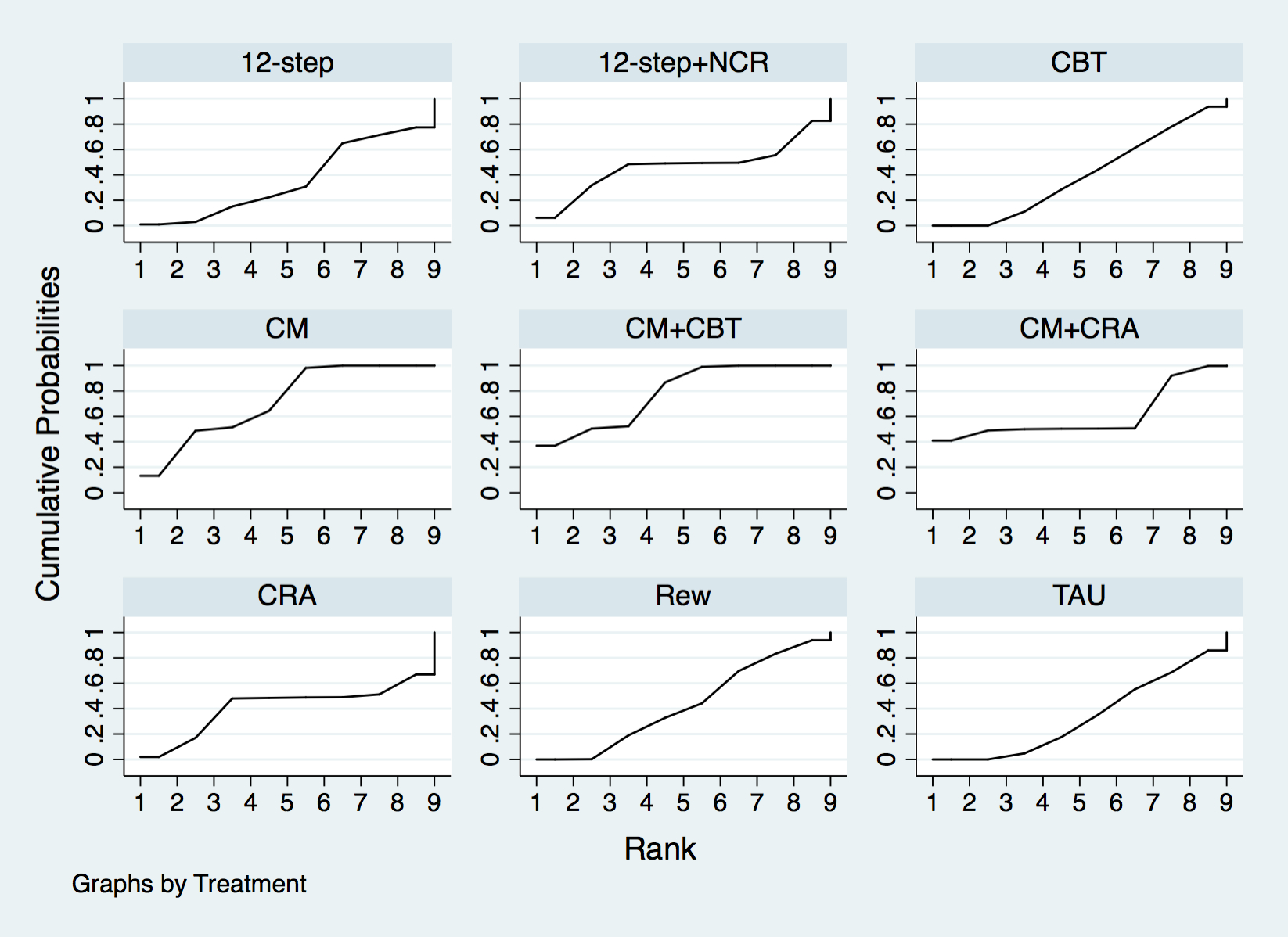


**S9g Fig. Cumulative Probability Plots (Random Effects Model). Longest Duration of Abstinence at the End of Treatment.**


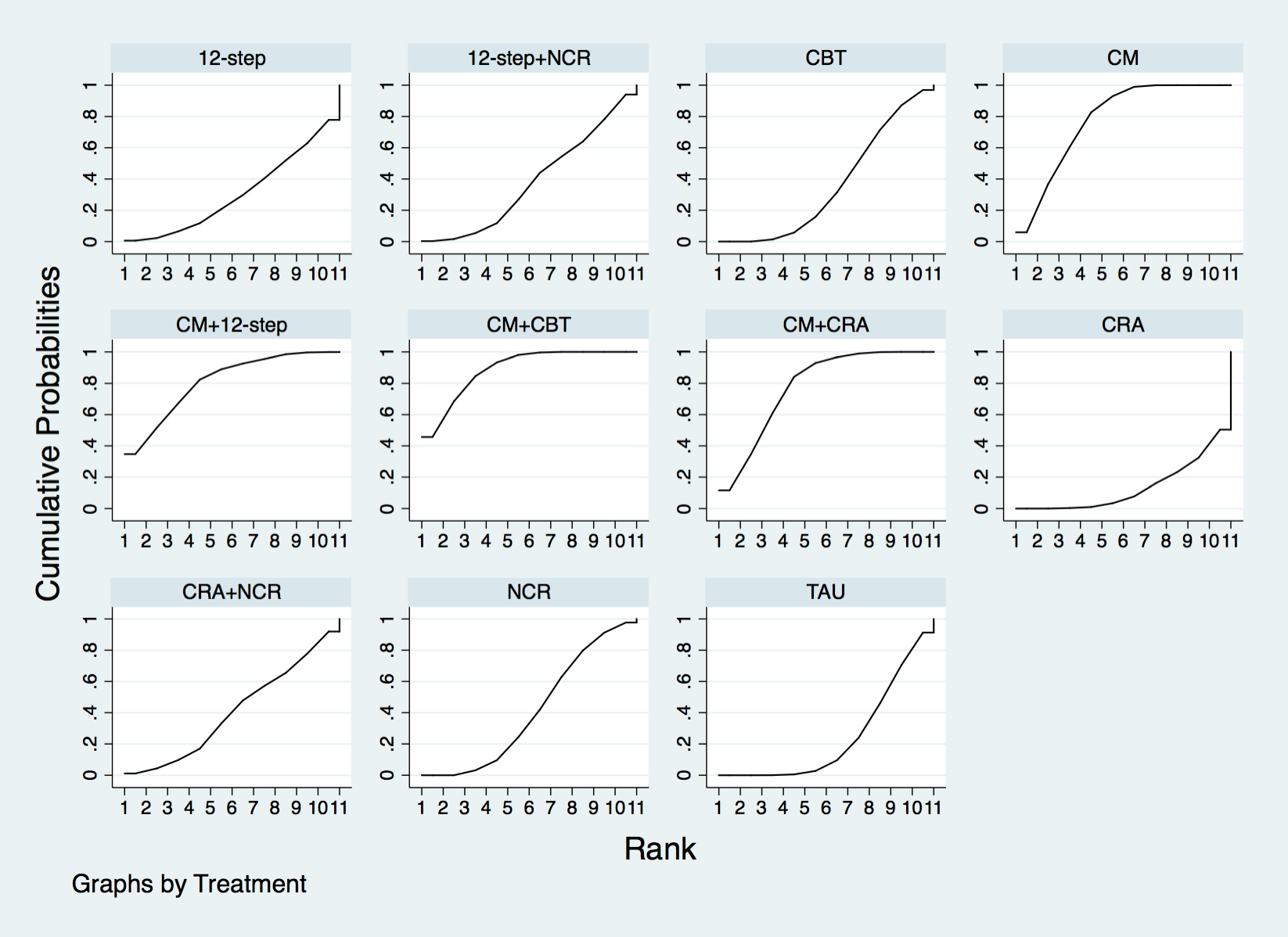

Supplement: S9 Fig — (DOCX) [file pmed.1002715.s010.docx]
