# Supplementary material for: Comparative efficacy and acceptability of psychosocial interventions for individuals with cocaine and amphetamine addiction: A systematic review and network meta-analysis
Source: PLoS Med. 2018 Dec 26;15(12):e1002715. doi: 10.1371/journal.pmed.1002715 (PMC6306153; doi:10.1371/journal.pmed.1002715)
Supplement: S10 Fig — (DOCX) [file pmed.1002715.s011.docx]

**S10 Fig. Sensitivity Network Meta-Analysis for Abstinence at the End of Treatment and Dropout at the End of Treatment by Considering only the Trials on Individuals Addicted to Cocaine and no other Stimulant.**

| **CBT** | 1.07  (0.66, 1.74) | 1.14  (0.64, 2.01) | **0.41**  **(0.21, 0.78)** | 0.75  (0.23, 2.41) | 0.53  (0.26, 1.09) | 0.92  (0.34, 2.45) | 2.40  (0.78, 7.34) | 1.13  (0.62, 2.04) | 0.96  (0.52, 1.77) | 1.44  (0.99, 2.10) | 1.61  (0.93, 2.81) | 1.88  (0.63, 5.58) |
| --- | --- | --- | --- | --- | --- | --- | --- | --- | --- | --- | --- | --- |
| **0.45**  **(0.28,0.73)** | **CM** | 1.06  (0.63, 1.76) | **0.38**  **(0.20, 0.71)** | 0.70  (0.22, 2.23) | 0.50  (0.23, 1.06) | 0.85  (0.32, 2.26) | 2.23  (0.72, 6.95) | 1.05  (0.70, 1.58) | 0.89  (0.44, 1.82) | 1.34  (0.88, 2.05) | 1.50  (0.79, 2.87) | 1.75  (0.59, 5.17) |
| **0.46**  **(0.27, 0.77)** | 1.02  (0.61, 1.70) | **CM+CBT** | **0.36**  **(0.17, 0.77)** | 0.66  (0.19, 2.27) | 0.47  (0.20, 1.09) | 0.81  (0.28, 2.34) | 2.11  (0.63, 7.05) | 0.99  (0.54, 1.82) | 0.84  (0.38, 1.88) | 1.27  (0.71, 2.28) | 1.42  (0.67, 3.01) | 1.66  (0.52, 5.27) |
| **0.38**  **(0.17, 0.84)** | 0.84  (0.40, 1.77) | 0.83  (0.35, 1.96) | **CM+CRA** | 1.84  (0.69, 4.89) | 1.31  (0.73, 2.34) | **2.25**  **(1.07, 4.72)** | **5.88**  **(1.73, 19.91)** | **2.77**  **(1.32, 5.78)** | **2.35**  **(1.01, 5.43)** | **3.54**  **(1.91, 6.54)** | **3.96**  **(1.81, 8.67)** | **4.61**  **(1.91, 11.14)** |
| 0.58  (0.13, 2.52) | 1.29  (0.30, 5.49) | 1.27  (0.28, 5.73) | 1.53  (0.44, 5.29) | **CM +12step** | 0.71  (0.23, 2.20) | 1.22  (0.47, 3.20) | 3.20  (0.67, 15.25) | 1.50  (0.44, 5.11) | 1.28  (0.35, 4.62) | 1.92  (0.61, 6.09) | 2.15  (0.62, 7.53) | 2.51  (0.93, 6.74) |
| 0.52  (0.18, 1.48) | 1.15  (0.40, 3.36) | 1.13  (0.37, 3.50) | 1.37  (0.53, 3.55) | 0.89  (0.19, 4.25) | **CRA** | 1.72  (0.68, 4.38) | **4.49**  **(1.25, 16.15)** | 2.11  (0.91, 4.89) | 1.79  (0.72, 4.45) | **2.70**  **(1.31, 5.58)** | **3.03**  **(1.28, 7.17)** | **3.52**  **(1.25, 9.97)** |
| 0.85  (0.23, 3.14) | 1.90  (0.53, 6.81) | 1.86  (0.48, 7.17) | 2.25  (0.79, 6.36) | 1.47  (0.39, 5.50) | 1.64  (0.40, 6.73) | **CRA+ NCR** | 2.61  (0.63, 10.87) | 1.23  (0.43, 3.49) | 1.04  (0.34, 3.19) | 1.57  (0.60, 4.11) | 1.76  (0.60, 5.17) | 2.05  (0.83, 5.07) |
| 0.54  (0.15, 1.98) | 1.21  (0.33, 4.43) | 1.18  (0.31, 4.58) | 1.43  (0.34, 6.05) | 0.93  (0.14, 6.24) | 1.04  (0.21, 5.31) | 0.64  (0.11, 3.77) | **MBT** | 0.47  (0.14, 1.56) | 0.40  (0.12, 1.35) | 0.60  (0.21, 1.73) | 0.67  (0.21, 2.19) | 0.78  (0.17, 3.53) |
| 1.31  (0.72, 2.38) | **2.93**  **(1.89, 4.55)** | **2.88**  **(1.54, 5.39)** | **3.48**  **(1.50, 8.07)** | 2.27  (0.51, 10.13) | 2.54  (0.82, 7.88) | 1.55  (0.41, 5.89) | 2.43  (0.63, 9.36) | **NCR** | 0.85  (0.38, 1.89) | 1.28  (0.73, 2.25) | 1.43  (0.68, 3.01) | 1.67  (0.53, 5.25) |
| 1.15  (0.55, 2.41) | **2.56**  **(1.13, 5.83)** | **2.52**  **(1.05, 6.02)** | **3.04**  **(1.08, 8.54)** | 1.98  (0.40, 9.94) | 2.22  (0.63, 7.82) | 1.35  (0.31, 5.86) | 2.13  (0.50, 9.03) | 0.87  (0.36, 2.13) | **SEPT** | 1.51  (0.82, 2.78) | 1.69  (0.87, 3.26) | 1.97  (0.58, 6.61) |
| 1.11  (0.73, 1.67) | **2.48**  **(1.65, 3.73)** | **2.43**  **(1.40, 4.22)** | **2.93**  **(1.39, 6.19)** | 1.91  (0.45, 8.12) | 2.15  (0.75, 6.17) | 1.31  (0.36, 4.69) | 2.05  (0.60, 7.07) | 0.84  (0.49, 1.44) | 0.97  (0.46, 2.04) | **TAU** | 1.12  (0.66, 1.90) | 1.30  (0.45, 3.81) |
| 0.89  (0.54, 1.46) | **1.99**  **(1.07, 3.70)** | 1.96  (0.99, 3.85) | 2.36  (0.98, 5.70) | 1.54  (0.34, 7.03) | 1.72  (0.55, 5.36) | 1.05  (0.27, 4.10) | 1.65  (0.43, 6.33) | 0.68  (0.33, 1.38) | 0.78  (0.36, 1.67) | 0.80  (0.48, 1.36) | **12step** | 1.16 (0.36,3.78) |
| 1.49  (0.35, 6.33) | 3.34  (0.81, 13.79) | 3.28  (0.75, 14.39) | **3.96**  **(1.18, 13.24)** | 2.58  (0.63, 10.55) | 2.89  (0.62, 13.44) | 1.76  (0.45, 6.84) | 2.77  (0.42, 18.17) | 1.14  (0.26, 4.95) | 1.30  (0.27, 6.37) | 1.35  (0.33, 5.57) | 1.68  (0.38, 7.46) | **12step+ NCR** |

Psychosocial treatment Abstinence at the end of treatment (OR [95% Cl]) Dropout due to any cause at the end of treatment (OR [95% Cl])

**Notes**. Psychosocial treatments are reported in alphabetical order. Comparisons should be read from left to right. The “abstinence at 12 weeks” and the “dropout due to any cause at 12 weeks “ estimates are located at the intersection of the column-defining treatment and the row-defining treatment. For abstinence, an OR above 1 favors the column-defining treatment. For dropout due to any cause, an OR above 1 favors the row-defining treatment. To obtain ORs for comparisons in the opposing direction, reciprocals should be taken. Significant results are in bold and underlined. CBT: cognitive behavioural therapy; CM: contingency management; CRA: community reinforcement approach; MBT: meditation based therapies; NCR: not contingent rewards; SEPT: supportive expressive psychodynamic therapy; TAU: treatment as usual; 12 step: twelve-step programme.
