# Supplementary material for: Comparative efficacy and acceptability of psychosocial interventions for individuals with cocaine and amphetamine addiction: A systematic review and network meta-analysis
Source: PLoS Med. 2018 Dec 26;15(12):e1002715. doi: 10.1371/journal.pmed.1002715 (PMC6306153; doi:10.1371/journal.pmed.1002715)
Supplement: S11 Fig — (DOCX) [file pmed.1002715.s012.docx]

**S11 Fig. Sensitivity Network Meta-Analysis for Abstinence and Dropout at the End of Treatment by Considering only the Trials on Individuals Addicted to Stimulants and on Opioid Substitution Therapy.**

| **CBT** | 1.19  (0.67, 2.12) | 1.85  (0.81, 4.22) | 1.28  (0.68, 2.41) | 1.32  (0.76, 2.28) | 2.11  (0.76, 5.82) |
| --- | --- | --- | --- | --- | --- |
| **0.54**  **(0.29, 0.99)** | **CM** | 1.55  (0.72, 3.34) | 1.08  (0.74, 1.56) | 1.10  (0.79, 1.55) | 1.77  (0.70, 4.43) |
| 0.53  (0.26, 1.08) | 0.99  (0.52, 1.87) | **CM + CBT** | 0.69  (0.32, 1.51) | 0.71  (0.32, 1.60) | 1.14  (0.35, 3.69) |
| 1.62  (0.80, 3.27) | **3.02**  **(1.93, 4.72)** | **3.06**  **(1.48, 6.29)** | **NCR** | 1.03  (0.63, 1.67) | 1.64  (0.61, 4.39) |
| 1.42  (0.80, 2.54) | **2.65**  **(1.67, 4.22)** | **2.69**  **(1.34, 5.40)** | 0.88  (0.48, 1.60) | **TAU** | 1.60  (0.68, 3.76) |
| 0.76  (0.27, 2.14) | 1.42  (0.53, 3.76) | 1.43  (0.47, 4.34) | 0.47  (0.16, 1.34) | 0.53  (0.23, 1.26) | **12 step** |

Psychosocial treatment Abstinence at the end of treatment (OR [95% Cl]) Dropout due to any cause at the end of treatment (OR [95% Cl])

**Notes**. Psychosocial treatments are reported in alphabetical order. Comparisons should be read from left to right. The “abstinence at the end of treatment” and the “dropout due to any cause at the end of treatment “ estimates are located at the intersection of the column-defining treatment and the row-defining treatment. For abstinence, an OR above 1 favors the column-defining treatment. For dropout due to any cause, an OR above 1 favors the row-defining treatment. To obtain ORs for comparisons in the opposing direction, reciprocals should be taken. Significant results are in bold and underlined. CBT: cognitive behavioural therapy; CM: contingency management; NCR: not contingent rewards; TAU: treatment as usual; 12 step: twelve-step programme
