# Supplementary material for: Comparative efficacy and acceptability of psychosocial interventions for individuals with cocaine and amphetamine addiction: A systematic review and network meta-analysis
Source: PLoS Med. 2018 Dec 26;15(12):e1002715. doi: 10.1371/journal.pmed.1002715 (PMC6306153; doi:10.1371/journal.pmed.1002715)

**S12a Fig. Study Limitations for Each Pairwise Estimate as the Risk of Bias Judgments from All Direct Estimates for Abstinence at the End of Treatment.**

The following figures were generated through the Confidence in Network Meta-Analysis Software (CINeMA) and present the risk of bias for each pairwise estimate. S12a Figure is for abstinence at the end of treatment, while S12b Figure is for dropout at the end of treatment. Low risk of bias is presented in green, unclear risk of bias in yellow and high risk of bias in red.


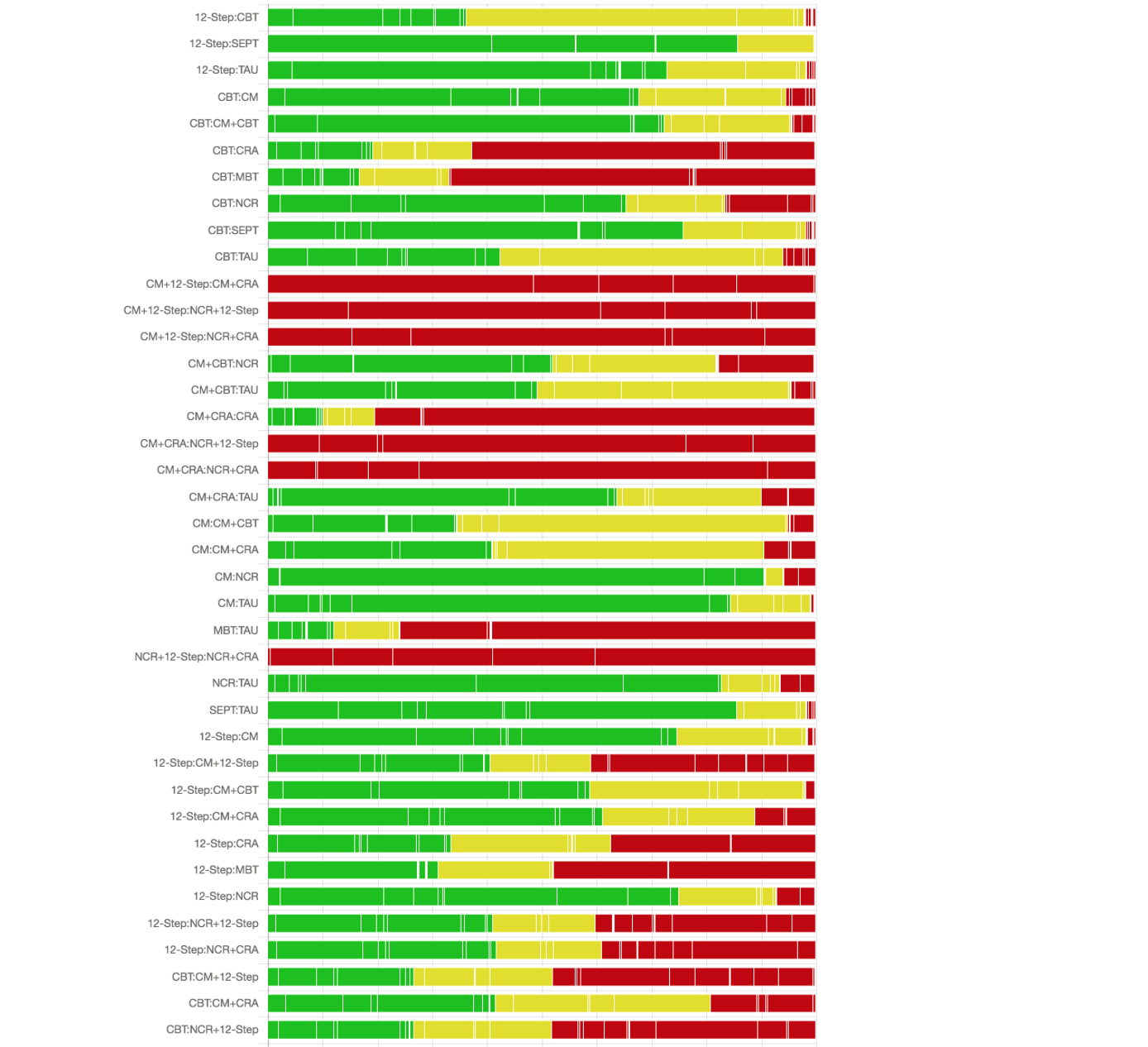


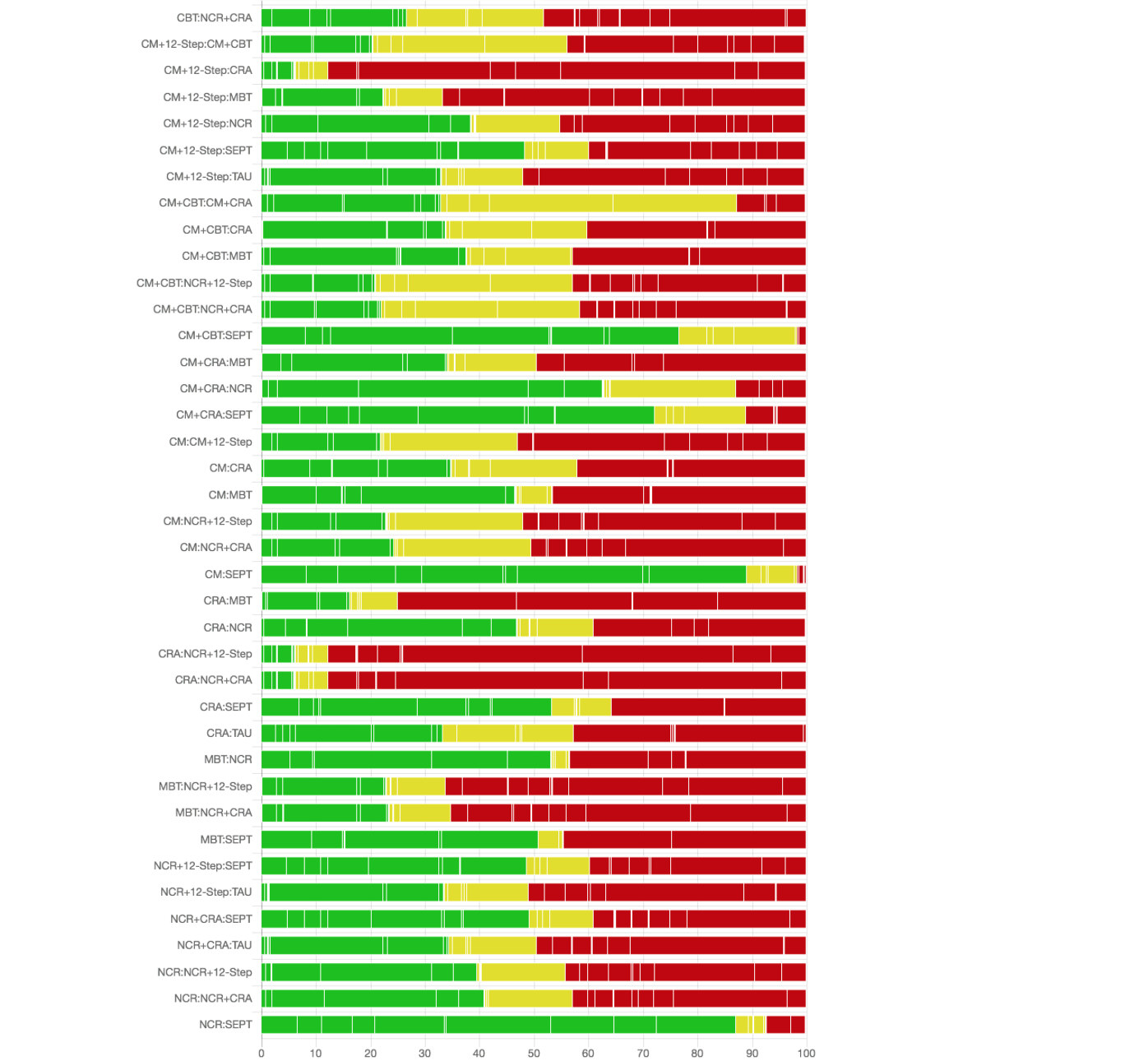


**S12b Fig. Study Limitations for Each Pairwise Estimate as the Risk of Bias Judgments from All Direct Estimates for Dropout at the End of Treatment.**


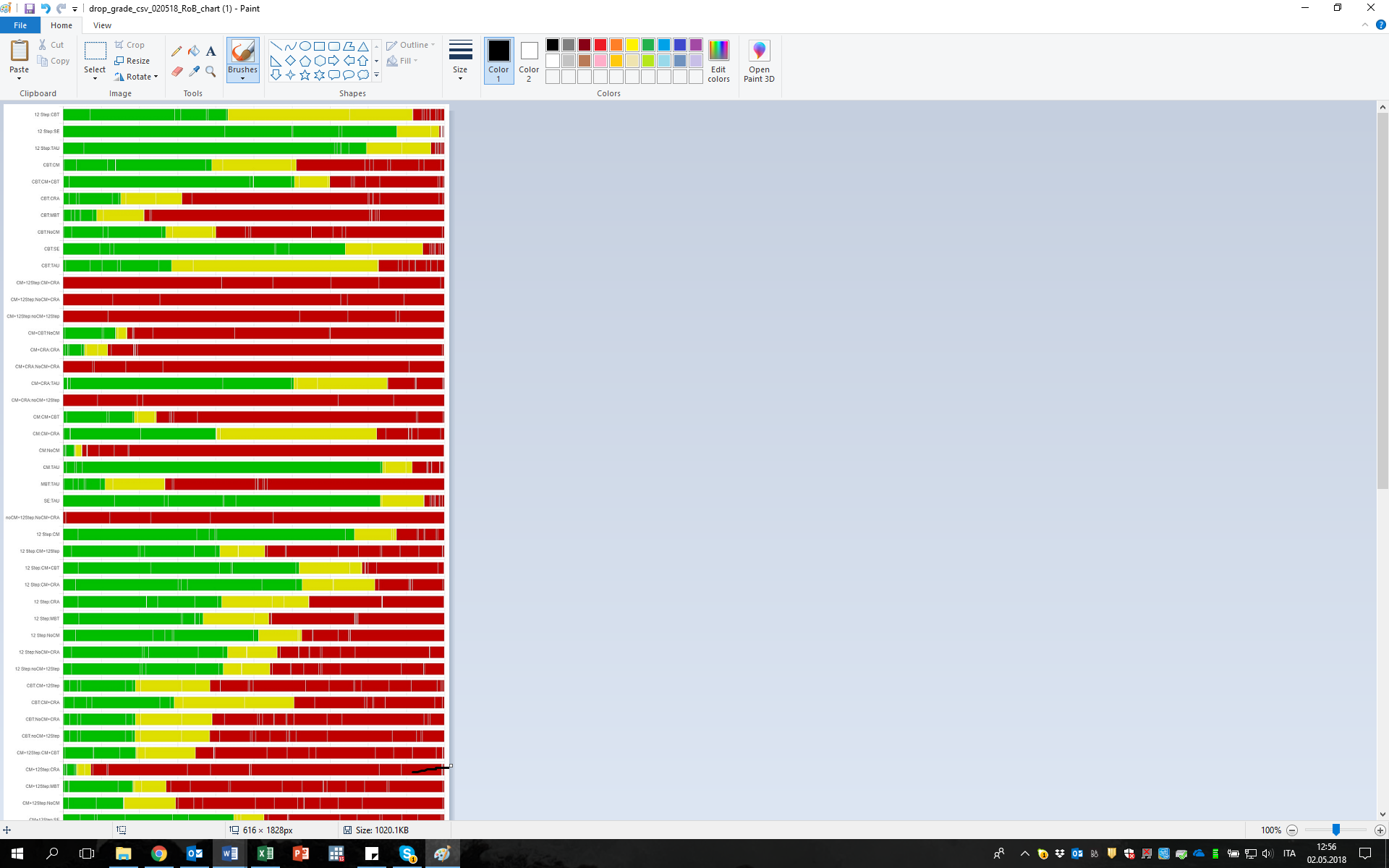


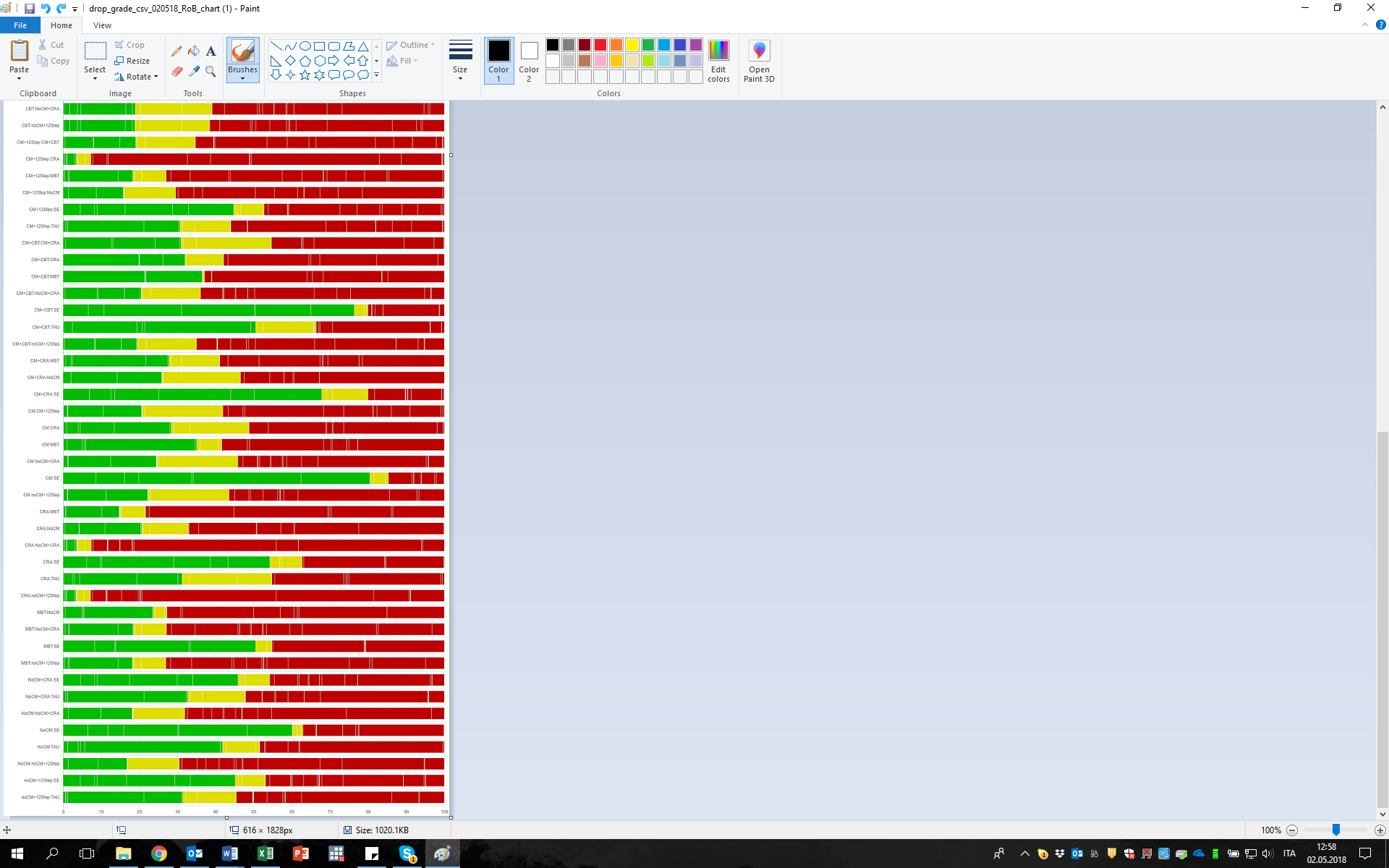

Supplement: S12 Fig — (DOCX) [file pmed.1002715.s013.docx]
