# Supplementary material for: Comparative efficacy and acceptability of psychosocial interventions for individuals with cocaine and amphetamine addiction: A systematic review and network meta-analysis
Source: PLoS Med. 2018 Dec 26;15(12):e1002715. doi: 10.1371/journal.pmed.1002715 (PMC6306153; doi:10.1371/journal.pmed.1002715)
Supplement: S13 Fig — (DOCX) [file pmed.1002715.s014.docx]

**S13a Figure. Networkplot by risk of bias for abstinence at the end of treatment.**

The following figures were generated through the Confidence in Network Meta-Analysis Software (CINeMA)^.^ The figures plot the network of eligible direct comparisons for abstinence at the end of treatment (A, 46 trials) and dropout due to any cause at the end of treatment (B, 43 studies). The width of the lines is proportional to the number of trials comparing every pair of treatments. The colour of each line represents the majority of risk of bias for each comparison. The prevalence of low/unclear/high risk of bias for each intervention is represented by pie charts. Low risk of bias is in green, unclear risk of bias in yellow and high risk of bias in red.

**
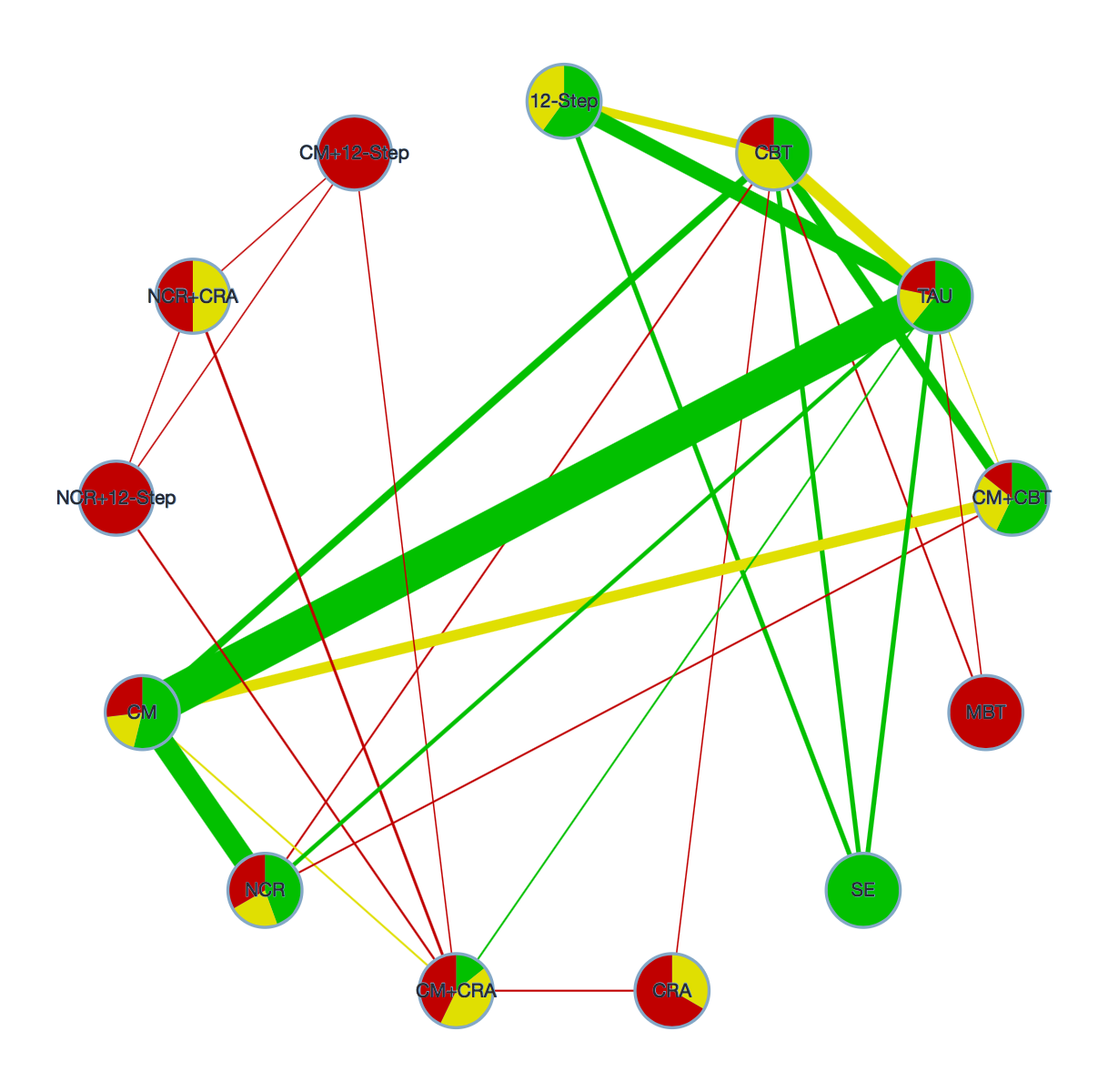
**

**S13b Figure. Networkplot by risk of bias for dropout at the end of treatment.**

**
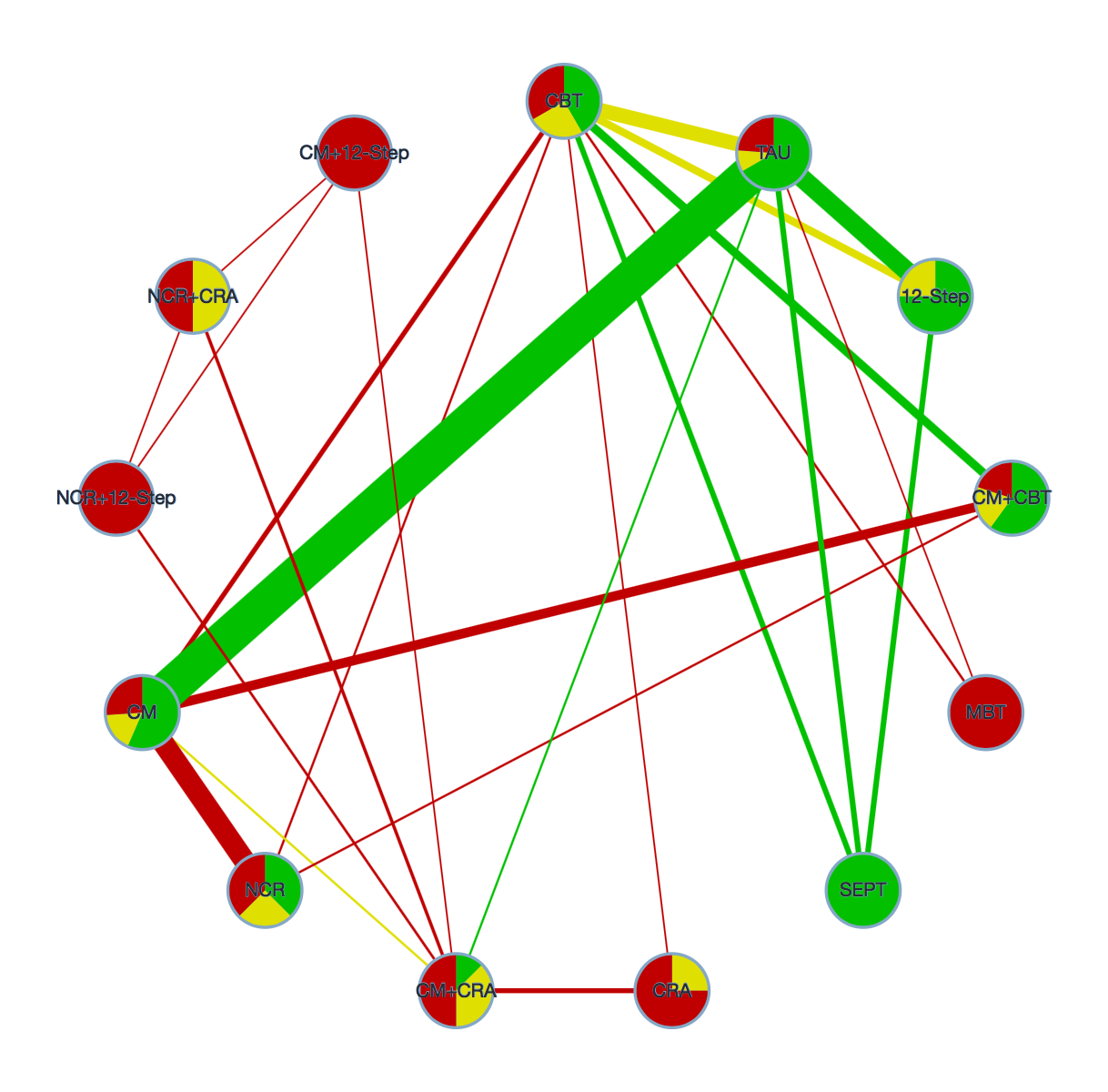
**
