# Supplementary material for: Comparative efficacy and acceptability of psychosocial interventions for individuals with cocaine and amphetamine addiction: A systematic review and network meta-analysis
Source: PLoS Med. 2018 Dec 26;15(12):e1002715. doi: 10.1371/journal.pmed.1002715 (PMC6306153; doi:10.1371/journal.pmed.1002715)
Supplement: S1 Table — (DOCX) [file pmed.1002715.s016.docx]

**S1 Table. Dropout rates based on each intervention.**

| **Intervention** | **Total sample (n)** | **Dropouts (n)** | **Dropout rate (%)** |
| --- | --- | --- | --- |
| CBT | 609 | 200 | 32.8 |
| CM | 1599 | 502 | 31.4 |
| CM + 12-Step | 37 | 10 | 27 |
| CM + CBT | 285 | 43 | 15.1 |
| CM + CRA | 233 | 48 | 20.6 |
| CRA | 49 | 17 | 34.7 |
| CRA + NCR | 69 | 14 | 20.3 |
| MBT | 88 | 53 | 60.2 |
| NCR | 457 | 102 | 22.3 |
| SEPT | 124 | 55 | 44.4 |
| TAU | 1478 | 594 | 40.2 |
| 12-Step | 461 | 151 | 32.7 |
| 12-Step + NCR | 56 | 29 | 51.8 |

Abbreviations: CBT, cognitive behavioral therapy; CM, contingency management; CRA, community reinforcement approach; MBT, meditation based treatments; n: number; NCR, non-contingent rewards; SEPT, supportive-expressive psychodynamic therapy; TAU, treatment as usual; 12-step, twelve-step program.
