# Supplementary material for: Comparative efficacy and acceptability of psychosocial interventions for individuals with cocaine and amphetamine addiction: A systematic review and network meta-analysis
Source: PLoS Med. 2018 Dec 26;15(12):e1002715. doi: 10.1371/journal.pmed.1002715 (PMC6306153; doi:10.1371/journal.pmed.1002715)
Supplement: S3 Table — (DOCX) [file pmed.1002715.s018.docx]

**S3 Table. Risk of Bias Summary.**

**These are review authors' judgments about each risk of bias item for each included study.**

| **Study** | **Random sequence generation**  **(selection bias)** | **Allocation concealment (selection bias)** | **Blinding of participants and personnel (performance bias)** | **Blinding of outcome assessment**  **(detection bias)** | **Incomplete outcome data**  **(attrition bias)** | **Selective reporting (reporting bias)** |
| --- | --- | --- | --- | --- | --- | --- |
| Carroll, 1994^1^ | 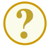 | 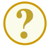 | 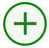 | 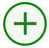 | **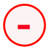** | 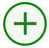 |
| Carroll, 1998^2^ | 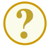 | 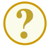 | 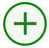 | 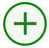 | 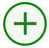 | 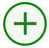 |
| Carroll, 2012^3^ | 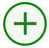 | 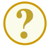 | 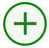 | 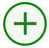 | 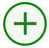 | 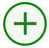 |
| Carroll, 2014^4^ | 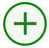 | 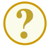 | 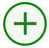 | 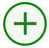 | 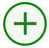 | 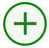 |
| Carroll, 2016^5^ | 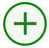 | 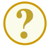 | 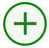 | 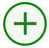 | 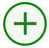 | 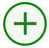 |
| Chen, 2013^6^ | 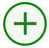 | 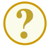 | 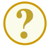 | 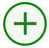 | **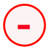** | 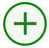 |
| Crits-Cristoph, 1999^7^ | 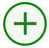 | 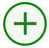 | 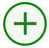 | 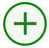 | 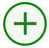 | 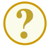 |
| Donovan, 2013^8^ | 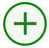 | 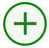 | 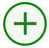 | 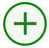 | 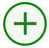 | 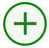 |
| Dursteler-MacFarland, 2013^9^ | 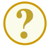 | 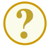 | 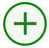 | 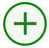 | 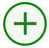 | 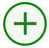 |
| Epstein, 2003^10^ | 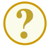 | 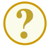 | 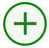 | 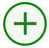 | **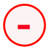** | 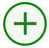 |
| Festinger, 2014^11^ | 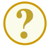 | 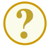 | 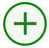 | 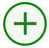 | 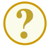 | 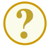 |
| Garcia-Fernandez, 2011^12^ | 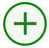 | 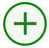 | 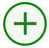 | 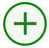 | **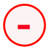** | 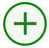 |
| Garcia-Rodriguez, 2009^13^ | 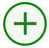 | 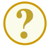 | 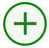 | 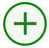 | 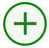 | 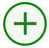 |
| Ghitza, 2007^14^ | 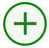 | 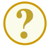 | 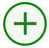 | 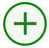 | 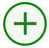 | 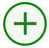 |
| Hagedorn, 2013^15^ | 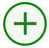 | 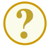 | 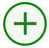 | 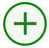 | **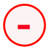** | 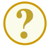 |
| Higgins, 1993^16^ | 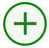 | 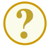 | 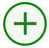 | 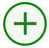 | 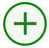 | **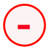** |
| Higgins, 1994^17^ | 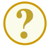 | 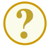 | 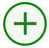 | 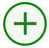 |  |  |
| Higgins, 2000^18^ |  |  |  |  |  |  |
| Higgins, 2003^19^ |  |  |  |  |  |  |
| Kirby, 1998^20^ |  |  |  |  |  |  |
| Landovitz, 2014^21^ |  |  |  |  |  |  |
| Ledgerwood, 2006^22^ |  |  |  |  |  |  |
| Maude-Griffin, 1998^23^ |  |  |  |  |  |  |
| McDonnell, 2013^24^ |  |  |  |  |  |  |
| McKay, 1997^25^ |  |  |  |  |  |  |
| Menza, 2010^26^ |  |  |  |  |  |  |
| Miguel, 2016^27^ |  |  |  |  |  |  |
| Milby, 2008^28^ |  |  |  |  |  |  |
| Peirce, 2006^29^ |  |  |  |  |  |  |
| Petitjean, 2014^30^ |  |  |  |  |  |  |
| Petry, 2002^31^ |  |  |  |  |  |  |
| Petry, 2005^32^ |  |  |  |  |  |  |
| Petry, 2005^33^ |  |  |  |  |  |  |
| Petry, 2007^34^ |  |  |  |  |  |  |
| Petry, 2012^35^ |  |  |  |  |  |  |
| Petry, 2012^36^ |  |  |  |  |  |  |
| Petry, 2013^37^ |  |  |  |  |  |  |
| Poling, 2006^38^ |  |  |  |  |  |  |
| Rawson, 2002^39^ |  |  |  |  |  |  |
| Rawson, 2006^40^ |  |  |  |  |  |  |
| Roll, 2013^41^ |  |  |  |  |  |  |
| Sanchez-Hervás, 2010^42^ |  |  |  |  |  |  |
| Schottenfeld, 2011^43^ |  |  |  |  |  |  |
| Secades-Villa, 2013^44^ |  |  |  |  |  |  |
| Shoptaw, 2005^45^ |  |  |  |  |  |  |
| Shoptaw, 2008^46^ |  |  |  |  |  |  |
| Silverman, 1996^47^ |  |  |  |  |  |  |
| Silverman, 1998^48^ |  |  |  |  |  |  |
| Smout, 2010^49^ |  |  |  |  |  |  |
| Umbricht, 2014^50^ |  |  |  |  |  |  |

Abbreviations: = Low risk of bias; = Unclear risk of bias;  = High risk of bias.

**References**

1. Carroll KM, Rounsaville BJ, Nich C, Gordon LT, Wirtz PW, Gawin F. One-year follow-up of psychotherapy and pharmacotherapy for cocaine dependence. Delayed emergence of psychotherapy effects. Arch Gen Psychiatry 1994;51(12):989-997. doi: 10.1001/archpsyc.1994.03950120061010 pmid: 7979888
2. Carroll KM, Nich C, Ball SA, McCance E, Rounsavile BJ. Treatment of cocaine and alcohol dependence with psychotherapy and disulfiram. Addiction 1998;93(5):713-727. doi: 10.1046/j.1360-0443.1998.9357137.x pmid: 9692270
3. Carroll KM, Nich C, Shi JM, Eagan D, Ball SA. Efficacy of disulfiram and Twelve Step Facilitation in cocaine-dependent individuals maintained on methadone: a randomized placebo-controlled trial. Drug Alcohol Depend 2012;126(1-2):224-231. doi: 10.1016/j.drugalcdep.2012.05.019 pmid: 22695473
4. Carroll KM, Kiluk BD, Nich C, et al. Computer-assisted delivery of cognitive-behavioral therapy: efficacy and durability of CBT4CBT among cocaine-dependent individuals maintained on methadone. Am J Psychiatry 2014;171(4):436-444. doi: 10.1176/appi.ajp.2013.13070987 pmid: 24577287
5. Carroll KM, Nich C, Petry NM, Eagan DA, Shi JM, Ball SA. A randomized factorial trial of disulfiram and contingency management to enhance cognitive behavioral therapy for cocaine dependence. Drug Alcohol Depend 2016;160:135-142. doi: 10.1016/j.drugalcdep.2015.12.036 pmid: 26817621
6. Chen KW, Berger CC, Gandhi D, Weintraub E, Lejuez CW. Adding integrative meditation with ear acupressure to outpatient treatment of cocaine addiction: a randomized controlled pilot study. J Altern Complement Med 2013;19(3):204-210. doi: 10.1089/acm.2011.0311 pmid: 23062020
7. Crits-Christoph P, Siqueland L, Blaine J, et al. Psychosocial treatments for cocaine dependence: National Institute on Drug Abuse Collaborative Cocaine Treatment Study. Arch Gen Psychiatry 1999;56(6):493-502. doi: 10.1001/archpsyc.56.6.493 pmid: 10359461
8. Donovan DM, Daley DC, Brigham GS, et al. Stimulant abuser groups to engage in 12-step: a multisite trial in the National Institute on Drug Abuse Clinical Trials Network. J Subst Abuse Treat 2013;44(1):103-114. doi: 10.1016/j.jsat.2012.04.004 pmid: 22657748
9. Dursteler-MacFarland KM, Farronato NS, Strasser J, et al. A randomized, controlled, pilot trial of methylphenidate and cognitive-behavioral group therapy for cocaine dependence in heroin prescription. J Clin Psychopharmacol 2013;33(1):104-108. doi: 10.1097/JCP.0b013e31827bfff4 pmid: 23277248
10. Epstein DH, Hawkins WE, Covi L, Umbricht A, Preston KL. Cognitive-behavioral therapy plus contingency management for cocaine use: findings during treatment and across 12-month follow-up. Psychol Addict Behav 2003;17(1):73-82. doi: 10.1037/0893-164X.17.1.73 pmid:12665084
11. Festinger DS, Dugosh KL, Kirby KC, Seymour BL. Contingency management for cocaine treatment: cash vs. vouchers. J Subst Abuse Treat 2014;47(2):168-174. doi: 10.1016/j.jsat.2014.03.001 pmid: 24746956
12. Garcia-Fernandez G, Secades-Villa R, Garcia-Rodriguez O, Sanchez-Hervas E, Fernandez-Hermida JR, Higgins ST. Adding voucher-based incentives to community reinforcement approach improves outcomes during treatment for cocaine dependence. Am J Addict 2011;20(5):456-461. doi: 10.1111/j.1521-0391.2011.00154.x pmid: 21838845
13. Garcia-Rodriguez O, Secades-Villa R, Alvarez Rodriguez H, et al. Effect of incentives on retention in an outpatient treatment for cocaine addicts. Psicothema. 2007;19(1):134-139. pmid: 17295995
14. Ghitza UE, Epstein DH, Schmittner J, Vahabzadeh M, Lin JL, Preston KL. Randomized trial of prize-based reinforcement density for simultaneous abstinence from cocaine and heroin. J Consult Clin Psychol 2007;75(5):765-774. doi: 10.1037/0022-006X.75.5.765 pmid: 17907858
15. Hagedorn HJ, Noorbaloochi S, Simon AB, et al. Rewarding early abstinence in Veterans Health Administration addiction clinics. J Subst Abuse Treat 2013;45(1):109-117. doi: 10.1016/j.jsat.2013.01.006 pmid: 23453480
16. Higgins ST, Budney AJ, Bickel WK, Hughes JR, Foerg F, Badger G. Achieving cocaine abstinence with a behavioral approach. Am J Psychiatry 1993;150(5):763-769. doi: 10.1176/ajp.150.5.763 pmid: 8480823
17. Higgins ST, Budney AJ, Bickel WK, Foerg FE, Donham R, Badger GJ. Incentives improve outcome in outpatient behavioral treatment of cocaine dependence. Arch Gen Psychiatry 1994;51(7):568-576. doi: 10.1001/archpsyc.1994.03950070060011 pmid: 8031230
18. Higgins ST, Wong CJ, Badger GJ, Ogden DE, Dantona RL. Contingent reinforcement increases cocaine abstinence during outpatient treatment and 1 year of follow-up. J Consult Clin Psychol 2000;68(1):64-72. doi: 10.1037/0022-006X.68.1.64 pmid: 10710841
19. Higgins ST, Sigmon SC, Wong CJ, et al. Community reinforcement therapy for cocaine-dependent outpatients. Arch Gen Psychiatry 2003;60(10):1043-1052. doi: 10.1001/archpsyc.60.9.1043 pmid: 14557150
20. Kirby KC, Marlowe DB, Festinger DS, Lamb RJ, Platt JJ. Schedule of voucher delivery influences initiation of cocaine abstinence. J Consult Clin Psychol 1998;66(5):761-767. doi: 10.1037/0022-006X.66.5.761 pmid: 9803694
21. Landovitz RJ, Fletcher JB, Shoptaw S, Reback CJ. Contingency management facilitates the use of postexposure prophylaxis among stimulant-using men who have sex with men. Open Forum Infect Dis 2015;2(1):ofu114. doi: 10.1093/ofid/ofu114 doi: 10.1093/ofid/ofu114 pmid: 25884003
22. Ledgerwood DM, Petry NM. Does contingency management affect motivation to change substance use?. Drug Alcohol Depend 2006;83(1):65-72. doi: 10.1016/j.drugalcdep.2005.10.012 pmid: 16310974
23. Maude-Griffin PM, Hohenstein JM, Humfleet GL, Reilly PM, Tusel DJ, Hall SM. Superior efficacy of cognitive-behavioral therapy for urban crack cocaine abusers: main and matching effects. J Consult Clin Psychol 1998;66(5):832-837. doi: 10.1037/0022-006X.66.5.832 pmid: 9803702
24. McDonell MG, Srebnik D, Angelo F, et al. Randomized controlled trial of contingency management for stimulant use in community mental health patients with serious mental illness. Am J Psychiatry 2013;170(1):94-101. doi: 10.1176/appi.ajp.2012.11121831 pmid: 23138961
25. McKay JR, Alterman AI, Cacciola JS, Rutherford MJ, O'Brien CP, Koppenhaver J. Group counseling versus individualized relapse prevention aftercare following intensive outpatient treatment for cocaine dependence: initial results. J Consult Clin Psychol 1997;65(5):778-788. doi: 10.1037/0022-006X.65.5.778 pmid: 9337497
26. Menza TW, Jameson DR, Hughes JP, Colfax GN, Shoptaw S, Golden MR. Contingency management to reduce methamphetamine use and sexual risk among men who have sex with men: a randomized controlled trial. BMC Public Health 2010;10:774-2458-10-774. doi: 10.1186/1471-2458-10-774 pmid: 21172026
27. Miguel AQ, Madruga CS, Cogo-Moreira H, et al. Contingency management is effective in promoting abstinence and retention in treatment among crack cocaine users in Brazil: A randomized controlled trial. Psychol Addict Behav 2016;30(5):536-543. doi:10.1037/adb0000192 pmid: 27442691
28. Milby JB, Schumacher JE, Vuchinich RE, Freedman MJ, Kertesz S, Wallace D. Toward cost-effective initial care for substance-abusing homeless. J Subst Abuse Treat 2008;34(2):180-191. doi: 10.1016/j.jsat.2007.03.003 pmid: 17512156
29. Peirce JM, Petry NM, Stitzer ML, et al. Effects of lower-cost incentives on stimulant abstinence in methadone maintenance treatment: a National Drug Abuse Treatment Clinical Trials Network study. Arch Gen Psychiatry 2006;63(2):201-208. doi: 10.1001/archpsyc.63.2.201 pmid: 16461864
30. Petitjean SA, Dursteler-MacFarland KM, Krokar MC, et al. A randomized, controlled trial of combined cognitive-behavioral therapy plus prize-based contingency management for cocaine dependence. Drug Alcohol Depend 2014;145:94-100. doi: 10.1016/j.drugalcdep.2014.09.785 pmid: 25456571
31. Petry NM, Martin B. Low-cost contingency management for treating cocaine- and opioid-abusing methadone patients. J Consult Clin Psychol 2002;70(2):398-405. doi: 10.1037/0022-006X.70.2.398 pmid: 11952198
32. Petry NM, Peirce JM, Stitzer ML, et al. Effect of prize-based incentives on outcomes in stimulant abusers in outpatient psychosocial treatment programs: a national drug abuse treatment clinical trials network study. Arch Gen Psychiatry 2005;62(10):1148-1156. doi: 10.1001/archpsyc.62.10.1148 pmid: 16203960
33. Petry NM, Martin B, Simcic F,Jr. Prize reinforcement contingency management for cocaine dependence: integration with group therapy in a methadone clinic. J Consult Clin Psychol 2005;73(2):354-359. doi: 10.1037/0022-006X.73.2.354 pmid: 15796645
34. Petry NM, Alessi SM, Hanson T, Sierra S. Randomized trial of contingent prizes versus vouchers in cocaine-using methadone patients. J Consult Clin Psychol 2007;75(6):983-991. doi: 10.1037/0022-006X.75.6.983 pmid: 18085914
35. Petry NM, Alessi SM, Ledgerwood DM. A randomized trial of contingency management delivered by community therapists. J Consult Clin Psychol 2012;80(2):286-298. doi: 10.1037/a0026826 pmid: 22250852
36. Petry NM, Barry D, Alessi SM, Rounsaville BJ, Carroll KM. A randomized trial adapting contingency management targets based on initial abstinence status of cocaine-dependent patients. J Consult Clin Psychol 2012;80(2):276-285. doi: 10.1037/a0026883 pmid: 22229758
37. Petry NM, Alessi SM, Rash CJ. A randomized study of contingency management in cocaine-dependent patients with severe and persistent mental health disorders. Drug Alcohol Depend 2013;130(1-3):234-237. doi: 10.1016/j.drugalcdep.2012.10.017 pmid: 23182410
38. Poling J, Oliveto A, Petry N, et al. Six-month trial of bupropion with contingency management for cocaine dependence in a methadone-maintained population. Arch Gen Psychiatry 2006;63(2):219-228. doi: 10.1001/archpsyc.63.2.219 pmid: 16461866
39. Rawson RA, Huber A, McCann M, et al. A comparison of contingency management and cognitive-behavioral approaches during methadone maintenance treatment for cocaine dependence. Arch Gen Psychiatry 2002;59(9):817-824. doi: 10.1001/archpsyc.59.9.817 pmid: 12215081
40. Rawson RA, McCann MJ, Flammino F, et al. A comparison of contingency management and cognitive-behavioral approaches for stimulant-dependent individuals. Addiction 2006;101(2):267-74. doi: 10.1111/j.1360-0443.2006.01312.x pmid: 16445555
41. Roll JM, Chudzynski J, Cameron JM, Howell DN, McPherson S. Duration effects in contingency management treatment of methamphetamine disorders. Addict Behav 2013;38(9):2455-2462. doi: 10.1016/j.addbeh.2013.03.018 pmid: 23708468
42. Secades-Villa R, Sanchez-Hervas E, Zacares-Romaguera F, Garcia-Rodriguez O, Santonja-Gomez FJ, Garcia-Fernandez G. Community Reinforcement Approach (CRA) for cocaine dependence in the Spanish public health system: 1 year outcome. Drug Alcohol Rev 2011;30(6):606-612. doi: 10.1111/j.1465-3362.2010.00250.x pmid: 21355914
43. Schottenfeld RS, Moore B, Pantalon MV. Contingency management with community reinforcement approach or twelve-step facilitation drug counseling for cocaine dependent pregnant women or women with young children. Drug Alcohol Depend 2011;118(1):48-55. doi: 10.1016/j.drugalcdep.2011.02.019 pmid: 21454024
44. Secades-Villa R, Garcia-Fernandez G, Pena-Suarez E, Garcia-Rodriguez O, Sanchez-Hervas E, Fernandez-Hermida JR. Contingency management is effective across cocaine-dependent outpatients with different socioeconomic status. J Subst Abuse Treat 2013;44(3):349-354. doi: 10.1016/j.jsat.2012.08.018 pmid: 22999380
45. Shoptaw S, Reback CJ, Peck JA, et al. Behavioral treatment approaches for methamphetamine dependence and HIV-related sexual risk behaviors among urban gay and bisexual men. Drug Alcohol Depend 2005;78(2):125-134. doi: 10.1016/j.drugalcdep.2004.10.004 pmid: 15845315
46. Shoptaw S, Reback CJ, Larkins S, et al. Outcomes using two tailored behavioral treatments for substance abuse in urban gay and bisexual men. J Subst Abuse Treat 2008;35(3):285-293. doi: 10.1016/j.jsat.2007.11.004 pmid:18329226
47. Silverman K, Higgins ST, Brooner RK, et al. Sustained cocaine abstinence in methadone maintenance patients through voucher-based reinforcement therapy. Arch Gen Psychiatry 1996;53(5):409-415. doi: 10.1001/archpsyc.1996.01830050045007 pmid: 8624184
48. Silverman K, Wong CJ, Umbricht-Schneiter A, Montoya ID, Schuster CR, Preston KL. Broad beneficial effects of cocaine abstinence reinforcement among methadone patients. J Consult Clin Psychol 1998;66(5):811-824. doi: 10.1037/0022-006X.66.5.811pmid: 9803700
49. Smout MF, Longo M, Harrison S, Minniti R, Wickes W, White JM. Psychosocial treatment for methamphetamine use disorders: a preliminary randomized controlled trial of cognitive behavior therapy and Acceptance and Commitment Therapy. Subst Abus 2010;31(2):98-107. doi:10.1080/08897071003641578 pmid: 20408061
50. Umbricht A, DeFulio A, Winstanley EL, et al. Topiramate for cocaine dependence during methadone maintenance treatment: a randomized controlled trial. Drug Alcohol Depend 2014;140:92-100. doi: 10.1016/j.drugalcdep.2014.03.033 pmid: 24814607.
