# Supplementary material for: Comparative efficacy and acceptability of psychosocial interventions for individuals with cocaine and amphetamine addiction: A systematic review and network meta-analysis
Source: PLoS Med. 2018 Dec 26;15(12):e1002715. doi: 10.1371/journal.pmed.1002715 (PMC6306153; doi:10.1371/journal.pmed.1002715)
Supplement: S4 Table — (DOCX) [file pmed.1002715.s019.docx]

**S4 Table. Summary Numbers of Studies and Patients from Pairwise Meta-analysis of Direct Comparisons.**

|  | | **Abstinence**  **at 12 weeks**  **(N/n)^a^** | | **Abstinence at the end of treatment**  **(N/n)** | **Abstinence at Follow-Up**  **(N/n)** | | **Dropout**  **at 12 weeks**  **(N/n)** | | **Dropout at the end of treatment**  **(N/n)** | | **Longest duration of abstinence**  **at 12 weeks**  **(N/n)** | **Longest duration of abstinence at the end of treatment**  **(N/n)** |
| --- | --- | --- | --- | --- | --- | --- | --- | --- | --- | --- | --- | --- |
| **CBT vs.** |  | |  | |  |  | |  | |  | |  |
| CM | 4/395 | | 4/395 | | 4/395 | 2/213 | | 2/213 | | 2/217 | | 2/217 |
| NCR | 1/97 | | 1/97 | | 1/97 | 1/97 | | 1/97 | | 1/97 | | 1/97 |
| TAU | 6/691 | | 6/691 | | 3/430 | 5/643 | | 5/643 | | 2/211 | | 2/211 |
| **CM vs.** |  | |  | |  |  | |  | |  | |  |
| NCR | 9/1156 | | 9/1137 | | 7/879 | 8/931 | | 8/931 | | 5/588 | | 6/675 |
| TAU | 14/1984 | | 14/1984 | | 9/1265 | 12/1686 | | 12/1686 | | 11/ 1792 | | 11/ 1792 |
| **CM+CBT vs.** |  | |  | |  |  | |  | |  | |  |
| CBT | 6/553 | | 6/553 | | 5/454 | 4/373 | | 4/373 | | 2/217 | | 2/277 |
| CM | 5/563 | | 5/561 | | 5/563 | 3/421 | | 3/421 | | 2/178 | | 3/384 |
| NCR | 1/98 | | 1/98 | | 1/98 | 1/98 | | 1/98 | | 1/98 | | 1/98 |
| TAU | 1/60 | | 1/60 | | 1/60 | NA | | NA | | NA | | NA |
| **CM+CRA vs.** |  | |  | |  |  | |  | |  | |  |
| CM | 1/100 | | 1/100 | | 1/100 | 1/100 | | 1/100 | | NA | | NA |
| CRA | 1/58 | | 2/98 | | 2/98 | 2/98 | | 3/216 | | 1/40 | | 2/158 |
| TAU | NA | | 1/96 | | NA | 1/96 | | 1/96 | | NA | | 1/96 |
| **CM+12 step vs.** |  | |  | |  |  | |  | |  | |  |
| CM+CRA | 1/73 | | 1/73 | | 1/73 | 1/73 | | 1/73 | | NA | | 1/73 |
| CRA+NCR | 1/72 | | 1/72 | | 1/72 | 1/72 | | 1/72 | | NA | | 1/72 |
| **CRA vs.** |  | |  | |  |  | |  | |  | |  |
| CBT | NA | | 1/74 | | 1/74 | NA | | 1/74 | | NA | | NA |
| **CRA+NCR vs.** |  | |  | |  |  | |  | |  | |  |
| CM+CRA | 1/71 | | 2/141 | | 2/141 | 2/141 | | 2/141 | | NA | | 1/71 |
| **MBT vs.** |  | |  | |  |  | |  | |  | |  |
| CBT | 1/104 | | 1/104 | | 1/104 | 1/104 | | 1/104 | | NA | | NA |
| TAU | 1/72 | | 1/72 | | NA | 1/72 | | 1/72 | | NA | | NA |
| **NCR vs.** |  | |  | |  |  | |  | |  | |  |
| TAU | 1/215 | | 1/215 | | 1/215 | NA | | NA | | 1/215 | | 1/215 |
| **SEPT vs.** |  | |  | |  |  | |  | |  | |  |
| CBT | 1/243 | | 1/243 | | 1/243 | 1/243 | | 1/243 | | NA | | NA |
| TAU | 1/247 | | 1/247 | | 1/247 | 1/247 | | 1/247 | | NA | | NA |
| 12 step | 1/245 | | 1/245 | | 1/245 | 1/245 | | 1/245 | | NA | | NA |
| **12 step vs.** |  | |  | |  |  | |  | |  | |  |
| CBT | 3/463 | | 3/463 | | 3/463 | 2/335 | | 2/335 | | 1/95 | | 1/95 |
| TAU | 3/827 | | 3/827 | | 2/715 | 3/827 | | 3/827 | | NA | | NA |
| **12 step+NCR vs.** |  | |  | |  |  | |  | |  | |  |
| CM+CRA | 2/111 | | 2/111 | | 2/111 | 2/111 | | 2/111 | | 1/38 | | 2/111 |
| CM+12 step | 1/74 | | 1/74 | | 1/74 | 1/74 | | 1/74 | | NA | | 1/74 |
| CRA+NCR | 1/72 | | 1/72 | | 1/72 | 1/72 | | 1/72 | | NA | | 1/72 |

**^a^** N= number of studies; n= number of patients; NA= not available.

|  | **Abstinence**  **at 12 weeks**  **OR (95% CI)** | **Abstinence**  **at the end of treatment**  **OR (95% CI)** | **Abstinence at Follow-Up**  **OR (95% CI)** | **Dropout**  **at 12 weeks**  **OR (95% CI)** | **Dropout at the end of treatment**  **OR (95% CI)** | **Longest duration of abstinence**  **at 12 weeks**  **SMD (95% CI)** | **Longest duration of abstinence at the end of treatment**  **SMD (95% CI)** |
| --- | --- | --- | --- | --- | --- | --- | --- |
| **CBT vs.** |  |  |  |  |  |  |  |
| CM | **0.43 (0.27,0.68)** | **0.43 (0.27,0.68)** | 0.93 (0.60,1.43) | 0.91 (0.47,1.74) | 0.91 (0.47,1.74) | **-0.65 (-0.96,-0.34)** | **-0.65 (-0.96,-0.34)** |
| NCR | 1.22 (0.37,3.94) | 1.22 (0.37,3.94) | 1.34 (0.48,3.77) | 0.81 (0.31,2.10) | 0.81 (0.31,2.10) | 0.14 (-0.24,0.54) | 0.14 (-0.24,0.54) |
| TAU | 1.03 (0.50,2.12) | 1.03 (0.50,2.12) | 1.31 (0.58,2.97) | **0.69 (0.50,0.94)** | **0.66 (0.47,0.92)** | 0.08 (-0.28,0.45) | 0.08 (-0.28,0.45) |
| **CM vs.** |  |  |  |  |  |  |  |
| NCR | **2.65 (1.58,4.43)** | **2.69 (1.61,4.51)** | **2.08 (1.22,3.54)** | 1.32 (0.84,2.07) | 1.32 (0.84,2.07) | **0.61 (0.17,1.05)** | **0.55 (0.19,0.90)** |
| TAU | **2.28 (1.50,3.45)** | **2.28 (1.50,3.45)** | 1.07 (0.79,1.44) | **0.65 (0.49,0.87)** | **0.65 (0.49,0.87)** | **0.56 (0.41,0.71)** | **0.56 (0.41,0.71)** |
| **CM+CBT vs.** |  |  |  |  |  |  |  |
| CBT | **2.32 (1.57,3.41)** | **2.00 (1.22,3.26)** | 1.05 (0.61,1.82) | 1.13 (0.66,1.94) | 1.21 (0.71,2.07) | **0.71 (0.29,1.12)** | **0.63 (0.31,0.94)** |
| CM | 1.14 (0.79,1.66) | 1.20 (0.84,1.71) | 1.08 (0.75,1.56) | 0.87 (0.37,2.02) | 0.87 (0.37,2.02) | 0.04 (-0.68,0.76) | 0.12 (-0.28,0.52) |
| NCR | **3.16 (1.11,9.01)** | **3.16 (1.11,9.01)** | 1.66 (0.61,4.51) | 1.36 (0.55,3.31) | 1.36 (0.55,3.31) | **0.64 (0.24,1.04)** | **0.64 (0.24,1.04)** |
| TAU | **3.28 (1.08,9.95)** | **3.28 (1.08, 9.95)** | 1.64 (0.52,5.12) | NA | NA | NA | NA |
| **CM+CRA vs.** |  |  |  |  |  |  |  |
| CM | **3.32 (1.39,7.90)** | 1.37 (0.62,3.03) | **2.62 (1.09,6.25)** | **0.20 (0.08,0.51)** | **0.26 (0.11,0.60)** | NA | NA |
| CRA | **4.29 (1.42,12.99)** | 1.60 (0.50,5.15) | 1.20 (0.51,2.81) | **0.37 (0.14,0.99)** | 0.68 (0.22,2.07) | **0.72 (0.07,1.36)** | **0.82 (0.49,1.15)** |
| TAU | NA | 2.13 (0.86,5.26) | NA | 0.42 (0.17,1.00) | **0.32 (0.14,0.75)** | NA | **0.57 (0.16,0.98)** |
| **CM+12 step vs.** |  |  |  |  |  |  |  |
| CM+CRA | 0.84 (0.30,2.32) | 0.81 (0.26,2.54) | 0.81 (0.26,2.54) | 1.29 (0.44,3.77) | 2.38 (0.85,6.64) | NA | 0.10 (-0.35,0.56) |
| CRA+NCR | 1.48 (0.49,4.45) | 1.40 (0.39,4.90) | 1.40 (0.39,4.90) | 1.48 (0.49,4.45) | 0.57 (0.22,1.46) | NA | **0.51 (0.047,0.98)** |
| **CRA vs.** |  |  |  |  |  |  |  |
| CBT | NA | 2.58 (0.62,10.64) | **2.77 (1.04,7.41)** | NA | 0.68 (0.27,1.73) | NA | NA |
| **CRA+NCR vs.** |  |  |  |  |  |  |  |
| CM+CRA | 0.56 (0.19,1.69) | 0.49 (0.19,1.27) | 0.43 (0.16,1.17) | 0.76 (0.34,1.69) | 2.11 (0.58,7.69) | NA | -0.40 (-0.87,0.06) |
| **MBT vs.** |  |  |  |  |  |  |  |
| CBT | 0.66 (0.17,2.51) | 0.66 (0.17,2.51) | 1.04 (0.24,4.41) | 1.24 (0.53,2.90) | 1.24 (0.53,2.90) | NA | NA |
| TAU | 2.05 (0.73,5.76) | 2.05 (0.73,5.76) | NA | 1.66 (0.63,4.36) | 1.66 (0.63,4.36) | NA | NA |
| **NCR vs.** |  |  |  |  |  |  |  |
| TAU | 0.86 (0.47,1.55) | 0.86 (0.47,1.55) | 0.64 (0.37,1.11) | NA | NA | **0.34 (0.07,0.61)** | **0.34 (0.07,0.61)** |
| **SEPT vs.** |  |  |  |  |  |  |  |
| CBT | 0.73 (0.39,1.37) | 1.08 (0.64,1.84) | 0.93 (0.56,1.54) | 1.22 (0.73,2.03) | 1.02 (0.60,1.74) | NA | NA |
| TAU | 0.58 (0.32,1.08) | 0.92 (0.54,1.54) | 0.98 (0.59,1.63) | 0.81 (0.49,1.33) | 0.59 (0.34,1.04) | NA | NA |
| 12 step | **0.35 (0.19,0.63)** | 0.66 (0.39,1.10) | 0.86 (0.52,1.43) | 0.66 (0.40,1.09) | 0.60 (0.34,1.07) | NA | NA |
| **12 step vs.** |  |  |  |  |  |  |  |
| CBT | 1.07 (0.48,2.37) | 1.01 (0.53,1.90) | 0.70 (0.34,1.43) | 1.47 (0.60,3.57) | 1.44 (0.69,2.99) | -0.07 (-0.48,0.32) | -0.07 (-0.48,0.32) |
| TAU | **1.66 (1.24,2.23)** | **1.57 (1.18,2.09)** | 0.93 (0.67,1.29) | **1.37 (1.01,1.85)** | 1.30 (0.94,1.79) | NA | NA |
| **12 step+NCR vs.** |  |  |  |  |  |  |  |
| CM+CRA | 0.21 (0.04,1.04) | 0.22 (0.04,1.14) | **0.28 (0.10,0.77)** | 0.76 (0.34,1.69) | **6.36 (2.63,15.34)** | -0.48 (-1.13,0.16) | **-0.45 (-0.83,-0.07)** |
| CM+12 step | 0.52 (0.16,1.62) | 0.51 (0.13,1.95) | 0.51 (0.13,1.95) | 1.84 (0.69,4.89) | 2.15 (0.85,5.44) | NA | **-0.51 (-0.97,-0.05)** |
| CRA+NCR | 0.77 (0.23,2.58) | 0.72 (0.17,2.96) | 0.72 (0.17,2.96) | 2.72 (0.94,7.84) | 1.23 (0.48,3.14) | NA | 0.00 (-0.46,0.46) |

**S4 Table. Summary Estimates from Pairwise Meta-Analysis of Direct Comparisons.**

Significant results are bolded and underscored. Regarding dichotomous outcomes: for abstinence, an OR above 1 favors the first treatment, for dropouts an OR below 1 favors the first treatment. Regarding continuous outcomes: for abstinence a SMD above 0 favors the first treatment. CI= confidence interval, NA= not available, OR= odds ratio, SMD= standardized mean difference
