# Supplementary material for: Comparative efficacy and acceptability of psychosocial interventions for individuals with cocaine and amphetamine addiction: A systematic review and network meta-analysis
Source: PLoS Med. 2018 Dec 26;15(12):e1002715. doi: 10.1371/journal.pmed.1002715 (PMC6306153; doi:10.1371/journal.pmed.1002715)
Supplement: S5 Table — (DOCX) [file pmed.1002715.s020.docx]

**S5a Table. Heterogeneity Test Result, I2 and Heterogeneity Estimate.**

**Abstinence at 12 weeks.**

| **Comparison** | **No. of studies** | **P-value** | **I^2^** | **τ^2^** |
| --- | --- | --- | --- | --- |
| 12step vs CBT | 3 | 0.020 | 74.3% | 0.3635 |
| 12step vs TAU | 3 | 0.945 | 0.0% | 0.0000 |
| CBT vs TAU | 6 | 0.006 | 69.0% | 0.5325 |
| CM+CBT vs CBT | 6 | 0.407 | 1.4% | 0.0033 |
| CM vs NCR | 9 | 0.002 | 67.9% | 0.3835 |
| CM vs TAU | 14 | 0.004 | 57.1% | 0.2930 |
| 12step +NCR vs CM+CRA | 2 | 0.099 | 63.3% | 0.8372 |
| CM+CBT vs CM | 5 | 0.724 | 0.0% | 0.0000 |
| CBT vs CM | 4 | 0.728 | 0.0% | 0.0000 |

**S5b Table. Heterogeneity Test Result, I2 and Heterogeneity Estimate.**

**Abstinence at the End of Treatment.**

| **Comparison** | **No. of studies** | **P-value** | **I^2^** | **τ^2^** |
| --- | --- | --- | --- | --- |
| 12step vs CBT | 3 | 0.071 | 62.2% | 0.1970 |
| 12step vs TAU | 3 | 0.810 | 0.0% | 0.0000 |
| CBT vs TAU | 6 | 0.004 | 71.1% | 0.5457 |
| CM+CBT vs CBT | 6 | 0.150 | 38.4% | 0.1440 |
| CM+CRA vs CRA | 2 | 0.275 | 16.1% | 0.1602 |
| CM vs NCR | 9 | 0.002 | 67.8% | 0.3848 |
| CM vs TAU | 14 | 0.004 | 57.1% | 0.2930 |
| 12step +NCR vs CM+CRA | 2 | 0.190 | 41.7% | 0.6135 |
| CRA+NCR vs CM+CRA | 2 | 0.698 | 0.0% | 0.0000 |
| CM+CBT vs CM | 5 | 0.642 | 0.0% | 0.0000 |
| CBT vs CM | 4 | 0.728 | 0.0% | 0.0000 |

**S5c Table. Heterogeneity Test Result, I2 and Heterogeneity Estimate.**

**Abstinence at the Longest Follow-Up after Study Completion.**

| **Comparison** | **No. of studies** | **P-value** | **I^2^** | **τ^2^** |
| --- | --- | --- | --- | --- |
| 12step vs CBT | 3 | 0.112 | 54.4% | 0.2071 |
| CBT vs TAU | 3 | 0.028 | 72.0% | 0.3659 |
| 12step vs TAU | 2 | 0.328 | 0.0% | 0.0000 |
| CM+CRA vs CRA | 2 | 0.957 | 0.0% | 0.0000 |
| CM vs NCR | 7 | 0.014 | 62.4% | 0.2818 |
| CM vs TAU | 9 | 0.219 | 25.2% | 0.0490 |
| 12step +NCR vs CM+CRA | 2 | 0.368 | 0.0% | 0.0000 |
| CRA+NCR vs CM+CRA | 2 | 0.439 | 0.0% | 0.0000 |
| CM+CBT vs CM | 5 | 0.392 | 2.5% | 0.0045 |
| CM+CBT vs CBT | 5 | 0.121 | 45.2% | 0.1753 |
| CBT vs CM | 4 | 0.474 | 0.0% | 0.0000 |

**S5d Table. Heterogeneity Test Result, I2 and Heterogeneity Estimate.**

**Dropout due to any Cause at 12 Weeks.**

| **Comparison** | **No. of studies** | **P-value** | **I^2^** | **τ^2^** |
| --- | --- | --- | --- | --- |
| CBT vs TAU | 5 | 0.964 | 0.0% | 0.0000 |
| 12step vs CBT | 2 | 0.238 | 28.2% | 0.1855 |
| 12step vs TAU | 3 | 0.830 | 0.0% | 0.0000 |
| CM+CBT vs CBT | 4 | 0.497 | 0.0% | 0.0000 |
| CM+CBT vs CM | 3 | 0.099 | 56.8% | 0.3112 |
| CM vs TAU | 12 | 0.186 | 26.3% | 0.0600 |
| CM+CRA vs CRA | 2 | 0.411 | 0.0% | 0.0000 |
| CM vs NCR | 8 | 0.095 | 42.5% | 0.1682 |
| 12step +NCR vs CM+CRA | 2 | 0.059 | 72.0% | 1.2114 |
| CRA+NCR vs CM+CRA | 2 | 0.748 | 0.0% | 0.0000 |
| CBT vs CM | 2 | 0.604 | 0.0% | 0.0000 |

**S5e Table. Heterogeneity Test Result, I2 and Heterogeneity Estimate.**

**Dropout due to any Cause at the End of Treatment.**

| **Comparison** | **No. of studies** | **P-value** | **I^2^** | **τ^2^** |
| --- | --- | --- | --- | --- |
| CBT vs TAU | 5 | 0.932 | 0.0% | 0.0000 |
| 12step vs CBT | 2 | 0.280 | 14.2% | 0.0795 |
| 12step vs TAU | 3 | 0.523 | 0.0% | 0.0000 |
| CM+CBT vs CBT | 4 | 0.718 | 0.0% | 0.0000 |
| CM vs TAU | 12 | 0.186 | 26.3% | 0.0600 |
| CM+CRA vs CRA | 3 | 0.033 | 70.6% | 0.6755 |
| CM vs NCR | 8 | 0.134 | 36.9% | 0.1251 |
| 12step +NCR vs CM+CRA | 2 | 0.421 | 0.0% | 0.0000 |
| CRA+NCR vs CM+CRA | 2 | 0.064 | 70.9% | 0.6170 |
| CM+CBT vs CM | 3 | 0.320 | 12.1% | 0.0214 |
| CBT vs CM | 2 | 0.604 | 0.0% | 0.0000 |

**S5f Table. Heterogeneity Test Result, I2 and Heterogeneity Estimate.**

**Longest Duration of Abstinence at 12 Weeks.**

| **Comparison** | **No. of studies** | **P-value** | **I^2^** | **τ^2^** |
| --- | --- | --- | --- | --- |
| CBT vs TAU | 2 | 0.172 | 46.4% | 0.0332 |
| CM vs TAU | 11 | 0.036 | 48.4% | 0.0264 |
| CM vs NCR | 5 | 0.000 | 83.1% | 0.1968 |
| CBT vs CM | 2 | 0.264 | 19.8% | 0.0100 |
| CM + CBT vs CM | 2 | 0.014 | 83.4% | 0.2291 |
| CM + CBT vs CBT | 2 | 0.140 | 54.2% | 0.0486 |

**S5g Table. Heterogeneity Test Result, I2 and Heterogeneity Estimate.**

**Longest Duration of Abstinence at the End of Treatment.**

| **Comparison** | **No. of studies** | **P-value** | **I^2^** | **τ^2^** |
| --- | --- | --- | --- | --- |
| CBT vs TAU | 2 | 0.172 | 46.4% | 0.0332 |
| CM vs TAU | 11 | 0.036 | 48.4% | 0.0264 |
| 12-step + NCR vs CM + CRA | 2 | 0.729 | 0.0% | 0.0000 |
| CM+CRA vs CRA | 2 | 0.682 | 0.0% | 0.0000 |
| CM + CBT vs CM | 3 | 0.025 | 72.9% | 0.0934 |
| CM + CBT vs CBT | 3 | 0.196 | 38.6% | 0.0305 |
| CM vs NCR | 6 | 0.000 | 79.0% | 0.1464 |
| CBT vs CM | 2 | 0.264 | 19.8% | 0.0100 |
