# Supplementary material for: Comparative efficacy and acceptability of psychosocial interventions for individuals with cocaine and amphetamine addiction: A systematic review and network meta-analysis
Source: PLoS Med. 2018 Dec 26;15(12):e1002715. doi: 10.1371/journal.pmed.1002715 (PMC6306153; doi:10.1371/journal.pmed.1002715)
Supplement: S6 Table — (DOCX) [file pmed.1002715.s021.docx]

**S6 Table. Heterogeneity estimates and evaluation of the Global Incoherence.**

| **Model assumption** | | **SD Heterogeneity** | **Chi–squared incoherence** | | **P-value** | |
| --- | --- | --- | --- | --- | --- | --- |
| Abstinence at 12 weeks | | | | | |  |
| Coherence | | 0.474 | Chi–squared (16) = 4.95 | | 0.996 | |
| Incoherence | | 0.646 |  |  |  |  |
| Abstinence at the end of treatment | | | | | |  |
| Coherence | 0.461 | | | Chi–squared (19) = 5.03 | 0.999 |  |
| Incoherence | 0.666 | | |  |  |  |
| Longest follow-up after study completion | | | | | |  |
| Coherence | | 0.228 | Chi–squared (17) = 11.24 | | 0.843 | |
| Incoherence | | 0.414 |  |  |  |  |
| Dropout due to any cause at 12 weeks | | | | | |  |
| Coherence | | 0.190 | Chi–squared (13) = 13.72 | | 0.393 | |
| Incoherence | | 0.233 |  |  |  |  |
| Dropout due to any cause at the end of treatment | | | | | |  |
| Coherence | | 0.212 | Chi–squared (14) = 10.09 | | 0.755 | |
| Incoherence | | 0.283 |  |  |  |  |
| Longest duration of abstinence at 12 weeks | | | | | |  |
| Coherence | | 0.263 | Chi–squared (6) = 4.18 | | 0.651 | |
| Incoherence | | 0.296 |  |  |  |  |
| Longest duration of abstinence at the end of treatment | | | | | |  |
| Coherence | | 0.215 | Chi–squared (9) = 5.51 | | 0.7876 | |
| Incoherence | | 0.259 |  |  |  |  |
