# Supplementary material for: Comparative efficacy and acceptability of psychosocial interventions for individuals with cocaine and amphetamine addiction: A systematic review and network meta-analysis
Source: PLoS Med. 2018 Dec 26;15(12):e1002715. doi: 10.1371/journal.pmed.1002715 (PMC6306153; doi:10.1371/journal.pmed.1002715)
Supplement: S7 Table — (DOCX) [file pmed.1002715.s022.docx]

Tests of local incoherence revealed that the percentages for inconsistent loops were to be expected according to empirical data with the methods of Veroniki et al.^1^

**S7a Table. Evaluation of the Local Incoherence. Abstinence at 12 Weeks.**

| **Loop** | **ROR** | **z-value** | **P-value** | **95%CI** | **τ^2^** |
| --- | --- | --- | --- | --- | --- |
| CM+CRA - CRA+NCR - 12step +NCR | 5.083 | 1.412 | 0.158 | (1.00,48.58) | 0.000 |
| CM+CRA - CM+12step - 12step +NCR | 5.083 | 1.458 | 0.145 | (1.00,45.21) | 0.000 |
| TAU - CBT - MBT | 2.990 | 0.783 | 0.434 | (1.00,46.35) | 0.533 |
| CBT - 12step - SEPT | 2.916 | 2.061 | 0.039 | (1.05,8.07) | 0.000 |
| TAU - CBT - 12step | 1.419 | 0.525 | 0.599 | (1.00,5.24) | 0.284 |
| TAU - CM - CM+CBT | 1.413 | 0.422 | 0.673 | (1.00,7.03) | 0.190 |
| TAU - CBT - SEPT | 1.373 | 0.210 | 0.833 | (1.00,26.22) | 0.907 |
| TAU - CBT - CM+CBT | 1.367 | 0.343 | 0.731 | (1.00,8.16) | 0.307 |
| TAU - NCR - CM+CBT | 1.208 | 0.226 | 0.821 | (1.00,6.21) | 0.000 |
| CM - CBT - CM+CBT | 1.188 | 0.368 | 0.713 | (1.00,2.97) | 0.000 |
| NCR - CM - CM+CBT | 1.164 | 0.183 | 0.855 | (1.00,5.88) | 0.250 |
| NCR - CBT - CM+CBT | 1.154 | 0.159 | 0.874 | (1.00,6.79) | 0.058 |
| NCR - CM - CBT | 1.100 | 0.103 | 0.918 | (1.00,6.65) | 0.292 |
| TAU - CM - CBT | 1.075 | 0.134 | 0.893 | (1.00,3.09) | 0.289 |
| TAU - NCR - CM | 1.042 | 0.054 | 0.957 | (1.00,4.76) | 0.374 |
| TAU - NCR - CBT | 1.021 | 0.016 | 0.987 | (1.00,12.82) | 0.533 |
| TAU - 12step - SEPT | 1.006 | 0.013 | 0.990 | (1.00,2.51) | 0.000 |
| CRA+NCR - CM+12step - 12step +NCR | . | . | . | . | 0.000 |
| CM+CRA - CRA+NCR - CM+12step | . | . | . | . | 0.000 |

**Note**: Loops CRA+NCR - CM+12step - 12step +NCR and CM+CRA - CRA+NCR - CM+12step are formed only by multi-arm trial(s) - Consistent by definition

**S7b Table. Evaluation of the Local Incoherence. Abstinence at the End of Treatment.**

| **Loop** | **ROR** | **z-value** | **P-value** | **95%CI** | **τ^2^** |
| --- | --- | --- | --- | --- | --- |
| CM+CRA - CM+12-step - 12-step+NCR | 5.554 | 1.193 | 0.233 | (1.00,92.84) | 0.000 |
| TAU - CBT - MBT | 2.997 | 0.774 | 0.439 | (1.00,48.25) | 0.546 |
| CBT - 12-step - SEPT | 2.297 | 1.782 | 0.075 | (1.00,5.73) | 0.000 |
| TAU - CBT - CRA - CM+CRA | 2.221 | 0.505 | 0.614 | (1.00,49.12) | 0.526 |
| CM - CBT - CM+CBT | 1.816 | 1.297 | 0.195 | (1.00,4.47) | 0.000 |
| TAU - CBT - CM+CBT | 1.597 | 0.477 | 0.633 | (1.00,10.90) | 0.403 |
| TAU - CBT - 12-step | 1.481 | 0.605 | 0.545 | (1.00,5.29) | 0.259 |
| TAU - CM - CM+CRA | 1.468 | 0.366 | 0.715 | (1.00,11.50) | 0.293 |
| CM+CRA - CRA+NCR - CM+12-step | 1.455 | 0.330 | 0.741 | (1.00,13.47) | 0.000 |
| TAU - CM - CM+CBT | 1.376 | 0.389 | 0.697 | (1.00,6.88) | 0.192 |
| NCR - CBT - CM+CBT | 1.368 | 0.290 | 0.772 | (1.00,11.43) | 0.220 |
| TAU - CBT - SEPT | 1.277 | 0.158 | 0.875 | (1.00,26.65) | 0.996 |
| CM - CBT - CRA - CM+CRA | 1.213 | 0.195 | 0.846 | (1.00,8.49) | 0.000 |
| TAU - NCR - CM+CBT | 1.208 | 0.226 | 0.821 | (1.00,6.21) | 0.000 |
| TAU - 12-step - SEPT | 1.192 | 0.426 | 0.670 | (1.00,2.67) | 0.000 |
| NCR - CM - CM+CBT | 1.117 | 0.134 | 0.894 | (1.00,5.64) | 0.251 |
| NCR - CM - CBT | 1.081 | 0.085 | 0.932 | (1.00,6.55) | 0.293 |
| CM+CRA - CRA+NCR - 12-step+NCR | 1.069 | 0.057 | 0.955 | (1.00,10.72) | 0.000 |
| TAU - CM - CBT | 1.068 | 0.121 | 0.904 | (1.00,3.08) | 0.297 |
| TAU - NCR - CM | 1.027 | 0.034 | 0.973 | (1.00,4.68) | 0.375 |
| TAU - NCR - CBT | 1.024 | 0.018 | 0.986 | (1.00,13.35) | 0.546 |
| CRA+NCR - CM+12-step - 12-step+NCR | . | . | . | . | 0.000 |

**Note**: Loop CRA+NCR - CM+12step - 12step+NCR is formed only by multi-arm trial(s) - Consistent by definition

**S7c Table. Evaluation of the Local Incoherence. Abstinence at the Longest Follow-Up after Study Completion.**

| **Loop** | **ROR** | **z-value** | **P-value** | **95%CI** | **τ^2^** |
| --- | --- | --- | --- | --- | --- |
| CM+CRA - CM+12-step - 12-step+NCR | 2.514 | 0.779 | 0.436 | (1.00,25.57) | 0.000 |
| CM+CRA - CRA+NCR - CM+12-step | 2.253 | 0.673 | 0.501 | (1.00,23.96) | 0.000 |
| CBT - 12-step - SEPT | 1.960 | 1.335 | 0.182 | (1.00,5.27) | 0.000 |
| TAU - CBT - 12-step | 1.583 | 0.565 | 0.572 | (1.00,7.80) | 0.177 |
| TAU - NCR - CM+CBT | 1.530 | 0.518 | 0.604 | (1.00,7.63) | 0.000 |
| CM+CRA - CRA+NCR - 12-step+NCR | 1.528 | 0.349 | 0.727 | (1.00,16.56) | 0.000 |
| TAU - CM - CM+CBT | 1.522 | 0.667 | 0.505 | (1.00,5.23) | 0.006 |
| TAU - NCR - CBT | 1.510 | 0.306 | 0.760 | (1.00,21.20) | 0.366 |
| NCR - CM - CBT | 1.454 | 0.446 | 0.656 | (1.00,7.54) | 0.227 |
| TAU - 12-step - SEPT | 1.387 | 0.775 | 0.438 | (1.00,3.17) | 0.000 |
| NCR - CM - CM+CBT | 1.262 | 0.296 | 0.767 | (1.00,5.90) | 0.205 |
| TAU - CM - CBT | 1.256 | 0.543 | 0.587 | (1.00,2.86) | 0.062 |
| NCR - CBT - CM+CBT | 1.211 | 0.163 | 0.871 | (1.00,12.10) | 0.290 |
| CM - CBT - CRA - CM+CRA | 1.188 | 0.208 | 0.835 | (1.00,6.01) | 0.000 |
| CM - CBT - CM+CBT | 1.175 | 0.308 | 0.758 | (1.00,3.29) | 0.100 |
| TAU - CBT - SEPT | 1.147 | 0.286 | 0.775 | (1.00,2.93) | 0.000 |
| TAU - NCR - CM | 1.095 | 0.147 | 0.883 | (1.00,3.68) | 0.167 |
| TAU - CBT - CM+CBT | 1.009 | 0.010 | 0.992 | (1.00,5.97) | 0.181 |
| CRA+NCR - CM+12-step - 12-step+NCR | . | . | . | \| | 0.000 |

**Note**: Loop CRA+NCR-CM+12-step-12-step+NCR is formed only by multi-arm trial(s) - Consistent by definition

**S7d Table. Evaluation of the Local Incoherence. Dropout due to any Cause at 12 Weeks.**

| **Loop** | **ROR** | **z-value** | **P-value** | **95%CI** | **τ^2^** |
| --- | --- | --- | --- | --- | --- |
| CM+CRA - CRA+NCR - 12-step+NCR | 7.159 | 1.858 | 0.063 | (1.00,57.09) | 0.000 |
| CM+CRA - CM+12-step - 12-step+NCR | 6.258 | 1.664 | 0.096 | (1.00,54.24) | 0.000 |
| CBT - 12-step - SEPT | 3.151 | 1.141 | 0.254 | (1.00,22.63) | 0.000 |
| TAU - CM - CM+CRA | 3.151 | 1.520 | 0.129 | (1.00,13.85) | 0.060 |
| TAU - CBT - MBT | 1.932 | 0.977 | 0.329 | (1.00,7.24) | 0.000 |
| NCR - CBT - CM+CBT | 1.807 | 0.793 | 0.428 | (1.00,7.80) | 0.000 |
| NCR - CM - CBT | 1.643 | 0.653 | 0.514 | (1.00,7.28) | 0.117 |
| CM+CRA - CRA+NCR - CM+12-step | 1.298 | 0.270 | 0.787 | (1.00,8.61) | 0.000 |
| TAU - 12-step - SEPT | 1.200 | 0.444 | 0.657 | (1.00,2.68) | 0.000 |
| CM - CBT - CM+CBT | 1.189 | 0.209 | 0.834 | (1.00,6.01) | 0.141 |
| TAU - CBT - 12-step | 1.146 | 0.376 | 0.707 | (1.00,2.33) | 0.000 |
| TAU - CM - CBT | 1.139 | 0.336 | 0.737 | (1.00,2.44) | 0.000 |
| NCR - CM - CM+CBT | 1.076 | 0.093 | 0.926 | (1.00,5.02) | 0.200 |
| TAU - CBT - SEPT | 1.066 | 0.151 | 0.880 | (1.00,2.43) | 0.000 |
| CRA+NCR - CM+12-step - 12-step+NCR | . | . | . | . | 0.000 |

**Note**: Loop CRA+NCR - CM+12-step - 12-step +NCR is formed only by multi-arm trial(s) - Consistent by definition

**S7e Table. Evaluation of the Local Incoherence. Dropout due to any Cause at the End of Treatment.**

| **Loop** | **ROR** | **z-value** | **P-value** | **95%CI** | **τ^2^** |
| --- | --- | --- | --- | --- | --- |
| CM+CRA - CRA+NCR - 12-step+NCR | 4.671 | 1.452 | 0.147 | (1.00,37.44) | 0.000 |
| CM+CRA - CRA+NCR - CM+12-step | 3.741 | 1.543 | 0.123 | (1.00,19.98) | 0.000 |
| CBT - 12-step - SEPT | 2.883 | 1.041 | 0.298 | (1.00,21.17) | 0.000 |
| CM+CRA - CM+12-step - 12-step+NCR | 2.277 | 0.730 | 0.466 | (1.00,20.76) | 0.000 |
| TAU - CBT - MBT | 2.014 | 1.036 | 0.300 | (1.00,7.57) | 0.000 |
| TAU - CM - CM+CRA | 1.885 | 0.894 | 0.371 | (1.00,7.56) | 0.060 |
| CM - CBT - CRA - CM+CRA | 1.712 | 0.417 | 0.677 | (1.00,21.51) | 0.354 |
| NCR - CBT - CM+CBT | 1.613 | 0.643 | 0.520 | (1.00,6.94) | 0.000 |
| TAU - 12-step - SEPT | 1.495 | 0.893 | 0.372 | (1.00,3.61) | 0.000 |
| NCR - CM - CBT | 1.459 | 0.516 | 0.606 | (1.00,6.11) | 0.093 |
| TAU - CBT - SEPT | 1.215 | 0.434 | 0.664 | (1.00,2.92) | 0.000 |
| TAU - CBT - 12-step | 1.204 | 0.483 | 0.629 | (1.00,2.55) | 0.000 |
| TAU - CBT - CRA - CM+CRA | 1.162 | 0.181 | 0.856 | (1.00,5.89) | 0.049 |
| TAU - CM - CBT | 1.093 | 0.227 | 0.820 | (1.00,2.35) | 0.000 |
| CM - CBT - CM+CBT | 1.079 | 0.120 | 0.905 | (1.00,3.73) | 0.000 |
| NCR - CM - CM+CBT | 1.037 | 0.056 | 0.955 | (1.00,3.77) | 0.098 |
| CRA+NCR - CM+12-step - 12-step+NCR | . | . | . | \| | 0.000 |

**Note**: Loop CRA+NCR - CM+12step - 12step+NCR is formed only by multi-arm trial(s) - Consistent by definition

**S7f Table. Evaluation of the Local Incoherence. Longest Duration of Abstinence at 12 Weeks.**

| **Loop** | **ROR** | **z-value** | **P-value** | **95%CI** | **τ^2^** |
| --- | --- | --- | --- | --- | --- |
| NCR - CBT - CM+CBT | 1.527 | 1.205 | 0.228 | (1.00,3.04) | 0.000 |
| TAU - NCR - CBT | 1.505 | 1.453 | 0.146 | (1.00,2.61) | 0.000 |
| NCR - CM - CBT | 1.375 | 0.512 | 0.609 | (1.00,4.65) | 0.121 |
| TAU - NCR - CM | 1.356 | 0.751 | 0.453 | (1.00,3.00) | 0.076 |
| TAU - CM - CBT | 1.193 | 0.605 | 0.545 | (1.00,2.11) | 0.026 |
| NCR - CM - CM+CBT | 1.096 | 0.129 | 0.897 | (1.00,4.38) | 0.174 |
| CM - CBT - CM+CBT | . | . | . | . | 0.091 |

**Note**: Loop CM - CBT - CM+CBT is formed only by multi-arm trial(s) - Consistent by definition

**S7g Table. Evaluation of the Local Incoherence. Longest Duration of Abstinence at the End of Treatment.**

| **Loop** | **ROR** | **z-value** | **P-value** | **95%CI** | **τ^2^** |
| --- | --- | --- | --- | --- | --- |
| TAU - NCR - CBT | 1.505 | 1.453 | 0.146 | (1.00,2.61) | 0.000 |
| NCR - CM - CBT | 1.453 | 0.688 | 0.491 | (1.00,4.21) | 0.086 |
| CM - CBT - CM+CBT | 1.320 | 0.888 | 0.375 | (1.00,2.43) | 0.023 |
| TAU-NCR-CM | 1.296 | 0.674 | 0.500 | (1.00,2.75) | 0.068 |
| NCR-CBT-CM+CBT | 1.261 | 0.704 | 0.481 | (1.00,2.41) | 0.000 |
| TAU - CM - CBT | 1.193 | 0.605 | 0.545 | (1.00,2.11) | 0.026 |
| CM+CRA - CM+12-step - 12-step+NCR | 1.151 | 0.301 | 0.764 | (1.00,2.88) | 0.000 |
| CM+CRA - CRA+NCR - 12-step+NCR | 1.151 | 0.299 | 0.765 | (1.00,2.90) | 0.000 |
| NCR - CM - CM+CBT | 1.087 | 0.155 | 0.877 | (1.00,3.12) | 0.100 |
| CRA+NCR - CM+12-step - 12-step+NCR | . | . | . | . | 0.000 |
| CM+CRA - CRA+NCR - CM+12-step | . | . | . | . | 0.000 |

Note: Loops CM+CRA-CRA+NCR-CM+12-step and CRA+NCR-CM+12-step-12-step+NCR are formed only by multi-arm trial(s) - Consistent by definition

**References**

1. Veroniki AA, Vasiliadis HS, Higgins JP, Salanti G. Evaluation of inconsistency in networks of interventions. Int J Epidemiol 2013;42(1):332-345. doi: 10.1093/ije/dys222 pmid: 23508418
