# Supplementary material for: Comparative efficacy and acceptability of psychosocial interventions for individuals with cocaine and amphetamine addiction: A systematic review and network meta-analysis
Source: PLoS Med. 2018 Dec 26;15(12):e1002715. doi: 10.1371/journal.pmed.1002715 (PMC6306153; doi:10.1371/journal.pmed.1002715)
Supplement: S8 Table — (DOCX) [file pmed.1002715.s023.docx]

Tests of incoherence by node-splitting method fitted the node-splitting model of Dias et al.^1^ The results reported the estimated direct and indirect treatment effects and their difference; the P-value for the difference is the test of coherence.

**S8a Table. Evaluation of the Incoherence by Side-Splitting Model. Abstinence at 12 Weeks.**

| **Comparisons** | **Direct** | | **Indirect** | | **Difference** | | | **τ^2^** |
| --- | --- | --- | --- | --- | --- | --- | --- | --- |
|  | **LogOR** | **SE** | **LogOR** | **SE** | **LogOR** | **SE** | **P-value** |  |
| TAU vs NCR | -0.15 | 0.58 | -0.11 | 0.30 | -0.04 | 0.65 | 0.946 | 0.4897 |
| TAU vs CM | 0.81 | 0.20 | 0.89 | 0.40 | -0.07 | 0.45 | 0.869 | 0.4864 |
| TAU vs CBT | 0.02 | 0.29 | 0.32 | 0.31 | -0.30 | 0.42 | 0.481 | 0.4807 |
| TAU vs 12 step | 0.53 | 0.32 | -0.05 | 0.48 | 0.59 | 0.58 | 0.312 | 0.4725 |
| TAU vs SEPT | -0.53 | 0.58 | -0.29 | 0.93 | -0.24 | 1.08 | 0.824 | 0.4900 |
| TAU vs CM + CBT | 1.15 | 0.74 | 0.95 | 0.30 | 0.20 | 0.80 | 0.805 | 0.4840 |
| TAU vs MBT | 0.72 | 0.71 | -0.28 | 0.85 | 1.00 | 1.11 | 0.366 | 0.4757 |
| NCR vs CM | 0.95 | 0.23 | 0.92 | 0.80 | 0.03 | 0.82 | 0.975 | 0.4879 |
| NCR vs CBT | 0.19 | 0.77 | 0.29 | 0.32 | -0.09 | 0.83 | 0.911 | 0.4842 |
| NCR vs CM + CBT | 1.13 | 0.72 | 1.08 | 0.36 | 0.06 | 0.80 | 0.944 | 0.4853 |
| CM vs CBT | -0.81 | 0.34 | -0.56 | 0.30 | -0.24 | 0.45 | 0.587 | 0.4806 |
| CM vs CM + CBT | 0.08 | 0.29 | 0.35 | 0.52 | -0.27 | 0.60 | 0.646 | 0.4820 |
| CM vs CM + CRA | 1.20 | 0.65 | -0.35 | 55.47 | 1.55 | 55.47 | 0.978 | 0.4745 |
| CBT vs 12 step | 0.08 | 0.35 | 0.40 | 0.46 | -0.32 | 0.58 | 0.585 | 0.4851 |
| CBT vs SEPT | -0.31 | 0.57 | -1.43 | 0.91 | 1.12 | 1.07 | 0.294 | 0.4736 |
| CBT vs CM + CBT | 0.80 | 0.28 | 0.88 | 0.61 | -0.07 | 0.67 | 0.913 | 0.4879 |
| CBT vs MBT | -0.41 | 0.83 | 0.60 | 0.74 | -1.00 | 1.11 | 0.366 | 0.4757 |
| CRA vs CM + CRA | 1.46 | 0.74 | 2.60 | 2.00 | -1.14 | 2.00 | 0.995 | 0.4745 |
| 12 step vs SEPT | -1.05 | 0.57 | -0.09 | 1.00 | -0.96 | 1.13 | 0.397 | 0.4805 |
| CM + CRA vs CRA + NCR | -0.57 | 0.73 | -3.82 | 2.28 | 3.25 | 2.37 | 0.171 | 0.4683 |
| CM + CRA vs CM + 12 step | -0.17 | 0.70 | -3.42 | 2.27 | 3.25 | 2.37 | 0.171 | 0.4683 |
| CM + CRA vs 12 step + NCR | -1.46 | 0.58 | -5.51 | 141.43 | 4.06 | 141.43 | 0.977 | 0.4745 |
| CRA + NCR vs CM + 12 step | . | . | . | . | . | . | . | . |
| CRA + NCR vs 12 step + NCR | -0.26 | 0.77 | -3.51 | 2.23 | 3.25 | 2.37 | 0.171 | 0.4683 |
| CM + 12 step vs 12 step + NCR | -0.65 | 0.75 | -3.90 | 2.23 | 3.25 | 2.37 | 0.171 | 0.4683 |

**S8b Table. Evaluation of the Incoherence by Side-Splitting Model. Abstinence at the End of Treatment.**

| **Comparisons** | **Direct** | | **Indirect** | | **Difference** | | | **τ^2^** |
| --- | --- | --- | --- | --- | --- | --- | --- | --- |
|  | **LogOR** | **SE** | **LogOR** | **SE** | **LogOR** | **SE** | **P-value** |  |
| TAU vs NCR | -0.15 | 0.56 | -0.16 | 0.30 | 0.01 | 0.64 | 0.993 | 0.4756 |
| TAU vs CM | 0.81 | 0.20 | 0.76 | 0.36 | 0.06 | 0.41 | 0.893 | 0.4731 |
| TAU vs CBT | 0.01 | 0.28 | 0.32 | 0.29 | -0.32 | 0.40 | 0.434 | 0.4638 |
| TAU vs 12 step | 0.47 | 0.31 | -0.08 | 0.47 | 0.55 | 0.57 | 0.333 | 0.4611 |
| TAU vs SEPT | -0.08 | 0.54 | 0.26 | 0.88 | -0.35 | 1.03 | 0.738 | 0.4751 |
| TAU vs CM + CBT | 1.15 | 0.73 | 0.86 | 0.29 | 0.29 | 0.79 | 0.712 | 0.4697 |
| TAU vs CM + CRA | 0.76 | 0.66 | 1.26 | 0.56 | -0.50 | 0.87 | 0.564 | 0.4708 |
| TAU vs MBT | 0.72 | 0.70 | -0.28 | 0.85 | 1.00 | 1.10 | 0.362 | 0.4623 |
| NCR vs CM | 0.96 | 0.22 | 0.90 | 0.78 | 0.05 | 0.80 | 0.946 | 0.4736 |
| NCR vs CBT | 0.19 | 0.76 | 0.33 | 0.31 | -0.14 | 0.82 | 0.862 | 0.4701 |
| NCR vs CM + CBT | 1.14 | 0.71 | 1.03 | 0.35 | 0.12 | 0.79 | 0.883 | 0.4711 |
| CM vs CBT | -0.81 | 0.33 | -0.52 | 0.28 | -0.29 | 0.44 | 0.502 | 0.4645 |
| CM vs CM + CBT | 0.11 | 0.29 | 0.06 | 0.50 | 0.04 | 0.58 | 0.941 | 0.4723 |
| CM vs CM + CRA | 0.32 | 0.62 | 0.19 | 0.59 | 0.13 | 0.85 | 0.881 | 0.4720 |
| CBT vs CRA | 0.95 | 0.86 | 0.27 | 0.80 | 0.68 | 1.17 | 0.563 | 0.4679 |
| CBT vs 12 step | 0.00 | 0.34 | 0.39 | 0.45 | -0.39 | 0.56 | 0.491 | 0.4690 |
| CBT vs SEPT | 0.08 | 0.54 | -0.75 | 0.88 | 0.83 | 1.03 | 0.420 | 0.4671 |
| CBT vs CM + CBT | 0.68 | 0.28 | 0.97 | 0.58 | -0.29 | 0.64 | 0.649 | 0.4729 |
| CBT vs MBT | -0.41 | 0.82 | 0.60 | 0.73 | -1.00 | 1.10 | 0.362 | 0.4623 |
| CRA vs CM + CRA | 0.50 | 0.63 | -0.18 | 0.99 | 0.68 | 1.17 | 0.563 | 0.4679 |
| 12 step vs SEPT | -0.42 | 0.54 | 0.12 | 0.97 | -0.54 | 1.11 | 0.626 | 0.4740 |
| CM + CRA vs CRA + NCR | -0.71 | 0.58 | -3.99 | 2.84 | 3.28 | 2.89 | 0.256 | 0.4599 |
| CM + CRA vs CM + 12 step | -0.20 | 0.74 | -1.94 | 1.82 | 1.74 | 1.96 | 0.375 | 0.4641 |
| CM + CRA vs 12 step + NCR | -1.38 | 0.68 | -1.70 | 2.25 | 0.32 | 2.32 | 0.890 | 0.4676 |
| CRA + NCR vs CM + 12 step | 0.34 | 0.79 | 0.66 | 2.15 | -0.32 | 2.32 | 0.890 | 0.4676 |
| CRA + NCR vs 12 step + NCR | -0.34 | 0.85 | -1.28 | 1.46 | 0.94 | 1.68 | 0.577 | 0.4664 |
| CM + 12 step vs 12 step + NCR | -0.65 | 0.82 | -3.94 | 2.73 | 3.28 | 2.89 | 0.256 | 0.4599 |

**S8c Table. Evaluation of the Incoherence by Side-Splitting Model. Abstinence at the Longest Follow-Up after Study Completion.**

| **Comparisons** | **Direct** | | **Indirect** | | **Difference** | | | **τ^2^** |
| --- | --- | --- | --- | --- | --- | --- | --- | --- |
|  | **LogOR** | **SE** | **LogOR** | **SE** | **LogOR** | **SE** | **P-value** |  |
| TAU vs NCR | -0.44 | 0.38 | -0.57 | 0.26 | 0.13 | 0.46 | 0.776 | 0.2628 |
| TAU vs CM | 0.08 | 0.16 | 0.22 | 0.32 | -0.15 | 0.36 | 0.679 | 0.2573 |
| TAU vs CBT | 0.17 | 0.26 | -0.05 | 0.25 | 0.22 | 0.36 | 0.542 | 0.2576 |
| TAU vs 12 step | -0.05 | 0.24 | -0.53 | 0.44 | 0.48 | 0.50 | 0.334 | 0.2449 |
| TAU vs SEPT | -0.01 | 0.36 | -0.42 | 0.64 | 0.40 | 0.74 | 0.586 | 0.2564 |
| TAU vs CM + CBT | 0.39 | 0.63 | 0.20 | 0.24 | 0.19 | 0.67 | 0.774 | 0.2441 |
| NCR vs CM | 0.64 | 0.20 | 0.55 | 0.57 | 0.10 | 0.60 | 0.874 | 0.2607 |
| NCR vs CBT | 0.30 | 0.58 | 0.64 | 0.27 | -0.34 | 0.64 | 0.591 | 0.2496 |
| NCR vs CM + CBT | 0.51 | 0.57 | 0.81 | 0.29 | -0.30 | 0.63 | 0.638 | 0.2486 |
| CM vs CBT | -0.08 | 0.25 | -0.01 | 0.25 | -0.07 | 0.36 | 0.848 | 0.2523 |
| CM vs CM + CBT | 0.05 | 0.22 | 0.48 | 0.47 | -0.44 | 0.52 | 0.403 | 0.2449 |
| CM vs CM + CRA | 0.96 | 0.51 | 1.17 | 0.75 | -0.21 | 0.91 | 0.817 | 0.2451 |
| CBT vs CRA | 1.02 | 0.56 | 0.81 | 0.71 | 0.21 | 0.91 | 0.816 | 0.2451 |
| CBT vs 12 step | -0.26 | 0.29 | -0.16 | 0.40 | -0.10 | 0.49 | 0.833 | 0.2680 |
| CBT vs SEPT | -0.07 | 0.37 | -0.50 | 0.67 | 0.42 | 0.77 | 0.582 | 0.2638 |
| CBT vs CM + CBT | 0.07 | 0.23 | 0.56 | 0.45 | -0.49 | 0.50 | 0.327 | 0.2354 |
| CBT vs MBT | 0.04 | 0.77 | -0.07 | 199.99 | 0.11 | 199.99 | 1.000 | 0.2286 |
| CRA vs CM + CRA | 0.19 | 0.47 | -0.02 | 0.78 | 0.21 | 0.91 | 0.817 | 0.2451 |
| 12 step vs SEPT | -0.14 | 0.35 | 0.82 | 0.71 | -0.96 | 0.79 | 0.225 | 0.2358 |
| CM + CRA vs CRA + NCR | -0.84 | 0.53 | -2.43 | 2.08 | 1.59 | 2.12 | 0.453 | 0.2316 |
| CM + CRA vs CM + 12 step | -0.20 | 0.62 | -1.94 | 1.54 | 1.74 | 1.65 | 0.293 | 0.2276 |
| CM + CRA vs 12 step + NCR | -1.25 | 0.53 | -2.58 | 2.14 | 1.33 | 2.17 | 0.542 | 0.2332 |
| CRA + NCR vs CM + 12 step | 0.34 | 0.68 | 1.66 | 2.04 | -1.32 | 2.17 | 0.542 | 0.2332 |
| CRA + NCR vs 12 step + NCR | -0.35 | 0.75 | -0.49 | 1.19 | 0.14 | 1.41 | 0.919 | 0.2366 |
| CM + 12 step vs 12 step + NCR | -0.65 | 0.71 | -2.25 | 1.95 | 1.59 | 2.12 | 0.453 | 0.2316 |

**S8d Table. Evaluation of the Incoherence by Side-Splitting Model. Dropout due to any Cause at 12 Weeks**.

| **Comparisons** | **Direct** | | **Indirect** | | **Difference** | | | **τ^2^** |
| --- | --- | --- | --- | --- | --- | --- | --- | --- |
|  | **LogOR** | **SE** | **LogOR** | **SE** | **LogOR** | **SE** | **P-value** |  |
| TAU vs CM | -0.41 | 0.13 | 0.58 | 0.50 | -0.99 | 0.51 | 0.114 | 0.1801 |
| TAU vs CBT | -0.37 | 0.17 | -0.06 | 0.37 | -0.31 | 0.41 | 0.894 | 0.2099 |
| TAU vs 12 step | 0.32 | 0.17 | 0.00 | 0.58 | 0.31 | 0.60 | 0.481 | 0.2184 |
| TAU vs SEPT | -0.21 | 0.28 | 0.04 | 0.50 | -0.25 | 0.57 | 0.801 | 0.2202 |
| TAU vs CM + CRA | -0.87 | 0.45 | -1.98 | 0.49 | 1.12 | 0.66 | 0.122 | 0.1704 |
| TAU vs MBT | 0.51 | 0.50 | -0.13 | 0.46 | 0.64 | 0.68 | 0.354 | 0.1924 |
| NCR vs CM | 0.30 | 0.19 | 0.62 | 0.92 | -0.32 | 0.94 | 0.716 | 0.2052 |
| NCR vs CBT | -0.21 | 0.53 | 0.50 | 0.29 | -0.70 | 0.61 | 0.269 | 0.1855 |
| NCR vs CM + CBT | 0.34 | 0.51 | 0.10 | 0.45 | 0.24 | 0.68 | 0.758 | 0.2057 |
| CM vs CBT | 0.08 | 0.56 | 0.01 | 0.22 | 0.08 | 0.60 | 0.801 | 0.2056 |
| CM vs CM + CBT | -0.071 | 0.40 | -0.17 | 0.50 | 0.10 | 0.65 | 0.982 | 0.2050 |
| CM vs CM + CRA | -1.59 | 0.48 | -0.48 | 0.46 | -1.12 | 0.66 | 0.122 | 0.1704 |
| CBT vs 12 step | 0.51 | 0.28 | 0.72 | 0.30 | -0.21 | 0.41 | 0.533 | 0.2182 |
| CBT vs SEPT | 0.20 | 0.29 | 0.04 | 0.58 | 0.16 | 0.65 | 0.928 | 0.2201 |
| CBT vs CM + CBT | -0.27 | 0.51 | -1.04 | 0.59 | 0.77 | 0.78 | 0.140 | 0.1853 |
| CBT vs MBT | 0.22 | 0.44 | 0.86 | 0.52 | -0.64 | 0.68 | 0.354 | 0.1924 |
| CRA vs CM + CRA | -0.98 | 0.50 | -2.65 | 103.41 | 1.67 | 103.41 | 0.987 | 0.1906 |
| 12 step vs SEPT | -0.41 | 0.28 | -0.57 | 0.61 | 0.16 | 0.67 | 0.842 | 0.2200 |
| CM + CRA vs CRA + NCR | -0.27 | 0.41 | 3.53 | 1.86 | -3.79 | 1.90 | 0.052 | 0.1721 |
| CM + CRA vs CM + 12 step | 0.26 | 0.55 | 1.38 | 1.24 | -1.12 | 1.38 | 0.424 | 0.1976 |
| CM + CRA vs 12 step + NCR | 1.41 | 0.45 | 0.26 | 1.52 | 1.15 | 1.60 | 0.485 | 0.1972 |
| CRA + NCR vs CM + 12 step | 0.39 | 0.57 | 1.55 | 1.47 | -1.15 | 1.60 | 0.485 | 0.1972 |
| CRA + NCR vs 12 step + NCR | 0.95 | 0.54 | 2.81 | 0.96 | -1.87 | 1.12 | 0.104 | 0.1822 |
| CM + 12 step vs 12 step + NCR | 0.61 | 0.50 | 4.41 | 1.85 | -3.80 | 1.90 | 0.052 | 0.1721 |

**S8e Table. Evaluation of the Incoherence by Side-Splitting Model. Dropout at the End of Treatment.**

| **Comparisons** | **Direct** | | **Indirect** | | **Difference** | | | **τ^2^** |
| --- | --- | --- | --- | --- | --- | --- | --- | --- |
|  | **LogOR** | **SE** | **LogOR** | **SE** | **LogOR** | **SE** | **P-value** |  |
| TAU vs CM | -0.42 | 0.14 | 0.00 | 0.31 | -0.42 | 0.34 | 0.216 | 0.2076 |
| TAU vs CBT | -0.40 | 0.20 | -0.36 | 0.26 | -0.05 | 0.33 | 0.888 | 0.2283 |
| TAU vs 12 step | 0.26 | 0.22 | -0.17 | 0.62 | 0.43 | 0.66 | 0.514 | 0.2318 |
| TAU vs SEPT | -0.52 | 0.36 | -0.05 | 0.60 | -0.47 | 0.72 | 0.514 | 0.2247 |
| TAU vs CM + CRA | -1.11 | 0.48 | -1.41 | 0.39 | 0.30 | 0.62 | 0.635 | 0.2244 |
| TAU vs MBT | 0.51 | 0.54 | -0.20 | 0.51 | 0.71 | 0.74 | 0.338 | 0.2125 |
| NCR vs CM | 0.20 | 0.18 | 0.50 | 0.84 | -0.30 | 0.86 | 0.728 | 0.2235 |
| NCR vs CBT | -0.21 | 0.53 | 0.28 | 0.27 | -0.49 | 0.60 | 0.412 | 0.2158 |
| NCR vs CM + CBT | 0.33 | 0.51 | 0.19 | 0.32 | 0.15 | 0.60 | 0.806 | 0.2254 |
| CM vs CBT | -0.10 | 0.37 | -0.02 | 0.21 | -0.08 | 0.42 | 0.845 | 0.2259 |
| CM vs CM + CBT | 0.00 | 0.26 | 0.05 | 0.46 | -0.05 | 0.53 | 0.921 | 0.2259 |
| CM vs CM + CRA | -1.33 | 0.47 | -0.67 | 0.40 | -0.66 | 0.61 | 0.287 | 0.2081 |
| CBT vs CRA | -0.38 | 0.52 | -0.86 | 0.49 | 0.48 | 0.72 | 0.506 | 0.2144 |
| CBT vs 12 step | 0.39 | 0.35 | 0.77 | 0.32 | -0.37 | 0.47 | 0.426 | 0.2276 |
| CBT vs SEPT | 0.02 | 0.36 | -0.10 | 0.67 | 0.12 | 0.76 | 0.875 | 0.2354 |
| CBT vs CM + CBT | 0.20 | 0.30 | -0.24 | 0.41 | 0.45 | 0.51 | 0.377 | 0.2200 |
| CBT vs MBT | 0.22 | 0.48 | 0.93 | 0.56 | -0.71 | 0.74 | 0.338 | 0.2125 |
| CRA vs CM + CRA | -0.17 | 0.32 | -0.65 | 0.64 | 0.48 | 0.72 | 0.505 | 0.2144 |
| 12 step vs SEPT | -0.50 | 0.36 | -0.99 | 0.74 | 0.49 | 0.84 | 0.555 | 0.2224 |
| CM + CRA vs CRA + NCR | 0.71 | 0.38 | 3.17 | 2.03 | -2.46 | 2.08 | 0.237 | 0.2086 |
| CM + CRA vs CM + 12 step | 0.87 | 0.56 | -0.48 | 1.21 | 1.35 | 1.37 | 0.325 | 0.2044 |
| CM + CRA vs 12 step + NCR | 1.86 | 0.47 | -1.04 | 1.38 | 2.90 | 1.48 | 0.050 | 0.1861 |
| CRA + NCR vs CM + 12 step | -0.55 | 0.51 | 2.35 | 1.39 | -2.90 | 1.48 | 0.050 | 0.1861 |
| CRA + NCR vs 12 step + NCR | 0.21 | 0.51 | 2.49 | 0.96 | -2.27 | 1.09 | 0.037 | 0.1820 |
| CM + 12 step vs 12 step + NCR | 0.77 | 0.52 | 3.22 | 2.01 | -2.46 | 2.08 | 0.237 | 0.2086 |

**S8f Table. Evaluation of the Incoherence by Side-Splitting Model. Longest Duration of Abstinence at 12 Weeks.**

| **Comparisons** | **Direct** | | **Indirect** | | **Difference** | | | **τ^2^** |
| --- | --- | --- | --- | --- | --- | --- | --- | --- |
|  | **LogOR** | **SE** | **LogOR** | **SE** | **LogOR** | **SE** | **P-value** |  |
| TAU vs NCR | 0.34 | 0.30 | 0.00 | 0.20 | 0.35 | 0.36 | 0.335 | 0.2687 |
| TAU vs CM | 0.59 | 0.10 | 0.86 | 0.30 | -0.27 | 0.31 | 0.392 | 0.2675 |
| TAU vs CBT | 0.09 | 0.24 | 0.03 | 0.25 | 0.06 | 0.34 | 0.864 | 0.2775 |
| NCR vs CM | 0.56 | 0.16 | 0.21 | 0.44 | 0.36 | 0.47 | 0.447 | 0.2732 |
| NCR vs CBT | 0.15 | 0.33 | -0.15 | 0.25 | 0.29 | 0.42 | 0.485 | 0.2658 |
| NCR vs CM + CBT | 0.64 | 0.35 | 0.67 | 0.35 | -0.02 | 0.49 | 0.964 | 0.2777 |
| CM vs CBT | -0.66 | 0.24 | -0.45 | 0.24 | -0.22 | 0.34 | 0.523 | 0.2684 |
| CM vs CM + CBT | 0.03 | 0.24 | 0.55 | 0.49 | -0.51 | 0.54 | 0.347 | 0.2567 |
| CBT vs 12 step | -0.08 | 0.33 | -0.11 | 63.24 | 0.03 | 63.24 | 1.000 | 0.2633 |
| CBT vs CM + CBT | 0.70 | 0.24 | 0.59 | 0.65 | 0.12 | 0.69 | 0.864 | 0.2775 |
| CRA vs CM + CRA | 0.72 | 0.42 | 0.49 | 89.45 | 0.24 | 89.45 | 0.998 | 0.2633 |
| CM + CRA vs 12 step + NCR | -0.49 | 0.42 | -0.72 | 89.47 | 0.24 | 89.47 | 0.998 | 0.2633 |

**S8g Table. Evaluation of the Incoherence by Side-Splitting Model. Longest Duration of Abstinence at the End of Treatment.**

| **Comparisons** | **Direct** | | **Indirect** | | **Difference** | | | **τ^2^** |
| --- | --- | --- | --- | --- | --- | --- | --- | --- |
|  | **LogOR** | **SE** | **LogOR** | **SE** | **LogOR** | **SE** | **P-value** |  |
| TAU vs NCR | 0.34 | 0.26 | 0.06 | 0.16 | 0.29 | 0.31 | 0.349 | 0.2209 |
| TAU vs CM | 0.58 | 0.09 | 0.74 | 0.25 | -0.16 | 0.27 | 0.544 | 0.2227 |
| TAU vs CBT | 0.09 | 0.21 | 0.10 | 0.20 | -0.02 | 0.29 | 0.956 | 0.2275 |
| TAU vs CM + CRA | 0.58 | 0.30 | 0.33 | 15.81 | 0.25 | 15.82 | 0.987 | 0.2159 |
| NCR vs CM | 0.50 | 0.13 | 0.19 | 0.37 | 0.31 | 0.39 | 0.42 | 0.2234 |
| NCR vs CBT | 0.15 | 0.30 | -0.13 | 0.21 | 0.28 | 0.36 | 0.441 | 0.2157 |
| NCR vs CM + CBT | 0.64 | 0.31 | 0.60 | 0.22 | 0.04 | 0.38 | 0.909 | 0.2265 |
| CM vs CBT | -0.66 | 0.21 | -0.37 | 0.19 | -0.30 | 0.28 | 0.287 | 0.2105 |
| CM vs CM + CBT | 0.12 | 0.17 | 0.24 | 0.34 | -0.11 | 0.38 | 0.762 | 0.2232 |
| CBT vs 12 step | -0.08 | 0.30 | -0.18 | 63.24 | 0.11 | 63.24 | 0.999 | 0.2159 |
| CBT vs CM + CBT | 0.62 | 0.18 | 0.76 | 0.35 | -0.13 | 0.40 | 0.733 | 0.2277 |
| CRA vs CM + CRA | 0.81 | 0.23 | 1.09 | 44.73 | -0.29 | 44.73 | 0.995 | 0.2159 |
| CM + CRA vs CRA + NCR | -0.41 | 0.33 | -0.69 | 0.98 | 0.28 | 1.03 | 0.784 | 0.2229 |
| CM + CRA vs CM + 12 step | 0.11 | 0.32 | -0.17 | 0.98 | 0.28 | 1.03 | 0.784 | 0.2229 |
| CM + CRA vs 12 step + NCR | -0.46 | 0.25 | -1.19 | 44.74 | 0.72 | 44.74 | 0.987 | 0.2159 |
| CRA + NCR vs CM + 12 step | . | . | . | . | . | . | . | . |
| CRA + NCR vs 12 step + NCR | 0.00 | 0.32 | -0.28 | 0.98 | 0.28 | 1.03 | 0.784 | 0.2229 |
| CM + 12 step vs 12 step + NCR | -0.51 | 0.32 | -0.80 | 0.98 | 0.28 | 1.03 | 0.784 | 0.2229 |

**References**

1. Dias S, Welton NJ, Caldwell DM, Ades AE. Checking consistency in mixed treatment comparison meta-analysis. Stat Med 2010;29(7-8):932-944. doi: 10.1002/sim.3767 pmid: 20213715.
