# Supplementary material for: Comparative efficacy and acceptability of psychosocial interventions for individuals with cocaine and amphetamine addiction: A systematic review and network meta-analysis
Source: PLoS Med. 2018 Dec 26;15(12):e1002715. doi: 10.1371/journal.pmed.1002715 (PMC6306153; doi:10.1371/journal.pmed.1002715)
Supplement: S9 Table — (DOCX) [file pmed.1002715.s024.docx]

**S9a Table. Treatment Ranking. Abstinence at 12 Weeks.**

| **Interventions** | **Abbreviation** | **SUCRA (%)** | **PrBest** | **Mean Rank** |
| --- | --- | --- | --- | --- |
| Cognitive behavioural therapy | CBT | 33.2 | 0.0 | 9.0 |
| Contingency management | CM | 65.1 | 0.7 | 5.2 |
| Contingency management plus cognitive behavioural therapy | CM + CBT | 71.9 | 4.3 | 4.4 |
| Contingency management plus community reinforcement approach | CM + CRA | 95.5 | 62.1 | 1.5 |
| Contingency management plus twelve step programme | CM + 12 step | 82.1 | 22.1 | 3.2 |
| Community reinforcement approach | CRA | 48.9 | 1.8 | 7.1 |
| Community reinforcement approach plus non-contingent rewards | CRA + NCR | 69.6 | 7.7 | 4.7 |
| Meditation based treatments | MBT | 40.5 | 1.0 | 8.1 |
| Non-contingent rewards | NCR | 18.0 | 0.0 | 10.8 |
| Supportive-expressive psychodynamic therapy | SEPT | 9.8 | 0.0 | 11.8 |
| Treatment as usual | TAU | 23.4 | 0.0 | 10.2 |
| Twelve step programme | 12 step | 44.1 | 0.1 | 7.7 |
| Twelve step programme plus non-contingent rewards | 12 step + NCR | 47.9 | 0.2 | 7.3 |

**S9b Table. Treatment Ranking. Abstinence at the End of Treatment.**

| **Interventions** | **Abbreviation** | **SUCRA (%)** | **PrBest** | **Mean Rank** |
| --- | --- | --- | --- | --- |
| Cognitive behavioural therapy | CBT | 38.8 | 0.0 | 8.3 |
| Contingency management | CM | 76.5 | 5.7 | 3.8 |
| Contingency management plus cognitive behavioural therapy | CM + CBT | 81.6 | 18.7 | 3.2 |
| Contingency management plus community reinforcement approach | CM + CRA | 87.1 | 30.2 | 2.6 |
| Contingency management plus twelve step programme | CM + 12 step | 61.1 | 17.8 | 5.7 |
| Community reinforcement approach | CRA | 69.6 | 17.5 | 4.6 |
| Community reinforcement approach plus non-contingent rewards | CRA + NCR | 43.3 | 3.1 | 7.8 |
| Meditation based treatments | MBT | 47.6 | 5.6 | 7.3 |
| Non-contingent rewards | NCR | 18.6 | 0.0 | 10.8 |
| Supportive-expressive psychodynamic therapy | SEPT | 31.0 | 0.6 | 9.3 |
| Treatment as usual | TAU | 26.9 | 0.0 | 9.8 |
| Twelve step programme | 12 step | 48.3 | 0.3 | 7.2 |
| Twelve step programme plus non-contingent rewards | 12 step + NCR | 19.8 | 0.5 | 10.6 |

**S9c Table. Treatment Ranking. Longest Follow-Up after Study Completion.**

| **Interventions** | **Abbreviation** | **SUCRA (%)** | **PrBest** | **Mean Rank** |
| --- | --- | --- | --- | --- |
| Cognitive behavioural therapy | CBT | 46.0 | 0.0 | 7.5 |
| Contingency management | CM | 50.7 | 0.0 | 6.9 |
| Contingency management plus cognitive behavioural therapy | CM + CBT | 60.6 | 0.5 | 5.7 |
| Contingency management plus community reinforcement approach | CM + CRA | 93.3 | 43.0 | 1.8 |
| Contingency management plus twelve step programme | CM + 12 step | 73.1 | 17.3 | 4.2 |
| Community reinforcement approach | CRA | 88.5 | 28.6 | 2.4 |
| Community reinforcement approach plus non-contingent rewards | CRA + NCR | 50.9 | 1.7 | 6.9 |
| Meditation based treatments | MBT | 47.0 | 8.5 | 7.4 |
| Non-contingent rewards | NCR | 8.0 | 0.0 | 12.0 |
| Supportive-expressive psychodynamic therapy | SEPT | 32.8 | 0.1 | 9.1 |
| Treatment as usual | TAU | 40.3 | 0.0 | 8.2 |
| Twelve step programme | 12 step | 27.4 | 0.0 | 9.7 |
| Twelve step programme plus non-contingent rewards | 12 step + NCR | 31.5 | 0.2 | 9.2 |

**S9d Table. Treatment Ranking. Dropout due to any Cause at 12 Weeks.**

| **Interventions** | **Abbreviation** | **SUCRA (%)** | **PrBest** | **Mean Rank** |
| --- | --- | --- | --- | --- |
| Cognitive behavioural therapy | CBT | 51.0 | 0.0 | 6.9 |
| Contingency management | CM | 48.3 | 0.0 | 7.2 |
| Contingency management plus cognitive behavioural therapy | CM + CBT | 56.5 | 0.5 | 6.2 |
| Contingency management plus community reinforcement approach | CM + CRA | 92.7 | 34.0 | 1.9 |
| Contingency management plus twelve step programme | CM + 12 step | 72.5 | 8.3 | 4.3 |
| Community reinforcement approach | CRA | 47.7 | 1.4 | 7.3 |
| Community reinforcement approach plus non-contingent rewards | CRA + NCR | 93.4 | 53.8 | 1.8 |
| Meditation based treatments | MBT | 18.0 | 0.0 | 10.8 |
| Non-contingent rewards | NCR | 71.0 | 1.7 | 4.5 |
| Supportive-expressive psychodynamic therapy | SEPT | 37.0 | 0.1 | 8.6 |
| Treatment as usual | TAU | 23.1 | 0.0 | 10.2 |
| Twelve step programme | 12-step | 8.7 | 0.0 | 12.0 |
| Twelve step programme plus non-contingent rewards | 12-step + NCR | 30.0 | 0.0 | 9.4 |

**S9e Table. Treatment Ranking. Dropout due to any Cause at the End of Treatment.**

| **Interventions** | **Abbreviation** | **SUCRA (%)** | **PrBest** | **Mean Rank** |
| --- | --- | --- | --- | --- |
| Cognitive behavioural therapy | CBT | 53.8 | 0.1 | 6.5 |
| Contingency management | CM | 49.5 | 0.0 | 7.1 |
| Contingency management plus cognitive behavioural therapy | CM + CBT | 49.0 | 0.2 | 7.1 |
| Contingency management plus community reinforcement approach | CM + CRA | 97.1 | 70.7 | 1.3 |
| Contingency management plus twelve step programme | CM + 12 step | 67.3 | 10.1 | 4.9 |
| Community reinforcement approach | CRA | 86.9 | 16.0 | 2.6 |
| Community reinforcement approach plus non-contingent rewards | CRA + NCR | 56.5 | 0.8 | 6.2 |
| Meditation based treatments | MBT | 18.9 | 0.1 | 10.7 |
| Non-contingent rewards | NCR | 68.0 | 1.2 | 4.8 |
| Supportive-expressive psychodynamic therapy | SEPT | 53.2 | 0.9 | 6.6 |
| Treatment as usual | TAU | 22.2 | 0.0 | 10.3 |
| Twelve step programme | 12 step | 11.1 | 0.0 | 11.7 |
| Twelve step programme plus non-contingent rewards | 12 step + NCR | 16.4 | 0.0 | 11.0 |

| **Interventions*** | **Abbreviation** | **SUCRA (%)** | **PrBest** | **Mean Rank** |
| --- | --- | --- | --- | --- |
| Cognitive behavioural therapy | CBT | 39.6 | 0.0 | 5.8 |
| Contingency management | CM | 72.0 | 13.2 | 3.2 |
| Contingency management plus cognitive behavioural therapy | CM + CBT | 78.1 | 36.8 | 2.7 |
| Contingency management plus community reinforcement approach | CM + CRA | 60.3 | 40.8 | 4.2 |
| Community reinforcement approach | CRA | 41.4 | 2.0 | 5.7 |
| Non-contingent rewards | NCR | 42.9 | 0.0 | 5.6 |
| Treatment as usual | TAU | 33.4 | 0.0 | 6.3 |
| Twelve step programme | 12 step | 35.7 | 0.9 | 6.1 |
| Twelve step programme plus non-contingent rewards | 12 step + NCR | 46.5 | 6.2 | 5.3 |

**S9f Table. Treatment Ranking. Longest Duration of Abstinence at 12 Weeks.**

***** The loop CM + CRA - CRA – 12step + NCR was not included in the SUCRA analysis because disconnected with the main network.

**S9g Table. Treatment Ranking. Longest Duration of Abstinence at the End of Treatment.**

| **Interventions** | **Abbreviation** | **SUCRA (%)** | **PrBest** | **Mean Rank** |
| --- | --- | --- | --- | --- |
| Cognitive behavioural therapy | CBT | 36.1 | 0.0 | 7.4 |
| Contingency management | CM | 77.8 | 6.0 | 3.2 |
| Contingency management plus cognitive behavioural therapy | CM + CBT | 89.0 | 45.7 | 2.1 |
| Contingency management plus community reinforcement approach | CM + CRA | 78.0 | 11.6 | 3.2 |
| Contingency management plus twelve step programme | CM + 12 step | 81.1 | 34.7 | 2.9 |
| Community reinforcement approach | CRA | 13.4 | 0.0 | 9.7 |
| Community reinforcement approach plus non-contingent rewards | CRA + NCR | 40.6 | 1.1 | 6.9 |
| Non-contingent rewards | NCR | 41.1 | 0.0 | 6.9 |
| Treatment as usual | TAU | 24.5 | 0.0 | 8.6 |
| Twelve step programme | 12 step | 30.5 | 0.6 | 8.0 |
| Twelve step programme plus non-contingent rewards | 12 step + NCR | 38.0 | 0.3 | 7.2 |
