# Supplementary material for: Comparative efficacy and acceptability of psychosocial interventions for individuals with cocaine and amphetamine addiction: A systematic review and network meta-analysis
Source: PLoS Med. 2018 Dec 26;15(12):e1002715. doi: 10.1371/journal.pmed.1002715 (PMC6306153; doi:10.1371/journal.pmed.1002715)
Supplement: S10 Table — (DOCX) [file pmed.1002715.s025.docx]

**S10a Table. Subgroup Network Meta-Analyses of each Psychosocial Treatment for Abstinence at the End of Treatment Compared with Treatment as Usual with Odds Ratio (95% CI)^a^.**

| **Characteristics (N/n)^b^** | **CBT** | **CM** | **CM+CBT** | **CM+CRA** | **CM+12 step** | **CRA** | **CRA+NCR** | **MBT** | **NCR** | **SEPT** | **12 step** | **12 step +NCR** |
| --- | --- | --- | --- | --- | --- | --- | --- | --- | --- | --- | --- | --- |
| **All trials** (46/6362) | 1.17  (0.79, 1.74) | **2.22**  **(1.59, 3.10)** | **2.45**  **(1.46, 4.10)** | **2.84**  **(1.24, 6.51)** | 1.82  (0.37, 8.88) | 2.10  (0.67, 6.59) | 1.25  (0.31, 5.02) | 1.37  (0.48, 3.93) | 0.86  (0.52, 1.42) | 1.01  (0.42, 2.46) | 1.35  (0.81, 2.25) | 0.70  (0.15, 3.21) |
| **Sex Ratio^c^** |  |  |  |  |  |  |  |  |  |  |  |  |
| Male to female ratio < 1 (11/2033) | 2.83  (0.98, 8.19) | **2.93**  **(1.88, 4.57)** | 3.29  (0.97, 11.14) | … | … | … | … | … | 1.22  (0.64, 2.32) | … | 1.62  (0.85, 3.07) | … |
| Male to female ratio > 1 (30/3758) | 0.80  (0.48, 1.34) | 1.54  (0.93, 2.53) | 2.28  (0.95, 5.51) | 2.05  (0.50, 8.46) | … | 0.87  (0.33, 2.28) | 0.92  (0.13, 6.43) | 0.60  (0.28, 1.28) | 1.70  (0.85, 3.41) |  | 1.26  (0.63, 2.53) | 0.17  (0.01, 2.28) |
| **Mean Age** |  |  |  |  |  |  |  |  |  |  |  |  |
| < 35 (13/1487) | 0.91  (0.54, 1.54) | 2.51  (0.84, 7.48) | 0.81  (0.25, 2.57) | **2.87**  **(1.29, 6.39)** | 1.92  (0.50, 7.34) | 2.04  (0.75, 5.52) | 1.30  (0.38, 4.42) | … | 1.13  (0.29, 4.45) | 0.91  (0.52, 1.58) | 1.26  (0.73, 2.19) | 0.76  (0.20, 2.92) |
| ≥ 35 (25/4231) | 1.12  (0.64, 1.96) | **2.45**  **(1.60, 3.75)** | **2.79**  **(1.42, 5.47)** | … | … | … | … | 2.05  (0.46, 9.13) | 0.90  (0.48, 1.72) | … | 1.72  (0.72, 4.10) | … |
| **Intensity of the treatment** | |  |  |  |  |  |  |  |  |  |  |  |
| ≤ 1 hour/week (6/436) | … | **4.73**  **(1.18,18.96)** | … | … | … | … | … | … | 0.48  (0.07, 3.49) | … | … | … |
| > 1 hour/week (16/2248) | 0.72  (0.46, 1.12) | **1.67**  **(1.08, 2.59)** | **1.80**  **(1.02, 3.17)** | **2.22**  **(1.05, 4.70)** | … | 1.43  (0.38, 5.29) | 0.89  (0.16, 4.95) | 2.05  (0.64, 6.60) | 0.73  (0.39, 1.37) | 0.85  (0.42, 1.69) | 1.28  (0.65, 2.55) | 0.17  (0.02, 1.86) |
| **Year of publication** | |  |  |  |  |  |  |  |  |  |  |  |
| > 2000 (36/5220) | 1.44  (0.90, 2.33) | **2.38**  **(1.70, 3.32)** | **2.82**  **(1.67, 4.77)** | **2.85**  **(1.28, 6.34)** | 2.33  (0.46, 11.7) | 2.91  (0.90, 9.40) | 1.66  (0.31, 8.93) | 1.50  (0.54, 4.18) | 1.04  (0.63, 1.71) | … | 1.71  (0.84, 3.48) | 1.21  (0.21, 6.85) |
| ≤ 2000 (10/1142) | 0.64  (0.26, 1.57) | 0.33  (0.03, 4.37) | … | … | … | … | … | … | 0.03  (0.00, 0.67) | 0.67  (0.20, 2.19) | 0.71  (0.26, 1.97) | … |
| **Type of stimulant** |  |  |  |  |  |  |  |  |  |  |  |  |
| Cocaine (34/3787) | 1.11  (0.73, 1.67) | **2.48**  **(1.65, 3.73)** | **2.43**  **(1.40, 4.22)** | **2.93**  **(1.39, 6.19)** | 1.91  (0.45, 8.12) | 2.15  (0.75, 6.17) | 1.31  (0.36, 4.69) | 2.05  (0.60, 7.07) | 0.84  (0.49, 1.44) | 0.97  (0.46, 2.04) | 1.24  (0.74, 2.11) | 0.74  (0.18, 3.06) |
| Amphetamines (4/511) | 1.41  (0.03, 63.4) | 2.05  (0.20, 20.9) | 1.64  (0.03, 76.8) | … | … | … | … | 0.94  (0.01,131.2) | … | … | … | … |
| **Characteristics (N/n)^b^** | **CBT** | **CM** | **CM+CBT** | **CM+CRA** | **CM+12 step** | **CRA** | **CRA+NCR** | **MBT** | **NCR** | **SEPT** | **12 step** | **12 step +NCR** |
| Cocaine + Amphetamines (8/2064) | 1.19  (0.28, 5.11) | 2.31  (0.88, 6.02) | 4.25  (0.65, 27.73) | … | … | … | … | … | 1.20  (0.25, 5.83) | … | 1.62  (0.29, 8.87) | … |
| **Risk of bias** |  |  |  |  |  |  |  |  |  |  |  |  |
| Low and moderate risk of bias (33/4983) | 1.18  (0.75, 1.86) | **2.18**  **(1.42, 3.34)** | **2.41**  **(1.31, 4.42)** | 2.55  (0.98, 6.64) | … | 0.54  (0.04, 7.82) | 1.02  (0.14, 7.59) | … | 0.94  (0.48, 1.82) | 1.02  (0.39, 2.68) | 1.35  (0.77, 2.36) | … |
| High risk of bias (13/1731) | 1.19  (0.35, 3.97) | **2.32**  **(1.21, 4.45)** | 2.45  (0.67, 9.00) | 3.56  (0.29, 44.30) | 2.34  (0.13, 42.2) | 3.06  (0.38, 24.6) | 1.67  (0.09, 31.30) | 1.37  (0.42, 4.43) | 0.72  (0.27, 1.89) | … | … | 0.89  (0.05, 15.32) |
| **Individuals on opioid therapy** | |  |  |  |  |  |  |  |  |  |  |  |
| < 50% (13/1251) | 1.06  (0.32, 3.48) | 1.45  (0.54, 3.92) | 1.99  (0.50, 7.89) | 2.05  (0.66, 6.38) | 1.29  (0.20, 8.41) | 1.20  (0.21, 7.00) | 0.89  (0.17, 4.70) | 2.05  (0.44, 9.58) | … | … | 0.63  (0.10, 3.80) | 0.49  (0.08, 2.92) |
| ≥ 50% (15/1679) | 1.42  (0.80, 2.54) | **2.65**  **(1.67, 4.22)** | **2.69**  **(1.34, 5.40)** | … | … | … | … | … | 0.88  (0.48, 1.60) | … | 1.88  (0.79, 4.43) | … |
| **Sample size** |  |  |  |  |  |  |  |  |  |  |  |  |
| ≥ 100 participants (26/5035) | 1.37  (0.87, 2.16) | **2.27**  **(1.56, 3.30)** | **2.88**  **(1.64, 5.07)** | 3.11  (0.92, 10.54) | 2.54  (0.39, 16.5) | … | 1.82  (0.26, 12.49) | 0.92  (0.18, 4.72) | 1.00  (0.59, 1.68) | 1.10  (0.47, 2.59) | 1.50  (0.89, 2.52) | 1.32  (0.18, 9.51) |
| < 100 participants (20/1327) | **0.34**  **(0.15, 0.76)** | **2.81**  **(1.34, 5.91)** | 0.51  (0.16, 1.61) | 1.90  (0.74, 4.90) | … | 1.09  (0.34, 3.46) | 0.76  (0.12, 4.68) | 2.05  (0.64, 6.63) | 0.29  (0.06, 1.35) | … | 0.31  (0.08, 1.11) | 0.15  (0.01, 1.71) |
| **Comorbid alcohol abuse** | |  |  |  |  |  |  |  |  |  |  |  |
| < 50% (16/2886) | 1.15  (0.51, 2.59) | **2.99**  **(1.35, 6.59)** | **3.66**  **(1.12, 11.93)** | … | … | … | … | … | 1.17  (0.44, 3.11) | … | 1.62  (0.42, 6.15) | … |
| ≥ 50 % (13/1128) | 1.26  (0.26, 6.08) | 2.27  (0.94, 5.50) | 3.15  (0.36, 27.53) | **2.95**  **(1.03, 8.44)** | … | 2.16  (0.50, 9.32) | 1.18  (0.14, 9.90) | … | … | … | 1.47  (0.42, 5.19) | 0.23  (0.02, 3.37) |

a An Odds ratio above 1 indicates the superiority of the specific psychosocial treatment against the treatment as usual.

b N= number of studies; n= number of patients.

c We excluded the trials with male-to-female ratio= 1.

**S10b Table. Subgroup Network Meta-Analyses of each Psychosocial Treatment for Dropout due to any Cause at the End of Treatment Compared with Treatment as Usual with Odds Ratio (95% CI)*.**

| **Characteristics (N/n)^b^** | **CBT** | **CM** | **CM+CBT** | **CM+CRA** | **CM+12 step** | **CRA** | **CRA+NCR** | **MBT** | **NCR** | **SEPT** | **12 step** | **12 step +NCR** |  |
| --- | --- | --- | --- | --- | --- | --- | --- | --- | --- | --- | --- | --- | --- |
| **All trials** (43/5737) | **0.68**  **(0.50, 0.92)** | **0.71**  **(0.55, 0.91)** | 0.71  (0.45, 1.13) | **0.28**  **(0.15, 0.50)** | 0.51  (0.16, 1.58) | **0.36**  **(0.18, 0.73)** | 0.62  (0.24, 1.60) | 1.14  (0.56, 2.35) | **0.57**  **(0.37, 0.86)** | 0.68  (0.38, 1.22) | 1.24  (0.83, 1.83) | 1.27  (0.44, 3.65) |  |
| **Sex Ratio^a^** |  |  |  |  |  |  |  |  |  |  |  |  |  |
| Male to female ratio < 1 (9/1918) | 0.70  (0.25, 1.99) | 0.74  (0.42, 1.30) | … | … | … | … | … | … | 0.89  (0.31, 2.53) | … | 1.43  (0.69, 2.99) | … |  |
| Male to female ratio > 1 (30/3796) | **0.65**  **(0.45, 0.94)** | 0.74  (0.53, 1.02) | 0.64  (0.37, 1.10) | **0.28**  **(0.15, 0.51)** | … | **0.36**  **(0.18, 0.74)** | 0.31  (0.09, 1.03) | 1.66  (0.57, 4.82) | **0.46**  **(0.27, 0.81)** | 0.64  (0.35, 1.20) | 1.09  (0.64, 1.87) | 3.25  (0.49, 21.35) |  |
| **Mean Age** |  |  |  |  |  |  |  |  |  |  |  |  |  |
| < 35 (15/1715) | 0.69  (0.31, 1.54) | 0.56  (0.19, 1.64) | 0.57  (0.10, 3.15) | **0.25**  **(0.09, 0.64)** | 0.48  (0.09, 2.46) | 0.37  (0.12, 1.09) | 0.58  (0.14, 2.31) | … | 0.52  (0.09, 2.88) | 0.61  (0.20, 1.87) | 0.88  (0.30, 2.56) | 1.26  (0.28, 5.61) |  |
| ≥ 35 (23/3506) | **0.61**  **(0.38, 0.98)** | 0.73  (0.53, 1.00) | 0.64  (0.35, 1.17) | … | … | … | … | 1.66  (0.57, 4.83) | **0.53**  **(0.31, 0.91)** | … | 1.48  (0.88, 2.49) | … |  |
| **Intensity of the treatment** | |  |  |  |  |  |  |  |  |  |  |  |  |
| ≤ 1 hour/week (6/436) | … | 0.74  (0.20, 2.66) | … | … | … | … | … | … | 1.33  (0.24, 7.48) | … | … | … |  |
| > 1 hour/week (12/1535) | **0.53**  **(0.29, 0.97)** | **0.54**  **(0.31, 0.94)** | 0.69  (0.31, 1.52) | **0.22**  **(0.10, 0.45)** | … | 0.52  (0.16, 1.69) | **0.24**  **(0.07, 0.88)** | 1.66  (0.56, 4.90) | 0.62  (0.22, 1.70) | 0.57  (0.28, 1.16) | 0.94  (0.45, 1.95) | 2.54  (0.37, 17.65) |  |
| **Year of publication** | |  |  |  |  |  |  |  |  |  |  |  |  |
| > 2000 (34/4711) | **0.61**  **(0.41, 0.89)** | **0.70**  **(0.54, 0.90)** | 0.67  (0.42, 1.08) | **0.29**  **(0.16, 0.52)** | 0.68  (0.20, 2.37) | **0.29**  **(0.14, 0.60)** | 1.19  (0.34, 4.15) | 1.07  (0.52, 2.22) | **0.52**  **(0.33, 0.79)** | … | 1.48  (0.90, 2.41) | 1.47  (0.42, 5.10) |  |
| ≤ 2000 (9/1026) | 0.69  (0.44, 1.09) | 0.72  (0.25, 2.02) | … | … | … | … | … | … | 1.29  (0.27, 6.11) | 0.64  (0.38, 1.09) | 1.00  (0.58, 1.75) | … |  |
| **Type of stimulant** |  |  |  |  |  |  |  |  |  |  |  |  |  |
| Cocaine (32/3324) | 0.69  (0.48, 1.01) | 0.75  (0.49, 1.14) | 0.79  (0.44, 1.42) | **0.28**  **(0.15, 0.52)** | 0.52  (0.16, 1.65) | **0.37**  **(0.18, 0.76)** | 0.64  (0.24, 1.66) | 1.66  (0.58, 4.77) | 0.78  (0.44, 1.38) | 0.66  (0.36, 1.23) | 1.12  (0.66, 1.90) | 1.30  (0.45, 3.81) |  |
| Amphetamines (3/349) | … | 0.58  (0.19, 1.82) | … | … | … | … | … | … | … | … | … | … |  |
| **Characteristics (N/n)^b^** | **CBT** | **CM** | **CM+CBT** | **CM+CRA** | **CM+12 step** | **CRA** | **CRA+NCR** | **MBT** | **NCR** | **SEPT** | **12 step** | **12 step +NCR** |  |
| Cocaine + Amphetamines (8/2064) | 0.58  (0.32, 1.02) | **0.69**  **(0.52, 0.92)** | 0.60  (0.26, 1.40) | … | … | … | … | … | **0.28**  **(0.15, 0.53)** | … | 1.43  (0.90, 2.29) | … |  |
| **Risk of bias** |  |  |  |  |  |  |  |  |  |  |  |  |  |
| Low and moderate risk of bias (29/3904) | **0.65**  **(0.46, 0.93)** | **0.70**  **(0.51, 0.97)** | 0.61  (0.36, 1.04) | **0.25**  **(0.13, 0.48)** | … | 1.11  (0.24, 5.21) | **0.28**  **(0.08, 0.91)** | … | **0.54**  **(0.31, 0.92)** | 0.67  (0.38, 1.17) | 1.23  (0.85, 1.79) | … |  |
| High risk of bias (14/1833) | 0.84  (0.46, 1.53) | 0.70  (0.46, 1.07) | 1.18  (0.48, 2.92) | 0.67  (0.17, 2.61) | 1.82  (0.33, 10.1) | 0.57  (0.18, 1.85) | 3.17  (0.57, 17.57) | 1.29  (0.60, 2.76) | 0.69  (0.34, 1.37) | … | … | 4.30  (0.83, 22.23) |  |
| **Individuals on opioid therapy** | |  |  |  |  |  |  |  |  |  |  |  |  |
| < 50% (13/1241) | 0.61  (0.25, 1.49) | 0.75  (0.33, 1.67) | 0.59  (0.19, 1.83) | **0.26**  **(0.10, 0.65)** | 0.49  (0.11, 2.25) | 0.34  (0.10, 1.17) | 0.59  (0.16, 2.19) | 1.66  (0.45, 6.11) | … | … | … | 1.27  (0.31, 5.24) |  |
| ≥ 50% (15/1689) | 0.76  (0.44, 1.31) | 0.91  (0.65, 1.27) | 1.40  (0.63, 3.15) | … | … | … | … | … | 0.97  (0.60, 1.58) | … | 1.60  (0.68, 3.76) | … |  |
| **Sample size** |  |  |  |  |  |  |  |  |  |  |  |  |  |
| ≥ 100 participants (24/4508) | **0.66**  **(0.47, 0.91)** | **0.73**  **(0.57, 0.95)** | 0.72  (0.45, 1.16) | **0.20**  **(0.08, 0.49)** | 0.47  (0.11, 1.90) | **0.12**  **(0.04, 0.41)** | 0.81  (0.20, 3.33) | 0.82  (0.32, 2.12) | **0.54**  **(0.36, 0.83)** | 0.68  (0.40, 1.16) | 1.29  (0.90, 1.85) | 1.00  (0.24, 4.09) |  |
| < 100 participants (19/1229) | 0.80  (0.33, 1.96) | **0.49**  **(0.28, 0.86)** | 0.79  (0.21, 2.99) | **0.30**  **(0.14, 0.63)** | 0.63  (0.25, 1.58) | 0.33  (0.10, 1.10) | 1.66  (0.63, 4.38) | 0.88  (0.24, 3.20) | 0.80  (0.33, 1.96) | … | 0.47  (0.06, 3.62) | 3.46  (0.52, 22.75) |  |
| **Comorbid alcohol abuse** | |  |  |  |  |  |  |  |  |  |  |  |  |
| < 50% (15/2970) | 0.63  (0.36, 1.11) | 0.80  (0.53, 1.20) | 0.83  (0.41, 1.68) | … | … | … | … | … | 0.67  (0.38, 1.17) | … | 1.43  (0.72, 2.85) | … |  |
| ≥ 50 % (12/1044) | 1.26  (0.25, 6.28) | 0.51  (0.22, 1.18) | 3.11  (0.14, 68.71) | **0.23**  **(0.09, 0.62)** | … | 0.68  (0.18, 2.62) | 0.26  (0.05, 1.44) | … | … | … | 1.28  (0.37, 4.37) | 2.74  (0.29, 25.68) |  |

a An Odds ratio above 1 indicates the superiority of the specific psychosocial treatment against the treatment as usual.

b N= number of studies; n= number of patients.

c We excluded the trials with male-to-female ratio= 1.
