# Supplementary material for: Comparative efficacy and acceptability of psychosocial interventions for individuals with cocaine and amphetamine addiction: A systematic review and network meta-analysis
Source: PLoS Med. 2018 Dec 26;15(12):e1002715. doi: 10.1371/journal.pmed.1002715 (PMC6306153; doi:10.1371/journal.pmed.1002715)
Supplement: S11 Table — (DOCX) [file pmed.1002715.s026.docx]

**S11a Table. Predictivity Intervals of Mixed Estimates for Abstinence at the End of Treatment.**

| **Comparison** | **OR** | **SE** | **Low**  **95% CI** | **Upper**  **95% CI** | **Low**  **95% PrI** | **Upper**  **95% PrI** |
| --- | --- | --- | --- | --- | --- | --- |
| NCR vs TAU | 0,86 | 1,29 | 0,52 | 1,42 | 0,28 | 2,60 |
| CM vs TAU | 2,22 | 1,19 | 1,59 | 3,10 | 0,79 | 6,24 |
| CBT vs TAU | 1,17 | 1,22 | 0,79 | 1,74 | 0,41 | 3,37 |
| CRA vs TAU | 2,10 | 1,79 | 0,67 | 6,59 | 0,44 | 10,02 |
| 12-step vs TAU | 1,35 | 1,30 | 0,81 | 2,25 | 0,44 | 4,11 |
| SEPT vs TAU | 1,01 | 1,57 | 0,42 | 2,46 | 0,26 | 3,94 |
| CM+CBT vs TAU | 2,45 | 1,30 | 1,46 | 4,10 | 0,80 | 7,47 |
| CM+CRA vs TAU | 2,84 | 1,53 | 1,24 | 6,51 | 0,76 | 10,58 |
| MBT vs TAU | 1,37 | 1,71 | 0,48 | 3,93 | 0,31 | 6,07 |
| CRA+NCR vs TAU | 1,25 | 2,03 | 0,31 | 5,02 | 0,21 | 7,40 |
| CM+12-step vs TAU | 1,82 | 2,24 | 0,37 | 8,88 | 0,26 | 12,87 |
| 12-step+NCR vs TAU | 0,70 | 2,18 | 0,15 | 3,21 | 0,10 | 4,67 |
| CM vs NCR | 2,59 | 1,24 | 1,70 | 3,93 | 0,89 | 7,52 |
| CBT vs NCR | 1,37 | 1,33 | 0,78 | 2,40 | 0,44 | 4,28 |
| CRA vs NCR | 2,45 | 1,85 | 0,73 | 8,20 | 0,49 | 12,34 |
| 12-step vs NCR | 1,57 | 1,42 | 0,79 | 3,13 | 0,47 | 5,31 |
| SEPT vs NCR | 1,18 | 1,66 | 0,43 | 3,20 | 0,28 | 5,00 |
| CM+CBT vs NCR | 2,85 | 1,36 | 1,55 | 5,24 | 0,89 | 9,17 |
| CM+CRA vs NCR | 3,31 | 1,60 | 1,32 | 8,28 | 0,83 | 13,16 |
| MBT vs NCR | 1,59 | 1,80 | 0,50 | 5,05 | 0,33 | 7,66 |
| CRA+NCR vs NCR | 1,46 | 2,09 | 0,34 | 6,18 | 0,23 | 9,05 |
| CM+12-step vs NCR | 2,12 | 2,30 | 0,42 | 10,85 | 0,29 | 15,68 |
| 12-step+NCR vs NCR | 0,81 | 2,23 | 0,17 | 3,93 | 0,12 | 5,70 |
| CBT vs CM | 0,53 | 1,24 | 0,35 | 0,81 | 0,18 | 1,54 |
| CRA vs CM | 0,95 | 1,79 | 0,30 | 2,98 | 0,20 | 4,53 |
| 12-step vs CM | 0,61 | 1,34 | 0,34 | 1,08 | 0,19 | 1,92 |
| SEPT vs CM | 0,46 | 1,60 | 0,18 | 1,15 | 0,11 | 1,83 |
| CM+CBT vs CM | 1,10 | 1,28 | 0,68 | 1,79 | 0,37 | 3,31 |
| CM+CRA vs CM | 1,28 | 1,52 | 0,56 | 2,92 | 0,34 | 4,76 |
| MBT vs CM | 0,62 | 1,75 | 0,21 | 1,84 | 0,13 | 2,81 |
| CRA+NCR vs CM | 0,56 | 2,03 | 0,14 | 2,26 | 0,10 | 3,33 |
| CM+12-step vs CM | 0,82 | 2,24 | 0,17 | 4,00 | 0,12 | 5,80 |
| 12-step+NCR vs CM | 0,31 | 2,18 | 0,07 | 1,45 | 0,05 | 2,11 |
| CRA vs CBT | 1,79 | 1,79 | 0,57 | 5,60 | 0,38 | 8,52 |
| 12-step vs CBT | 1,15 | 1,31 | 0,68 | 1,94 | 0,38 | 3,53 |
| SEPT vs CBT | 0,86 | 1,58 | 0,35 | 2,10 | 0,22 | 3,37 |
| CM+CBT vs CBT | 2,09 | 1,28 | 1,29 | 3,38 | 0,70 | 6,26 |
| CM+CRA vs CBT | 2,43 | 1,56 | 1,02 | 5,78 | 0,63 | 9,29 |
| MBT vs CBT | 1,17 | 1,72 | 0,40 | 3,39 | 0,26 | 5,23 |
| CRA+NCR vs CBT | 1,07 | 2,06 | 0,26 | 4,39 | 0,18 | 6,45 |
| CM+12-step vs CBT | 1,55 | 2,27 | 0,31 | 7,74 | 0,22 | 11,20 |
| 12-step+NCR vs CBT | 0,60 | 2,20 | 0,13 | 2,80 | 0,09 | 4,07 |
| 12-step vs CRA | 0,64 | 1,86 | 0,19 | 2,18 | 0,13 | 3,27 |
| SEPT vs CRA | 0,48 | 2,06 | 0,12 | 1,99 | 0,08 | 2,93 |
| CM+CBT vs CRA | 1,16 | 1,85 | 0,35 | 3,87 | 0,23 | 5,84 |
| **Comparison** | **OR** | **SE** | **Low**  **95% CI** | **Upper**  **95% CI** | **Low**  **95% PrI** | **Upper**  **95% PrI** |
| CM+CRA vs CRA | 1,35 | 1,69 | 0,48 | 3,80 | 0,31 | 5,89 |
| MBT vs CRA | 0,65 | 2,19 | 0,14 | 3,01 | 0,10 | 4,38 |
| CRA+NCR vs CRA | 0,59 | 2,17 | 0,13 | 2,72 | 0,09 | 3,96 |
| CM+12-step vs CRA | 0,87 | 2,38 | 0,16 | 4,74 | 0,11 | 6,82 |
| 12-step+NCR vs CRA | 0,33 | 2,31 | 0,06 | 1,72 | 0,04 | 2,48 |
| SEPT vs 12-step | 0,75 | 1,59 | 0,30 | 1,86 | 0,19 | 2,96 |
| CM+CBT vs 12-step | 1,81 | 1,40 | 0,93 | 3,52 | 0,54 | 6,03 |
| CM+CRA vs 12-step | 2,11 | 1,63 | 0,81 | 5,46 | 0,52 | 8,60 |
| MBT vs 12-step | 1,01 | 1,80 | 0,32 | 3,19 | 0,21 | 4,84 |
| CRA+NCR vs 12-step | 0,92 | 2,12 | 0,21 | 4,02 | 0,15 | 5,88 |
| CM+12-step vs 12-step | 1,35 | 2,32 | 0,26 | 7,04 | 0,18 | 10,16 |
| 12-step+NCR vs 12-step | 0,52 | 2,26 | 0,10 | 2,55 | 0,07 | 3,70 |
| CM+CBT vs SEPT | 2,42 | 1,65 | 0,91 | 6,44 | 0,58 | 10,10 |
| CM+CRA vs SEPT | 2,81 | 1,84 | 0,85 | 9,29 | 0,56 | 14,01 |
| MBT vs SEPT | 1,35 | 2,00 | 0,35 | 5,24 | 0,24 | 7,75 |
| CRA+NCR vs SEPT | 1,23 | 2,31 | 0,24 | 6,34 | 0,17 | 9,16 |
| CM+12-step vs SEPT | 1,80 | 2,51 | 0,30 | 10,93 | 0,21 | 15,65 |
| 12-step+NCR vs SEPT | 0,69 | 2,45 | 0,12 | 3,98 | 0,08 | 5,71 |
| CM+CRA vs CM+CBT | 1,16 | 1,60 | 0,46 | 2,93 | 0,29 | 4,65 |
| MBT vs CM+CBT | 0,56 | 1,79 | 0,18 | 1,75 | 0,12 | 2,66 |
| CRA+NCR vs CM+CBT | 0,51 | 2,10 | 0,12 | 2,18 | 0,08 | 3,19 |
| CM+12-step vs CM+CBT | 0,74 | 2,31 | 0,14 | 3,83 | 0,10 | 5,53 |
| 12-step+NCR vs CM+CBT | 0,29 | 2,24 | 0,06 | 1,39 | 0,04 | 2,01 |
| MBT vs CM+CRA | 0,48 | 1,97 | 0,13 | 1,82 | 0,09 | 2,69 |
| CRA+NCR vs CM+CRA | 0,44 | 1,77 | 0,14 | 1,35 | 0,09 | 2,05 |
| CM+12-step vs CM+CRA | 0,64 | 1,99 | 0,17 | 2,48 | 0,11 | 3,66 |
| 12-step+NCR vs CM+CRA | 0,25 | 1,92 | 0,07 | 0,89 | 0,05 | 1,32 |
| CRA+NCR vs MBT | 0,91 | 2,43 | 0,16 | 5,19 | 0,11 | 7,45 |
| CM+12-step vs MBT | 1,33 | 2,63 | 0,20 | 8,86 | 0,14 | 12,65 |
| 12-step+NCR vs MBT | 0,51 | 2,56 | 0,08 | 3,24 | 0,06 | 4,62 |
| CM+12-step vs CRA+NCR | 1,46 | 2,08 | 0,35 | 6,12 | 0,24 | 8,98 |
| 12-step+NCR vs CRA+NCR | 0,56 | 2,09 | 0,13 | 2,37 | 0,09 | 3,47 |
| 12-step+NCR vs CM+12-step | 0,38 | 2,17 | 0,08 | 1,74 | 0,06 | 2,54 |

**S11b Table. Predictivity Intervals of Mixed Estimates for Dropout at the End of Treatment**

| **Comparison** | **OR** | **SE** | **Low**  **95% CI** | **Upper**  **95% CI** | **Low**  **95% PrI** | **Upper**  **95% PrI** |
| --- | --- | --- | --- | --- | --- | --- |
| NCR vs TAU | 0,57 | 1,24 | 0,37 | 0,86 | 0,30 | 1,07 |
| CM vs TAU | 0,71 | 1,14 | 0,55 | 0,91 | 0,42 | 1,19 |
| CBT vs TAU | 0,68 | 1,17 | 0,50 | 0,92 | 0,39 | 1,19 |
| CRA vs TAU | 0,36 | 1,43 | 0,18 | 0,73 | 0,15 | 0,87 |
| 12-step vs TAU | 1,24 | 1,22 | 0,83 | 1,83 | 0,67 | 2,29 |
| SEPT vs TAU | 0,68 | 1,35 | 0,38 | 1,22 | 0,31 | 1,47 |
| CM+CBT vs TAU | 0,71 | 1,27 | 0,45 | 1,13 | 0,36 | 1,39 |
| CM+CRA vs TAU | 0,28 | 1,35 | 0,15 | 0,50 | 0,13 | 0,60 |
| MBT vs TAU | 1,14 | 1,45 | 0,56 | 2,35 | 0,47 | 2,81 |
| CRA+NCR vs TAU | 0,62 | 1,62 | 0,24 | 1,60 | 0,20 | 1,89 |
| CM+12-step vs TAU | 0,51 | 1,79 | 0,16 | 1,58 | 0,14 | 1,87 |
| 12-step+NCR vs TAU | 1,27 | 1,71 | 0,44 | 3,65 | 0,37 | 4,31 |
| CM vs NCR | 1,25 | 1,19 | 0,88 | 1,76 | 0,70 | 2,23 |
| CBT vs NCR | 1,20 | 1,27 | 0,75 | 1,92 | 0,61 | 2,36 |
| CRA vs NCR | 0,64 | 1,49 | 0,29 | 1,39 | 0,25 | 1,65 |
| 12-step vs NCR | 2,18 | 1,34 | 1,23 | 3,84 | 1,02 | 4,64 |
| SEPT vs NCR | 1,20 | 1,43 | 0,59 | 2,43 | 0,50 | 2,90 |
| CM+CBT vs NCR | 1,25 | 1,31 | 0,74 | 2,12 | 0,61 | 2,58 |
| CM+CRA vs NCR | 0,49 | 1,42 | 0,24 | 0,96 | 0,20 | 1,15 |
| MBT vs NCR | 2,02 | 1,52 | 0,89 | 4,57 | 0,75 | 5,41 |
| CRA+NCR vs NCR | 1,09 | 1,67 | 0,40 | 2,99 | 0,34 | 3,53 |
| CM+12-step vs NCR | 0,89 | 1,83 | 0,27 | 2,94 | 0,23 | 3,47 |
| 12-step+NCR vs NCR | 2,24 | 1,76 | 0,74 | 6,79 | 0,63 | 8,01 |
| CBT vs CM | 0,96 | 1,19 | 0,68 | 1,36 | 0,54 | 1,72 |
| CRA vs CM | 0,51 | 1,44 | 0,25 | 1,04 | 0,21 | 1,24 |
| 12-step vs CM | 1,75 | 1,26 | 1,11 | 2,75 | 0,90 | 3,39 |
| SEPT vs CM | 0,96 | 1,38 | 0,51 | 1,80 | 0,43 | 2,16 |
| CM+CBT vs CM | 1,01 | 1,25 | 0,65 | 1,55 | 0,53 | 1,92 |
| CM+CRA vs CM | 0,39 | 1,36 | 0,21 | 0,71 | 0,18 | 0,85 |
| MBT vs CM | 1,62 | 1,47 | 0,76 | 3,43 | 0,64 | 4,08 |
| CRA+NCR vs CM | 0,88 | 1,62 | 0,34 | 2,27 | 0,29 | 2,68 |
| CM+12-step vs CM | 0,72 | 1,79 | 0,23 | 2,25 | 0,19 | 2,65 |
| 12-step+NCR vs CM | 1,80 | 1,72 | 0,62 | 5,18 | 0,53 | 6,11 |
| CRA vs CBT | 0,53 | 1,43 | 0,26 | 1,07 | 0,22 | 1,28 |
| 12-step vs CBT | 1,82 | 1,26 | 1,16 | 2,85 | 0,94 | 3,52 |
| SEPT vs CBT | 1,00 | 1,36 | 0,55 | 1,82 | 0,46 | 2,19 |
| CM+CBT vs CBT | 1,05 | 1,27 | 0,66 | 1,67 | 0,53 | 2,05 |
| CM+CRA vs CBT | 0,41 | 1,37 | 0,22 | 0,76 | 0,18 | 0,91 |
| MBT vs CBT | 1,68 | 1,44 | 0,82 | 3,44 | 0,69 | 4,10 |
| CRA+NCR vs CBT | 0,91 | 1,64 | 0,35 | 2,40 | 0,29 | 2,83 |
| CM+12-step vs CBT | 0,75 | 1,80 | 0,24 | 2,37 | 0,20 | 2,80 |
| 12-step+NCR vs CBT | 1,87 | 1,73 | 0,64 | 5,46 | 0,54 | 6,45 |
| 12-step vs CRA | 3,42 | 1,50 | 1,55 | 7,55 | 1,31 | 8,96 |
| SEPT vs CRA | 1,88 | 1,58 | 0,77 | 4,59 | 0,65 | 5,43 |
| CM+CBT vs CRA | 1,97 | 1,50 | 0,89 | 4,37 | 0,75 | 5,19 |
| **Comparison** | **OR** | **SE** | **Low**  **95% CI** | **Upper**  **95% CI** | **Low**  **95% PrI** | **Upper**  **95% PrI** |
| CM+CRA vs CRA | 0,76 | 1,33 | 0,43 | 1,34 | 0,36 | 1,63 |
| MBT vs CRA | 3,17 | 1,65 | 1,19 | 8,43 | 1,01 | 9,95 |
| CRA+NCR vs CRA | 1,72 | 1,60 | 0,68 | 4,34 | 0,58 | 5,13 |
| CM+12-step vs CRA | 1,40 | 1,77 | 0,46 | 4,32 | 0,39 | 5,10 |
| 12-step+NCR vs CRA | 3,52 | 1,70 | 1,25 | 9,92 | 1,06 | 11,71 |
| SEPT vs 12-step | 0,55 | 1,37 | 0,30 | 1,02 | 0,25 | 1,23 |
| CM+CBT vs 12-step | 0,58 | 1,35 | 0,32 | 1,04 | 0,26 | 1,26 |
| CM+CRA vs 12-step | 0,22 | 1,43 | 0,11 | 0,45 | 0,09 | 0,54 |
| MBT vs 12-step | 0,93 | 1,51 | 0,41 | 2,07 | 0,35 | 2,46 |
| CRA+NCR vs 12-step | 0,50 | 1,68 | 0,18 | 1,39 | 0,15 | 1,64 |
| CM+12-step vs 12-step | 0,41 | 1,84 | 0,12 | 1,36 | 0,10 | 1,61 |
| 12-step+NCR vs 12-step | 1,03 | 1,77 | 0,34 | 3,16 | 0,28 | 3,73 |
| CM+CBT vs SEPT | 1,05 | 1,44 | 0,51 | 2,16 | 0,43 | 2,57 |
| CM+CRA vs SEPT | 0,41 | 1,52 | 0,18 | 0,92 | 0,15 | 1,09 |
| MBT vs SEPT | 1,68 | 1,59 | 0,68 | 4,16 | 0,58 | 4,92 |
| CRA+NCR vs SEPT | 0,91 | 1,75 | 0,30 | 2,75 | 0,26 | 3,24 |
| CM+12-step vs SEPT | 0,75 | 1,91 | 0,21 | 2,66 | 0,18 | 3,15 |
| 12-step+NCR vs SEPT | 1,87 | 1,84 | 0,56 | 6,19 | 0,48 | 7,32 |
| CM+CRA vs CM+CBT | 0,39 | 1,44 | 0,19 | 0,79 | 0,16 | 0,94 |
| MBT vs CM+CBT | 1,61 | 1,52 | 0,70 | 3,67 | 0,59 | 4,35 |
| CRA+NCR vs CM+CBT | 0,87 | 1,69 | 0,31 | 2,43 | 0,26 | 2,87 |
| CM+12-step vs CM+CBT | 0,71 | 1,85 | 0,21 | 2,38 | 0,18 | 2,81 |
| 12-step+NCR vs CM+CBT | 1,78 | 1,78 | 0,58 | 5,50 | 0,49 | 6,50 |
| MBT vs CM+CRA | 4,15 | 1,60 | 1,66 | 10,36 | 1,40 | 12,25 |
| CRA+NCR vs CM+CRA | 2,25 | 1,46 | 1,08 | 4,71 | 0,90 | 5,60 |
| CM+12-step vs CM+CRA | 1,84 | 1,64 | 0,69 | 4,87 | 0,59 | 5,75 |
| 12-step+NCR vs CM+CRA | 4,61 | 1,56 | 1,92 | 11,06 | 1,62 | 13,08 |
| CRA+NCR vs MBT | 0,54 | 1,82 | 0,17 | 1,76 | 0,14 | 2,08 |
| CM+12-step vs MBT | 0,44 | 1,98 | 0,12 | 1,69 | 0,10 | 2,00 |
| 12-step+NCR vs MBT | 1,11 | 1,91 | 0,31 | 3,94 | 0,26 | 4,66 |
| CM+12-step vs CRA+NCR | 0,82 | 1,63 | 0,31 | 2,13 | 0,27 | 2,52 |
| 12-step+NCR vs CRA+NCR | 2,05 | 1,58 | 0,83 | 5,05 | 0,70 | 5,97 |
| 12-step+NCR vs CM+12-step | 2,51 | 1,65 | 0,94 | 6,72 | 0,79 | 7,93 |
