# Supplementary material for: Comparative efficacy and acceptability of psychosocial interventions for individuals with cocaine and amphetamine addiction: A systematic review and network meta-analysis
Source: PLoS Med. 2018 Dec 26;15(12):e1002715. doi: 10.1371/journal.pmed.1002715 (PMC6306153; doi:10.1371/journal.pmed.1002715)
Supplement: S12 Table — (DOCX) [file pmed.1002715.s027.docx]

**S12a Table. Evaluation of the Quality of Evidence Using GRADE Framework for Abstinence at the End of Treatment.**

| **Comparison** | | | | **Study Limitations** | | | **Imprecision** | | | **Heterogeneity** | | **Incoherence** | | | **Indirectness** | | | **Publication bias** | | **Confidence in OR for abstinence** |
| --- | --- | --- | --- | --- | --- | --- | --- | --- | --- | --- | --- | --- | --- | --- | --- | --- | --- | --- | --- | --- |
| **Mixed evidence** | |  | | |  | | |  | | | | |  | | |  | | |  | |
| 12-Step vs CBT | | | | Some concerns | | | Major concerns | | | No concerns | | No concerns | | | No concerns | | | Undetected | | **Very low** |
| 12-Step vs SEPT | | | | No concerns | | | Some concerns | | | No concerns | | No concerns | | | No concerns | | | Undetected | | **Moderate** |
| 12-Step vs TAU | | | | No concerns | | | Some concerns | | | Some concerns | | No concerns | | | No concerns | | | Undetected | | **Low** |
| CBT vs CM | | | | No concerns | | | Some concerns | | | Some concerns | | No concerns | | | No concerns | | | Undetected | | **Low** |
| CBT vs CM+CBT | | | | No concerns | | | No concerns | | | Major concerns | | No concerns | | | No concerns | | | Undetected | | **Low** |
| CBT vs CRA | | | | Major concerns | | | Some concerns | | | No concerns | | No concerns | | | No concerns | | | Undetected | | **Very low** |
| CBT vs MBT | | | | Major concerns | | | Some concerns | | | No concerns | | No concerns | | | No concerns | | | Undetected | | **Very low** |
| CBT vs NCR | | | | No concerns | | | Some concerns | | | No concerns | | No concerns | | | No concerns | | | Undetected | | **Moderate** |
| CBT vs SEPT | | | | No concerns | | | Some concerns | | | No concerns | | No concerns | | | No concerns | | | Undetected | | **Moderate** |
| CBT vs TAU | | | | Some concerns | | | Some concerns | | | No concerns | | No concerns | | | No concerns | | | Undetected | | **Low** |
| CM vs CM+CBT | | | | Some concerns | | | Some concerns | | | No concerns | | No concerns | | | No concerns | | | Undetected | | **Low** |
| CM vs CM+CRA | | | | Some concerns | | | Some concerns | | | No concerns | | No concerns | | | No concerns | | | Undetected | | **Low** |
| CM vs NCR | | | | No concerns | | | No concerns | | | Some concerns | | No concerns | | | No concerns | | | Undetected | | **Moderate** |
| CM vs TAU | | | | No concerns | | | No concerns | | | Some concerns | | No concerns | | | No concerns | | | Undetected | | **Moderate** |
| CM+12-Step vs CM+CRA | | | | Major concerns | | | Some concerns | | | No concerns | | No concerns | | | No concerns | | | Undetected | | **Very low** |
| CM+12-Step vs NCR+12-Step | | | | Major concerns | | | Some concerns | | | No concerns | | No concerns | | | No concerns | | | Undetected | | **Very low** |
| CM+12-Step vs NCR+CRA | | | | Major concerns | | | Some concerns | | | No concerns | | No concerns | | | No concerns | | | Undetected | | **Very low** |
| CM+CBT vs NCR | | | | No concerns | | | No concerns | | | Some concerns | | No concerns | | | No concerns | | | Undetected | | **Moderate** |
| CM+CBT vs TAU | | | | No concerns | | | No concerns | | | Some concerns | | No concerns | | | No concerns | | | Undetected | | **Moderate** |
| CM+CRA vs CRA | | | | Major concerns | | | Some concerns | | | No concerns | | No concerns | | | No concerns | | | Undetected | | **Very low** |
| CM+CRA vs NCR+12-Step | | | | Major concerns | | | Some concerns | | | No concerns | | No concerns | | | No concerns | | | Undetected | | **Very low** |
| CM+CRA vs NCR+CRA | | | | Major concerns | | | Some concerns | | | No concerns | | No concerns | | | No concerns | | | Undetected | | **Very low** |
| CM+CRA vs TAU | | | | No concerns | | | No concerns | | | Some concerns | | No concerns | | | No concerns | | | Undetected | | **Moderate** |
| MBT vs TAU | | | | Major concerns | | | Some concerns | | | No concerns | | No concerns | | | No concerns | | | Undetected | | **Very low** |
| NCR vs TAU | | | | No concerns | | | Some concerns | | | No concerns | | No concerns | | | No concerns | | | Undetected | | **Moderate** |
| NCR+12-Step vs NCR+CRA | | | | Major concerns | | | Some concerns | | | No concerns | | No concerns | | | No concerns | | | Undetected | | **Very low** |
| SEPT vs TAU | | | | No concerns | | | Some concerns | | | No concerns | | No concerns | | | No concerns | | | Undetected | | **Moderate** |
| **Indirect evidence** |  | |  | | |  | | |  | |  | | |  | | |  | | |  |
| 12-Step vs CM | | | | No concerns | | | Some concerns | | | Some concerns | | No concerns | | | No concerns | | | Undetected | | **Low** |
| 12-Step vs CM+12-Step | | | | Some concerns | | | Some concerns | | | No concerns | | No concerns | | | No concerns | | | Undetected | | **Low** |
| 12-Step vs CM+CBT | | | | No concerns | | | Some concerns | | | Some concerns | | No concerns | | | No concerns | | | Undetected | | **Low** |
| 12-Step vs CM+CRA | | | | No concerns | | | Some concerns | | | Some concerns | | No concerns | | | No concerns | | | Undetected | | **Low** |
| 12-Step vs CRA | | | | Some concerns | | | Some concerns | | | No concerns | | No concerns | | | No concerns | | | Undetected | | **Low** |
| 12-Step vs MBT | | | | Some concerns | | | Some concerns | | | No concerns | | No concerns | | | No concerns | | | Undetected | | **Low** |
| 12-Step vs NCR | | | | No concerns | | | Some concerns | | | No concerns | | No concerns | | | No concerns | | | Undetected | | **Moderate** |
| 12-Step vs NCR+12-Step | | | | Some concerns | | | Some concerns | | | No concerns | | No concerns | | | No concerns | | | Undetected | | **Low** |
| 12-Step vs NCR+CRA | | | | Some concerns | | | Some concerns | | | No concerns | | No concerns | | | No concerns | | | Undetected | | **Low** |
| CBT vs CM+12-Step | | | | Some concerns | | | Some concerns | | | No concerns | | No concerns | | | No concerns | | | Undetected | | **Low** |
| CBT vs CM+CRA | | | | Some concerns | | | Some concerns | | | Some concerns | | No concerns | | | No concerns | | | Undetected | | **Very low** |
| CBT vs NCR+12-Step | | | | Some concerns | | | Some concerns | | | No concerns | | No concerns | | | No concerns | | | Undetected | | **Low** |
| CBT vs NCR+CRA | | | | Some concerns | | | Some concerns | | | No concerns | | No concerns | | | No concerns | | | Undetected | | **Low** |
| CM vs CM+12-Step | | | | Some concerns | | | Some concerns | | | No concerns | | No concerns | | | No concerns | | | Undetected | | **Low** |
| CM vs CRA | | | | Some concerns | | | Some concerns | | | No concerns | | No concerns | | | No concerns | | | Undetected | | **Low** |
| CM vs MBT | | | | Some concerns | | | Some concerns | | | No concerns | | No concerns | | | No concerns | | | Undetected | | **Low** |
| CM vs NCR+12-Step | | | | Some concerns | | | Some concerns | | | No concerns | | No concerns | | | No concerns | | | Undetected | | **Low** |
| CM vs NCR+CRA | | | | Some concerns | | | Some concerns | | | No concerns | | No concerns | | | No concerns | | | Undetected | | **Low** |
| CM vs SEPT | | | | No concerns | | | Some concerns | | | Some concerns | | No concerns | | | No concerns | | | Undetected | | **Low** |
| CM+12-Step vs CM+CBT | | | | Some concerns | | | Some concerns | | | No concerns | | No concerns | | | No concerns | | | Undetected | | **Low** |
| CM+12-Step vs CRA | | | | Major concerns | | | Some concerns | | | No concerns | | No concerns | | | No concerns | | | Undetected | | **Very low** |
| CM+12-Step vs MBT | | | | Some concerns | | | Some concerns | | | No concerns | | No concerns | | | No concerns | | | Undetected | | **Low** |
| CM+12-Step vs NCR | | | | Some concerns | | | Some concerns | | | No concerns | | No concerns | | | No concerns | | | Undetected | | **Low** |
| CM+12-Step vs SEPT | | | | Some concerns | | | Some concerns | | | No concerns | | No concerns | | | No concerns | | | Undetected | | **Low** |
| CM+12-Step vs TAU | | | | Some concerns | | | Some concerns | | | No concerns | | No concerns | | | No concerns | | | Undetected | | **Low** |
| CM+CBT vs CM+CRA | | | | Some concerns | | | Some concerns | | | No concerns | | No concerns | | | No concerns | | | Undetected | | **Low** |
| CM+CBT vs CRA | | | | Some concerns | | | Some concerns | | | No concerns | | No concerns | | | No concerns | | | Undetected | | **Low** |
| CM+CBT vs MBT | | | | Some concerns | | | Some concerns | | | No concerns | | No concerns | | | No concerns | | | Undetected | | **Low** |
| CM+CBT vs NCR+12-Step | | | | Some concerns | | | Some concerns | | | No concerns | | No concerns | | | No concerns | | | Undetected | | **Low** |
| CM+CBT vs NCR+CRA | | | | Some concerns | | | Some concerns | | | No concerns | | No concerns | | | No concerns | | | Undetected | | **Low** |
| CM+CBT vs SEPT | | | | No concerns | | | Some concerns | | | Some concerns | | No concerns | | | No concerns | | | Undetected | | **Low** |
| CM+CRA vs MBT | | | | Some concerns | | | Some concerns | | | No concerns | | No concerns | | | No concerns | | | Undetected | | **Low** |
| CM+CRA vs NCR | | | | Some concerns | | | No concerns | | | Some concerns | | No concerns | | | No concerns | | | Undetected | | **Low** |
| CM+CRA vs SEPT | | | | No concerns | | | Some concerns | | | Some concerns | | No concerns | | | No concerns | | | Undetected | | **Low** |
| CRA vs MBT | | | | Major concerns | | | Some concerns | | | No concerns | | No concerns | | | No concerns | | | Undetected | | **Very low** |
| CRA vs NCR | | | | Some concerns | | | Some concerns | | | No concerns | | No concerns | | | No concerns | | | Undetected | | **Low** |
| CRA vs NCR+12-Step | | | | Major concerns | | | Some concerns | | | No concerns | | No concerns | | | No concerns | | | Undetected | | **Very low** |
| CRA vs NCR+CRA | | | | Major concerns | | | Some concerns | | | No concerns | | No concerns | | | No concerns | | | Undetected | | **Very low** |
| CRA vs SEPT | | | | Some concerns | | | Some concerns | | | No concerns | | No concerns | | | No concerns | | | Undetected | | **Low** |
| CRA vs TAU | | | | Some concerns | | | Some concerns | | | No concerns | | No concerns | | | No concerns | | | Undetected | | **Low** |
| MBT vs NCR | | | | Some concerns | | | Some concerns | | | No concerns | | No concerns | | | No concerns | | | Undetected | | **Low** |
| MBT vs NCR+12-Step | | | | Some concerns | | | Some concerns | | | No concerns | | No concerns | | | No concerns | | | Undetected | | **Low** |
| MBT vs NCR+CRA | | | | Some concerns | | | Some concerns | | | No concerns | | No concerns | | | No concerns | | | Undetected | | **Low** |
| MBT vs SEPT | | | | Some concerns | | | Some concerns | | | No concerns | | No concerns | | | No concerns | | | Undetected | | **Low** |
| NCR vs NCR+12-Step | | | | Some concerns | | | Some concerns | | | No concerns | | No concerns | | | No concerns | | | Undetected | | **Low** |
| NCR vs NCR+CRA | | | | Some concerns | | | Some concerns | | | No concerns | | No concerns | | | No concerns | | | Undetected | | **Low** |
| NCR vs SEPT | | | | No concerns | | | Some concerns | | | No concerns | | No concerns | | | No concerns | | | Undetected | | **Moderate** |
| NCR+12-Step vs SEPT | | | | Some concerns | | | Some concerns | | | No concerns | | No concerns | | | No concerns | | | Undetected | | **Low** |
| NCR+12-Step vs TAU | | | | Some concerns | | | Some concerns | | | No concerns | | No concerns | | | No concerns | | | Undetected | | **Low** |
| NCR+CRA vs SEPT | | | | Some concerns | | | Some concerns | | | No concerns | | No concerns | | | No concerns | | | Undetected | | **Low** |
| NCR+CRA vs TAU | | | | Some concerns | | | Some concerns | | | No concerns | | No concerns | | | No concerns | | | Undetected | | **Low** |

**S12b Table. Evaluation of the Quality of Evidence Using GRADE Framework for Dropout at the End of Treatment.**

| **Comparison** | **Study Limitations** | | **Imprecision** | **Heterogeneity** | | **Incoherence** | | **Indirectness** | | **Publication bias** | | **Confidence in OR for abstinence** |
| --- | --- | --- | --- | --- | --- | --- | --- | --- | --- | --- | --- | --- |
| **Mixed evidence** | |  |  | |  | |  | |  | |  |  |
| 12-Step vs CBT | Some concerns | | Some concerns | No concerns | | No concerns | | No concerns | | Undetected | | **Low** |
| 12-Step vs SEPT | No concerns | | Some concerns | No concerns | | No concerns | | No concerns | | Undetected | | **Moderate** |
| 12-Step vs TAU | No concerns | | Some concerns | Some concerns | | No concerns | | No concerns | | Undetected | | **Low** |
| CBT vs CM | No concerns | | Some concerns | No concerns | | No concerns | | No concerns | | Undetected | | **Moderate** |
| CBT vs CM+CBT | No concerns | | Some concerns | No concerns | | No concerns | | No concerns | | Undetected | | **Moderate** |
| CBT vs CRA | Major concerns | | Some concerns | No concerns | | No concerns | | No concerns | | Undetected | | **Very low** |
| CBT vs MBT | Major concerns | | Some concerns | Some concerns | | No concerns | | No concerns | | Undetected | | **Very low** |
| CBT vs NCR | Major concerns | | Some concerns | No concerns | | No concerns | | No concerns | | Undetected | | **Very low** |
| CBT vs SEPT | No concerns | | Some concerns | No concerns | | No concerns | | No concerns | | Undetected | | **Moderate** |
| CBT vs TAU | Some concerns | | Some concerns | No concerns | | No concerns | | No concerns | | Undetected | | **Low** |
| CM vs CM+CBT | Major concerns | | Some concerns | No concerns | | No concerns | | No concerns | | Undetected | | **Very low** |
| CM vs CM+CRA | Some concerns | | No concerns | Some concerns | | No concerns | | No concerns | | Undetected | | **Low** |
| CM vs NCR | Major concerns | | Some concerns | Some concerns | | No concerns | | No concerns | | Undetected | | **Very low** |
| CM vs TAU | No concerns | | Some concerns | No concerns | | No concerns | | No concerns | | Undetected | | **Moderate** |
| CM+12-Step vs CM+CRA | Major concerns | | Some concerns | No concerns | | No concerns | | No concerns | | Undetected | | **Very low** |
| CM+12-Step vs NCR+12-Step | Major concerns | | Some concerns | No concerns | | No concerns | | No concerns | | Undetected | | **Very low** |
| CM+12-Step vs NCR+CRA | Major concerns | | Some concerns | No concerns | | Some concerns | | No concerns | | Undetected | | **Very low** |
| CM+CBT vs NCR | Major concerns | | Some concerns | No concerns | | No concerns | | No concerns | | Undetected | | **Very low** |
| CM+CRA vs CRA | Major concerns | | Some concerns | No concerns | | No concerns | | No concerns | | Undetected | | **Very low** |
| CM+CRA vs NCR+12-Step | Major concerns | | No concerns | No concerns | | Some concerns | | No concerns | | Undetected | | **Very low** |
| CM+CRA vs NCR+CRA | Major concerns | | Some concerns | No concerns | | No concerns | | No concerns | | Undetected | | **Very low** |
| CM+CRA vs TAU | No concerns | | No concerns | No concerns | | No concerns | | No concerns | | Undetected | | **High** |
| MBT vs TAU | Major concerns | | Some concerns | No concerns | | No concerns | | No concerns | | Undetected | | **Very low** |
| NCR+12-Step vs NCR+CRA | Major concerns | | Some concerns | Some concerns | | Some concerns | | No concerns | | Undetected | | **Very low** |
| SEPT vs TAU | No concerns | | Some concerns | Some concerns | | No concerns | | No concerns | | Undetected | | **Low** |
| **Indirect evidence** | |  |  | |  | |  | |  | |  |  |
| 12-Step vs CM | No concerns | | Some concerns | No concerns | | No concerns | | No concerns | | Undetected | | **Moderate** |
| 12-Step vs CM+12-Step | Some concerns | | Some concerns | No concerns | | No concerns | | No concerns | | Undetected | | **Low** |
| 12-Step vs CM+CBT | Some concerns | | Some concerns | No concerns | | No concerns | | No concerns | | Undetected | | **Low** |
| 12-Step vs CM+CRA | Some concerns | | No concerns | No concerns | | No concerns | | No concerns | | Undetected | | **Moderate** |
| 12-Step vs CRA | Some concerns | | No concerns | No concerns | | No concerns | | No concerns | | Undetected | | **Moderate** |
| 12-Step vs MBT | Some concerns | | Some concerns | No concerns | | No concerns | | No concerns | | Undetected | | **Low** |
| 12-Step vs NCR | Some concerns | | No concerns | Some concerns | | No concerns | | No concerns | | Undetected | | **Low** |
| 12-Step vs NCR+12-Step | Some concerns | | Some concerns | No concerns | | No concerns | | No concerns | | Undetected | | **Low** |
| 12-Step vs NCR+CRA | Some concerns | | Some concerns | No concerns | | No concerns | | No concerns | | Undetected | | **Low** |
| CBT vs CM+12-Step | Some concerns | | Some concerns | No concerns | | No concerns | | No concerns | | Undetected | | **Low** |
| CBT vs CM+CRA | Some concerns | | No concerns | Some concerns | | No concerns | | No concerns | | Undetected | | **Low** |
| CBT vs NCR+12-Step | Some concerns | | Some concerns | No concerns | | No concerns | | No concerns | | Undetected | | **Low** |
| CBT vs NCR+CRA | Some concerns | | Some concerns | No concerns | | No concerns | | No concerns | | Undetected | | **Low** |
| CM vs CM+12-Step | Some concerns | | Some concerns | No concerns | | No concerns | | No concerns | | Undetected | | **Low** |
| CM vs CRA | Some concerns | | Some concerns | No concerns | | No concerns | | No concerns | | Undetected | | **Low** |
| CM vs MBT | Some concerns | | Some concerns | No concerns | | No concerns | | No concerns | | Undetected | | **Low** |
| CM vs NCR+12-Step | Some concerns | | Some concerns | No concerns | | No concerns | | No concerns | | Undetected | | **Low** |
| CM vs NCR+CRA | Some concerns | | Some concerns | No concerns | | No concerns | | No concerns | | Undetected | | **Low** |
| CM vs SEPT | No concerns | | Some concerns | No concerns | | No concerns | | No concerns | | Undetected | | **Moderate** |
| CM+12-Step vs CM+CBT | Some concerns | | Some concerns | No concerns | | No concerns | | No concerns | | Undetected | | **Low** |
| CM+12-Step vs CRA | Major concerns | | Some concerns | No concerns | | No concerns | | No concerns | | Undetected | | **Very low** |
| CM+12-Step vs MBT | Major concerns | | Some concerns | No concerns | | No concerns | | No concerns | | Undetected | | **Very low** |
| CM+12-Step vs NCR | Major concerns | | Some concerns | No concerns | | No concerns | | No concerns | | Undetected | | **Very low** |
| CM+12-Step vs SEPT | Some concerns | | Some concerns | No concerns | | No concerns | | No concerns | | Undetected | | **Low** |
| CM+12-Step vs TAU | Some concerns | | Some concerns | No concerns | | No concerns | | No concerns | | Undetected | | **Low** |
| CM+CBT vs CM+CRA | Some concerns | | No concerns | Some concerns | | No concerns | | No concerns | | Undetected | | **Low** |
| CM+CBT vs CRA | Some concerns | | Some concerns | Some concerns | | No concerns | | No concerns | | Undetected | | **Very low** |
| CM+CBT vs MBT | Some concerns | | Some concerns | No concerns | | No concerns | | No concerns | | Undetected | | **Low** |
| CM+CBT vs NCR+12-Step | Some concerns | | Some concerns | No concerns | | No concerns | | No concerns | | Undetected | | **Low** |
| CM+CBT vs NCR+CRA | Some concerns | | Some concerns | No concerns | | No concerns | | No concerns | | Undetected | | **Low** |
| CM+CBT vs SEPT | No concerns | | Some concerns | No concerns | | No concerns | | No concerns | | Undetected | | **Moderate** |
| CM+CBT vs TAU | Some concerns | | Some concerns | Some concerns | | No concerns | | No concerns | | Undetected | | **Very low** |
| CM+CRA vs MBT | Some concerns | | No concerns | No concerns | | No concerns | | No concerns | | Undetected | | **Moderate** |
| CM+CRA vs NCR | Some concerns | | Some concerns | No concerns | | No concerns | | No concerns | | Undetected | | **Low** |
| CM+CRA vs SEPT | Some concerns | | Some concerns | No concerns | | No concerns | | No concerns | | Undetected | | **Low** |
| CRA vs MBT | Major concerns | | Some concerns | No concerns | | No concerns | | No concerns | | Undetected | | **Very low** |
| CRA vs NCR | Some concerns | | Some concerns | No concerns | | No concerns | | No concerns | | Undetected | | **Low** |
| CRA vs NCR+12-Step | Major concerns | | No concerns | Some concerns | | No concerns | | No concerns | | Undetected | | **Very low** |
| CRA vs NCR+CRA | Major concerns | | Some concerns | No concerns | | No concerns | | No concerns | | Undetected | | **Very low** |
| CRA vs SEPT | Some concerns | | Some concerns | No concerns | | No concerns | | No concerns | | Undetected | | **Low** |
| CRA vs TAU | Some concerns | | No concerns | Some concerns | | No concerns | | No concerns | | Undetected | | **Low** |
| MBT vs NCR | Some concerns | | Some concerns | Some concerns | | No concerns | | No concerns | | Undetected | | **Very low** |
| MBT vs NCR+12-Step | Major concerns | | Some concerns | No concerns | | No concerns | | No concerns | | Undetected | | **Very low** |
| MBT vs NCR+CRA | Major concerns | | Some concerns | No concerns | | No concerns | | No concerns | | Undetected | | **Very low** |
| MBT vs SEPT | Some concerns | | Some concerns | No concerns | | No concerns | | No concerns | | Undetected | | **Low** |
| NCR vs NCR+12-Step | Major concerns | | Some concerns | No concerns | | No concerns | | No concerns | | Undetected | | **Very low** |
| NCR vs NCR+CRA | Some concerns | | Some concerns | No concerns | | No concerns | | No concerns | | Undetected | | **Low** |
| NCR vs SEPT | Some concerns | | Some concerns | No concerns | | No concerns | | No concerns | | Undetected | | **Low** |
| NCR vs TAU | Some concerns | | Some concerns | No concerns | | No concerns | | No concerns | | Undetected | | **Low** |
| NCR+12-Step vs SEPT | Some concerns | | Some concerns | No concerns | | No concerns | | No concerns | | Undetected | | **Low** |
| NCR+12-Step vs TAU | Some concerns | | Some concerns | No concerns | | No concerns | | No concerns | | Undetected | | **Low** |
| NCR+CRA vs SEPT | Some concerns | | Some concerns | No concerns | | No concerns | | No concerns | | Undetected | | **Low** |
| NCR+CRA vs TAU | Some concerns | | Some concerns | No concerns | | No concerns | | No concerns | | Undetected | | **Low** |
