# Supplementary material for: Comparative efficacy and acceptability of psychosocial interventions for individuals with cocaine and amphetamine addiction: A systematic review and network meta-analysis
Source: PLoS Med. 2018 Dec 26;15(12):e1002715. doi: 10.1371/journal.pmed.1002715 (PMC6306153; doi:10.1371/journal.pmed.1002715)
Supplement: S1 Text — (DOCX) [file pmed.1002715.s028.docx]

**S1 Text. Search Strategy and Results**

**1. Cochrane Drug and Alcohol Group Specialised Register search strategy**

**CDAG Specialised register (via CRSLive)**

1. ((amphetamine* OR cocaine OR diethylpropion OR ephedrine OR methylphenidate OR pemoline OR phenmetrazine OR phendimetrazine OR phenylpropanolamine OR phenilpropanolamine OR methamphetamine OR dextroamphetamine OR psychostimulant*):ti,xdi) AND (INREGISTER)
2. (counsel* OR psychoeducat* OR educat*:ti,ab,xin) AND (INREGISTER)
3. ((psychological NEAR2 (therap* OR treatment*)):ti,ab,xin) AND (INREGISTER)
4. (((social OR peer OR group) NEAR2 support) OR (self NEXT help) OR (cognitive NEAR2 (therap* OR behav*)):ti,ab,xin) AND (INREGISTER)
5. (CBT:xin) AND (INREGISTER)
6. (mindfulness OR relax* OR ((family OR couple) NEAR2 therap*):ti,ab,xin) AND (INREGISTER)
7. psycho*:xin
8. #2 OR #3 OR #4 OR #5 OR #6 OR #7
9. #1 AND #8

**2. CENTRAL search strategy**

**CENTRAL (via onlinelibrary.wiley.com)**

1. MeSH descriptor: [Substance-Related Disorders] explode all trees
2. ((psychostimulant* or polydrug* or drug* or substance) near/3 (abuse* or abusing or depend* or addict* or disorder*)):ti,ab
3. #1 or #2
4. MeSH descriptor: [Psychotherapy] explode all trees
5. psychotherap* or psychosocial or voucher or reinforcement or motivation* or contingent* or biofeedback or community or stimulation or education* or counsel*:ti,ab,kw
6. (social near/2 skill*):ti,ab
7. (coping near/2 skill):ti,ab
8. MeSH descriptor: [Counseling] explode all trees
9. (behavi* near/2 therap*):ti,ab
10. MeSH descriptor: [Reinforcement (Psychology)] explode all trees
11. (brief near intervention):ti,ab
12. (early near intervention):ti,ab
13. (minimal near intervention):ti,ab
14. (cognitive near therapy):ti,ab
15. (family near therapy):ti,ab
16. (stress near management near training):ti,ab
17. (supportive near expressive near therapy):ti,ab
18. MeSH descriptor: [Social Support] explode all trees
19. MeSH descriptor: [Case Management] explode all trees
20. (self near control near training):ti,ab
21. neurobehavioral*:ab,ti
22. #4 or #5 or #6 or #7 or #8 or #9 or #10 or #11 or #12 or #13 or #14 or #15 or #16 or #17 or #18 or #19 or #20 or #21
23. amphetamine:ti,ab,kw (Word variations have been searched)
24. diethylpropion:ti,ab,kw (Word variations have been searched)
25. methylphenidate:ti,ab,kw (Word variations have been searched)
26. pemoline:ti,ab,kw (Word variations have been searched)
27. phenmetrazine:ti,ab,kw (Word variations have been searched)
28. phendimetrazine:ti,ab,kw (Word variations have been searched)
29. phenylpropanolamine:ti,ab,kw (Word variations have been searched)
30. Phenylpropanolamine:ti,ab,kw (Word variations have been searched)
31. ephedrine:ti,ab,kw (Word variations have been searched)
32. cocaine:ti,ab,kw (Word variations have been searched)
33. #23 or #24 or #25 or #26 or #27 or #28 or #29 or #30 or #31 or #32
34. #3 and #22 and #33 in Trials

**3. MEDLINE search strategy**

**MEDLINE (via PubMed)**

1. Substance-related disorders[MeSH]
2. drug use*[tiab]
3. ((psychostimulant*[tiab] OR drug[tiab] OR substance[tiab]) AND (abuse*[tiab] OR dependence*[tiab] OR use*[tiab] OR disorder*[tiab] OR addict*[tiab]))
4. #1 OR #2 OR #3
5. Amphetamine[MeSH] OR amphetamine*[tiab] OR Diethylpropion[MeSH] OR diethylpropion*[tiab] OR methylphenidate[tiab] OR methilphenidate[tiab] OR Methylphenidate[MeSH] OR Pemoline[MeSH] OR pemoline[tiab] OR Phenmetrazine[MeSH] OR phenmetrazine[tiab] OR phendimetrazine[tiab] OR Phenylpropanolamine[MeSH] OR phenilpropanolamine[tiab] OR phenylpropanolamine[tiab] OR Ephedrine[tiab] OR Ephedrine[MeSH] OR cocaine[tiab] OR Cocaine[MeSH] OR Methamphetamine[Mesh] OR Dextroamphetamine[Mesh]
6. psychotherapy[MeSH] OR incentive*[tiab] OR voucher[tiab] OR psychotherap*[tiab] OR psychosocial*[tiab] OR behaviour therapy[tiab] OR behavior therapy[tiab] OR reinforcement[tiab] OR motivation*[tiab] OR contingent*[tiab] OR advice[tiab] OR biofeedback[tiab] OR community[tiab] OR stimulation[tiab] OR education*[tiab] OR brief intervention[tiab] OR early intervention[tiab] OR minimal intervention[tiab] OR counselling[MeSH] OR counsel*[tiab] OR cognitive therapy[tiab] OR family therapy[tiab] OR social skill[tiab] OR stress management training[tiab] OR supportive expressive therapy[tiab] OR neurobehavioral*[tiab] OR coping skill*[tiab] OR "self-control training"[tiab] OR social support[MeSH] OR relaxation techniques[MeSH] OR case management[MeSH]
7. #4 AND #5 AND #6
8. controlled clinical trial[pt]
9. random*[tiab]
10. placebo[tiab]
11. drug therapy[sh]
12. trial[tiab]
13. groups[tiab]
14. "Randomized Controlled Trial"[Publication Type]
15. #8 OR #9 OR #10 OR #11 OR #12 OR #13 OR #14
16. Animals[mh] NOT Humans[mh]
17. #15 NOT #16
18. #7 AND #17

**4. EMBASE search strategy**

**EMBASE (via embase.com)**

'drug dependence'/exp OR 'drug abuse'/exp OR ((psychostimulant* OR drug OR substance) NEAR/2 (abuse* OR dependence* OR use* OR disorder* OR addict*)):ab,ti AND ('amphetamine'/exp OR amphetamine*:ab,ti OR diethylpropion:ab,ti OR 'methylphenidate'/exp OR methylphenidate:ab,ti OR 'pemoline'/exp OR pemoline:ab,ti OR 'phenmetrazine'/exp OR 'phenmetrazine':ab,ti OR phendimetrazine:ab,ti OR 'phenylpropanolamine'/exp OR phenilpropanolamine:ab,ti OR 'ephedrine'/exp OR ephedrine:ab,ti OR 'cocaine'/exp OR cocaine:ab,ti) AND ('psychotherapy'/exp OR (social NEAR/2 skill*):ab,ti OR (coping NEAR/2 skill*):ab,ti OR 'self-control training' OR 'counseling'/exp OR counselling:ab,ti OR (behavi* NEAR/2 therapy):ab,ti OR 'reinforcement'/exp OR incentive*:ab,ti OR voucher:ab,ti OR psychotherap*:ab,ti OR psychosocial*:ab,ti OR reinforcement:ab,ti OR motivation*:ab,ti OR contingent*:ab,ti OR advice:ab,ti OR biofeedback:ab,ti OR community:ab,ti OR stimulation:ab,ti OR education*:ab,ti OR (stress NEAR/2 management):ab,ti OR (supportive NEAR/2 expressive):ab,ti) AND ('randomized controlled trial'/exp OR 'single blind procedure'/exp OR 'double blind procedure'/exp OR 'crossover procedure'/exp OR random*:ab,ti OR placebo*:ab,ti OR allocat*:ab,ti OR crossover*:ab,ti OR 'cross over':ab,ti OR trial:ti OR (doubl* NEXT/1 blind*):ab,ti) NOT ('animal experiment'/de OR 'nonhuman'/de OR 'animal'/de NOT 'human'/de

**5. CINAHL search strategy**

**CINAHL (via EBSCO HOST)**

1. (MH "Substance Use Disorders+")
2. TX(drug N3 addict*) or TX(drug N3 dependen*) or TX(drug N3 abuse*) or TX(drug N3 misus*)
3. TX(substance N3 addict*) or TX(substance N3 dependen*) or TX(substance N3 abuse*) or TX(substance N3 misus*)
4. TX(addict* OR overdos* OR intoxicat* OR abstin* OR abstain OR withdraw* OR abus* OR misus* OR disorder* OR dependen*)
5. TX(use* N2 drug) or TX(use* N2 disorder) or TX(use* N2 illicit)
6. TX(use* N2 drug) or TX(use* N2 disorder) or TX(use* N2 illicit)
7. (MM "Counseling")
8. (MH "Motivational Interviewing")
9. (MH "Psychotherapy+")
10. TI incentive* OR voucher OR psychotherap* OR psychosocial* OR reinforcement OR motivation* OR contingent* OR advice
11. AB incentive* OR voucher OR psychotherap* OR psychosocial* OR reinforcement OR motivation* OR contingent* OR advice
12. TI (contingency N1 management) OR AB (contingency N1 management)
13. TI (behaviour* N2 therapy) OR AB (behaviour* N2 therapy)
14. (MH "Reinforcement (Psychology)+")
15. MH "Clinical Trials+"
16. PT Clinical trial
17. TI clinic* N1 trial* or AB clinic* N1 trial*
18. TI ( singl* or doubl* or trebl* or tripl* ) and TI ( blind* or mask* )
19. AB ( singl* or doubl* or trebl* or tripl* ) and AB ( blind* or mask* )
20. TI randomi?ed control* trial* or AB randomi?ed control* trial*
21. MH "Random Assignment"
22. TI random* allocat* or AB random* allocat*
23. MH "Placebos"
24. TI placebo* or AB placebo*
25. MH "Quantitative Studies"
26. S15 OR S16 OR S17 OR S18 OR S19 OR S20 OR S21 OR S22 OR S23 OR S24 OR S25
27. S1 OR S2 OR S3 OR S4 OR S5 OR S6
28. (MH "Amphetamines+")
29. TI amphetamine* OR AB amphetamine*
30. TI Diethylpropion OR AB Diethylpropion
31. (MH "Methylphenidate") OR TX methylphenidate OR TX methilphenidate
32. TX pemoline
33. TI Phenmetrazine OR AB Phenmetrazine
34. MH Phenylpropanolamine OR TI phenilpropanolamine OR AB phenilpropanolamine OR TI phenylpropanolamine OR AB phenylpropanolamine
35. TX Ephedrine
36. MH Cocaine OR TI Cocaine OR AB Cocaine
37. S28 OR S29 OR S30 OR S31 OR S32 OR S33 OR S34 OR S35 OR S36
38. S27 AND S37
39. TX((psychostimulant* ) N3 (abuse* OR dependence* OR disorder* OR addict*))
40. S38 OR S39
41. S7 OR S8 OR S9 OR S10 OR S11 OR S12 OR S13 OR S14
42. S26 AND S40 AND S41

**6. Web of Science search strategy**

**WOS (via THOMSON REUTERS)**

Indexes=SCI-EXPANDED, SSCI, A&HCI, ESCI Timespan=All years

1. TOPIC: ((amphetamine* OR cocaine OR diethylpropion OR ephedrine OR methylphenidate OR pemoline OR phenmetrazine OR phendimetrazine OR phenylpropanolamine OR phenilpropanolamine OR psychostimulant*) NEAR/3 (abuse* OR depend* OR use* OR disorder* OR addict*))
2. TS= clinical trial* OR TS=research design OR TS=comparative stud* OR TS=evaluation stud* OR TS=controlled trial* OR TS=prospective stud* OR TS=random* OR TS=placebo* OR TS=(single blind*) OR TS=(double blind*)
3. #2 AND #1
4. TS=(counsel* OR psychoeducat* OR educat* OR (psychological NEAR/2 (therap* OR treatment*)) OR psychotherap* OR psychosocial* OR psychoanalytic OR ((social OR peer OR group) NEAR/2 support) OR (self NEXT help) OR (cognitive NEAR/2 (therap* OR behav*)) OR mindfulness OR relax* OR ((family OR couple) NEAR/2 therap*))
5. #4 AND #3

**7. PsycINFO search strategy**

**PsycINFO (via EBSCO HOST)**

1. DE "Clinical Trials"
2. TX random* OR TX clinical N3 trial* OR TX reserch N3 design* OR TX evaluat N3 stud* OR TX prospective* N3 stud* OR TX ( (singl* OR doubl* or tripl* or trebl*) N3 (blind* or mask* or dummy)
3. PO (animal not human)
4. S1 OR S2
5. S4 NOT S3
6. DE "Drug Dependency" OR DE "Drug Addiction"
7. TX((drug* or substance* or psychostimolant*) N3 ( use or abuse* or dependen* or disorder* or addict*))
8. S6 OR S7
9. DE "Amphetamine" OR DE "Dextroamphetamine" OR DE "Methamphetamine"
10. DE "Methylphenidate" OR TI Methylphenidate OR AB Methylphenidate
11. DE "Pemoline" OR TI Pemoline OR AB Pemoline
12. DE "Phenmetrazine" OR TI Phenmetrazine OR AB Phenmetrazine
13. TI phendimetrazine OR AB phendimetrazine
14. (DE "Ephedrine") OR TI Ephedrine OR AB Ephedrine
15. DE "Cocaine" TI cocaine OR AB cocaine
16. S9 OR S10 OR S11 OR S12 OR S13 OR S14 OR S15
17. S5 AND S8 AND S16
18. DE "Psychotherapy" OR DE "Behavior Therapy" OR DE "Brief Psychotherapy" OR DE "Brief Relational Therapy" OR DE "Cognitive Behavior Therapy" OR DE "Emotion Focused Therapy" OR DE "Experiential Psychotherapy" OR DE "Expressive Psychotherapy" OR DE "Group Psychotherapy" OR DE "Guided Imagery" OR DE"Individual Psychotherapy" OR DE "Insight Therapy" OR DE "Integrative Psychotherapy" OR DE "Interpersonal Psychotherapy" OR DE "Network Therapy" OR DE "Psychodynamic Psychotherapy" OR DE "Psychotherapeutic Counseling" OR DE "Rational Emotive Behavior Therapy" OR DE "Reality Therapy" OR DE "RelationshipTherapy" OR DE"Supportive Psychotherapy"
19. TI(Reinforcement* OR psychosocial* OR incentive* OR voucher* OR contingen* OR motivation* OR Psychotherap* OR education*) OR AB(Reinforcement* OR psychosocial* OR incentive* OR voucher* OR contingen* OR motivation* OR Psychotherap* OR education*)
20. TX (marital N2 therapy) OR TX (marital N2 behavi* N2 therapy) OR TX (community N2 reinforcement) OR TX (stress N2 management N2 training) OR TX (covert N2 sensitization) OR TX(confrontational N2 intervention*) OR TX (group N2 drug N2 counseling) OR TX (supportive N2 expressive N2 therapy) OR TX (cocaine N2 anonymou*) OR TX (neurobehavioral N2 treatment*)
21. TX (social N2 skills)
22. TX (coping N2 skills)
23. (S18 OR S19 OR S20 OR S21 OR S22)
24. S17 AND S23

We searched for ongoing clinical trials and unpublished trials via Internet searches on the following web-sites:

- ClinicalTrials.gov ([www.clinicaltrials.gov](http://www.clinicaltrials.gov/));
- World Health Organization (WHO) International Clinical Trials Registry Platform (ICTRP) ([apps.who.int/trialsearch/](http://apps.who.int/trialsearch/)).

We also used the following sources:

- References of the articles obtained by any means.
- Conference proceedings likely to contain trials relevant to the review. These included the Society for the Study of Addiction, International Harm Reduction Association and American Association for the Treatment of Opioid Dependence.
- We contacted investigators and relevant trial authors, seeking information about unpublished or incomplete trials.

| **Numbers of citations by each database** | |
| --- | --- |
| **Databases, trial registers and other sources** | **Citations** |
| **Databases:** |  |
| PubMed | 2092 |
| Central | 1075 |
| Web of Science | 1071 |
| Embase | 1181 |
| CINAHL | 670 |
| PsycINFO | 535 |
| CDAG Specialized Register | 501 |
| **Total (databases)** | **7125** |
|  |  |
| **Trial registers:** |  |
| ClinicalTrials.gov | 118 |
| World Health Organization (WHO) International Clinical Trials Registry Platform (ICTRP) ([apps.who.int/trialsearch/](http://apps.who.int/trialsearch/)). | 9 |
| **Total (trial registers)** | **127** |
|  |  |
| **Other sources:** | **9** |
|  |  |
| **Total citations:** | **7261** |
