# Supplementary material for: Comparative efficacy and acceptability of psychosocial interventions for individuals with cocaine and amphetamine addiction: A systematic review and network meta-analysis
Source: PLoS Med. 2018 Dec 26;15(12):e1002715. doi: 10.1371/journal.pmed.1002715 (PMC6306153; doi:10.1371/journal.pmed.1002715)
Supplement: S2 Text — (DOCX) [file pmed.1002715.s029.docx]

**S2 Text. Summary of Statistical Analysis and GRADE.**

The full methods and statistical analyses are presented in detail in the pre-established protocol registered on PROSPERO (CRD42017042900) and available at <https://www.crd.york.ac.uk/PROSPERO/display_record.asp?ID=CRD42017042900>.

First, we compared every treatment intervention carrying out pairwise meta-analyses using a random effects model in STATA.^1^ The relative treatment effects of the competing interventions were estimated using odds ratio (OR) for dichotomous outcomes (abstinence at 12 weeks, at the end of treatment, and at the longest follow-up after study completion; dropouts due to any cause at 12 weeks and at the end of treatment) and standardized mean difference (SMD) for continuous outcomes (the longest duration of abstinence at 12 weeks and at the end of treatment) with their 95% confidence intervals (CIs). An OR above 1 indicated that the intervention was associated with greater odds of the outcome than the comparator, while an OR below 1 indicated that the intervention was associated with lower odds of the outcome than the comparator. Therefore, for abstinence, an OR above 1 favored the first intervention versus the comparator, for dropouts an OR below 1 favored the first intervention versus the comparator. An SMD equal to zero indicated that the compared interventions had equivalent effects. For the “longest duration of abstinence” – measured in weeks-, an improvement was associated with higher values. Therefore, an SMD above zero indicated that the intervention was associated with a higher “longest duration of abstinence” than the comparator, while an SMD below zero indicated that the intervention was associated with lower “longest duration of abstinence” than the comparator. Therefore, a SMD above 0 favored the first intervention versus the comparator. We assessed statistical heterogeneity in each pairwise comparison with the τ^2^, I^2^ statistic and p value.^2^ If at least ten studies were available, we used the funnel plot and Egger’s test to detect publication bias.^2^

We performed a network meta-analysis using a random-effects model within a frequentist setting in STATA using the ’mvmeta’ command STATA routines available at http://www.mtm.uoi.gr .^1,3,4^ Network meta-analysis synthesized both direct evidence (from head to head trial comparisons) and indirect evidence (estimating the relative effectiveness between pairs of interventions even if these have never been compared directly in trials). The models enabled us to estimate the probability for each intervention to be the best for each outcome, given the relative effect sizes as estimated in the network meta-analysis. We obtained a treatment hierarchy using the surface under the cumulative ranking curve (SUCRA) and mean ranks. SUCRA is expressed as the probability of each intervention to be the best for each outcome considered.^5^ A SUCRA of 80% meant that the intervention of interest was associated with 80% of probability of being always the best without uncertainty. The highest the SUCRA for an intervention, the most the intervention is favored over the others.^6^

A key assumption of network analysis was that of coherence. Coherence in a network of interventions refers to the agreement between direct and indirect evidence on the same comparisons. Joint analysis can be misleading if the network is substantially incoherent. Incoherence can be present if the trials in the network have very different protocols and their inclusion/exclusion criteria are not comparable or may result as an uneven distribution of the effect modifiers across groups of trials that compare different treatments. We first checked for any erroneous data abstraction. Then, to evaluate the presence of incoherence locally, we used the loop-specific approach. This method evaluates the coherence assumption in each closed loop of the network separately as the difference between direct and indirect estimates for a specific comparison in the loop (incoherence factor).^7^ The magnitude of the incoherence factors and their 95% CIs were used to infer about the presence of incoherence in each loop. We assumed a common heterogeneity estimate within each loop.^8^ We also used a node-splitting approach to separate evidence on each comparison into direct and indirect evidence.^9^ We then measured global incoherence using the between-studies standard deviation (SD) (heterogeneity parameter) and compared the magnitude of the heterogeneity variance with the empirical distribution as derived by Turner et al.^10^

Coherence implies also that the studies comparing different interventions were similar in terms of these patient and study characteristics. To check this, we performed some subgroup analyses for the primary outcomes by using the following effect modifiers: Year of publication, sex ratio, mean age group, intensity of the treatment, type of stimulant, risk of bias, opioid therapy, sample size, comorbid alcohol abuse. Additionally, we performed sensitivity network meta-analyses for primary outcomes by considering: a) only trials on individuals addicted to cocaine and no other stimulant, b) only trials on individuals addicted to stimulants and on opioid substitution therapy.

Our study is reported according to the Preferred Reporting Items for Systematic Reviews Incorporating Network Meta-analyses of Health Care Interventions (PRISMA-NMA) statement extension for network meta-analysis.^11^

**GRADE quality assessment of the comparisons in the network**

We applied the GRADE for the primary outcomes (abstinence and dropout due to any cause at the end of treatment) by using the Confidence in Network Meta-Analysis Software (CINeMA).^12^ CINeMA is a software which uses the netmeta R-package for performing Network meta-analysis of the data.^13^ We assessed each network estimate according to the following criteria.

(1) Study limitation: In each direct comparison, we judged the risk of bias as low, moderate, or high considering the risk of bias assessment for the majority of the studies. We assigned numerical scores to these risk of bias judgments: 1 for low, 2 for moderate, and 3 for high risk of bias. Then, we derived the judgment for study limitations for each pairwise estimate as a weighted average of the risk of bias judgments from all direct estimates and the contribution of each direct estimate to the network estimates from the contributions matrix.

(2) Indirectness: We trust that the included studies in our review answered to the targeted research question in terms of populations, treatments, and outcomes. We have assured transitivity in our network by limiting the included studies to moderate/high addiction severity. We further ran various subgroup and sensitivity network meta-analyses and assured that they did not violate transitivity of the network. However, we are not able to evaluate the transitivity for comparisons formed by single-connected nodes. Therefore, we downgraded the network estimates for indirectness when a single-connected node (i.e. contingency management + community reinforcement approach versus community reinforcement approach) forms them and it is not possible to derive an indirect estimate from a closed loop.

(3) Inconsistency: We rated two concepts, heterogeneity and incoherence (as disagreement between direct and indirect estimates), in this domain. For heterogeneity in each pairwise estimates, we looked at the CIs and predictive intervals for each pairwise network estimates and we checked whether the conclusions did not change between CIs and predictive intervals. If conclusions changed, we downgraded the comparison. We also compared the tau^2^ for pairwise comparisons with at least two studies and with the expected tau^2^ for non-pharma interventions and subjective outcome (following Turner et al.^11^). For incoherence in direct/mixed evidence, we compared the p-value from side-splitting test and design-by-treatment test.

(4) Imprecision: We considered a clinically meaningful threshold for OR to be 0.80 or 1.25 and downgraded the estimate if the OR point estimate was 1 or more and the lower limit of its CI was below 0.80; or if the OR point estimate was less than 1 and the upper limit of its CI was above 1.25.

(5) Publication bias: We looked at the comparison-adjusted funnel plot to check the presence of overall publication bias. The line of regression is not suggestive of publication bias for the primary outcomes.

Finally, we assigned to each comparison an overall qualitative judgment based on four levels of quality of evidence: high, moderate, low, very low.

We derived the overall judgment of the quality of evidence considering the domains altogether. Intransitivity could produce incoherence, which sometimes cannot be detected in the data because it could be possibly concealed by the large heterogeneity (which is also part of the incoherence domain). Moreover, heterogeneity is responsible to a large degree for the low confidence in some comparisons due to imprecision. We considered these concepts jointly to derive the overall judgments.

**References**

Chaimani A, Mavridis D, Salanti G. A hands-on practical tutorial on performing meta-analysis with Stata. Evid Based Ment Health 2014;17(4):111-116. doi: 10.1136/eb-2014-101967 pmid: 25288685

2. Higgins JPT, Green S. Cochrane Handbook for Systematic Reviews of Interventions Version 5.1.0 [updated March 2011]. The Cochrane Collaboration, 2011. Available from www.cochrane-handbook.org.

3. White IR. Multivariate random-effects meta-regression: Updates to mvmeta. Stata J 2011;11:2555-270.

4. White IR, Barrett JK, Jackson D, Higgins JP. Consistency and inconsistency in network meta-analysis: model estimation using multivariate meta-regression. Res Synth Methods 2012;3(2):111-125. doi: 10.1002/jrsm.1045 pmid: 26062085

5. Chaimani A, Higgins JP, Mavridis D, Spyridonos P, Salanti G. Graphical tools for network meta-analysis in STATA. PLoS One 2013;8(10):e76654. doi: 10.1371/journal.pone.0076654 pmid: 24098547

6. Salanti G, Higgins JP, Ades AE, Ioannidis JP. Evaluation of networks of randomized trials. Stat Methods Med Res 2008;17(3):279-301. doi: 10.1177/0962280207080643 pmid: 17925316

7. Veroniki AA, Vasiliadis HS, Higgins JP, Salanti G. Evaluation of inconsistency in networks of interventions. Int J Epidemiol 2013;42(1):332-345. doi: 10.1093/ije/dys222 pmid: 23508418

8. Chaimani S. Visualizing assumptions and results in network meta-analysis: the network graphs package. Stata J 2015;15(4):905-950.

9. Dias S, Welton NJ, Caldwell DM, Ades AE. Checking consistency in mixed treatment comparison meta-analysis. Stat Med 2010;29(7-8):932-944. doi: 10.1002/sim.3767 pmid: 20213715

10. Turner RM, Davey J, Clarke MJ, Thompson SG, Higgins JP. Predicting the extent of heterogeneity in meta-analysis, using empirical data from the Cochrane Database of Systematic Reviews. Int J Epidemiol 2012;41(3):818-827. doi: 10.1093/ije/dys041 pmid: 22461129

11. Hutton B, Salanti G, Caldwell DM, et al. The PRISMA extension statement for reporting of systematic reviews incorporating network meta-analyses of health care interventions: checklist and explanations. Ann Intern Med 2015;162(11):777-784. doi: 10.7326/M14-2385 pmid: 26030634

12. CINeMA: Confidence in Network Meta-Analysis [Software]. Institute of Social and Preventive Medicine, University of Bern, 2017. Available from cinema.ispm.ch

13. Rücker G, Schwarzer G, Krahn U, and König J. netmeta: Network Meta-Analysis using Frequentist Methods, 2017. R package version 0.9-5. Available from https://CRAN.R-project.org/package=netmeta
