# Supplementary material for: Comparative efficacy and acceptability of psychosocial interventions for individuals with cocaine and amphetamine addiction: A systematic review and network meta-analysis
Source: PLoS Med. 2018 Dec 26;15(12):e1002715. doi: 10.1371/journal.pmed.1002715 (PMC6306153; doi:10.1371/journal.pmed.1002715)
Supplement: S3 Text — (DOCX) [file pmed.1002715.s030.docx]

**S3 Text. Full Reference List of Included Trials.**

**Carroll 1994**

- Carroll KM, Nich C, Rounsaville BJ. Differential symptom reduction in depressed cocaine abusers treated with psychotherapy and pharmacotherapy*. J Nerv Ment Dis* 1995;183(4):251-259. doi: 10.1097/00005053-199504000-00012 pmid: 7714514
- Carroll KM, Rounsaville BJ, Gordon LT, et al. Psychotherapy and pharmacotherapy for ambulatory cocaine abusers*. Arch Gen Psychiatry* 1994;51(3):177-187. doi: 10.1001/archpsyc.1994.03950030013002 pmid: 8122955
- Carroll KM, Rounsaville BJ, Nich C, Gordon LT, Wirtz PW, Gawin F. One-year follow-up of psychotherapy and pharmacotherapy for cocaine dependence. Delayed emergence of psychotherapy effects*. Arch Gen Psychiatry* 1994;51(12):989-997. doi: 10.1001/archpsyc.1994.03950120061010 pmid: 7979888

**Carroll 1998**

- Carroll KM, Nich C, Ball SA, McCance E, Frankforter TL, Rounsaville BJ. One-year follow-up of disulfiram and psychotherapy for cocaine-alcohol users: sustained effects of treatment*. Addiction* 2000;95(9):1335-1349. doi: 10.1046/j.1360-0443.2000.95913355.x pmid: 11048353
- Carroll KM, Nich C, Ball SA, McCance E, Rounsavile BJ. Treatment of cocaine and alcohol dependence with psychotherapy and disulfiram*. Addiction* 1998;93(5):713-727. doi: 10.1046/j.1360-0443.1998.9357137.x pmid: 9692270

**Carroll 2012**

- Carroll KM, Nich C, Shi JM, Eagan D, Ball SA. Efficacy of disulfiram and Twelve Step Facilitation in cocaine-dependent individuals maintained on methadone: a randomized placebo-controlled trial*. Drug Alcohol Depend* 2012;126(1-2):224-231. doi: 10.1016/j.drugalcdep.2012.05.019 pmid: 22695473

**Carroll 2014**

- Carroll KM, Kiluk BD, Nich C, et al. Computer-assisted delivery of cognitive-behavioral therapy: efficacy and durability of CBT4CBT among cocaine-dependent individuals maintained on methadone*. Am J Psychiatry* 2014;171(4):436-444. doi: 10.1176/appi.ajp.2013.13070987 pmid: 24577287
- Kiluk BD, Serafini K, Frankforter T, Nich C, Carroll KM. Only connect: The working alliance in computer-based cognitive behavioral therapy*. Behav Res Ther* 2014;63:139-146. doi: 10.1016/j.brat.2014.10.003 pmid: 25461789

**Carroll 2016**

- Carroll KM, Nich C, Petry NM, Eagan DA, Shi JM, Ball SA. A randomized factorial trial of disulfiram and contingency management to enhance cognitive behavioral therapy for cocaine dependence*. Drug Alcohol Depend* 2016;160:135-142. doi: 10.1016/j.drugalcdep.2015.12.036 pmid: 26817621

**Chen 2013**

- Chen KW, Berger CC, Gandhi D, Weintraub E, Lejuez CW. Adding integrative meditation with ear acupressure to outpatient treatment of cocaine addiction: a randomized controlled pilot study*. J Altern Complement Med* 2013;19(3):204-210. doi: 10.1089/acm.2011.0311 pmid: 23062020

**Crits-Christoph 1999**

- Barber JP, Foltz C, Crits-Christoph P, Chittams J. Therapists' adherence and competence and treatment discrimination in the NIDA Collaborative Cocaine Treatment Study*. J Clin Psychol* 2004;60(1):29-41. doi: 10.1002/jclp.10186 pmid: 14692007
- Crits-Christoph P, Siqueland L, Blaine J, et al. The National Institute on Drug Abuse Collaborative Cocaine Treatment Study. Rationale and methods*. Arch Gen Psychiatry* 1997;54(8):721-726. doi: 10.1001/archpsyc.1997.01830200053007 pmid: 9283507
- Crits-Christoph P, Siqueland L, Blaine J, et al. Psychosocial treatments for cocaine dependence: National Institute on Drug Abuse Collaborative Cocaine Treatment Study*. Arch Gen Psychiatry* 1999;56(6):493-502. doi: 10.1001/archpsyc.56.6.493 pmid: 10359461
- Najavits LM, Harned MS, Gallop RJ, et al. Six-month treatment outcomes of cocaine-dependent patients with and without PTSD in a multisite national trial*. J Stud Alcohol Drugs* 2007;68(3):353-361. pmid:17446974
- Siqueland L, Crits-Christoph P, Frank A, et al. Predictors of dropout from psychosocial treatment of cocaine dependence*. Drug Alcohol Depend* 1998;52(1):1-13. doi: 10.1016/S0376-8716(98)00039-8 pmid: 9788001
- Weiss RD, Griffin ML, Gallop RJ, et al. The effect of 12-step self-help group attendance and participation on drug use outcomes among cocaine-dependent patients*. Drug Alcohol Depend* 2005;77(2):177-184. doi: 10.1016/j.drugalcdep.2004.08.012 pmid:15664719
- Worley M, Gallop R, Gibbons MB, et al. Additional treatment services in a cocaine treatment study: level of services obtained and impact on outcome*. Am J Addict* 2008;17(3):209-217. doi: 10.1080/10550490802021994 pmid: 18463998

**Donovan 2013**

- Donovan DM, Daley DC, Brigham GS, et al. Stimulant abuser groups to engage in 12-step: a multisite trial in the National Institute on Drug Abuse Clinical Trials Network*. J Subst Abuse Treat* 2013;44(1):103-114. doi: 10.1016/j.jsat.2012.04.004 pmid: 22657748

**Dursteler-MacFarland 2013**

- Dursteler-MacFarland KM, Farronato NS, Strasser J, et al. A randomized, controlled, pilot trial of methylphenidate and cognitive-behavioral group therapy for cocaine dependence in heroin prescription*. J Clin Psychopharmacol* 2013;33(1):104-108. doi: 10.1097/JCP.0b013e31827bfff4 pmid: 23277248

**Epstein 2003**

- Epstein DH, Hawkins WE, Covi L, Umbricht A, Preston KL. Cognitive-behavioral therapy plus contingency management for cocaine use: findings during treatment and across 12-month follow-up. *Psychol Addict Behav* 2003;17(1):73-82. doi: 10.1037/0893-164X.17.1.73 pmid:12665084

**Festinger 2014**

- Festinger DS, Dugosh KL, Kirby KC, Seymour BL. Contingency management for cocaine treatment: cash vs. vouchers*. J Subst Abuse Treat* 2014;47(2):168-174. doi: 10.1016/j.jsat.2014.03.001 pmid: 24746956
- NCT01366716. Contingency management for cocaine dependence: cash versus vouchers. clinicaltrials.gov/show/NCT01366716. Accessed November 9, 2016.

**Garcia-Fernandez 2011**

- Garcia-Fernandez G, Secades-Villa R, Garcia-Rodriguez O, et al. Long-term benefits of adding incentives to the community reinforcement approach for cocaine dependence *Eur Addict Res* 2011;17(3):139-145. doi: 10.1159/000324848 pmid: 21447950
- Garcia-Fernandez G, Secades-Villa R, Garcia-Rodriguez O, Sanchez-Hervas E, Fernandez-Hermida JR, Higgins ST. Adding voucher-based incentives to community reinforcement approach improves outcomes during treatment for cocaine dependence*. Am J Addict* 2011;20(5):456-461. doi: 10.1111/j.1521-0391.2011.00154.x pmid: 21838845

**Garcia-Rodriguez 2007**

- Garcia-Rodriguez O, Secades-Villa R, Alvarez Rodriguez H, et al. Effect of incentives on retention in an outpatient treatment for cocaine addicts*. Psicothema* 2007;19(1):134-139. pmid: 17295995
- Garcia-Rodriguez O, Secades-Villa R, Higgins ST, et al. Effects of voucher-based intervention on abstinence and retention in an outpatient treatment for cocaine addiction: a randomized controlled trial*. Exp Clin Psychopharmacol* 2009;17(3):131-138. doi: 10.1037/a0015963 pmid: 19586227
- Secades-Villa R, Garcia-Rodriguez O, Higgins ST, Fernandez-Hermida JR, Carballo JL. Community reinforcement approach plus vouchers for cocaine dependence in a community setting in Spain: six-month outcomes*. J Subst Abuse Treat* 2008;34(2):202-207. doi: 10.1016/j.jsat.2007.03.006 pmid: 17512158

**Ghitza 2007**

- Ghitza UE, Epstein DH, Preston KL. Contingency management reduces injection-related HIV risk behaviors in heroin and cocaine using outpatients*. Addict Behav* 2008;33(4):593-604. doi:10.1016/j.addbeh.2007.11.009 pmid:18068905
- Ghitza UE, Epstein DH, Schmittner J, Vahabzadeh M, Lin JL, Preston KL. Effect of reinforcement probability and prize size on cocaine and heroin abstinence in prize-based contingency management*. J Appl Behav Anal* 2008;41(4):539-549. doi: 10.1901/jaba.2008.41-539 pmid: 19192858
- Ghitza UE, Epstein DH, Schmittner J, Vahabzadeh M, Lin JL, Preston KL. Randomized trial of prize-based reinforcement density for simultaneous abstinence from cocaine and heroin*. J Consult Clin Psychol* 2007;75(5):765-774. doi: 10.1037/0022-006X.75.5.765 pmid: 17907858

**Hagedorn 2013**

- Hagedorn HJ, Noorbaloochi S, Simon AB, et al. Rewarding early abstinence in Veterans Health Administration addiction clinics*. J Subst Abuse Treat* 2013;45(1):109-117. doi: 10.1016/j.jsat.2013.01.006 pmid: 23453480

**Higgins 1993**

- Higgins ST, Budney AJ, Bickel WK, Hughes JR, Foerg F, Badger G. Achieving cocaine abstinence with a behavioral approach*. Am J Psychiatry* 1993;150(5):763-769. doi: 10.1176/ajp.150.5.763 pmid: 8480823
- Higgins ST, Budney AJ, Bickel WK, Badger GJ, Foerg FE, Ogden D. Outpatient behavioral treatment for cocaine dependence: One-year outcome*. Exp Clin Psychopharmacol* 1995;3(2):205-212.

**Higgins 1994**

- Higgins ST, Budney AJ, Bickel WK, Foerg FE, Donham R, Badger GJ. Incentives improve outcome in outpatient behavioral treatment of cocaine dependence*. Arch Gen Psychiatry* 1994;51(7):568-576. doi: 10.1001/archpsyc.1994.03950070060011 pmid: 8031230

**Higgins 2000**

- Higgins ST, Wong CJ, Badger GJ, Ogden DE, Dantona RL. Contingent reinforcement increases cocaine abstinence during outpatient treatment and 1 year of follow-up*. J Consult Clin Psychol* 2000;68(1):64-72. doi: 10.1037/0022-006X.68.1.64 pmid: 10710841

**Higgins 2003**

- Higgins ST, Sigmon SC, Wong CJ, et al. Community reinforcement therapy for cocaine-dependent outpatients*. Arch Gen Psychiatry* 2003;60(10):1043-1052. doi: 10.1001/archpsyc.60.9.1043 pmid: 14557150

**Kirby 1998**

- Kirby KC, Marlowe DB, Festinger DS, Lamb RJ, Platt JJ. Schedule of voucher delivery influences initiation of cocaine abstinence*. J Consult Clin Psychol* 1998;66(5):761-767. doi: 10.1037/0022-006X.66.5.761pmid: 9803694

**Landovitz 2015**

- Landovitz RJ, Fletcher JB, Shoptaw S, Reback CJ. Contingency management facilitates the use of postexposure prophylaxis among stimulant-using men who have sex with men *Open Forum Infect Dis*. 2015;2(1):ofu114. doi: 10.1093/ofid/ofu114 pmid: 25884003

**Ledgerwood 2006**

- Ledgerwood DM, Petry NM. Does contingency management affect motivation to change substance use?*. Drug Alcohol Depend* 2006;83(1):65-72. doi: 10.1016/j.drugalcdep.2005.10.012 pmid: 16310974

**Maude-Griffin 1998**

- Maude-Griffin PM, Hohenstein JM, Humfleet GL, Reilly PM, Tusel DJ, Hall SM. Superior efficacy of cognitive-behavioral therapy for urban crack cocaine abusers: main and matching effects*. J Consult Clin Psychol* 1998;66(5):832-837. doi: 10.1037/0022-006X.66.5.832 pmid: 9803702

**McDonell 2013**

- McDonell MG, Srebnik D, Angelo F, et al. Randomized controlled trial of contingency management for stimulant use in community mental health patients with serious mental illness*. Am J Psychiatry* 2013;170(1):94-101. doi: 10.1176/appi.ajp.2012.11121831 pmid: 23138961

**McKay 1997**

- McKay JR, Alterman AI, Cacciola JS, Rutherford MJ, O'Brien CP, Koppenhaver J. Group counseling versus individualized relapse prevention aftercare following intensive outpatient treatment for cocaine dependence: initial results*. J Consult Clin Psychol* 1997;65(5):778-788. doi: 10.1037/0022-006X.65.5.778 pmid: 9337497
- McKay JR, Alterman AI, Cacciola JS, O'Brien CP, Koppenhaver JM, Shepard DS. Continuing care for cocaine dependence: comprehensive 2-year outcomes*. J Consult Clin Psychol* 1999;67(3):420-427. doi: 10.1037/0022-006X.67.3.420 pmid: 10369063

**Menza 2010**

- Menza TW, Jameson DR, Hughes JP, Colfax GN, Shoptaw S, Golden MR. Contingency management to reduce methamphetamine use and sexual risk among men who have sex with men: a randomized controlled trial*. BMC Public Health* 2010;10:774-2458-10-774. doi: 10.1186/1471-2458-10-774 pmid: 21172026

**Miguel 2016**

- Miguel AQ, Madruga CS, Cogo-Moreira H, et al. Contingency management is effective in promoting abstinence and retention in treatment among crack cocaine users in Brazil: A randomized controlled trial*. Psychol Addict Behav* 2016;30(5):536-543. doi:10.1037/adb0000192 pmid: 27442691

**Milby 2008**

- Milby JB, Schumacher JE, Vuchinich RE, Freedman MJ, Kertesz S, Wallace D. Toward cost-effective initial care for substance-abusing homeless*. J Subst Abuse Treat* 2008;34(2):180-191. doi: 10.1016/j.jsat.2007.03.003 pmid: 17512156
- Milby JB, Schumacher JE, Wallace D, Vuchinich R, Mennemeyer ST, Kertesz SG. Effects of sustained abstinence among treated substance-abusing homeless persons on housing and employment*. Am J Public Health* 2010;100(5):913-918. doi: 10.2105/AJPH.2008.152975 pmid: 19833998

**Peirce 2006**

- Peirce JM, Petry NM, Stitzer ML, et al. Effects of lower-cost incentives on stimulant abstinence in methadone maintenance treatment: a National Drug Abuse Treatment Clinical Trials Network study*. Arch Gen Psychiatry* 2006;63(2):201-208. doi: 10.1001/archpsyc.63.2.201 pmid: 16461864

**Petitjean 2014**

- Petitjean SA, Dursteler-MacFarland KM, Krokar MC, et al. A randomized, controlled trial of combined cognitive-behavioral therapy plus prize-based contingency management for cocaine dependence*. Drug Alcohol Depend* 2014;145:94-100. doi: 10.1016/j.drugalcdep.2014.09.785 pmid: 25456571
- NCT00877435. Prize reinforcement contingency management for cocaine dependence: a 24-week randomized controlled trial. clinicaltrials.gov/show/NCT00877435. Accessed March 8, 2016.

**Petry 2002**

- Petry NM, Martin B. Low-cost contingency management for treating cocaine- and opioid-abusing methadone patients*. J Consult Clin Psychol* 2002;70(2):398-405. doi: 10.1037/0022-006X.70.2.398 pmid: 11952198

**Petry 2005**

- Petry NM, Peirce JM, Stitzer ML, et al. Effect of prize-based incentives on outcomes in stimulant abusers in outpatient psychosocial treatment programs: a national drug abuse treatment clinical trials network study*. Arch Gen Psychiatry* 2005;62(10):1148-1156. doi: 10.1001/archpsyc.62.10.1148 pmid: 16203960
- Peirce JM, Petry NM, Roll JM, et al. Correlates of stimulant treatment outcome across treatment modalities*. Am J Drug Alcohol Abuse* 2009;35(1):48-53. doi: 10.1080/00952990802455444 pmid: 19152207

**Petry 2005**

- Petry NM, Martin B, Simcic F,Jr. Prize reinforcement contingency management for cocaine dependence: integration with group therapy in a methadone clinic*. J Consult Clin Psychol* 2005;73(2):354-359. doi: 10.1037/0022-006X.73.2.354 pmid: 15796645

**Petry 2007**

- Petry NM, Alessi SM, Hanson T, Sierra S. Randomized trial of contingent prizes versus vouchers in cocaine-using methadone patients*. J Consult Clin Psychol* 2007;75(6):983-991. doi: 10.1037/0022-006X.75.6.983 pmid: 18085914

**Petry 2012**

- Petry NM, Alessi SM, Ledgerwood DM. A randomized trial of contingency management delivered by community therapists*. J Consult Clin Psychol* 2012;80(2):286-298. doi: 10.1037/a0026826 pmid: 22250852

**Petry 2012**

- Petry NM, Barry D, Alessi SM, Rounsaville BJ, Carroll KM. A randomized trial adapting contingency management targets based on initial abstinence status of cocaine-dependent patients*. J Consult Clin Psychol* 2012;80(2):276-285. doi: 10.1037/a0026883 pmid: 22229758

**Petry 2013**

- Petry NM, Alessi SM, Rash CJ. A randomized study of contingency management in cocaine-dependent patients with severe and persistent mental health disorders*. Drug Alcohol Depend* 2013;130(1-3):234-237. doi: 10.1016/j.drugalcdep.2012.10.017 pmid: 23182410

**Poling 2006**

- Poling J, Oliveto A, Petry N, et al. Six-month trial of bupropion with contingency management for cocaine dependence in a methadone-maintained population*. Arch Gen Psychiatry* 2006;63(2):219-228. doi: 10.1001/archpsyc.63.2.219 pmid: 16461866

**Rawson 2002**

- Rawson RA, Huber A, McCann M, et al. A comparison of contingency management and cognitive-behavioral approaches during methadone maintenance treatment for cocaine dependence*. Arch Gen Psychiatry* 2002;59(9):817-824. doi: 10.1001/archpsyc.59.9.817 pmid: 12215081
- Farabee D, Rawson R, McCann M. Adoption of drug avoidance activities among patients in contingency management and cognitive-behavioral treatments*. J Subst Abuse Treat* 2002;23(4):343-350. doi: 10.1016/S0740-5472(02)00297-0 pmid: 12495796

**Rawson 2006**

- Rawson RA, McCann MJ, Flammino F, et al. A comparison of contingency management and cognitive-behavioral approaches for stimulant-dependent individuals. *Addiction* 2006 ;101(2):267-74. doi: 10.1111/j.1360-0443.2006.01312.x pmid: 16445555

**Roll 2013**

- Roll JM, Chudzynski J, Cameron JM, Howell DN, McPherson S. Duration effects in contingency management treatment of methamphetamine disorders*. Addict Behav* 2013;38(9):2455-2462. doi: 10.1016/j.addbeh.2013.03.018 pmid: 23708468

**Sanchez-Hervas 2010**

- Secades-Villa R, Sanchez-Hervas E, Zacares-Romaguera F, Garcia-Rodriguez O, Santonja-Gomez FJ, Garcia-Fernandez G. Community Reinforcement Approach (CRA) for cocaine dependence in the Spanish public health system: 1 year outcome*. Drug Alcohol Rev* 2011;30(6):606-612. doi: 10.1111/j.1465-3362.2010.00250.x pmid: 21355914
- Sanchez-Hervas E, Secades-Villa R, Zacares Romaguera F, Garcia-Rodriguez O, Fernandez-Hermida JR, Santonja Gomez FJ. Psychological treatment for cocaine addicts in public health services [Tratamiento psicologico para dependientes a la cocaina en la red sanitaria publica]. *Trastort Adict* 2008;10(4):275-283. doi: 10.1016/S1575-0973(08)76375-X
- Sanchez-Hervas E, Zacares-Romaguera F, Garcia-Rodriguez O, Secades-Villa R, Fernandez-Hermida JR. Community reinforcement approach (CRA) for cocaine addicts: establishment in a public health setting [Programa de reforzamiento comunitario (CRA) para adictos a la cocaína : implantación en un dispositivo sanitario pùblico]*. An Psiquiatr* 2008;24(4):153-158.

**Schottenfeld 2011**

- Schottenfeld RS, Moore B, Pantalon MV. Contingency management with community reinforcement approach or twelve-step facilitation drug counseling for cocaine dependent pregnant women or women with young children*. Drug Alcohol Depend* 2011;118(1):48-55. doi: 10.1016/j.drugalcdep.2011.02.019 pmid: 21454024
- NCT00914381. Behavioral treatment for cocaine dependent women. clinicaltrials.gov/show/NCT00914381. Accessed November/9, 2016.

**Secades Villa 2013**

- Secades-Villa R, Garcia-Fernandez G, Pena-Suarez E, Garcia-Rodriguez O, Sanchez-Hervas E, Fernandez-Hermida JR. Contingency management is effective across cocaine-dependent outpatients with different socioeconomic status*. J Subst Abuse Treat* 2013;44(3):349-354. doi: 10.1016/j.jsat.2012.08.018 pmid: 22999380

**Shoptaw 2005**

- Shoptaw S, Reback CJ, Peck JA, et al. Behavioral treatment approaches for methamphetamine dependence and HIV-related sexual risk behaviors among urban gay and bisexual men*. Drug Alcohol Depend* 2005;78(2):125-134. doi: 10.1016/j.drugalcdep.2004.10.004 pmid: 15845315
- Jaffe A, Shoptaw S, Stein J, Reback CJ, Rotheram-Fuller E. Depression ratings, reported sexual risk behaviors, and methamphetamine use: latent growth curve models of positive change among gay and bisexual men in an outpatient treatment program*. Exp Clin Psychopharmacol* 2007;15(3):301-307. doi: 10.1037/1064-1297.15.3.301 pmid:17563217

**Shoptaw 2008**

- Shoptaw S, Reback CJ, Larkins S, et al. Outcomes using two tailored behavioral treatments for substance abuse in urban gay and bisexual men*. J Subst Abuse Treat* 2008;35(3):285-293. doi: 10.1016/j.jsat.2007.11.004 pmid:18329226

**Silverman 1996**

- Silverman K, Higgins ST, Brooner RK, et al. Sustained cocaine abstinence in methadone maintenance patients through voucher-based reinforcement therapy*. Arch Gen Psychiatry* 1996;53(5):409-415. doi: 10.1001/archpsyc.1996.01830050045007 pmid: 8624184

**Silverman 1998**

- Silverman K, Wong CJ, Umbricht-Schneiter A, Montoya ID, Schuster CR, Preston KL. Broad beneficial effects of cocaine abstinence reinforcement among methadone patients*. J Consult Clin Psychol* 1998;66(5):811-824. doi: 10.1037/0022-006X.66.5.811pmid: 9803700

**Smout 2010**

- Smout MF, Longo M, Harrison S, Minniti R, Wickes W, White JM. Psychosocial treatment for methamphetamine use disorders: a preliminary randomized controlled trial of cognitive behavior therapy and Acceptance and Commitment Therapy*. Subst Abus* 2010;31(2):98-107. doi:10.1080/08897071003641578 pmid: 20408061

**Umbricht 2014**

- Umbricht A, DeFulio A, Winstanley EL, et al. Topiramate for cocaine dependence during methadone maintenance treatment: a randomized controlled trial. *Drug Alcohol Depend* 2014;140:92-100. doi: 10.1016/j.drugalcdep.2014.03.033 pmid: 24814607
- NCT00685178. Clinical Trial of Topiramate for Cocaine Addiction. clinicaltrials.gov/ct2/show/NCT00685178 Accessed May 13, 2018.
