# Supplementary material for: Comparative efficacy and acceptability of psychosocial interventions for individuals with cocaine and amphetamine addiction: A systematic review and network meta-analysis
Source: PLoS Med. 2018 Dec 26;15(12):e1002715. doi: 10.1371/journal.pmed.1002715 (PMC6306153; doi:10.1371/journal.pmed.1002715)
Supplement: S4 Text — (DOCX) [file pmed.1002715.s031.docx]

**S4 Text. Description of psychosocial interventions used in the trials.**

**1) Contingency management (CM)**

CM is a behavioural intervention that emphasizes the positive reinforcement of healthy behaviours, whereby stimulant users are rewarded when they provide drug-free urine samples.

The reward for abstinence varies between trials. In some instances, cash is given to participants^5,11,38^, but most trials use vouchers of different values to minimize the risk of patients spending cash on drugs. ^10,11,17,19,21,24,26,27,31,34,39,40,45,47,48,50^ Other studies use a lottery system whereby patients draw a token that is worth prizes of escalating values.^12-15,24,29,30,32,33,35-37,41^ Only one trial provides housing and paid work as awards.^28^

CM has also been studied in add-on to bupropion hydrochloride^38^, topiramate^50^ and placebo.^38,50^

Duration of intervention in trials included: 6-25 weeks.

**2)** **Non-contingent rewards (NCR)**

NCR is a control therapy, usually compared to CM, whereby prizes are given to the patients regardless from the submission of drug-positive or drug-free urine samples.^10,14,21,24,35,38,47,48,50^

NCR has also been studied in add-on to bupropion hydrochloride^38^, topiramate^50^ and placebo.^38^

Duration of intervention in trials included: 6-25 weeks.

**3) Cognitive behavioural therapy (CBT)**

Cognitive behavioural interventions assess and modify unhelpful cognitions and maladaptive behaviours leading to substance use. Trials in stimulant use disorders include cognitive behavioural therapy (CBT), gay-specific CBT (G-CBT) and computer based-CBT (CBT4CBT).

CBT for stimulant use disorders is divided into functional analysis and coping-skills training and has been studied in several trials.^1,2,7,9,10,23,25,39,40^

In addition to the standard cognitive-behavioural principles, G-CBT considers cultural aspects of stimulant use by men-who-have-sex-with-men, including circuit parties and sex clubs.^45,46^

CBT4CBT involves a user-friendly programme using games, cartoons, quizzes, and other interactive exercises to teach skills and strategies for avoiding drug consumption. This approach bypasses the cost and complexity of CBT training for clinicians and the lack of CBT-trained staff.^4^

CBT has also been studied in add-on to desipramine^1^, disulfiram^2,3,5^, methylphenidate^9^, and placebo.^1,3,5,9^

Duration of intervention in trials included: 8-36 weeks.

**4) Contingency management plus cognitive behavioural therapy (CM + CBT)**

Several trials use CM in add-on to CBT.^5,10,28,30,39,40,45^

The combination of CBT+CM has also been studied in add-on to disulfiram and placebo.^4^

Duration of intervention in trials included: 12-24 weeks.

**5)** **Community reinforcement approach (CRA)**

CRA is a multilayered intensive intervention that teaches drug avoidance skills, encourages lifestyle changes, gives relationship counselling, and addresses comorbid stimulant use and psychiatric disorders. It involves functional analysis and coping-skills training. Social, familial, recreational, and vocational reinforces are largely used, providing a comprehensive and supportive structure to treatment.^17,42^

Duration of intervention in trials included: 24 weeks.

**6) Contingency management plus community reinforcement approach (CM + CRA)**

Incentives such as vouchers are frequently added to improve compliance with CRA. In this combined approach, patients’ significant relatives are regularly informed of all the urinalyses results and co-operate to support them in maintaining abstinence.^12,13,16-19,42^

Duration of intervention in trials included: 24 weeks.

**7) Community reinforcement approach plus non-contingent rewards (CRA + NCR)**

Two trials study CRA in add-on to NCR ^18,43^

Duration of intervention in trials included: 24 weeks.

**8) 12-step programme (12-step)**

The 12-step is based on spiritual and relational principles applied to a fellowship of companions associated by the willingness to fight addiction to stimulants. All members commence a 12-step path, which starts with acquiring the awareness of being addicted and then accepting to surrender to a “higher Power”. The self-help group reduces social isolation and conveys support and empathy from people facing similar problems, all in complete anonymity.^2,3,8,23^

12-step has also been studied in add-on to disulfiram^2,3^, and placebo.^3^

Duration of intervention in trials included: 8-36 weeks.

**9) Contingency management plus 12-step programme (CM + 12-step)**

One trial uses 12-step in add-on to CM ^43^

Duration of intervention in trials included: 24 weeks.

**10) 12-step programme plus non-contingent rewards (12-step + NCR)**

Two trials use 12-step in add-on to NCR ^16,43^

Duration of intervention in trials included: 24 weeks.

**11) Meditation-based treatments (MBT)**

MBT include several practices whereby individuals train their minds to pay greater attention to their internal and external experiences. MBT involve body scan, yoga, tai-chi and mindfulness meditation. The common goal is to develop a non-judgmental approach to stressful experiences, leading to detachment and lower reactivity to stimuli associated with stimulant use.^49^

Duration of intervention in trials included: 12 weeks.

**12) Supportive-expressive psychodynamic therapy (SEPT)**

SEPT uses a core conflictual relationship theme based on the patients’ hopes and needs from relationships (i.e. wish), the reactions elicited from others and their experience of them (i.e. response from others), and their own reactions to the latter (i.e. response from self). Patients become progressively more conscious of the three components of the theme and therefore appreciate how their past and present relationships are linked to their cravings and relapses.^7^

Duration of intervention in trials included: 36 weeks.

**13) Treatment as usual (TAU)**

TAU is a non-specific therapy including case management and any unstructured, non-manualized, psychosocial intervention.^1-4,6-9,11,13,15,20,22,25-27,29,31-37,39,41,45^

TAU has also been studied in add-on to desipramine^1^, disulfiram^2,3^, methilphenidate^9^ and placebo.^1,3,9^

Duration of intervention in trials included: 6-36 weeks.

**References**

1. Carroll KM, Rounsaville BJ, Nich C, Gordon LT, Wirtz PW, Gawin F. One-year follow-up of psychotherapy and pharmacotherapy for cocaine dependence. Delayed emergence of psychotherapy effects. Arch Gen Psychiatry 1994;51(12):989-997. doi: 10.1001/archpsyc.1994.03950120061010 pmid: 7979888
2. Carroll KM, Nich C, Ball SA, McCance E, Rounsavile BJ. Treatment of cocaine and alcohol dependence with psychotherapy and disulfiram. Addiction 1998;93(5):713-727. doi: 10.1046/j.1360-0443.1998.9357137.x pmid: 9692270
3. Carroll KM, Nich C, Shi JM, Eagan D, Ball SA. Efficacy of disulfiram and Twelve Step Facilitation in cocaine-dependent individuals maintained on methadone: a randomized placebo-controlled trial. Drug Alcohol Depend 2012;126(1-2):224-231. doi: 10.1016/j.drugalcdep.2012.05.019 pmid: 22695473
4. Carroll KM, Kiluk BD, Nich C, et al. Computer-assisted delivery of cognitive-behavioral therapy: efficacy and durability of CBT4CBT among cocaine-dependent individuals maintained on methadone. Am J Psychiatry 2014;171(4):436-444. doi: 10.1176/appi.ajp.2013.13070987 pmid: 24577287
5. Carroll KM, Nich C, Petry NM, Eagan DA, Shi JM, Ball SA. A randomized factorial trial of disulfiram and contingency management to enhance cognitive behavioral therapy for cocaine dependence. Drug Alcohol Depend 2016;160:135-142. doi: 10.1016/j.drugalcdep.2015.12.036 pmid: 26817621
6. Chen KW, Berger CC, Gandhi D, Weintraub E, Lejuez CW. Adding integrative meditation with ear acupressure to outpatient treatment of cocaine addiction: a randomized controlled pilot study. J Altern Complement Med 2013;19(3):204-210. doi: 10.1089/acm.2011.0311 pmid: 23062020
7. Crits-Christoph P, Siqueland L, Blaine J, et al. Psychosocial treatments for cocaine dependence: National Institute on Drug Abuse Collaborative Cocaine Treatment Study. Arch Gen Psychiatry 1999;56(6):493-502. doi: 10.1001/archpsyc.56.6.493 pmid: 10359461
8. Donovan DM, Daley DC, Brigham GS, et al. Stimulant abuser groups to engage in 12-step: a multisite trial in the National Institute on Drug Abuse Clinical Trials Network. J Subst Abuse Treat 2013;44(1):103-114. doi: 10.1016/j.jsat.2012.04.004 pmid: 22657748
9. Dursteler-MacFarland KM, Farronato NS, Strasser J, et al. A randomized, controlled, pilot trial of methylphenidate and cognitive-behavioral group therapy for cocaine dependence in heroin prescription. J Clin Psychopharmacol 2013;33(1):104-108. doi: 10.1097/JCP.0b013e31827bfff4 pmid: 23277248
10. Epstein DH, Hawkins WE, Covi L, Umbricht A, Preston KL. Cognitive-behavioral therapy plus contingency management for cocaine use: findings during treatment and across 12-month follow-up. Psychol Addict Behav 2003;17(1):73-82. doi: 10.1037/0893-164X.17.1.73 pmid:12665084
11. Festinger DS, Dugosh KL, Kirby KC, Seymour BL. Contingency management for cocaine treatment: cash vs. vouchers. J Subst Abuse Treat 2014;47(2):168-174. doi: 10.1016/j.jsat.2014.03.001 pmid: 24746956
12. Garcia-Fernandez G, Secades-Villa R, Garcia-Rodriguez O, Sanchez-Hervas E, Fernandez-Hermida JR, Higgins ST. Adding voucher-based incentives to community reinforcement approach improves outcomes during treatment for cocaine dependence. Am J Addict 2011;20(5):456-461. doi: 10.1111/j.1521-0391.2011.00154.x pmid: 21838845
13. Garcia-Rodriguez O, Secades-Villa R, Alvarez Rodriguez H, et al. Effect of incentives on retention in an outpatient treatment for cocaine addicts. Psicothema. 2007;19(1):134-139. pmid: 17295995
14. Ghitza UE, Epstein DH, Schmittner J, Vahabzadeh M, Lin JL, Preston KL. Randomized trial of prize-based reinforcement density for simultaneous abstinence from cocaine and heroin. J Consult Clin Psychol 2007;75(5):765-774. doi: 10.1037/0022-006X.75.5.765 pmid: 17907858
15. Hagedorn HJ, Noorbaloochi S, Simon AB, et al. Rewarding early abstinence in Veterans Health Administration addiction clinics. J Subst Abuse Treat 2013;45(1):109-117. doi: 10.1016/j.jsat.2013.01.006 pmid: 23453480
16. Higgins ST, Budney AJ, Bickel WK, Hughes JR, Foerg F, Badger G. Achieving cocaine abstinence with a behavioral approach. Am J Psychiatry 1993;150(5):763-769. doi: 10.1176/ajp.150.5.763 pmid: 8480823
17. Higgins ST, Budney AJ, Bickel WK, Foerg FE, Donham R, Badger GJ. Incentives improve outcome in outpatient behavioral treatment of cocaine dependence. Arch Gen Psychiatry 1994;51(7):568-576. doi: 10.1001/archpsyc.1994.03950070060011 pmid: 8031230
18. Higgins ST, Wong CJ, Badger GJ, Ogden DE, Dantona RL. Contingent reinforcement increases cocaine abstinence during outpatient treatment and 1 year of follow-up. J Consult Clin Psychol 2000;68(1):64-72. doi: 10.1037/0022-006X.68.1.64 pmid: 10710841
19. Higgins ST, Sigmon SC, Wong CJ, et al. Community reinforcement therapy for cocaine-dependent outpatients. Arch Gen Psychiatry 2003;60(10):1043-1052. doi: 10.1001/archpsyc.60.9.1043 pmid: 14557150
20. Kirby KC, Marlowe DB, Festinger DS, Lamb RJ, Platt JJ. Schedule of voucher delivery influences initiation of cocaine abstinence. J Consult Clin Psychol 1998;66(5):761-767. doi: 10.1037/0022-006X.66.5.761 pmid: 9803694
21. Landovitz RJ, Fletcher JB, Shoptaw S, Reback CJ. Contingency management facilitates the use of postexposure prophylaxis among stimulant-using men who have sex with men. Open Forum Infect Dis 2015;2(1):ofu114. doi: 10.1093/ofid/ofu114 doi: 10.1093/ofid/ofu114 pmid: 25884003
22. Ledgerwood DM, Petry NM. Does contingency management affect motivation to change substance use?. Drug Alcohol Depend 2006;83(1):65-72. doi: 10.1016/j.drugalcdep.2005.10.012 pmid: 16310974
23. Maude-Griffin PM, Hohenstein JM, Humfleet GL, Reilly PM, Tusel DJ, Hall SM. Superior efficacy of cognitive-behavioral therapy for urban crack cocaine abusers: main and matching effects. J Consult Clin Psychol 1998;66(5):832-837. doi: 10.1037/0022-006X.66.5.832 pmid: 9803702
24. McDonell MG, Srebnik D, Angelo F, et al. Randomized controlled trial of contingency management for stimulant use in community mental health patients with serious mental illness. Am J Psychiatry 2013;170(1):94-101. doi: 10.1176/appi.ajp.2012.11121831 pmid: 23138961
25. McKay JR, Alterman AI, Cacciola JS, Rutherford MJ, O'Brien CP, Koppenhaver J. Group counseling versus individualized relapse prevention aftercare following intensive outpatient treatment for cocaine dependence: initial results. J Consult Clin Psychol 1997;65(5):778-788. doi: 10.1037/0022-006X.65.5.778 pmid: 9337497
26. Menza TW, Jameson DR, Hughes JP, Colfax GN, Shoptaw S, Golden MR. Contingency management to reduce methamphetamine use and sexual risk among men who have sex with men: a randomized controlled trial. BMC Public Health 2010;10:774-2458-10-774. doi: 10.1186/1471-2458-10-774 pmid: 21172026
27. Miguel AQ, Madruga CS, Cogo-Moreira H, et al. Contingency management is effective in promoting abstinence and retention in treatment among crack cocaine users in Brazil: A randomized controlled trial. Psychol Addict Behav 2016;30(5):536-543. doi:10.1037/adb0000192 pmid: 27442691
28. Milby JB, Schumacher JE, Vuchinich RE, Freedman MJ, Kertesz S, Wallace D. Toward cost-effective initial care for substance-abusing homeless. J Subst Abuse Treat 2008;34(2):180-191. doi: 10.1016/j.jsat.2007.03.003 pmid: 17512156
29. Peirce JM, Petry NM, Stitzer ML, et al. Effects of lower-cost incentives on stimulant abstinence in methadone maintenance treatment: a National Drug Abuse Treatment Clinical Trials Network study. Arch Gen Psychiatry 2006;63(2):201-208. doi: 10.1001/archpsyc.63.2.201 pmid: 16461864
30. Petitjean SA, Dursteler-MacFarland KM, Krokar MC, et al. A randomized, controlled trial of combined cognitive-behavioral therapy plus prize-based contingency management for cocaine dependence. Drug Alcohol Depend 2014;145:94-100. doi: 10.1016/j.drugalcdep.2014.09.785 pmid: 25456571
31. Petry NM, Martin B. Low-cost contingency management for treating cocaine- and opioid-abusing methadone patients. J Consult Clin Psychol 2002;70(2):398-405. doi: 10.1037/0022-006X.70.2.398 pmid: 11952198
32. Petry NM, Peirce JM, Stitzer ML, et al. Effect of prize-based incentives on outcomes in stimulant abusers in outpatient psychosocial treatment programs: a national drug abuse treatment clinical trials network study. Arch Gen Psychiatry 2005;62(10):1148-1156. doi: 10.1001/archpsyc.62.10.1148 pmid: 16203960
33. Petry NM, Martin B, Simcic F,Jr. Prize reinforcement contingency management for cocaine dependence: integration with group therapy in a methadone clinic. J Consult Clin Psychol 2005;73(2):354-359. doi: 10.1037/0022-006X.73.2.354 pmid: 15796645
34. Petry NM, Alessi SM, Hanson T, Sierra S. Randomized trial of contingent prizes versus vouchers in cocaine-using methadone patients. J Consult Clin Psychol 2007;75(6):983-991. doi: 10.1037/0022-006X.75.6.983 pmid: 18085914
35. Petry NM, Alessi SM, Ledgerwood DM. A randomized trial of contingency management delivered by community therapists. J Consult Clin Psychol 2012;80(2):286-298. doi: 10.1037/a0026826 pmid: 22250852
36. Petry NM, Barry D, Alessi SM, Rounsaville BJ, Carroll KM. A randomized trial adapting contingency management targets based on initial abstinence status of cocaine-dependent patients. J Consult Clin Psychol 2012;80(2):276-285. doi: 10.1037/a0026883 pmid: 22229758
37. Petry NM, Alessi SM, Rash CJ. A randomized study of contingency management in cocaine-dependent patients with severe and persistent mental health disorders. Drug Alcohol Depend 2013;130(1-3):234-237. doi: 10.1016/j.drugalcdep.2012.10.017 pmid: 23182410
38. Poling J, Oliveto A, Petry N, et al. Six-month trial of bupropion with contingency management for cocaine dependence in a methadone-maintained population. Arch Gen Psychiatry 2006;63(2):219-228. doi: 10.1001/archpsyc.63.2.219 pmid: 16461866
39. Rawson RA, Huber A, McCann M, et al. A comparison of contingency management and cognitive-behavioral approaches during methadone maintenance treatment for cocaine dependence. Arch Gen Psychiatry 2002;59(9):817-824. doi: 10.1001/archpsyc.59.9.817 pmid: 12215081
40. Rawson RA, McCann MJ, Flammino F, et al. A comparison of contingency management and cognitive-behavioral approaches for stimulant-dependent individuals. Addiction 2006;101(2):267-74. doi: 10.1111/j.1360-0443.2006.01312.x pmid: 16445555
41. Roll JM, Chudzynski J, Cameron JM, Howell DN, McPherson S. Duration effects in contingency management treatment of methamphetamine disorders. Addict Behav 2013;38(9):2455-2462. doi: 10.1016/j.addbeh.2013.03.018 pmid: 23708468
42. Secades-Villa R, Sanchez-Hervas E, Zacares-Romaguera F, Garcia-Rodriguez O, Santonja-Gomez FJ, Garcia-Fernandez G. Community Reinforcement Approach (CRA) for cocaine dependence in the Spanish public health system: 1 year outcome. Drug Alcohol Rev 2011;30(6):606-612. doi: 10.1111/j.1465-3362.2010.00250.x pmid: 21355914
43. Schottenfeld RS, Moore B, Pantalon MV. Contingency management with community reinforcement approach or twelve-step facilitation drug counseling for cocaine dependent pregnant women or women with young children. Drug Alcohol Depend 2011;118(1):48-55. doi: 10.1016/j.drugalcdep.2011.02.019 pmid: 21454024
44. Secades-Villa R, Garcia-Fernandez G, Pena-Suarez E, Garcia-Rodriguez O, Sanchez-Hervas E, Fernandez-Hermida JR. Contingency management is effective across cocaine-dependent outpatients with different socioeconomic status. J Subst Abuse Treat 2013;44(3):349-354. doi: 10.1016/j.jsat.2012.08.018 pmid: 22999380
45. Shoptaw S, Reback CJ, Peck JA, et al. Behavioral treatment approaches for methamphetamine dependence and HIV-related sexual risk behaviors among urban gay and bisexual men. Drug Alcohol Depend 2005;78(2):125-134. doi: 10.1016/j.drugalcdep.2004.10.004 pmid: 15845315
46. Shoptaw S, Reback CJ, Larkins S, et al. Outcomes using two tailored behavioral treatments for substance abuse in urban gay and bisexual men. J Subst Abuse Treat 2008;35(3):285-293. doi: 10.1016/j.jsat.2007.11.004 pmid:18329226
47. Silverman K, Higgins ST, Brooner RK, et al. Sustained cocaine abstinence in methadone maintenance patients through voucher-based reinforcement therapy. Arch Gen Psychiatry 1996;53(5):409-415. doi: 10.1001/archpsyc.1996.01830050045007 pmid: 8624184
48. Silverman K, Wong CJ, Umbricht-Schneiter A, Montoya ID, Schuster CR, Preston KL. Broad beneficial effects of cocaine abstinence reinforcement among methadone patients. J Consult Clin Psychol 1998;66(5):811-824. doi: 10.1037/0022-006X.66.5.811pmid: 9803700
49. Smout MF, Longo M, Harrison S, Minniti R, Wickes W, White JM. Psychosocial treatment for methamphetamine use disorders: a preliminary randomized controlled trial of cognitive behavior therapy and Acceptance and Commitment Therapy. Subst Abus 2010;31(2):98-107. doi:10.1080/08897071003641578 pmid: 20408061
50. Umbricht A, DeFulio A, Winstanley EL, et al. Topiramate for cocaine dependence during methadone maintenance treatment: a randomized controlled trial. Drug Alcohol Depend 2014;140:92-100. doi: 10.1016/j.drugalcdep.2014.03.033 pmid: 24814607.

**Legend**

| **Intervention** | **Abbreviation** |
| --- | --- |
| Cognitive behavioural therapy | CBT |
| Contingency management | CM |
| Contingency management plus cognitive behavioural therapy | CM + CBT |
| Contingency management plus community reinforcement approach | CM + CRA |
| Contingency management plus twelve step programme | CM + 12 step |
| Community reinforcement approach | CRA |
| Community reinforcement approach plus non-contingent rewards | CRA + NCR |
| Meditation based treatments | MBT |
| Non-contingent rewards | NCR |
| Supportive-expressive psychodynamic therapy | SEPT |
| Treatment as usual | TAU |
| Twelve step programme | 12 step |
| Twelve step programme plus non-contingent rewards | 12 step + NCR |
